# High-Definition Brain Network (HDBN) Delineation of CDKL5 Deficiency Disorder (CDD) in Genetically Engineered Mice

**DOI:** 10.3390/biom16050652

**Published:** 2026-04-28

**Authors:** Dalton West, Noah William Coulson, Devin Raine Everaldo Cortes, Kristina Elsa Schwab, Thomas Becker-Szurszewski, Sean Hartwick, Margaret Caroline Stapleton, Gabriella Marie Saladino, Cecilia Wen-Ya Lo, Christina M. Patterson, Subramanian Subramanian, Deepa Soundara Rajan, Yijen Lin Wu

**Affiliations:** 1 Department of Pediatrics, School of Medicine, University of Pittsburgh, Pittsburgh, PA 15219, USA; daw253@pitt.edu (D.W.); nwc15@pitt.edu (N.W.C.); drc76@pitt.edu (D.R.E.C.); kes234@pitt.edu (K.E.S.); thb72@pitt.edu (T.B.-S.); hartwick@pitt.edu (S.H.); maggie.stapleton10@gmail.com (M.C.S.); gabisaladino@gmail.com (G.M.S.); cel36@pitt.edu (C.W.-Y.L.); pattersoncm3@upmc.edu (C.M.P.); rajands@upmc.edu (D.S.R.); 2 Department of Bioengineering, Swanson School of Engineering, University of Pittsburgh, Pittsburgh, PA 15261, USA; 3 Animal Imaging Core, Rangos Research Center, UPMC Children’s Hospital of Pittsburgh, Pittsburgh, PA 15224, USA; 4 UPMC Children’s Hospital of Pittsburgh, Pittsburgh, PA 15224, USA; subramanian.subramanian2@chp.edu; 5 Department of Radiology, School of Medicine, University of Pittsburgh, Pittsburgh, PA 15260, USA

**Keywords:** CDKL5, CDD, MRI, neurodevelopment, brain network, diffusion tensor imaging

## Abstract

Cyclin-Dependent Kinase-Like 5 (CDKL5) Deficient Disorder (CDD) is a rare X-linked developmental and epileptic encephalopathy characterized by early-onset refractory epilepsy, severe neurodevelopmental impairment, and lifelong disability. Although more than thirty anti-seizure medications are available, most CDD patients remain pharmaco-resistant. Gene-based therapies are emerging, but therapeutic development is hindered by marked clinical heterogeneity, small patient populations, and the lack of robust, translatable brain-based biomarkers for clinical trials. Genetically engineered *Cdkl5* mouse models recapitulate many cognitive, behavioral, and molecular features of CDD, yet their utility is limited by the absence of overt seizures, precluding seizure-based outcome measures. Here, we establish high-definition brain network (HDBN) biomarkers using advanced diffusion MRI tractography combined with graph-theoretical analysis to quantify whole-brain network organization in *Cdkl5* knockout mice. Diffusion MRI enables non-invasive mapping of axonal connectivity by leveraging anisotropic water diffusion, while high-angular-resolution acquisition overcomes key limitations of conventional diffusion tensor imaging in regions with complex fiber architecture. We demonstrate that *Cdkl5* knockout mice exhibit reproducible and region-specific disruptions in brain network organization, prominently affecting the somatosensory and somatomotor cortex, hippocampus, hypothalamus, amygdala, and superior colliculus—regions implicated in cognition, learning and memory, homeostasis, anxiety, and visual–motor function. In contrast, networks within the entorhinal cortex remain largely preserved. These findings identify HDBN metrics as sensitive, non-invasive biomarkers that capture clinically relevant circuit-level abnormalities in CDD. Because diffusion MRI–based network analyses are directly translatable across species, HDBN biomarkers provide a unified framework for therapeutic evaluation in mouse models, large animals, and human clinical trials, enabling longitudinal monitoring of disease progression and treatment response.

## 1. Introduction

Cyclin-Dependent Kinase-Like 5 (CDKL5) Deficient Disorder (CDD) [1,2,3,4], affecting ~2.36 per 100,000 live births, is an X-linked (*Xp22.13*, OMIM #300203) [5,6,7,8,9] developmental and epileptic encephalopathy (DEE2, OMIM #300672) characterized by early-onset refractory epilepsy, hypotonia, cortical visual impairments, global developmental delay, and severe intellectual disabilities [1,2,3,4,10,11,12]. CDD and Rett syndrome show substantial clinical and biological overlaps and were considered an early-onset variant [10,13,14]. However, due to different genetic mutations (CDD—*CDKL5*, Rett—*MECP2*), they are now recognized as distinct but related DEE within the Rett spectrum [10,15]. The *CDKL5* gene encodes a serine/threonine protein kinase active in the brain and is essential for both neurodevelopment and normal neuronal function [10,16], as well as synaptic signaling and cellular division [10]. Despite the availability of more than thirty anti-seizure medications (ASMs) [17], most CDD patients are resistant and continue to experience frequent sequent seizures [3,18]. No effective cure is available [19]. Even Ztalmy (ganaxolone)—an ASM for CDD-associated seizures that was approved by the Food and Drug Administration (FDA) in 2022—does not eliminate the high disease burden for health care systems [20,21] and caregivers [22,23,24]. Even if seizures can sometimes be controlled, CDD patients still suffer neurodevelopmental deficits (NDD), greatly impacting their quality of life. There is an unmet clinical need for new CDD intervention. Several gene therapy [25,26,27,28,29,30] efforts are underway to treat CDD. However, CDD patients present highly heterogeneous [10,11,31] clinical manifestations and varied temporal progression courses. Compounded with low prevalence and small patient populations [32], these make clinical trials for therapeutic development for CDD very challenging. With the only current available biomarkers, a measurable trait, being invasive EEG monitoring or genetic testing [33], there is a lack of robust brain-based biomarkers for CDD clinical trials.

Genetically engineered mouse (GEM) models are indispensable for modeling human diseases for mechanistical understanding and therapeutic development. *Cdkl5* knockout (KO) [34] and knock-in (KI) [35] mice have existed for several years. *Cdkl5* mice recapitulated some cognitive and behavioral deficits [36,37] similar to those in CDD patients, as well as molecular and pathological features [38,39]. However, their pre-clinical utility for developing new therapeutics has been hampered by a key deficit—*Cdkl5* KO or KI mice do not exhibit overt seizures as CDD patients do, thus one is unable to use seizures for therapeutic screening. Although some aged *Cdkl5* mice can display heterogenous epileptic spasm [40] and age-related cognitive and motor declines [41,42], it is inappropriate to use aging phenotypes for neurodevelopmental investigations, because aged wild-type (WT) mice can also display epileptic spasm [43]. There is a major gap of a robust, brain-based biomarker for utilizing *Cdkl5* mice for therapeutic development. Although large animal models of CDD which may exhibit seizures are being developed, there are no inbred strains for pigs or non-human primates, resulting in heterogeneous and variable manifestations of CDD symptoms similar to humans. The same challenge of human clinical trials remains in the large animal models of CDD.

The goal of this study is to establish clinically relevant brain-based biomarkers that will enable therapeutic testing of not only *Cdkl5* KO mice, but of large animals and human clinical trials as well. Similar to human CDD patients, *Cdkl5* KO mice exhibit impairments in spatial learning and memory and anxiety- and fear-related behaviors [36]. Cognitive functions are carried by unique organizations of the brain network, the “information highways,” to process signals and information in the brain. We hypothesize that *Cdkl5* KO mice and CDD patients have altered brain network organization, resulting in cognitive deficits. This notion is consistent with the observation that loss of glutamatergic neurons in the hippocampus of *Cdkl5* KO mice disrupted the hippocampal micro-circuitry, leading to impaired spatial learning and memory [39].

Diffusion MRI tractography is a non-invasive way to map neuronal connections in the entire brain. Diffusion MRI [44,45,46,47,48,49,50,51,52] leverages anisotropic water diffusion properties in the biological tissue to generate MRI contrast [53,54], providing a non-invasive way to map white matter connections. In neuronal fibers, water molecules can readily diffuse along neuronal fiber orientation, but are very limited perpendicular to the neuronal fiber orientation due to diffusion barriers of cell membranes and myeline sheath [55,56,57,58], allowing non-invasive mapping of neuronal track orientations and connections in the brain. Diffusion tractography models water diffusion patterns in neuronal axons as a Gaussian distribution, and the determination of axonal direction by the principal direction of the tensor [44,45]. Based on this principal direction, the trajectories of the axonal connections can be tracked by diffusion fiber tractography, a computational approach which reveals the axonal orientations and connections between brain areas [59,60].

Diffusion tensor imaging (DTI) [44,45] is the most widely used diffusion MRI model but has key limitations, particularly its inability to capture complex fiber orientations [61]. To address this, we use high-definition diffusion MRI with high-angular resolution and graph theory-based brain network analysis to develop high-definition brain networks (HDBNs) in Cdkl5 KO mice. These HDBNs align with observed cognitive and behavioral deficits. Because this approach is noninvasive and applicable to humans and large animals, HDBN-based biomarkers have strong potential for clinical translation, including monitoring disease progression and evaluating gene or drug therapies.

Our data demonstrate that HDBN can sensitively delineate brain network architecture in *Cdkl5* KO mice, to distinguish them from WT mice. *Cdkl5* KO mice displayed markedly altered neuronal network organization in the somatosensory and somatomotor cortices, hippocampus, hypothalamus, amygdala, and superior colliculus. These are the brain regions involved in cognition, learning and memory, homeostasis, anxiety and fear condition, and visual/motor functions. This is consistent with the clinical presentations of CDD patients with cognitive, learning and memory deficits, anxiety, and cortical visual impairments. On the other hand, the entorhinal cortex, part of the olfactory circuitry, appears to be normal.

HDBN can sensitively detect the brain network in CDD mouse models correlated with clinical presentations of CDD patients. As HDBN characteristics are qualitative and reproducible, it can potentially be a brain-based biomarker for therapeutic development in large animal models and clinical trials.

## 2. Materials and Methods

### 2.1. Animals

All animals received humane care in compliance with the NIH Office of Laboratory Animal Welfare (OLAW) guidelines. Animal protocols were approved by the University of Pittsburgh Institutional Animal Care and Use Committee (IACUC Protocol # 25066689, approval date: 4 June 2025). Mice were provided with ad libitum water and chow. *Cdkl5* [34] breeding pairs were obtained from the Jackson Laboratory (B6.129(FVB)-*Cdkl5^tm1.1Joez^*/J, Jax Strain #: 021967) as well as BL6/J (C57BL/6J, Jax Strain # 000664). Hemizygous *Cdkl5* males (Y/-, n = 12) and male BL6/J (n = 8) were included in the study. All animals were weaned on post-natal day p28. Male littermates were housed with 2–4 littermates per cage on a 12:12 h dark/light schedule. Brains were harvested for diffusion MRI at 3.5–5 months. Animals were euthanized with 5% isoflurane followed by cervical dislocation. The heart was exposed with a chest incision through the ribs. The brain was then perfused with 7 mL of 4% paraformaldehyde (PFA) via cardiac perfusion. The brain was then harvested and placed in 4% PFA for at least two days at 4 °C. The brain was then placed in phosphate-buffered saline (PBS) at 4 °C for two days for re-hydration before imaging. The ex vivo imaging allowed for longer imaging to capture, allowing for a much higher spatial resolution.

### 2.2. High-Resolution Diffusion and Anatomical MRI Acquisition

#### 2.2.1. MRI Sample Preparation

Well-fixed mouse brains were transferred to a custom-made MRI holder immersed in Fomblin-Y (perfluoropolyether, Milipore Sigma (St. Louis, MO, USA), HVAC 18/8, average molecular weight 1800) to eliminate susceptibility artifact at the air–tissue interface and background proton signals. High-resolution MRI was acquired on a Bruker Biospec 7T/30 system (Bruker Biospin MRI, Billerica, MA, USA) with a 35 mm quadrature coil for both signal transmission and reception. Two mouse brains are scanned at the same time.

#### 2.2.2. High-Resolution 3D Isotropic Diffusion MRI Acquisition

High-resolution 3D isotropic diffusion MRI images were acquired with spin echo diffusion preparation, field of view (FOV) = 40 mm × 11 mm × 11 mm, acquisition matrix = 256 × 70 × 70, voxel size = 0.003796 mm^3^, repetition time (TR) = 1000 ms, echo time (TE) = 16.665 ms, diffusion gradient duration (d) = 4 ms, diffusion gradient separation (D) = 8 ms, diffusion directions = 30, b = 1200 s/mm^2^.

#### 2.2.3. High-Resolution 3D Isotropic Anatomical MRI

High-resolution 3D anatomical MRI was acquired for co-registration of diffusion MRI to the atlas space. Three-dimensional T_2_-weighted isotropic anatomical MRI was acquired with 3D Rapid Imaging with Relaxation Enhancement (RARE), a Fast Spin-Echo (FSE) sequence, with exactly the same geometry as the diffusion scans, for anatomical registration in the same brain space with the following parameters: FOV = 40 mm × 11 mm × 11 mm, matrix size = 512 × 141 × 141, TR = 1000 ms, TE = 12 ms, RARE factor = 8, effective TE = 48 ms, refocusing flip angle 180 degrees.

### 2.3. Mouse Brain Template

Two ex vivo mouse brains underwent Diffusion MRI at the same time, so each of the diffusion-weighed imaging (DWI) Nifti volumes contained two mouse brains. The first volume of the DWI data was used to detect each mouse brain via watershed segmentation and a manually guided segmentation approach. Based on this segmentation for multiple mouse brains, the DWI data of each animal were stored in separate 4D Nifti files.

### 2.4. Atlas-Based Segmentation

**Figure 1:**
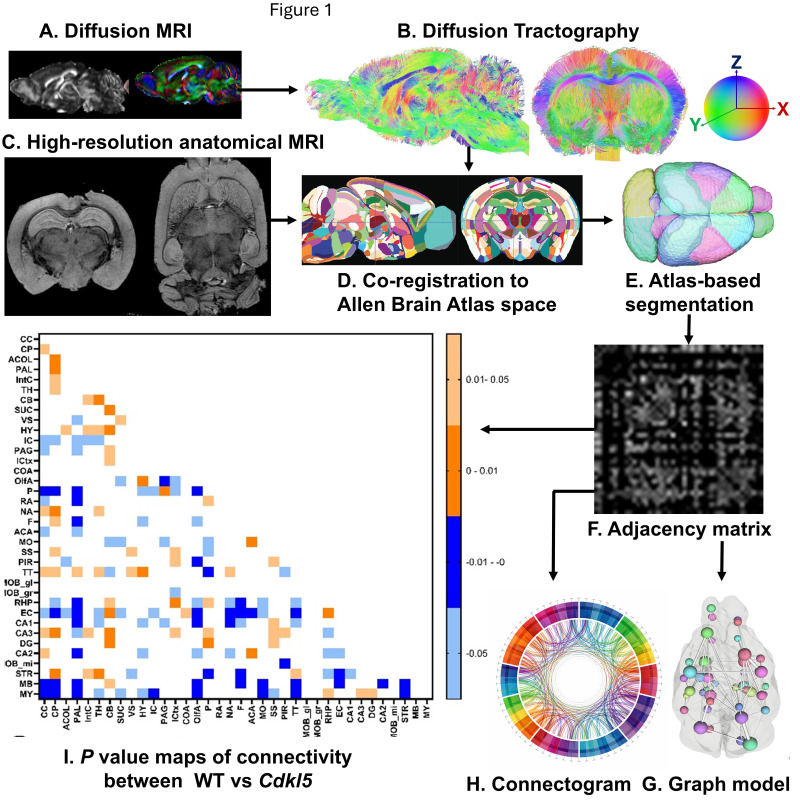
Flow diagram of diffusion MRI tractography and high-definition brain network (HDBN) delineation. (**A**) Representative images of diffusion MRI. (**B**) High-definition 3D diffusion tractography generated from diffusion MRI with fiber directionality represented by color (red being left-right. blue is front-back, and green representing top-bottom). (**C**) Parallel acquisition of high-resolution T_2_-weighted anatomical MRI with the same geometry as diffusion MRI. (**D**) Co-registration of diffusion tractography and high-resolution anatomical MRI the Allen brain atlas space to perform (**E**) Atlas based segmentation. (**F**) Adjacency matrix that is generated from a combination of tractography through various regions of the atlas-based segmentation. (**G**) Brain topology and (**H**) connectogram generated from adjacency matrix. (**I**) Group comparisons between WT and *Cdkl5* KO mice to generate *p*-value matrix for brain region connectivity that are significantly different in the adjacency matrixes.

We have established an automatic atlas-based segmentation pipeline for mouse brains. The 3D volumes of high-resolution RARE anatomical MRI (Figure 1C) and diffusion MRI (Figure 1A) are first registered to the Allen mouse brain atlas [62,63,64] space as described previously [65] using ANTx2, a custom MATLAB 2018a toolbox and segmented into gray matter (GM), white matter (WM), and cerebrospinal fluid (CSF) tissue maps using the Unified Segmentation approach [66] as implemented in statistical parametric maps (SPM) [67]. For the segmentation task, the tissue probability maps (TPMs) are generated based on Hikishima et al. [68] tissue classification. A weighted image is constructed using the tissue segments of the animal and the TPMs of the template. The weighted images are co-registered using affine and nonlinear B-spline transformation via the Elastix package [69]. The resulting parameters for forward transformation are stored to allow a subsequent image transformation from native animal space to mouse template space. Parameter files for inverse transformation are also generated and stored to allow a subsequent image transformation from template space to native space (e.g., hemispheric brain mask). Using the files for inverse transformation, we transform the template to the native, 1st volume of the 3D data to create the final brain segmentation mask. Each mouse brain is parcellated into 72 regions without assigning any region of interest (ROI) or region of avoidance (ROA). Abbreviations for each brain region are listed in Table S1.

### 2.5. Diffusion Tractography

Quantification of fiber tracking (Figure 1B) was completed using the DSI studio June 2018 version, with a minimum fiber track length of 0, maximum fiber track length of 300 mm, tracking algorithm RK4, angular threshold 0, and a total of 1,000,000 seeds calculated using a 0.07710 threshold which was recommended within the program. These parameters were used for whole-brain seeding, left hemisphere fiber tracking, and right hemisphere fiber tracking. Seeds were placed on an entire hemisphere and the corresponding hemisphere atlas added to see where, of the 36 regions, the fiber tracts mapped. This allowed us to assess the amount of fiber tracts in each region. The process was repeated for the right hemisphere. We investigated both the ipsilateral and contralateral fiber tracking to see if the left and right hemispheres were comparable. For the contralateral tracking, seeds were placed in the left hemisphere and compared to the right hemisphere’s fiber numbers. This was then repeated with the opposite hemispheres. After performing the ipsilateral and contralateral fiber tracking, we ultimately found them to not be significant and instead combined both into whole-brain fiber tracking. The whole-brain fiber tracking was performed on predetermined regions of interest that consisted of cerebellum, entorhinal cortex, hippocampus, hypothalamus, medulla, somatomotor cortex, somatosensory cortex, superior colliculus, thalamus, and visual cortex. Of these, the hippocampus region was made by combining the CA1, CA2, CA3, and dentate gyrus (DG) regions and the isocortex region was used for the visual cortex. The whole-brain tract rendering was set to local index and either FA, MD, AD, or RD (Table S2) on a heat scale of 0–1 in DSI studio. The fiber track colors indicate their direction with red being left to right fibers, blue being front to back fibers, and green being top to bottom fibers.

### 2.6. Adjacency Matrix and Connectogram

The hemisphere of the 72-region atlas-based parcellation used as the ROIs to create the adjacency matrices (Figure 1F) and connectograms (Figure 1H) depended on ipsilateral or contralateral analysis. For close-range fiber analysis, the hemisphere ipsilateral to where the seeds were placed of the 72-region atlas-based parcellation was used as the ROIs, and an adjacency matrix was calculated by using the count of the connecting tracks in DSI studios after tractography. For long-range fiber analysis, the hemisphere contralateral to where the seeds were placed was used for calculation of the count of connecting tracts. The graph theory extraction threshold for both ipsilateral and contralateral was 0.001. In contralateral analysis, heat maps will display all 72 × 72 regions. Chord diagrams of adjacency matrices were generated using the circlize package in R, version 0.4.17, where row and column size were matched, creating ribbons that are to scale with the number of connections between two regions of interest, each color representing a different region. Graphs are undirected. Chord diagrams of contralateral data display cross hemisphere connections, either 36 × 36 left seeds to map tracks to right regions and vice versa, to avoid redundancy.

### 2.7. Network Topography

Brain network topology was characterized for WT and *Cdkl5* mice following the previously described protocol [65]. Using the same tractography that made the adjacency matrices, graph theoretical analysis was calculated by DSI studio for various network parameters. The graph theory [70,71] extraction threshold was 0.001. These network parameters included measures of efficiency, global efficiency and small worldness, and measures of segregation, local efficiency, and clustering coefficient.

The process from imaging to network topology and connectogram are depicted in Figure 1. Characterizing neuronal networks into network measures through graph theory describes the physiological aspect of information processing, quantifying structure and function [72]. Network measures in the DSI studio follow the implementation of the brain connectivity toolbox. Graph theoretical analysis treats brain connections like a graph, so its topology can be quantitatively described by network parameters. We graph weighted measures, such that the connectivity matrix will be normalized so the maximum value of the matrix is one.

### 2.8. Statistical Analysis

We performed multiple unpaired *t*-tests with corrections to assess the volume, FA, AD, MD, and RD of regional ROIs. For differences in fiber tracking, we used multiple unpaired *t*-tests at 95% confidence level. A *p*-value map was then generated showing the regional differences where the *p* < 0.05. A *t*-test was used for network parameters as well.

Differences between WT and KO brain connectivities were performed by comparing the distribution of fiber tracts in between all 36(36 − 1)/2 = 630 pairs of brain regions. We excluded pairs where >50% of their observations were 0 and, to alleviate skewing and make the data more amenable to linear models, log-transformed the data after adding a pseudo count of 1 to avoid taking the log of zero. T-tests with n_WT_ + n_KO_ − 2 = 17 degrees of freedom were used to compare WT and KO pairs and resulting *p* values (Figure 1H) were adjusted using the Benjamini Hochberg procedure to adjust for multiple testing by controlling the FDR at 5% [73]. These computations were performed in Rv4.1.2.

## 3. Results

### 3.1. Probing Brain Micro-Environment and Connectivity with Diffusion MRI and Diffusion Tractography

Diffusion MRI (Figure 1A) generating high-definition fiber tractography (HDFT, Figure 1B) is a non-invasive way to characterize brain micro-environment and to map neuronal connections in the entire brain. Diffusion MRI [44,45,46,47,48,49,50,51,52] leverages anisotropic water diffusion properties in the biological tissue to generate MRI contrast [53,54] sensitive to the local structural or pathophysiological micro-environment. Neuronal fibers (axons, long dendrites) found in white matter tracks, fasciculi, and commissures are the long, slender projections of neurons that transmit electrical and chemical signals inter-connecting brain regions to form “information highway” networks that process information and conduct cognitive functions. There are also afferent (sensory) fibers and efferent (motor) fibers to carry information to and from the central nervous system (CNS) respectively, as well as autonomic fibers to relay information to regulate involuntary sympathetic and parasympathetic functions. In neuronal fibers, water molecules can readily diffuse along neuronal fiber orientation, but are very limited perpendicular to the neuronal fiber orientation due to diffusion barriers of cell membranes and myeline sheath [55,56,57,58]. Diffusion MRI leverages this anisotropic restricted water diffusion property in nerves to non-invasively map neuronal track orientations and connections in the brain. Diffusion tractography models water diffusion patterns in neuronal fibers as a Gaussian distribution and determination of axonal direction by the three principal directions of the tensor [44,45].

Although diffusion tensor imaging (DTI) is by far the most widely used diffusion MRI method, its major limitations have been well described [61,74], particularly its inability to represent the crossing of multiple fibers [75]. The diffusion tensor model assumes one dominant fiber orientation per voxel. This assumption fails when multiple fiber populations with different orientations are present, leading to misinterpretation of DTI metrics [61]. Mouse brains (~1 to 1.5 cm in length, ~0.4–0.5 cm^3^ in volume) are ~3000 times smaller than human brains (~14 to 16 cm in length, ~1200–1400 cm^3^ in volume) [76,77,78,79]. Even with much smaller voxel sizes, conventional DTI [80,81,82] often fall short and cannot distinguish multiple fiber orientations within a voxel in small mouse brains. Several alternative advanced methods have been proposed to address this limitation [83]. Here we use high-angular generalized q-sampling imaging (GQI) [84] with deterministic fiber tracking for this study [50,85,86], optimized for small mouse brains.

GQI [84] is a model-free reconstruction method that quantifies the density of diffusing water at different orientations. This measurement, termed spin distribution function (SDF), is an orientation distribution function of diffusing spins. Studies have shown its greater sensitivity and specificity to white matter characteristics and pathology [87,88,89]. GQI can calculate SDF from a variety of diffusion datasets [90,91], including the DSI dataset, high-angular resolution diffusion imaging (HARDI) [92,93,94], multiple-shell [95,96,97], or combined DTI datasets. GQI provides an analytical relation to compute SDF, and the reconstruction requires only a simple matrix multiplication.

The deterministic fiber tracking algorithm makes use of the local fiber orientations to delineate the whole trajectory. It starts from a seeding point and propagates along fiber orientation until the termination criteria are met. Examples of this approach include the fiber assignment continuous tracking (FACT) method [50] and streamline fiber tracking algorithm [46], both of which assume a single fiber orientation per voxel. With high-angular resolution data, multiple fiber orientations can be resolved per voxel to catch the complex connections of the fiber geometry [85,98,99], more suitable for small mouse brains.

By quantifying the tensors for these principal directions, the trajectories of the axonal connections can be tracked by diffusion fiber tractography, which reveals the axonal orientations and connections between brain areas [59,60]. Eigenvalues of each tensor direction (λ1, λ2, λ3) can quantify diffusivity (Table S2) for each brain region, sensitive to the local micro-structure and micro-environment, thus they can reflect neurodevelopmental and pathophysiological features [100,101,102]. Fractional anisotropy (FA) [44] quantifies the degrees of anisotropy (non-uniformity) of water diffusion in neuronal fibers. FA represents the degree to which diffusion within a voxel-of-interest in isotropic or anisotropic, with a value of 0 representing the former and 1 representing the latter FA is a fraction derived from the ratio between λ1, λ2, and λ3. It has a value ranging from 0 (isotropic) to 1 (totally anisotropic). FA reflects neuronal fiber integrity and coherency. Axial diffusivity (AD) [101] measures diffusion along the axonal orientation, which can reflect axonal integrity and or axonal injury. AD, denoted by λ parallel, quantifies how fast water diffuses along the axonal fibers [103,104,105]. It is estimated by λ1, the first eigenvalue of the tensor. Radial diffusivity (RD) [101] measures diffusion perpendicular to the axonal orientation, reflecting myeline integrity. RD, denoted by λ perpendicular, quantified how fast water diffuses across the axonal bundles. It is estimated by (λ2 + λ3)/2, the average of the second and third eigenvalues of the tensor. It can be used to study demyelination [106]. Mean diffusivity (MD), or apparent diffusion coefficient (ADC), measures overall diffusivity for all directions [101]. MD is the diffusivity average from the three eigenvalues of the tensor. It is often regarded as an approximation of the overall ADC.

Quantitative diffusivity (FA, AD, RD, MD) is sensitive to the micro-environment and micro-structure, thus providing a non-invasive way to probe the pathophysiological and neurodevelopmental features in live brains. Numerous studies have investigated the relation between diffusivity and pathological conditions. For example, axonal injury, traumatic injury, stroke, amyotrophic lateral sclerosis (ALS), and advanced multiple sclerosis (MS) can increase AD, demyelination can increase RD, a tumor can decrease MD, and immune cell infiltration has decreased MD. Vasogenic edema increases MD/ADC whereas cytotoxic edema decreases MD/ADC. Age-dependent changes in AD, RD, and MD during neurodevelopment are well recognized [107,108,109]. Developing brains rapidly change axonal characteristics in axonal density, axonal caliber, and myelination [107,108,109]. Diffusive parameters can be used to characterize brain maturation in pre-term infants [110,111,112,113] which showed gestation-age dependent and brain-region specific changes in AD, RD, and MD. Children with dyslexia [114] showed changes in these diffusivity parameters. Thus, quantitative diffusivity is increasingly used as biomarkers for white matter for neurodevelopment [107,108,109], brain maturation [110,111,112,113], cognitive abilities [114,115], brain injury [116,117,118,119,120], and neurodegeneration [121,122,123,124].

Diffusion MRI was successful in detecting selective vulnerability of the frontal lobes in Rett Syndrome [125]. It is conceivable that quantitative diffusivity might be able to quantify brain changes in CDD to provide a non-invasive quantitative brain-based biomarker for CDD trials.

**Figure 2:**
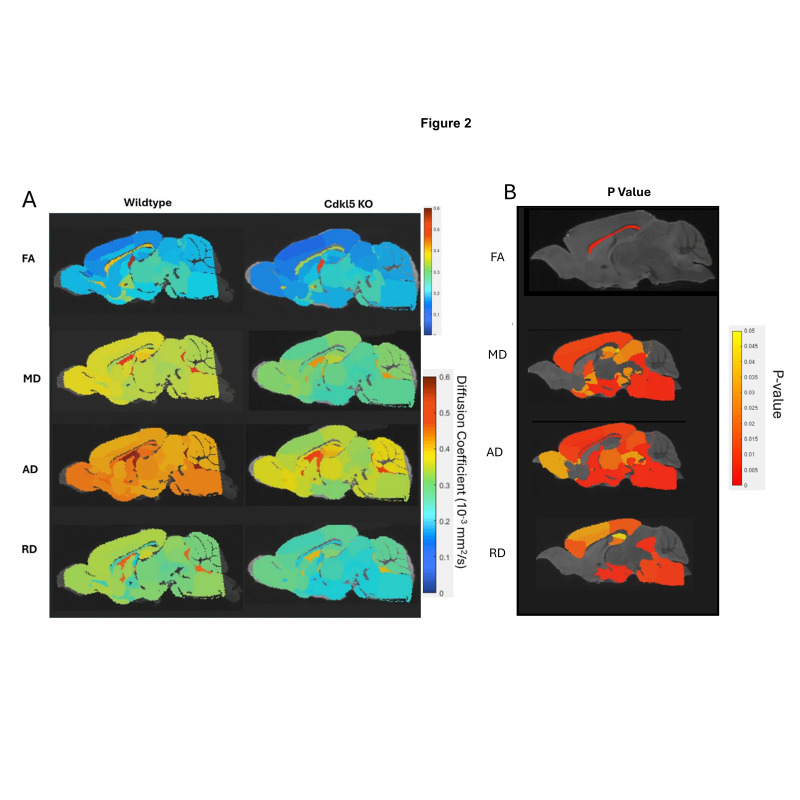
Group comparison of quantitative diffusivity shows a significant difference in FA, MD, AD, and RD. (**A**) Representative images of whole brain have been rendered on a scale of 0–0.6 for average fractional anisotropy (FA, the degree to which diffusion within a voxel-of-interest is isotropic or anisotropic), medial diffusivity (MD, measures diffusivity in all directions), axial diffusivity (AD, measures diffusion parallel to the axonal orientation), and radial diffusivity (RD, measures diffusion perpendicular to the axonal orientation) per ROI. General patterning of FA appears to be the same between WT (n = 8) and Cdkl5 (n = 12), but different patterning for MD, AD, and RD. (**B**) Representative images highlighting regions that are significantly different according to the *t*-test.

We first investigated quantitative diffusivity in hemizygous male *Cdkl5* KO mouse brains compared to those of the age- and sex-matched WT counterparts (Figure 2). Group-averaged quantitative diffusivity mapping covers 3D volumes of the entire brain. They can be viewed in any orientation for each brain region, allowing visual comparison between WT and *Cdkl5* KO mice. Figure 2 shows a mid-sagittal view of the 3D isotropic mapping of group-averaged FA, MD, AD, and RD maps for WT and *Cdkl5* KO mice (Figure 2A) as well as the *p*-value maps (Figure 2B and Table S3) for group comparisons between the hemizygous male *Cdkl5* KO mice and the WT counterparts. A two-sample unpaired *t*-test was used to determine the brain regions with significant differences between the two groups (Figure 2B, and Table S3).

*Cdkl5* KO mice showed significant changes in FA in the corpus collosum (Figure 2B), the colossal white matter gateway for communicating between the left and right hemispheres. This is consistent with the observation that CDKL5 sculpts functional callosal connectivity to promote cognitive flexibility [126].

MD, AD, and RD (Figure 2B) were altered in the somatosensory and somatomotor cortex, hippocampus, amygdala, hypothalamus, superior colliculus, pons, and other subcortical regions (Table S3). Somatosensory and somatomotor cortex are crucial for cognitive functioning and decision making, hippocampus [127,128,129] for contextual learning and memory, amygdala [130,131,132,133,134] for anxiety- and fear-like responses, hypothalamus for homeostasis, pons for connecting to spinal cord, and superior colliculus [135,136,137] for visual/motor functions involved in processing optical stimuli, orienting attention, and coordinating hand–eye movements. We found that the brain regions showed significant different diffusivity in *Cdkl5* KO mice are consistent with clinical symptoms in CDD patients. This suggests that brain-region specific quantitative diffusivity mapping can potentially be brain-based biomarkers for CDD.

Our data showed that quantitative diffusivity with diffusion MRI can sensitively detect micro-environmental changes in *Cdkl5* KO mice. The brain areas with significant changes correlated with the brain regions underlying cognitive disabilities found in CDD patients.

### 3.2. Global High-Definition Fiber Tractography (HDFT)

Next, we compared streamlines of the 72 regions on a whole-brain level. The high-definition fiber tractography (HDFT Figure 1B) and high-resolution anatomical T_2_-weighted MRI (Figure 1C) were first co-registered to the Allen Brain Atlas [62,63,64] space (Figure 1D) for atlas-based segmentation (Figure 1E) to parcellate each mouse brain into 72 brain regions.

The diffusion parameters (Figure 2, Table S3) showed significant differences in many regions between *Cdkl5* and WT mice. We investigated if the neuronal fiber numbers were also altered. To do this, the numbers of diffusion streamlines passing through each brain region for each mouse were generated, then the group mean streamline numbers are compared between *Cdkl5* and WT mice using a two-sample unpaired *t*-test adjusting for FDR at 5%. The average numbers of diffusion streamlines, reflecting the total neuronal fibers, were significantly altered in several regions of *Cdkl5* mice (Table S4, highlighted brain regions), including corpus callosum (LCC), cerebellum (LCB), motor related superior colliculus (LSUC), hypothalamus (LHY), inferior colliculus (LIC), amygdala (LCOA), somatomotor cortex (LMO), somatosensory cortex (LSS), piriform cortex (LPIR), taenia tecta (LTT), retrohippocampal region (LRHP), and mid-brain (LMB). Each of the altered brain regions are responsible for different cognitive functions— the cortex [138,139] involved in executive functions, hippocampus [140,141,142,143,144,145] in contextual learning, amygdala [130,131,132,133,134] in anxiety and fear conditioning, cerebellum [146,147] in motor functions, piriform cortex [148,149] for olfactory processing, hypothalamus [150,151] for homeostasis, autonomic regulation, emotional expression, and stress responses, whereas the motor-related superior colliculus [152,153,154] transforms sensory and cognitive signals into motor commands for saccadic eye movements and coordination of eye–head–body movements. These altered brain regions carrying out cognitive functions correlated with clinical presentations of CDD patients [1,2,3,4,5,6,7,8,9,10,11,12], such as intellectual disabilities; anxiety and irritability; circadian rhythm dysregulation and sleep disturbances; autonomic dysfunctions; visual and cortical visual impairments with poor visual tracking and reduced eye contact; hypotonia and motor abnormalities with spasticity, dystonia, or poor motor coordination.

Our data demonstrated that *Cdkl5* mutant mice exhibited region-specific alterations in diffusion-derived streamline density, a proxy for neuronal fiber numbers, within brain regions implicated in the cognitive impairments characteristic to CDD clinical manifestation.

### 3.3. Brain Topology with High-Definition Brain Network (HDBN) Analysis

We further characterize the brain topology in *Cdkl5* brains using graph theory. Brain topology [72,155,156,157] refers to the organizational principles of the brain’s structural or functional networks, describing how neural elements (e.g., neurons, neuronal populations, or brain regions) are interconnected, formalized using graph theory [155,158,159], in which brain regions are modeled as nodes and their anatomical or functional connections as edges. This framework enables quantitative characterization of global and local network properties that govern information integration, segregation, and robustness of brain systems. Graph theory provides a powerful mathematical framework for delineating brain network architecture by representing the brain as a complex system of interconnected elements and enabling quantitative characterization of its organization across multiple spatial scales. In this framework, nodes typically correspond to anatomically or functionally defined brain regions, while edges represent structural or functional connections between nodes, derived from diffusion tractography. Topological features of brain networks include measures such as density, clustering coefficient, path length, global or local efficiency, and small worldness, which collectively capture how efficiently information is transferred and processed across the brain. Alterations in brain topology have been widely reported in neurodevelopmental, neuropsychiatric, and neurodegenerative disorders, suggesting that disrupted network organization is a core mechanism underlying cognitive and behavioral impairments.

Here we investigate if disruption of the brain network architecture underlies neurocognitive impairments in CDD. Adjacency matrices (Figure 1F) represent interconnections among 72 brain regions. Adjacency matrices (Figure 1F) capturing interregional connectivity among 72 brain regions were aggregated across individual mice within the WT or *Cdkl5* cohorts to generate a group-level connectogram (Figure 1H), which is a circular graphical representation that visualizes the structural or functional connectivity between brain regions, depicting nodes as regions and links as their pairwise connections. The brain topology can also be represented with the ball-and-stick model (Figure 1G), which is a simplified network representation in which brain regions are modeled as nodes (“balls”) and their structural or functional connections are modeled as edges (“sticks”), allowing the topological organization of the brain (e.g., hubs, modules, and small-world properties) to be analyzed using graph theory.

To compare connectivity between WT and *Cdkl5* KO brains, we performed two-sample unpaired *t*-test for pair-wise comparisons of the distribution of fiber tracts in between all 36(36 − 1)/2 = 630 pairs of brain regions, aggregating among all individual brains to generate the adjusted *p*-value matrix (Figure 1I), using the two-step adjusted *p* values with signs. The lighter colors represent |*p*| values are in the range of 0.01–0.05, whereas the darker colors represent |*p*| values in the range of 0–0.01. Although *p* values do not have signs, we artificially assign “positive” or “negative” denotations to indicate the direction of deviations of *Cdkl5* brains from the WT brains. The blue colors denote artificially assigned “negative” *p* values, indicating *Cdkl5* KO brains have less connections than WT brains, whereas the orange colors denote artificially assigned “positive” *p* values, indicating *Cdkl5* KO brains have more connections than the WT counterparts. The adjusted *p*-value matrix can compare shorter pair-wide connections within the hemispheres (ipsilateral) or longer-range pair-wide connections to the other hemisphere (contralateral). The topological analysis can be performed at the whole-brain levels or for each sub-circuitry.

In brain network analysis using graph theory [160,161,162,163,164], brain network topological characteristics can be quantified by network parameters. Healthy brains typically exhibit a “small-world” [165,166,167] topology. The “small worldness” in brain networks refers to a topological property where the brain’s connectivity graph shows both high local clustering (segregation) and short average path lengths (global integration), supporting efficient information processing compared to random networks. In neuroscience, it is quantified by comparing the clustering coefficient and path length of a real brain network to those of equivalent random graphs, with a small-world index (σ) > 1 indicating small-world characteristics in structural and functional connectomes. The “path length” refers to the average shortest number of edges that must be traversed to connect any two nodes, reflecting the efficiency of information transfer across the network. Shorter path lengths indicate faster global communication between brain regions, while longer path lengths suggest less efficient connectivity. The “clustering coefficient” is a measure in network theory that quantifies how “clustered” or “connected” a node’s neighbors are. In other words, it tells you the likelihood that two nodes that are both connected to a common node are also connected to each other. It is commonly used in social networks, brain networks, and other complex networks to describe local connectivity. The “global efficiency” measures the ability of the network to integrate information across the whole brain. It quantifies how efficiently signals can travel between any pair of nodes in the network. The “local efficiency” measures how efficiently information is exchanged within the immediate neighborhood of a node. It reflects the fault tolerance of the network—how well communication is preserved if a node is removed. The “density” is a measure of how many connections (edges) exist in a network relative to the maximum possible number of connections. For a brain network, nodes typically represent brain regions, and edges represent structural (e.g., white matter tracts) or functional (e.g., correlated activity) connections. High density can mean that brain regions are highly interconnected; the network is more integrated, whereas low density usually means fewer connections relative to possible ones, indicating that the brain network is sparser. “Transitivity” is a measure of the tendency for nodes (brain regions) to form triangles, reflecting the likelihood that two neighbors of a node are also connected. It is a global measure of clustering in the network. Essentially, high transitivity indicates tightly interconnected modules or communities in the brain. The “assortativity coefficient” measures the tendency of nodes (brain regions) to connect to other nodes that are similar in some property, most commonly degree (number of connections). If the assortativity coefficient is positive, high-degree nodes tend to connect to other high-degree nodes (and low-degree to low-degree), showing a “like-with-like” connectivity pattern. If it is negative, high-degree nodes tend to connect to low-degree nodes, indicating disassortative mixing. If it is near zero, connections are roughly random with respect to node degree. In brain networks, assortativity can indicate network resilience and organization. Positive assortativity suggests robustness against random damage because highly connected hubs are interconnected. Negative assortativity often occurs in biological networks where hubs connect to peripheral nodes, which can optimize communication efficiency. In graph theory applied to brain networks, a “rich club” refers to a set of highly connected hub nodes that are more densely interconnected with each other than expected by chance. These nodes typically have a high degree of connections and form a “club” of influential regions that facilitate efficient communication and integration across the brain. The “rich club” organization of the brain supports global integration, efficient information transfer, and resilience to localized damage.

### 3.4. Data-Driven Network Topology Characterization of Cdkl5 KO Brains

We first characterize brain network topology in *Cdkl5* KO brains by a data-driven approach. Our data found significant changes in diffusion parameters (Figure 2, Table S3) and diffusion streamlines (Table S4) which reflect the numbers of neuronal fibers in the cerebellum, superior colliculus, hippocampus, thalamus, entorhinal cortex, hypothalamus, medulla, motor cortex, and somatosensory cortex. All these areas carry out cognitive functions that are characteristic of CDD clinical manifestations. We delineate network topology in these brain regions.

#### 3.4.1. Network Topology Changes in Cdkl5 at the Whole-Brain Level

**Figure 3:**
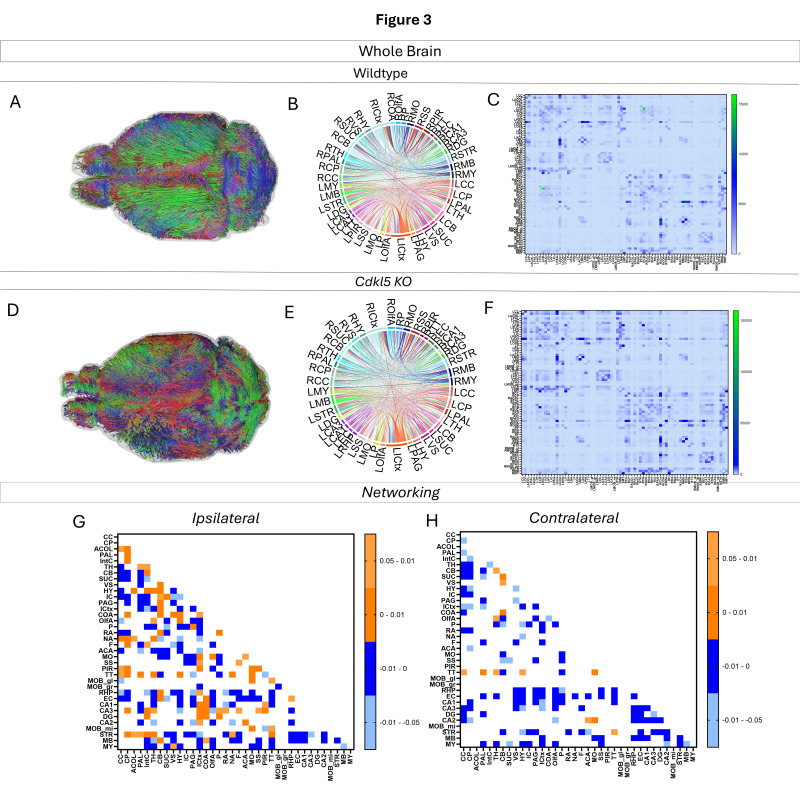
Diffusion tractography and brain network analysis for the whole brain shows a difference in multiple regional connections. (**A**,**D**) Diffusion tractography (**B**,**E**) connectogram (**C**,**F**) adjacency matrix for WT (n = 8) (**A**–**C**) or hemizygous *Cdkl5* KO (n = 12) mice (**D**–**F**). (**G**,**H**) *p*-value matrix to compare connectomes, an adjacency matrix of signed FDR adjusted *p* values (S5) shows connections between regions where *Cdkl5* KO > WT (0.01–0.05 light orange, 0.005–0.01 dark orange) and connections between regions where WT > *Cdkl5* KO (0.0–−0.05 light blue, −0.005–−0.01 dark blue) for (**G**) ipsilateral hemisphere connections (left-to-left and right-to-right hemisphere), and (**H**) contralateral connections (right to left and left to right hemispheres).

Brain topological analysis at the whole-brain level (Figure 3 and Table S5) found extensive changes in many pairs of connections for the *Cdkl5* group. While *Cdkl5* brains showed many brain connections deviated from those in WT counterparts (blue or orange colors in *p*-value matrixes in Figure 3G,H), many brain connections are normal (blank in the *p*-value matrixes in Figure 3G,H). None of the brain network parameters by graph theory at the whole-brain level (small worldness, path length, clustering coefficient, global efficiency, local efficiency, density, transitivity, assortativity coefficient, or rich clubs, etc.) showed significant differences between *Cdkl5* KO and WT brains. This indicates that *Cdkl5* brain network changes are region specific in local circuitries, not at the global whole-brain level.

#### 3.4.2. Regional Network Topology Changes in Cdkl5 Brains

**Figure 4:**
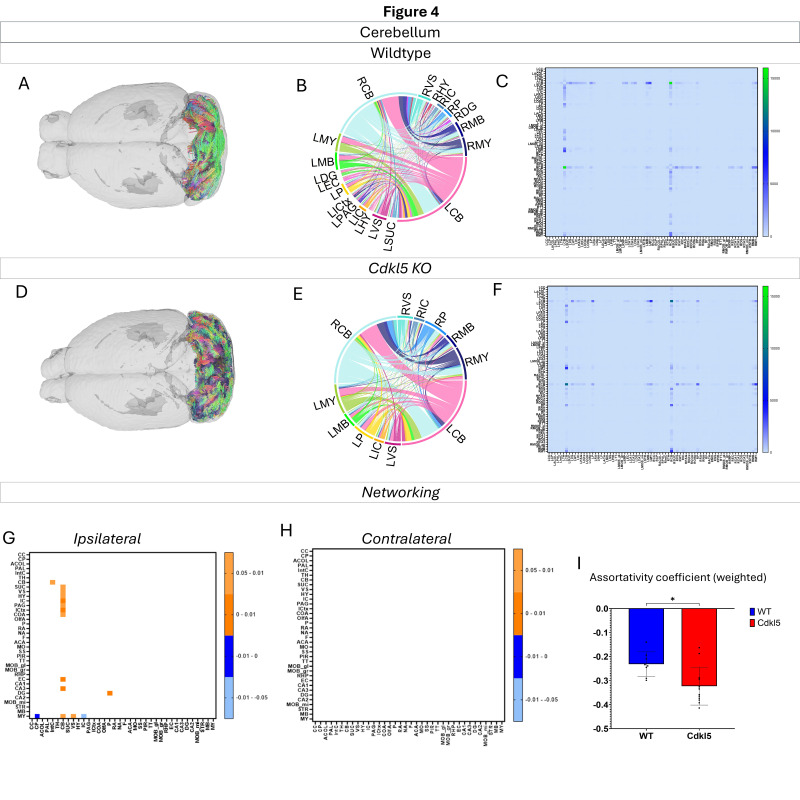
Diffusion tractography and brain network analysis for cerebellum showcases differences in both the *p*-value maps and the assortativity coefficient. (**A**,**D**) Diffusion tractography (**B**,**E**) connectogram (**C**,**F**) adjacency matrix for WT (n = 8) (**A**–**C**) or hemizygous *Cdkl5* KO (n = 12) mice (**D**–**F**). (**G**,**H**) A *p*-value matrix to compare connectomes, an adjacency matrix of signed FDR adjusted *p* values (S6) shows connections between regions where *Cdkl5* KO > WT (0.01–0.05 light orange, 0.005–0.01 dark orange) and connections between regions where WT > *Cdkl5* KO (0.0–−0.05 light blue, −0.005–−0.01 dark blue) for (**G**) ipsilateral hemisphere connections (left to left and right to right hemisphere), (**H**) contralateral connections (right to left and left to right hemispheres), (**I**) network parameter-assortativity coefficient (weighted). Blue—WT, red—*Cdkl5* KO. The “*” above the bar graph indicates a statistical difference.

Networking of the cerebellum (Figure 4, Table S6) shows that there are a variety of changes in the connections through the cerebellum. The ipsilateral (Figure 4G) and contralateral (Figure 4H) showcase regional connections are altered with the majority of them showing a significantly more WT Connections. Furthermore, the network parameters (Figure 4I) show that the assortativity coefficient is also significantly altered.

**Figure 5:**
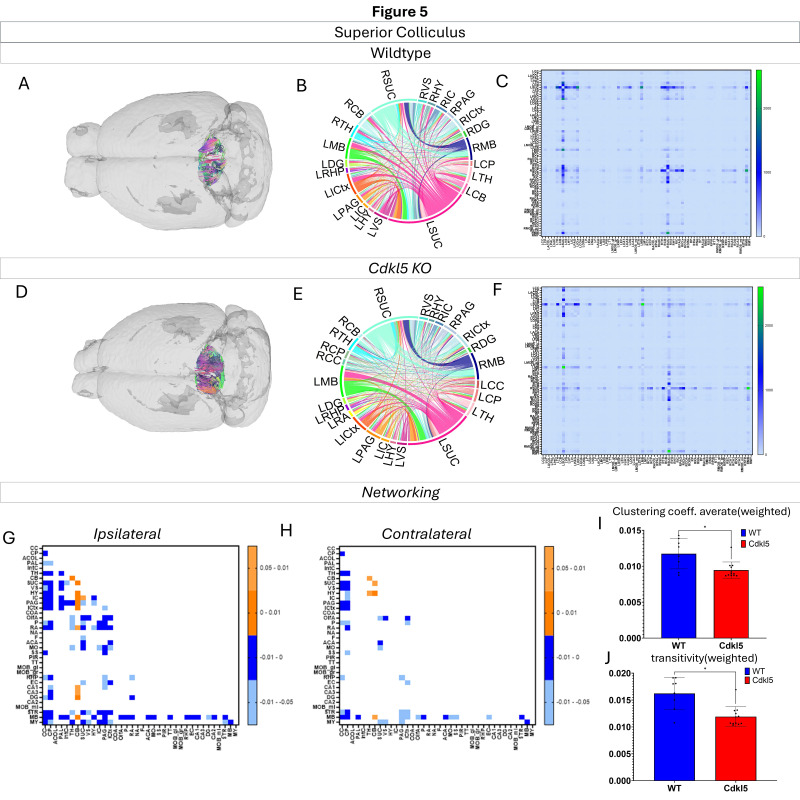
Diffusion tractography and brain network analysis for the superior colliculus shows differences in networking and network parameters. (**A**,**D**) Diffusion tractography (**B**,**E**) connectogram (**C**,**F**) adjacency matrix for WT (n = 8) (**A**–**C**) or hemizygous *Cdkl5* KO (n = 12) mice (**D**–**F**). (**G**,**H**) A *p*-value matrix to compare connectomes, an adjacency matrix of signed FDR adjusted *p* values (S7) shows connections between regions where *Cdkl5* KO > WT (0.01–0.05 light orange, 0.005–0.01 dark orange) and connections between regions where WT > *Cdkl5* KO (0.0–−0.05 light blue, −0.005–−0.01 dark blue) for (**G**) ipsilateral hemisphere connections (left to left and right to right hemisphere), (**H**) contralateral connections (right to left and left to right hemispheres), (**I**,**J**) network parameters, The “*” above the bar graphs indicates a statistical difference. (**I**) clustering coefficient (weighted), (**J**) transitivity (weighted). Blue—WT, red—*Cdkl5* KO.

Networking of the superior colliculus (Figure 5, Table S7) shows that there are a variety of changes in the connections through the superior colliculus. The ipsilateral (Figure 5G) and contralateral (Figure 5H) showcase regional connections are altered with many of them showing a significant increase in the *Cdkl5* KO mice regional connections. Furthermore, the network parameters show that the clustering coefficient average (Figure 5I) and transitivity (Figure 5J) are also significantly altered.

**Figure 6:**
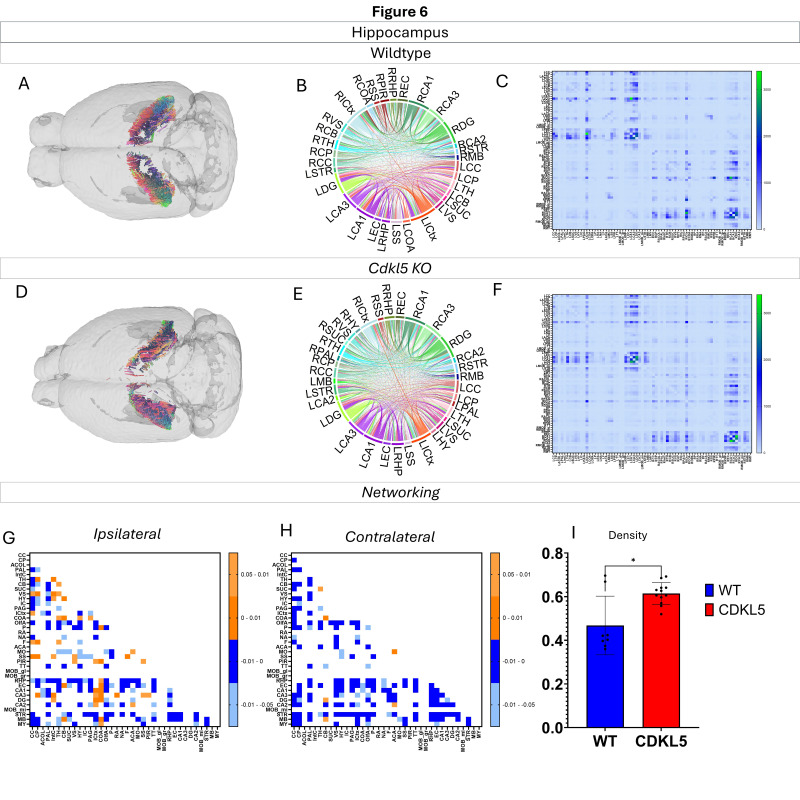
Diffusion tractography and brain network analysis for the hippocampus shows differences in the *p*-value matrix and density. (**A**,**D**) Diffusion tractography (**B**,**E**) connectogram (**C**,**F**) adjacency matrix for WT (n = 8) (**A**–**C**) or hemizygous *Cdkl5* KO (n = 12) mice (**D**–**F**). (**G**,**H**) A *p*-value matrix to compare connectomes, an adjacency matrix of signed FDR adjusted *p* values (S8) shows connections between regions where *Cdkl5* KO > WT (0.01–0.05 light orange, 0.005–0.01 dark orange) and connections between regions where WT > *Cdkl5* KO (0.0–−0.05 light blue, −0.005–−0.01 dark blue) for (**G**) ipsilateral hemisphere connections (left to left and right to right hemisphere), (**H**) right contralateral connections (right to left and left to right hemispheres), (**I**) network parameter–density. Blue—WT, red—*Cdkl5* KO. The “*” above the bar graph indicates a statistical difference.

Networking of the hippocampus (Figure 6, Table S8) shows that there are a variety of changes in the connections through the hippocampus. The ipsilateral (Figure 6G) and contralateral (Figure 6H) showcase regional connections that are altered with most of them showing a significant increase in the *Cdkl5* KO mice regional connections. Additionally, the network parameters (Figure 6I) show that the density is also significantly altered.

**Figure 7:**
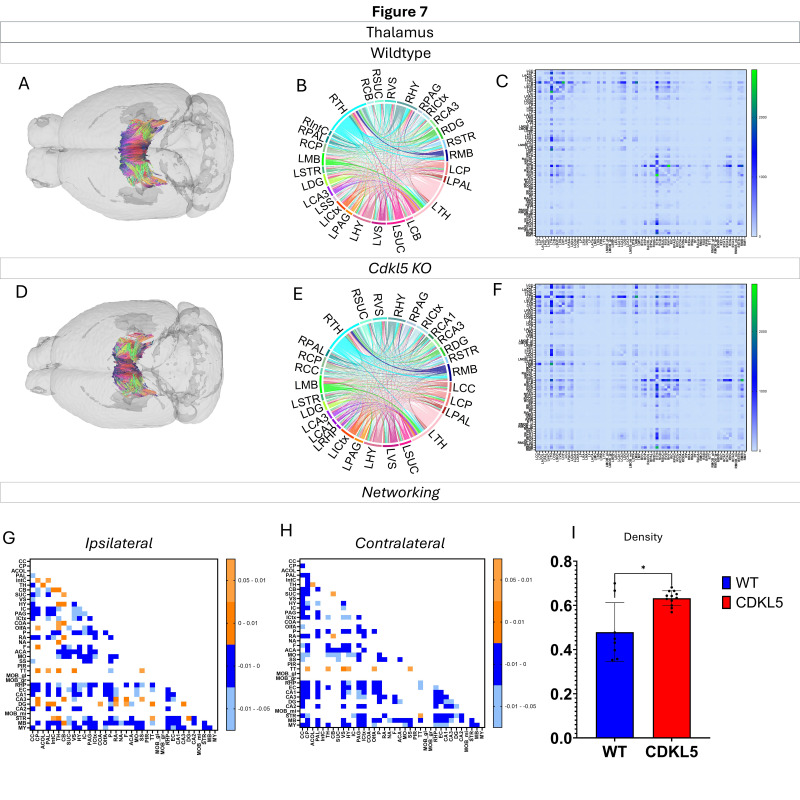
Diffusion tractography and brain network analysis for the thalamus shows a difference in networking and density. (**A**,**D**) Diffusion tractography (**B**,**E**) connectogram (**C**,**F**) adjacency matrix for WT (n = 8) (**A**–**C**) or hemizygous *Cdkl5* KO (n = 12) mice (**D**–**F**). (**G**,**H**) A *p*-value matrix to compare connectomes, an adjacency matrix of signed FDR adjusted *p* values (S9) shows connections between regions where *Cdkl5* KO > WT (0.01–0.05 light orange, 0.005–0.01 dark orange) and connections between regions where WT > *Cdkl5* KO (0.0–−0.05 light blue, −0.005–−0.01 dark blue) for (**G**) ipsilateral hemisphere connections (left to left and right to right hemisphere), (**H**) contralateral connections (right to left and left to right hemispheres), (**I**) network parameter–density. Blue—WT, red—*Cdkl5* KO. The “*” above the bar graph indicates a statistical difference.

Networking of the thalamus (Figure 7, Table S9) shows that there are a variety of changes in the connections through the thalamus. The ipsilateral (Figure 7G) and contralateral (Figure 7H) showcase regional connections that are altered with most of them showing a significant increase in the *Cdkl5* KO mice regional connections. The network parameters (Figure 7I) also show that the density is significantly altered.

**Figure 8:**
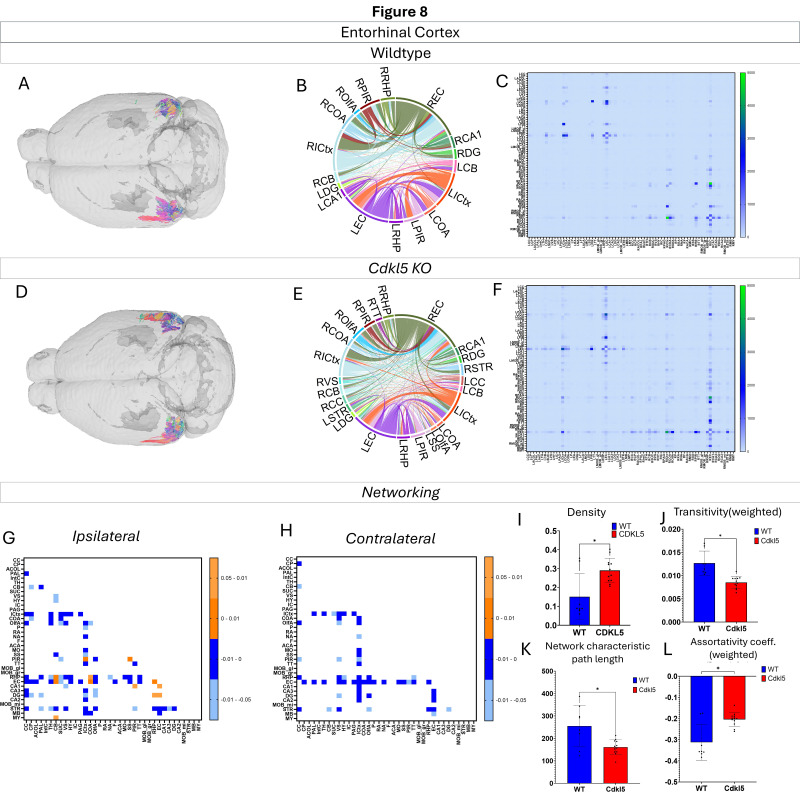
Diffusion tractography and brain network analysis for the entorhinal cortex demonstrates the differences in networking and network parameters. (**A**,**D**) Diffusion tractography (**B**,**E**) connectogram (**C**,**F**) adjacency matrix for WT (n = 8) (**A**–**C**) or hemizygous *Cdkl5* KO n = 12) mice (**D**–**F**). (**G**,**H**) A *p*-value matrix to compare connectomes, an adjacency matrix of signed FDR adjusted *p* values (S10) shows connections between regions where *Cdkl5* KO > WT (0.01–0.05 light orange, 0.005–0.01 dark orange) and connections between regions where WT > *Cdkl5* KO (0.0–−0.05 light blue, −0.005–−0.01 dark blue) for (**G**) ipsilateral hemisphere connections (left to left and right to right hemisphere), (**H**) contralateral connections (right to left and left to right hemispheres), (**I**–**L**) network parameter where significance is indicated by a “*”, (**I**) density, (**J**) transitivity (weighted), (**K**) path length, (**L**) assortativity coefficient (weighted). Blue—WT, red—*Cdkl5* KO.

Networking of the entorhinal cortex (Figure 8, Table S10) shows that there are a variety of changes in the connections through the entorhinal cortex. The ipsilateral (Figure 8G) and contralateral (Figure 8H) showcase regional connections that are altered with most of them showing a significant increase in the *Cdkl5* KO mice regional connections. The network parameters demonstrate that the density (Figure 8I), transitivity (Figure 8J), network characteristic path length (Figure 8K) and assortativity coefficient (Figure 8L) are altered.

**Figure 9:**
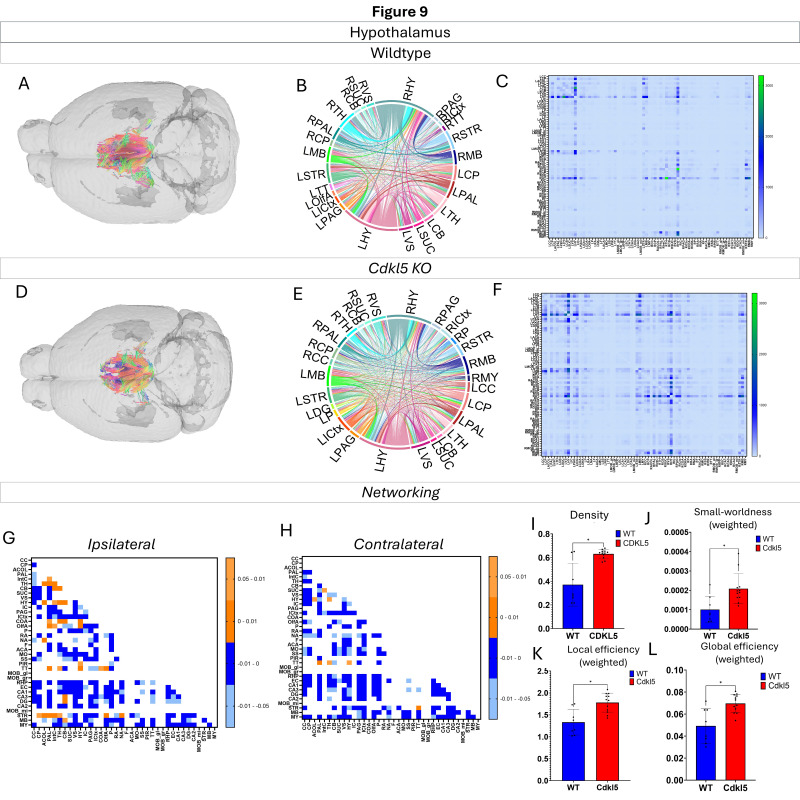
Diffusion tractography and brain network analysis for the hypothalamus describes a multitude of changes regarding networking and the network parameters. (**A**,**D**) Diffusion tractography (**B**,**E**) connectogram (**C**,**F**) adjacency matrix for WT (n = 8) (**A**–**C**) or hemizygous *Cdkl5* KO (n = 12) mice (**D**–**F**). (**G**,**H**) A *p*-value matrix to compare connectomes, an adjacency matrix of signed FDR adjusted *p* values (S11) shows connections between regions where *Cdkl5* KO > WT (0.01–0.05 light orange, 0.005–0.01 dark orange) and connections between regions where WT > *Cdkl5* KO (0.0–−0.05 light blue, −0.005–−0.01 dark blue) for (**G**) ipsilateral hemisphere connections (left to left and right to right hemisphere), (**H**) contralateral connections (right to left and left to right hemispheres), (**I**–**L**) network parameter where the "*" indicates statistical significance, (**I**) density, (**J**) small worldness (weighted), (**K**) local coefficiency (weighted), (**L**) global efficiency (weighted). Blue—WT, red—*Cdkl5* KO.

Networking of the hypothalamus (Figure 9, Table S11) shows that there are a variety of changes in the connections through the hypothalamus. The ipsilateral (Figure 9G) and contralateral (Figure 9H) showcase regional connections that are altered with most of them showing a significant increase in the *Cdkl5* KO mice regional connections. The network parameters (Figure 9K) demonstrate that the density (Figure 9I), small worldness (Figure 9J), local efficiency (Figure 9K), and global efficiency (Figure 9L) are altered.

**Figure 10:**
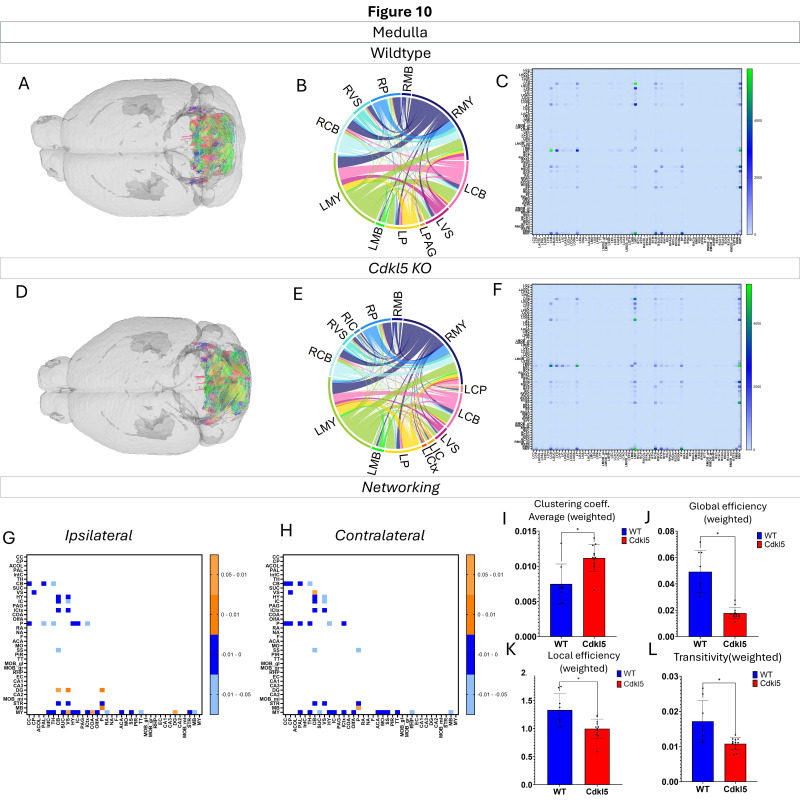
Diffusion tractography and brain network analysis for the medulla demonstrates changes in the *p*-value maps and network parameters. (**A**,**B**) Diffusion tractography (**B**,**E**) connectogram (**C**,**F**) adjacency matrix for WT (n = 8) (**A**–**C**) or hemizygous *Cdkl5* KO (n = 12) mice (**D**–**F**). (**G**–**J**) A *p*-value matrix to compare connectomes, an adjacency matrix of signed FDR adjusted *p* values (S12) shows connections between regions where *Cdkl5* KO > WT (0.01–0.05 light orange, 0.005–0.01 dark orange) and connections between regions where WT > *Cdkl5* KO (0.0–−0.05 light blue, −0.005–−0.01 dark blue) for (**G**) ipsilateral hemisphere connections (left to left and right to right hemisphere), (**H**) contralateral connections (right to left and left to right hemispheres), (**I**–**L**) network parameter, (**I**) clustering coefficient, (**J**) global efficiency, (**K**) local efficiency, (**L**) transitivity. Blue—WT, red—*Cdkl5* KO. The “*” above the bar graphs indicates a statistical difference.

Networking of the medulla (Figure 10, Table S12) shows that there are a variety of changes in the connections through the medulla. The ipsilateral (Figure 10G) and contralateral (Figure 10H) showcase regional connections that are altered with most of them showing a significant increase in the *Cdkl5* KO mice regional connections. The network parameters demonstrate that the clustering coefficient average (Figure 10I), global efficiency (Figure 10J), local efficiency (Figure 10K), and transitivity (Figure 10L) are all changed.

**Figure 11:**
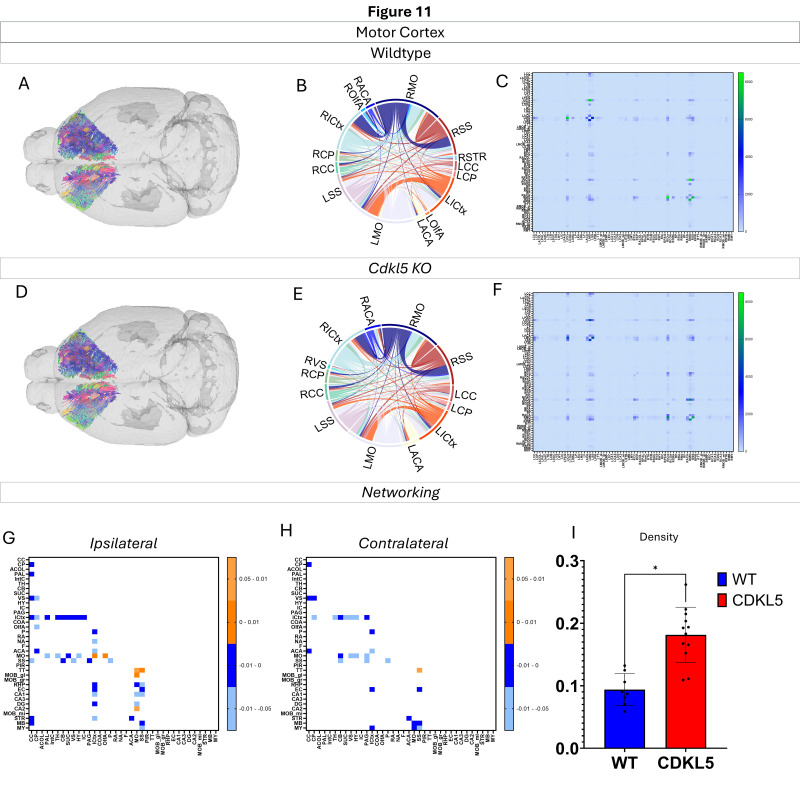
Diffusion tractography and brain network analysis for the motor cortex shows a difference in networking as well as density. (**A**,**D**) Diffusion tractography (**B**,**E**) connectogram (**C**,**F**) adjacency matrix for WT (n = 8) (**A**–**C**) or hemizygous *Cdkl5* KO (n = 12) mice (**D**–**F**). (**G**,**H**) A *p*-value matrix to compare connectomes, an adjacency matrix of signed FDR adjusted *p* values (S13) shows connections between regions where *Cdkl5* KO > WT (0.01–0.05 light orange, 0.005–0.01 dark orange) and connections between regions where WT > *Cdkl5* KO (0.0–−0.05 light blue, −0.005–−0.01 dark blue) for (**G**) ipsilateral hemisphere connections (left to left and right to right hemisphere), (**H**) contralateral connections (right to left and left to right hemispheres), (**I**) network parameter–density. Blue—WT, red—*Cdkl5* KO. The “*” above the bar graph indicates a statistical difference.

Networking of the motor cortex (Figure 11, Table S13) shows that there are a variety of changes in the connections through the motor cortex. The ipsilateral (Figure 11G) and contralateral (Figure 11H) showcase regional connections that are altered with most of them showing a significant increase in the *Cdkl5* KO mice regional connections. The network parameters (Figure 11I) show that the density is changed.

**Figure 12:**
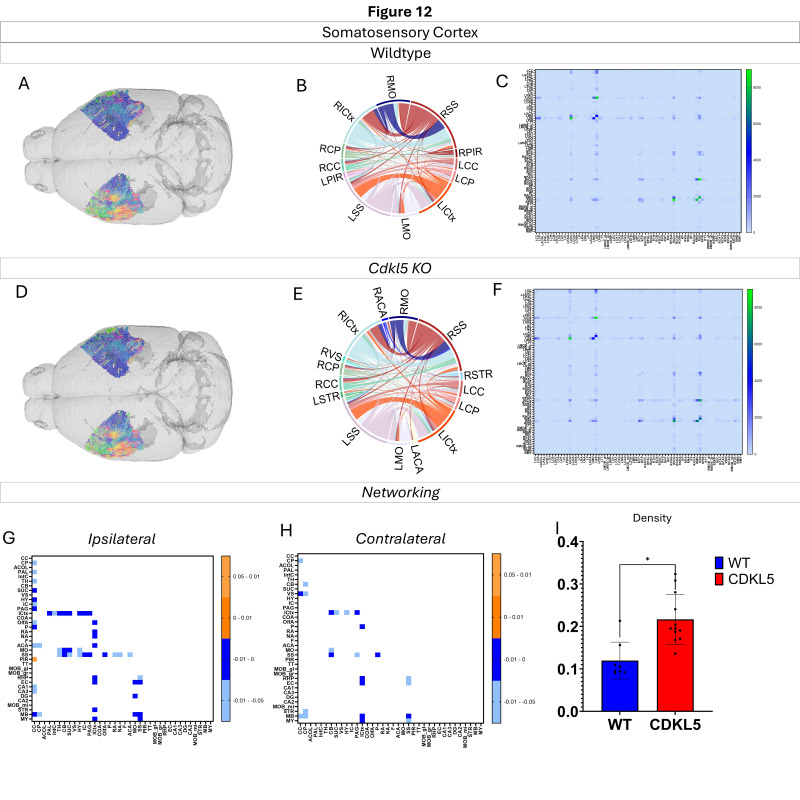
Diffusion tractography and brain network analysis for the somatosensory cortex shows the *Cdkl5* KO mice have significantly less connections in some areas and a higher density. (**A**,**D**) Diffusion tractography (**B**,**E**) connectogram (**C**,**F**) adjacency matrix for WT (n = 8) (**A**–**C**) or hemizygous *Cdkl5* KO (n = 12) mice (**D**–**F**). (**G**,**H**) A *p*-value matrix to compare connectomes, an adjacency matrix of signed FDR adjusted *p* values (S14) shows connections between regions where *Cdkl5* KO > WT (0.01–0.05 light orange, 0.005–0.01 dark orange) and connections between regions where WT > *Cdkl5* KO (0.0–−0.05 light blue, −0.005–−0.01 dark blue) for (**G**) ipsilateral hemisphere connections (left to left and right to right hemisphere), (**H**) contralateral connections (right to left and left to right hemispheres), (**I**) network parameter–density. Blue—WT, red—*Cdkl5* KO.The “*” above the bar graph indicates a statistical difference.

Networking of the somatosensory cortex (Figure 12, Table S14) shows that there are a variety of changes in the connections through the somatosensory cortex. The ipsilateral (Figure 12G) and contralateral (Figure 12H) showcase regional connections that are altered with most of them showing a significant increase in the *Cdkl5* KO mice regional connections. The network parameters (Figure 12I) show that the density is changed.

**Figure 13:**
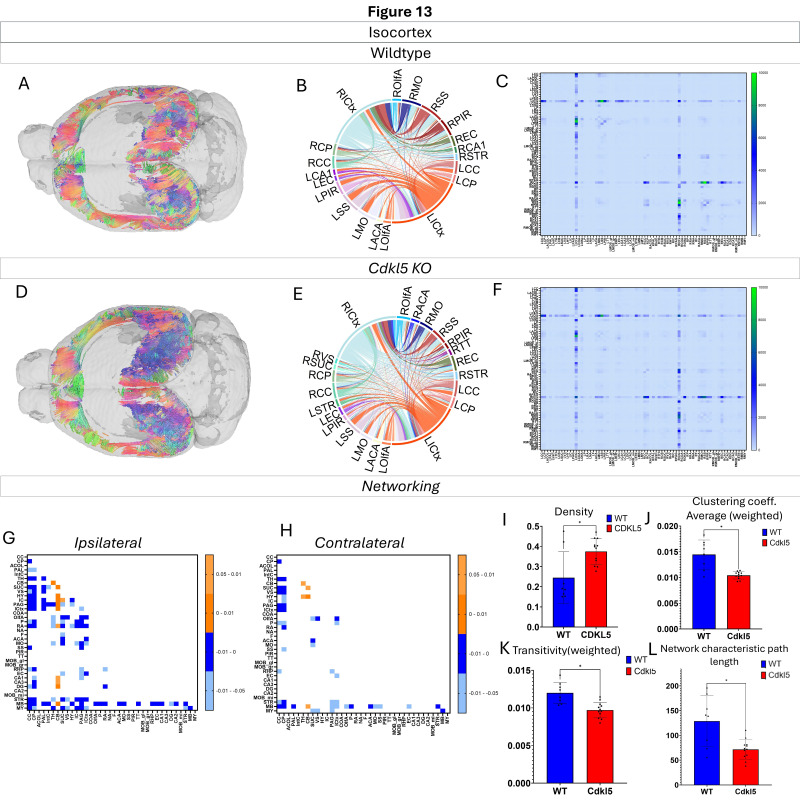
Diffusion tractography and brain network analysis for the isocortex showcases differences in the networking as well as networking parameters. (**A**,**D**) Diffusion tractography (**B**,**E**) connectogram (**C**,**F**) adjacency matrix for WT (n = 8) (**A**–**C**) or hemizygous *Cdkl5* KO (n = 12) mice (**D**–**F**). (**G**,**H**) A *p*-value matrix to compare connectomes, an adjacency matrix of signed FDR adjusted *p* values (S15) shows connections between regions where *Cdkl5* KO > WT (0.01–0.05 light orange, 0.005–0.01 dark orange) and connections between regions where WT > *Cdkl5* KO (0.0–−0.05 light blue, −0.005–−0.01 dark blue) for (**G**) ipsilateral hemisphere connections (left to left right to right hemisphere), (**H**) contralateral connections (right to left and left to right hemispheres), (**I**–**L**) network parameter, (**I**) density, (**J**) clustering coefficient, (**K**) transitivity, (**L**) network characteristic path length (weighted). Blue—WT, red—*Cdkl5* KO. The “*” above the bar graphs indicates a statistical difference.

Networking of the isocortex (Figure 13, Table S15) shows that there are a variety of changes in the connections through the isocortex. The ipsilateral (Figure 13G) and contralateral (Figure 13H) showcase regional connections that are altered with most of them showing a significant increase in the *Cdkl5* KO mice regional connections. The network parameters demonstrate that the density (Figure 13I), clustering coefficient average (Figure 13J), transitivity (Figure 13K), and network characteristic path length (Figure 13L) are all changed.

We then characterized regional brain network topology in brain regions that showed significant changes in diffusion parameters (Figure 2, Table S3) and diffusion streamlines (Table S4) which reflect the numbers of neuronal fibers, including the cerebellum (Figure 4, Table S6), superior colliculus (Figure 5, Table S7), hippocampus (Figure 6, Table S8), thalamus (Figure 7, Table S9), entorhinal cortex (Figure 8, Table S10), hypothalamus (Figure 9, Table S11), medulla (Figure 10, Table S12), motor cortex (Figure 11, Table S13), and somatosensory cortex (Figure 12, Table S14). All these areas carry out cognitive functions that are characteristic of CDD clinical manifestations. All these brain areas showed varied degrees of altered brains short-ranged in the ipsilateral hemispheres (Figure 4, Figure 5, Figure 6, Figure 7, Figure 8, Figure 9, Figure 10, Figure 11 and Figure 12G) as well as in the long-ranged contralateral hemispheres (Figure 4, Figure 5, Figure 6, Figure 7, Figure 8, Figure 9, Figure 10, Figure 11 and Figure 12H). Contrary to the whole-brain network topology, all these brain regions showed changes in brain network parameters. The hypothalamus (Figure 9J) showed changes in small worldness. The entorhinal cortex (Figure 8K) and isocortex (Figure 13L) showed changes in path length. The superior colliculus (Figure 5I), medulla (Figure 10I), and isocortex (Figure 13J) showed changes in clustering coefficient. The hypothalamus (Figure 9L) and medulla (Figure 10J) showed changes in global efficiency. The hypothalamus (Figure 9K) and medulla (Figure 10K) also showed changes in local efficiency. The hippocampus (Figure 6I), thalamus (Figure 7I), entorhinal cortex (Figure 8I), hypothalamus (Figure 9I), motor cortex (Figure 11I), somatosensory cortex (Figure 12I), and isocortex (Figure 13I) showed changes in density. The superior colliculus (Figure 5J), entorhinal cortex (Figure 8J), and medulla (Figure 10L) showed changes in transitivity. The cerebellum (Figure 4I) and entorhinal cortex (Figure 8L) showed changes in assortativity coefficient. The regional brain network topological analysis indicates that Cdkl5 deficiency leads to regional brain network alterations in brain-region specific manners.

### 3.5. Hypothesis-Driven Characterization of Brain Circuitry for CDD

We then characterize brain circuitry indicated by clinical presentations of CDD patients, in particular, circuitry for anxiety and fear-related behavior (3.5.1) and hippocampal–thalamic projection in the temporal lobe epilepsy (3.5.2).

#### 3.5.1. Brain Network Characterization in the Fear Conditioning Circuitry

**Figure 14:**
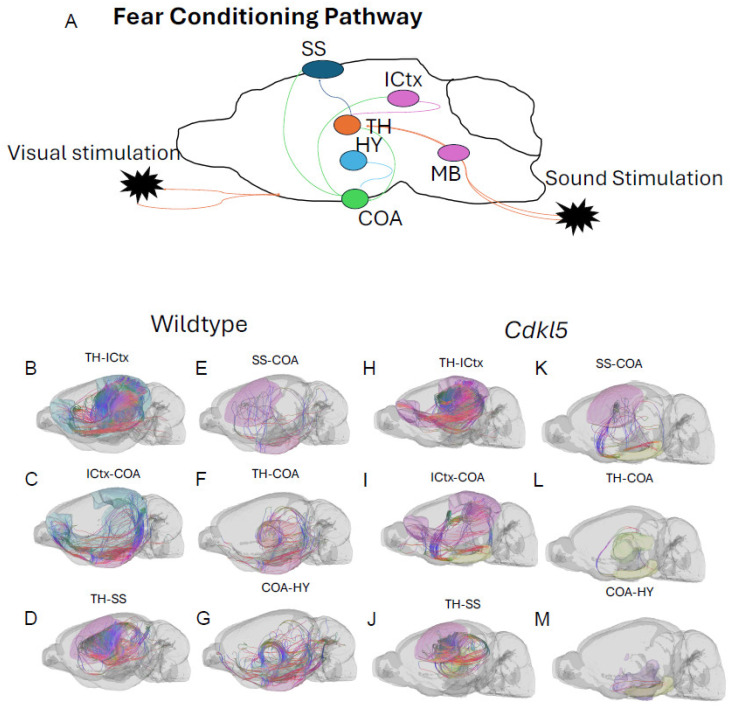
Diffusion tractography and brain network analysis for the fear conditioning pathway. (**A**) Diagram of fear conditioning pathway from visual stimulation through the optic track and through sound stimulation to midbrain, thalamus (TH), hypothalamus (HY), amygdala (CoA), somatosensory cortex (SS), and isocortex (ICtx). (**B**–**M**) Diffusion tractography for (**B**–**G**) WT (n = 8) and (**H**–**M**) Cdkl5 (n = 12), for connections between TH-ICtx (**B**,**H**), ICtx-COA (**C**,**I**), TH-SS (**D**,**J**), SS-COA (**E**,**K**), TH-COA (**F**,**L**), COA-HY (**G**,**M**).

**Figure 15:**
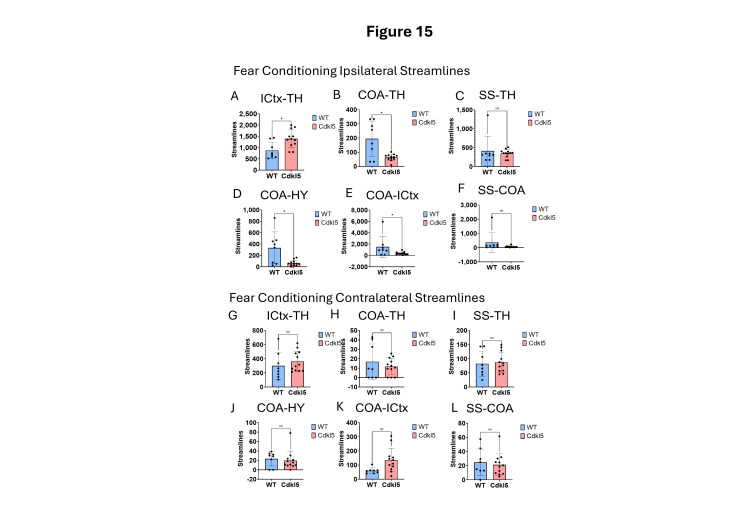
Diffusion tractography and brain network analysis for the fear conditioning shows significant changes in fiber streamlines. (**A**–**F**) Group-averaged connections in ipsilateral (**G**–**L**) or the contralateral connections (S-X) for ICtx-TH (**A**,**G**), COA-TH (B, H), SS-TH (**C**–**I**), COA-HY (**D**,**J**), COA-ICtx (**E**,**K**), SS-COA (**F**,**L**). Blue—WT (n = 8), red—*Cdkl5* KO (n = 12). The “*” above each bar graph indicates there is a significant difference. The “nd” indicates that there is no statistical difference.

CDD patients can show anxiety and fear-related behaviors with increased irritability, anxiety and fear-related behavior, and emotional dysregulation, which suggests potential amygdala dysfunction. *Cdkl5* KO mice [36] displayed similar behavioral phenotypes with significant increases in anxiety- and fear-related responses, as well as hypervigilance. *Cdkl5* KO mice performed poorly in various rodent behavioral testing paradigms, including the fear conditioning [34]. The neuronal circuitry for amygdala-dependent fear conditioning [168] has been uncovered, which involves neuronal pathways (Figure 14A) from the external stimulation to the thalamus (TH, Figure 14A orange), somatosensory cortex (SS, Figure 14A, dark blue), isocortex (ICtx, Figure 14A, purple), hypothalamus (HY, Figure 14A, light blue), and amygdala (CoA, Figure 14A, green). We characterized the neuronal connections between these brain regions in WT (Figure 14B–G, and Figure 15 blue) and *Cdkl5* KO brains (Figure 14H–M, and Figure 15 red). Neuronal connections between the isocortex and thalamus, (Figure 15A, ICtx-TH), the amygdala and thalamus (Figure 15B COA-TH), the amygdala and hypothalamus (Figure 15D, COA-HY), and the amygdala and isocortex (Figure 15E, COA-ICtx) in the ipsilateral hemispheres are significantly altered in *Cdkl5* KO mice compared to the WT counterparts. This indicates that the maladapted neuronal circuitry underlies the behavioral deficits of the fear conditioning paradigm in *Cdkl5* KO mice. On the other hand, long-range connections of these areas to the contralateral hemispheres (Figure 15G–L) are not different. This suggests that the neurocognitive deficits of fear conditioning arise from local neuronal circuitry not from the long-range inter-hemisphere connections.

#### 3.5.2. Brain Network Characterization of Circuitry in the Temporal Lobe Epilepsy

**Figure 16:**
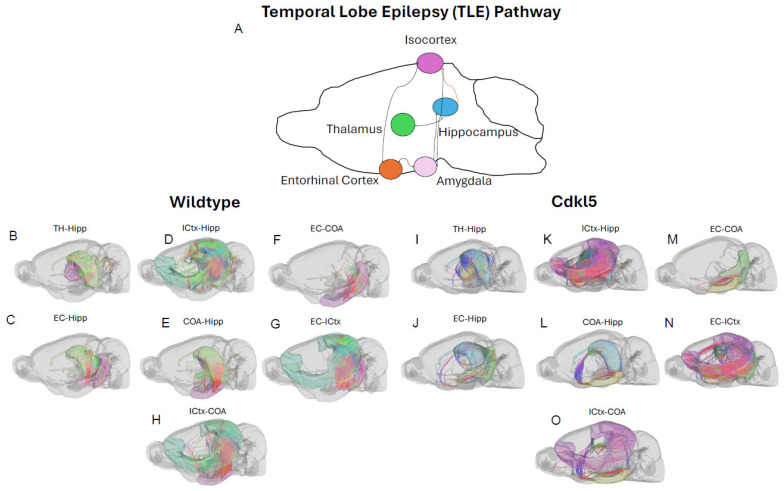
Diffusion tractography and brain network analysis for temporal lobe epilepsy (TLE). (**A**) Diagram of temporal lobe epilepsy pathways from the hippocampus (blue) to thalamus (green), isocortex (dark pink), amygdala (light pink), or entorhinal cortex (orange). (**B**–**O**) Diffusion tractography for (**B**–**H**) WT (n = 8) and (**I**–**O**) Cdkl5 (n = 12), for connections between the thalamus and hippocampus (**B**,**I**), entorhinal cortex–hippocampus (**C**,**J**), isocortex–hippocampus (**D**,**K**), amygdala–hippocampus (**E**,**L**), entorhinal cortex–amygdala (**F**,**M**), entorhinal cortex–isocortex (**G**,**N**), and isocortex–amygdala (**H**,**O**).

The majority of CDD patients suffer from refractory epilepsy with frequent seizures that cannot be controlled by current ASMs. Temporal lobe epilepsy (TLE) [169] is the most common focal epilepsy and often involves complex alterations in neuronal pathways. Understanding the circuitry helps explain seizure generation, propagation, and associated cognitive/emotional symptoms. In addition to key brain regions involved in TLE, such as the hippocampus (Figure 16A blue), entorhinal cortex (Figure 16A orange), amygdala (Figure 16A, light pink), and isocortex (Figure 16A, lavender), the hippocampal (Figure 16A blue)–thalamic (Figure 16A green) pathway [170,171,172] is critically involved in both seizure propagation and modulation. The thalamus is a target for surgery [173] or deep brain stimulation (DBS) [174,175] to treat drug-resistant TLE. Furthermore, the thalamus [176,177] is a crucial brain structure for sensory relay, motor integration, cognitive and emotional processing, and regulation of consciousness, wakefulness, and sleep rhythms. These functions are often impaired in CDD patients.

**Figure 17:**
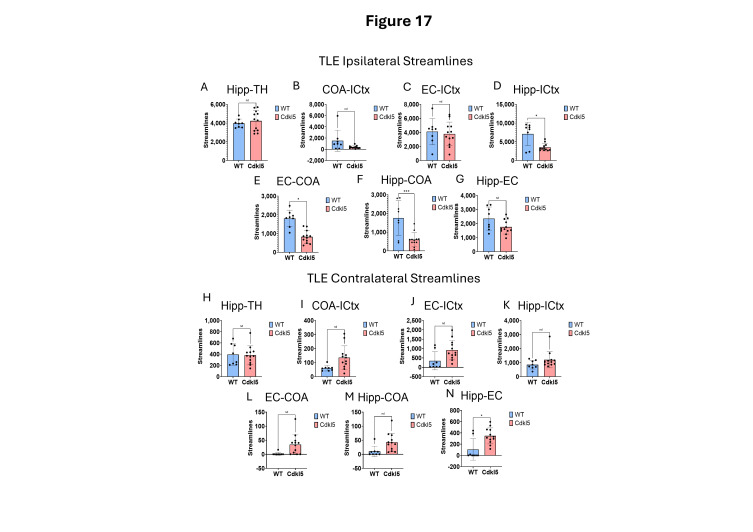
Diffusion tractography and brain network analysis for temporal lobe epilepsy shows significant changes in fiber streamlines. (**A**–**N**) Group-averaged TLE ipsilateral (**A**–**G**) or contralateral connections (**H**–**N**) for Hipp-TH (**A**,**H**), COA-ICtx (**B**,**I**), EC-ICtx (**C**,**J**), –Hipp-ICtx (**D**,**K**), EC-COA (**E**,**L**), Hipp-COA (**F**,**M**), and Hipp-EC (**G**,**N**). Blue—WT (n = 8), Red—*Cdkl5* KO (n = 12). The “*” above each bar graph indicates there is a significant difference. The “nd” indicates that there is no statistical difference.

Here we characterize the TLE and hippocampal–thalamic pathway in *Cdkl5* mice (Figure 16 and Figure 17). The neuronal connections between the hippocampus and isocortex (Figure 17D Hipp-ICtx), entorhinal cortex and amygdala (Figure 17E EC-CoA), and hippocampus and amygdala (Figure 17F, Hipp-CoA) are significantly altered in the ipsilateral hemisphere. However, surprisingly, the hippocampal–thalamic connections (Figure 17A, Hipp-TH) and hippocampal–entorhinal cortex connections (Figure 17G, Hipp-EC) remain largely unchanged. Similar to the fear conditioning circuitry, the long-range TLE connections to the contralateral hemispheres remain the same. This again indicates the re-wiring of the local circuitry.

## 4. Discussion

Although some CDD patients do have abnormal brain structures, most CDD patients have unremarkable anatomical MRI. CDD patients did not show reduced cortical volumes until later in age [178,179]. Similar to most CDD patients, *Cdkl5* KO mice showed largely normal gross brain anatomy with no significant cerebral atrophy or gross structural abnormalities detectable on standard MRI scans, except for subtle regional changes [180,181]. Our study demonstrated that diffusion MRI can sensitively detect neurodevelopmental differences in hemizygous *Cdkl5* KO mice. Although gross anatomy is largely normal, quantitative diffusivity (FA, AD, RD, MD) can sensitively detect regional alteration in *Cdkl5* KO mice. The brain regions that showed altered quantitative diffusivity correlated to the brain regions underlying clinical presentations of cognitive impairments of CDD patients. Our study showed that diffusion MRI is more sensitive than conventional anatomical MRI in detecting neurodevelopmental changes in *Cdkl5* KO mice, consistent with clinical observations in CDD patients [178,179].

Diffusion MRI can be non-invasively performed longitudinally in patients. Regional diffusion parameters (FA, AD, RD, MD) are quantitative and thus can potentially be individual patient-specific brain-based biomarkers for differential diagnosis, to track CDD progression, and to monitor therapeutic efficacy for clinical trials. Although each diffusion parameter (FA, AD, RD, or MD) is non-specific and can be influenced by many pathological conditions related to demyelination, injury, inflammation, infections, etc., combined profiles of all diffusion parameters and brain-region specific patterning may better depict the specific brain changes in individual CDD patients. Furthermore, it can be used to aid precision medicine to monitor CDD progression and CDD repression with therapeutic interventions.

In addition to neurodevelopmental changes, some CDD patients have frequent refractory epilepsy that cannot be controlled by current ASMs. Prolonged seizures with excessive neuron excitation, such as status epilepticus, can lead to excess glutamate release, calcium overload, bioenergetic crisis, and excitotoxicity and metabolic stress resulting in regional brain injury and inflammation. This can acutely change diffusion MRI parameters. To avoid confounding diffusion MRI readouts, diffusion MRI should not be performed acutely after seizure episodes, but should wait until the acute seizure effects resolve for more accurate readouts.

Our data-driven approach showed that *Cdkl5* KO mice exhibit region-specific alterations in diffusion-derived streamline density, a proxy for neuronal fiber organization, within brain regions implicated in the cognitive impairments characteristic of CDKL5 deficiency disorder (CDD) and underlying neurobehavioral phenotypes in *Cdkl5* KO mice [34,126]. Our data-driven approach identified regional alterations that converged with known CDD neuropathology, particularly within the cerebellum and superior colliculus. *Cdkl5* KO mice exhibited significant changes in cerebellar fiber counts, consistent with human imaging studies implicating cerebellar dysfunction in CDD [182]. Notably, disruptions were observed not only in cerebellar afferent and efferent connectivity but also in fibers traversing the cerebellum, suggesting that Cdkl5 loss impairs cerebellar hub-like integration and long-range information flow across distributed brain networks. The superior colliculus [180], a key node in visual processing [183], also showed marked network disruption, aligning with cortical-visual deficits reported in CDD patients [184]. *Cdkl5* KO mice displayed reduced incoming and outgoing connections but increased through passing fibers, indicating a shift from active network participation toward passive conduit-like function. These changes were accompanied by a decreased global clustering coefficient and transitivity, consistent with impaired local network segregation and reduced efficiency of sensorimotor integration. The hippocampus has been consistently implicated as a vulnerable region in individuals with CDD [182]; therefore, we examined hippocampal network organization in greater detail. To capture hippocampal-wide connectivity, we combined ROIs encompassing CA1, CA2, CA3, and the dentate gyrus into a single hippocampal node. Network analysis revealed pronounced alterations in hippocampal fiber connectivity in *Cdkl5* KO mice relative to WT controls, indicating disrupted integration of this region within the broader brain network. In addition to changes in fiber connectivity, we observed a significant increase in hippocampal network density in *Cdkl5* KO mice. This elevated density is consistent with prior reports of hippocampal enlargement in Cdkl5-deficient mice and may reflect aberrant circuit expansion, altered synaptic pruning, or compensatory reorganization of hippocampal circuitry associated with CDKL5 loss. The thalamus serves as a major integrative hub, relaying and modulating information across cortical and subcortical systems that support sensory processing, cognition, and motor control. In *Cdkl5* KO mice, thalamic network architecture was disrupted in multiple dimensions. Specifically, we observed a significant reduction in the number of fibers traversing the thalamus, accompanied by a marked decrease in thalamic network density. These findings suggest impaired thalamic connectivity and reduced network integration, potentially contributing to widespread circuit dysfunction in CDD. While some regions showed alterations in network metrics without changes at the whole-brain level, the circuitry that these regions are a part of end up changed. This suggests that there may be a compensatory process where alternative pathways are recruited to sustain the overall network function.

Our study demonstrated that even though *Cdkl5* KO mouse brains appear to be structurally normal without overt anatomical abnormalities, the brain networks, “the information highways” of the brain, are wired very differently. We have successfully probed these changes in brain network development using high-definition brain network (HDBN) delineation in *Cdkl5* KO mouse brains. Our findings demonstrate that HDBN provides a sensitive and precise method for mapping brain network architecture in *Cdkl5* KO mice, allowing clear differentiation from WT controls. *Cdkl5* KO mice exhibited pronounced alterations in neuronal network organization across multiple key regions, including the somatosensory and somatomotor cortices, hippocampus, hypothalamus, amygdala, and superior colliculus. These regions are critically involved in cognition, learning and memory, regulation of homeostasis, anxiety and fear conditioning, and visual–motor function correlated with neurocognitive phenotypes in *Cdkl5* KO mice [34,36,185,186]. The observed network disruptions in these areas closely parallel the clinical manifestations of CDD patients, who frequently present with cognitive and memory deficits [1,2,3,4], anxiety [187], and cortical visual impairments [184]. Notably, the entorhinal cortex, a central component of the olfactory circuitry, appeared largely unaffected, suggesting region-specific vulnerability in Cdkl5 deficiency. Together, these results highlight both the utility of HDBN for sensitive detection of network-level brain abnormalities and the selective impact of *Cdkl5* loss on functional brain circuits relevant to CDD pathophysiology. This is indicative that CDD, as a developmental and epileptic encephalopathy (DEE), even without epilepsy in mice, the brain network developed abnormally in *Cdkl5* mice. This is similar to clinical observations that some CDD patients do not have frequent seizures, or some seizures can be controlled, but CDD patients still suffer from neurodevelopmental disabilities suggesting underlying brain network abnormalities. Our study suggests that HDBN characterizing brain network topology might be more appropriate and sensitive for depicting CDKL5 deficiency in the brains for both humans and mice. HDBN delineation can also enable utilizing *Cdkl5* KO mice for mechanistic understanding and therapeutic development despite lacking overt seizures.

Both CDD patients and *Cdkl5* KO mice displayed anxiety and fear-related behaviors, suggesting amygdala dysfunctions. Our hypothesis-driven characterization of brain circuitry for fear conditioning found significant alterations of amygdala-related connections, confirming phenotypical observations in *Cdkl5* mouse models and in clinical manifestation of CDD patients.

Our characterization of neural circuitry for TLE further demonstrates the maladaptive neuronal network development underlie cognitive challenges in CDD. TLE is the most frequent form of epilepsy in CDD. TLE [188,189,190] emerges from maladaptive circuitry within the mesial temporal lobe, particularly the hippocampus, entorhinal cortex, and amygdala. Hyperexcitability of pyramidal neurons, combined with loss of inhibitory interneurons, disrupts excitation–inhibition balance, while mossy fiber sprouting of dentate granule cells establishes recurrent excitatory loops that promote seizure initiation. The entorhinal cortex–hippocampus loop sustains epileptiform activity, and amygdala interactions facilitate propagation and emotional manifestations. Hippocampal sclerosis, characterized by selective hippocampal neuronal loss, further remodels circuitry to favor hyperexcitability. Additional temporal neocortical and thalamic networks mediate seizure spread, producing complex partial or secondary generalized seizures. These findings highlight TLE as a network disorder [191,192,193] in which synaptic reorganization, interneuron loss, and aberrant connectivity converge to generate recurrent, self-sustaining epileptic activity. Surprisingly, we found that the hippocampal–thalamic pathway in *Cdkl5* KO mice was normal. Most CDD patients suffer from refractory epilepsy, predominantly TLE. The hippocampal–thalamus project is important in managing drug-resistant TLE. However, *Cdkl5* KO mice do not exhibit TLE. Our hypothesis-driven characterization of brain circuitry for the hippocampal–thalamic project found no changes in *Cdkl5* KO mice. *Cdkl5* KO mice have normal hippocampus–thalamus projection. This is consistent with the observed phenotypes for *Cdkl5* KO mice. Our data demonstrate that HDBN can be sensitive in testing hypothesis-driven brain circuitry delineation in CDD and other DEE.

The Allen Brain Atlas is constructed based on ex vivo histology with very thin slices (100 µm sampling density) and 0.35 µm pixel resolution [194] yielding 461 brain regions [64], whereas MRI voxels are 78 µm and 156 µm in size for anatomical and diffusion MRI, respectively. Many Allen Brain Atlas brain regions are too small and below the detection threshold of the MRI signal-to-noise ratio (SNR). Thus, we combined smaller brain regions in the Allen Brain Atlas to form 72 ROIs used in our analysis. This yielded good SNR and robust and reproducible HDBN results.

Conventional DTI can fall short of resolving neuronal fibers in small mouse brains. Our study used model-free generalized q-sampling imaging (GQI) [84] with high-angular resolutions and a deterministic fiber tracking algorithm more optimal for high-definition fiber tractography in small mouse brains, having overcome the drawbacks of conventional DTI. In conjunction with topological analysis with graph theory, our study has successfully depicted the neuronal network architecture in *Cdkl5* KO mouse brains. Although *Cdkl5* KO mice do not exhibit over seizures as CDD patients, HDBN can sensitively depict neurodevelopmental changes in *Cdkl5* KO mice, providing a sensitive, robust, and quantitative means for using *Cdkl5* KO mice for preclinical therapeutic development.

Studies show that neurocognitive abnormalities due to CDKL5 deficiency can be reversible. Terzic et al. [195] demonstrated that post-developmental loss of CDKL5 in mice leads to abnormal neuronal morphology and behavioral deficits, but restoring CDKL5 expression later in life ameliorated these deficits and reversed many functional abnormalities. CDKL5 is important for maintaining neuronal functions beyond development [195,196]. Using a conditional rescue mouse model, restoration of Cdkl5 expression [195] after the early stages of brain development significantly ameliorated CDD-associated behavioral deficits and normalized aberrant NMDA receptor–mediated signaling. These findings demonstrate that CDKL5 function is required not only during early neurodevelopment but also for the maintenance of mature neural circuit function. Importantly, the ability to reverse core behavioral and synaptic abnormalities following post-developmental gene restoration provides compelling evidence that key aspects of CDD pathophysiology remain plastic beyond early critical periods. Together, these results underscore the potential for disease modification rather than purely symptomatic treatment and support the existence of a broad therapeutic time window for interventions targeting CDKL5-related deficits. Restoration of cognitive and behavioral deficits later in life with CDKL5 suggests normalization of the brain network. HDBN can sensitively delineate and quantify normalization of the brain network with therapeutic interventions.

## 5. Conclusions

Our study is consistent with published data demonstrating that the Cdkl5 KO mice show an altered neurological networking pattern. We demonstrated the profound alterations that the global Cdkl5 KO mouse model has on the neurological patterning. We found that the cerebellum, superior colliculus, hippocampus, as well as the thalamus all demonstrate having their network significantly altered. We also show that the streamlines within the fear conditioning pathway are also significantly affected by the Cdkl5 KO. Further studies to determine the time frame that HDBN changes after therapeutic intervention can facilitate applications to accelerate therapeutic development for CDD beyond neurodevelopment. It would be beneficial to perform our HDBN on genetically altered pigs that show epileptic features in order to translate more relevantly to humans.

## Data Availability

The original contributions presented in this study are included in the article/Supplementary Material. Further inquiries can be directed to the corresponding author.

## Supplementary Materials

The following supporting information can be downloaded at: https://www.mdpi.com/article/10.3390/biom16050652/s1, Table S1: Abbreviations for the 72 brain regions are listed for the Left (L) and Right (R); Table S2: Diffusion parameters; Table S3: Quantitative diffusivity and *p*-values comparing Cdkl5 KO vs WT mice for each mouse brain region; Table S4: Streamlines for each mouse brain region; Table S5: Adjust *p* values for group comparisons of pair-wised connectivity for the whole brain; Table S6: Adjust *p* values for group comparisons of pair-wised connectivity for Cerebellum; Table S7: Adjust *p* values for group comparisons of pair-wised connectivity for Superior Colliculus; Table S8: Adjust *p* values for group comparisons of pair-wised connectivity for Hippocampus; Table S9: Adjust *p* values for group comparisons of pair-wised connectivity for Thalamus; Table S10: Adjust *p* values for group comparisons of pair-wised connectivity for Entorhinal Cortex; Table S11: Adjust *p* values for group comparisons of pair-wised connectivity for Hypothalamus; Table S12: Adjust *p* values for group comparisons of pair-wised connectivity for Medulla; Table S13: Adjust *p* values for group comparisons of pair-wised connectivity for Motor Cortex; Table S14: Adjust *p* values for group comparisons of pair-wised connectivity for Somatosensory Cortex; Table S15: Adjust *p* values for group comparisons of pair-wised connectivity for IsoCortex.

## Abbreviations

CDKL5	Cyclin-dependent kinase-like 5
CDD	CDKL5 Deficient Disorder
DEE	developmental and epileptic encephalopathy
ASM	anti-seizure medication
NDD	neurodevelopmental deficit
KO	Knock out
MRI	Magnetic Resonance Imaging
DTI	Diffusion Tensor Imaging
DWI	Diffusion weighted imaging
HDFT	High-definition Fiber Tractography
HDBN	High-definition Brain Network
ROI	Region of Interest
FOV	Field of View
TE	Echo Time
TR	Repetition Time
FA	Fractional anisotropy
AD	Axial diffusivity
RD	radial diffusivity
MD	mean diffusivity
ADC	apparent diffusion coefficient
RARE	Rapid Imaging with Relaxation Enhancement
FAS	Fast Spin-Echo
GQI	generalized q-sampling imaging
SDF	spin distribution function
TPM	tissue probability map
SPM	statistical parametric maps
GM	grey matter
WM	white matter
OLAW	Office of Laboratory Animal Welfare
IACUC	Institutional Animal Care and Use Committee
FDA	Food and Drug Administration
GEM	genetically engineered mouse
WT	wild type
