# Supplementary material for: High-Definition Brain Network (HDBN) Delineation of CDKL5 Deficiency Disorder (CDD) in Genetically Engineered Mice
Source: Biomolecules. 2026 Apr 28;16(5):652. doi: 10.3390/biom16050652 (PMC13204541; doi:10.3390/biom16050652)
Supplement: Supplementary file 1 [file biomolecules-16-00652-s001.zip › biomolecules-4156387-supplementary.pdf]

S1: Abbreviations for the 72 brain regions are listed for the Left (L) and Right (R).

**Abbreviation**	**Brain Regions**
LCC	L_corpus_callosum
LCP	L_Caudoputamen
LACOL	L_anterior_commissure_olfactory_limb
LPAL	L_Pallidum_MODIF
LIntC	L_internal_capsule
LTH	L_Thalamus
LCB	L_Cerebellum
LSUC	L_Superior_colliculus_motor_related_MODIF
LVS	L_ventricular_systems
LHY	L_Hypothalamus_MODIF
LIC	L_Inferior_colliculus_MODIF
LPAG	L_Periaqueductal_gray
LICtx	L_Isocortex_MODIF
LCOA	L_Cortical_amygdalar_area_MODIF
LOlfA	L_Olfactory_areas_MODIF
LP	L_Pons
LRA	L_Midbrain_reticular_nucleus
LNA	L_Nucleus_accumbens
LF	L_fimbria
LACA	L_Anterior_cingulate_area
LMO	L_Somatomotor_areas
LSS	L_Somatosensory_areas
LPIR	L_Piriform_area
LTT	L_Taenia_tecta_MODIF
LMOB_gl	L_Accessory_olfactory_bulb_glomerular_layer
LMOB_gr	L_Accessory_olfactory_bulb_granular_layer
LRHP	L_Retrohippocampal_region_MODIF
LEC	L_Entorhinal_area
LCA1	L_Field_CA1
LCA3	L_Field_CA3
LDG	L_Dentate_gyrus

LCA2	L_Field_CA2_MODIF
LMOB_mi	L_Accessory_olfactory_bulb_mitral_layer
LSTR	L_Striatum_MODIF
LMB	L_Midbrain_MODIF
LMY	L_Medulla
RCC	R_corpus_callosum
RCP	R_Caudoputamen
RACOL	R_anterior_commissure_olfactory_limb
RPAL	R_Pallidum_MODIF
RIntC	R_internal_capsule
RTH	R_Thalamus
RCB	R_Cerebellum
RSUC	R_Superior_colliculus_motor_related_MODIF
RVS	R_ventricular_systems
RHY	R_Hypothalamus_MODIF
RIC	R_Inferior_colliculus_MODIF
RPAG	R_Periaqueductal_gray
RICtx	R_Isocortex_MODIF
RCOA	R_Cortical_amygdalar_area_MODIF
ROlfA	R_Olfactory_areas_MODIF
RP	R_Pons
RRA	R_Midbrain_reticular_nucleus
RNA	R_Nucleus_accumbens
RF	R_fimbria
RACA	R_Anterior_cingulate_area
RMO	R_Somatomotor_areas
RSS	R_Somatosensory_areas
RPIR	R_Piriform_area
RTT	R_Taenia_tecta_MODIF
RMOB_gl	R_Accessory_olfactory_bulb_glomerular_layer
RMOB_gr	R_Accessory_olfactory_bulb_granular_layer
RRHP	R_Retrohippocampal_region_MODIF
REC	R_Entorhinal_area

RCA1	R_Field_CA1
RCA3	R_Field_CA3
RDG	R_Dentate_gyrus
RCA2	R_Field_CA2_MODIF
RMOB_mi	R_Accessory_olfactory_bulb_mitral_layer
RSTR	R_Striatum_MODIF
RMB	R_Midbrain_MODIF
RMY	R_Medulla

S2. Diffusion parameters

Diffusion Parameters	Abbreviation	Definition
Fractional Anisotropy	FA	FA[44] quantifies the degrees of anisotropy (non-uniformity) of water diffusion in neuronal fibers. FA represents the degree to which diffusion within a voxel-of-interest in isotropic or anisotropic, with a value of 0 representing the former and 1 representing the latter FA is a fraction derived from the ratio between λ1, λ2, and λ3. It has a value ranged from 0 (isotropic) to 1 (totally anisotropic). FA reflects neuronal fiber integrity and coherency.
Axial Diffusivity	AD	AD [102] measure diffusion along the axonal orientation, reflecting axonal integrity. AD, denoted by λ parallel, quantifies how fast water diffuses along the axonal fibers[104-106]. It is estimated by λ1, the first eigenvalue of the tensor. AD can increase by axonal injury, traumatic injury, stroke, amyotrophic lateral sclerosis (ALS), advanced multiple sclerosis (MS), and neurodegenerative diseases.
Radial Diffusivity	RD	RD[102] measure diffusion perpendicular to the axonal orientation, reflecting myeline integrity. RD, denoted by λ perpendicular, quantified how fast water diffuses across the axonal bundles. It is estimated by (λ2+λ3)/2, the average of the second and third eigenvalues of the tensor. RD can increase by demyelination[107], such as in multiple sclerosis (MS), traumatic brain injury, DEE, metabolic disorders, and neurodegenerative diseases.
Mean Diffusivity or Apparent Diffusion Coefficient	MD or ADC	MD or ADC measures overall diffusivity for all directions[102]. MD is the diffusivity average from the three eigenvalues of the tensor. MD can increase by vasogenic edema. MD can decrease by tumors, immune cell infiltration, cytotoxic edema.

S3: Quantitative diffusivity and P-values comparing *Cdkl5* KO vs WT mice for each mouse brain region.

Region Name	Abbv	FA			MD			AD			RD		
		WT	Cdkl5	p	WT	Cdkl5	p	WT	Cdkl5	p	WT	Cdkl5	p
L_corpus_callosum	LCC	0.3874	0.3151	0.0004	0.3874	0.2756	0.1185	0.4711	0.3697	0.0001	0.2619	0.2311	0.9983
L_Caudoputamen	LCP	0.1872	0.1699	0.9999	0.1872	0.3000	0.2241	0.4260	0.3615	0.1031	0.3215	0.2740	0.5045
L_anterior_commissure_olfactory_limb	LACOL	0.3784	0.3846	0.9999	0.3784	0.2707	0.0733	0.4804	0.4039	0.0120	0.2587	0.2096	0.3349
L_Pallidum_MODIF	LPAL	0.3121	0.2688	0.6165	0.3121	0.2749	0.0310	0.4503	0.3580	0.0004	0.2763	0.2348	0.4489
L_internal_capsule	LIntC	0.5331	0.4996	0.9300	0.5331	0.2742	0.1536	0.5373	0.4401	0.0001	0.2141	0.1887	0.9983
L_Thalamus	LTH	0.2615	0.2481	0.9999	0.2615	0.2908	0.0725	0.4421	0.3681	0.0196	0.3006	0.2534	0.2661
L_Cerebellum	LCB	0.2070	0.1889	0.9999	0.2070	0.3178	0.9999	0.4217	0.3880	0.9993	0.3036	0.2863	0.9999
L_Superior_colliculus_motor_related_MODIF	LSUC	0.1885	0.1671	0.9999	0.1885	0.2884	0.0256	0.4140	0.3377	0.0124	0.3156	0.2653	0.0817
L_ventricular_systems	LVS	0.1914	0.1606	0.9999	0.1914	0.4084	0.9999	0.6136	0.5441	0.9999	0.4602	0.3761	0.9999
L_Hypothalamus_MODIF	LHY	0.2227	0.2233	0.9999	0.2227	0.2633	0.0001	0.4292	0.3293	0.0001	0.3104	0.2322	0.0001
L_Inferior_colliculus_MODIF	LIC	0.1964	0.1787	0.9999	0.1964	0.3166	0.7827	0.4269	0.3795	0.7566	0.3241	0.2891	0.8947
L_Periaqueductal_gray	LPAG	0.1983	0.1811	0.9999	0.1983	0.2637	0.0002	0.4172	0.3189	0.0001	0.3120	0.2397	0.0012
L_Isocortex_MODIF	LICtx	0.1563	0.1411	0.9999	0.1563	0.2951	0.0058	0.4322	0.3527	0.0066	0.3424	0.2741	0.0130
L_Cortical_amygdalar_area_MODIF	LCOA	0.1447	0.1354	0.9999	0.1447	0.2949	0.0001	0.4471	0.3480	0.0001	0.3635	0.2741	0.0001
L_Olfactory_areas_MODIF	LOlfA	0.1952	0.1621	0.9999	0.1952	0.3109	0.2026	0.4559	0.3853	0.0364	0.3323	0.2836	0.6520
L_Pons	LP	0.2632	0.2653	0.9999	0.2632	0.2791	0.0021	0.4531	0.3654	0.0012	0.2999	0.2375	0.0078
L_Midbrain_reticular_nucleus	LRA	0.2416	0.2288	0.9999	0.2416	0.2743	0.1147	0.4125	0.3416	0.0348	0.2849	0.2413	0.3634
L_Nucleus_accumbens	LNA	0.1830	0.1618	0.9999	0.1830	0.3080	0.1631	0.4307	0.3665	0.1078	0.3311	0.2831	0.3331
L_fimbria	LF	0.5527	0.4955	0.8625	0.5527	0.2911	0.5214	0.5544	0.4577	0.0001	0.2140	0.2079	0.9999
L_Anterior_cingulate_area	LACA	0.1915	0.1636	0.9988	0.1915	0.2925	0.0057	0.4367	0.3502	0.0015	0.3346	0.2704	0.0299
L_Somatomotor_areas	LMO	0.1544	0.1194	0.9986	0.1544	0.2905	0.0083	0.4245	0.3414	0.0032	0.3353	0.2726	0.0347
L_Somatosensory_areas	LSS	0.1424	0.1274	0.9999	0.1424	0.2971	0.0035	0.4287	0.3505	0.0086	0.3488	0.2783	0.0050
L_Piriform_area	LPIR	0.1832	0.1468	0.9906	0.1832	0.3081	0.0001	0.4697	0.3684	0.0001	0.3585	0.2841	0.0009
L_Taenia_tecta_MODIF	LTT	0.1872	0.1786	0.9999	0.1872	0.3090	0.1084	0.4408	0.3772	0.1176	0.3355	0.2803	0.1783
L_Accessory_olfactory_bulb_glomerular_layer	LMOB_gl	0.1502	0.1343	0.9999	0.1502	0.3076	0.9999	0.3681	0.3604	0.9999	0.2836	0.2868	0.9999
L_Accessory_olfactory_bulb_granular_layer	LMOB_gr	0.2003	0.1652	0.9795	0.2003	0.2758	0.0001	0.4478	0.3429	0.0001	0.3337	0.2518	0.0008
L_Retrohippocampal_region_MODIF	LRHP	0.2273	0.2000	0.9995	0.2273	0.3093	0.0444	0.4577	0.3802	0.0099	0.3306	0.2781	0.1954
L_Entorhinal_area	LEC	0.1586	0.1459	0.9999	0.1586	0.3001	0.0005	0.4431	0.3530	0.0007	0.3476	0.2767	0.0012
L_Field_CA1	LCA1	0.2061	0.2068	0.9999	0.2061	0.3172	0.0340	0.4593	0.3913	0.0570	0.3397	0.2829	0.0488
L_Field_CA3	LCA3	0.2080	0.2078	0.9999	0.2080	0.3348	0.3510	0.4691	0.4158	0.4640	0.3453	0.2988	0.4167
L_Dentate_gyrus	LDG	0.1795	0.1826	0.9999	0.1795	0.3349	0.0223	0.4769	0.4100	0.0693	0.3660	0.3031	0.0217
L_Field_CA2_MODIF	LCA2	0.2492	0.2226	0.9999	0.2492	0.3174	0.3157	0.4629	0.3945	0.0536	0.3207	0.2822	0.8295
L_Accessory_olfactory_bulb_mitral_layer	LMOB_mi	0.1735	0.1474	0.9999	0.1735	0.3034	0.6178	0.4213	0.3609	0.1906	0.3201	0.2810	0.9632
L_Striatum_MODIF	LSTR	0.2316	0.1923	0.8158	0.2316	0.2947	0.0094	0.4545	0.3622	0.0004	0.3200	0.2657	0.1152
L_Midbrain_MODIF	LMB	0.2730	0.2521	0.9999	0.2730	0.2800	0.0290	0.4355	0.3535	0.0039	0.2887	0.2430	0.1742
L_Medulla	LMY	0.2211	0.2032	0.9999	0.2211	0.2776	0.0004	0.4465	0.3458	0.0001	0.3139	0.2468	0.0041
R_corpus_callosum	RCC	0.3943	0.3243	0.0006	0.3943	0.2771	0.6135	0.4685	0.3819	0.0015	0.2581	0.2310	0.9999
R_Caudoputamen	RCP	0.1927	0.1814	0.9999	0.1927	0.3079	0.6934	0.4272	0.3747	0.5065	0.3215	0.2796	0.9022
R_anterior_commissure_olfactory_limb	RACOL	0.4009	0.3915	0.9999	0.4009	0.2763	0.4240	0.4851	0.4121	0.0235	0.2510	0.2145	0.9852
R_Pallidum_MODIF	RPAL	0.3188	0.2702	0.1936	0.3188	0.2743	0.1669	0.4526	0.3684	0.0025	0.2755	0.2342	0.9250
R_internal_capsule	RIntC	0.5607	0.4980	0.0034	0.5607	0.2751	0.9137	0.5402	0.4513	0.0009	0.2012	0.1890	0.9999
R_Thalamus	RTH	0.2660	0.2409	0.9999	0.2660	0.2937	0.4576	0.4411	0.3773	0.1146	0.2976	0.2572	0.9014
R_Cerebellum	RCB	0.2039	0.1877	0.9999	0.2039	0.3141	0.9999	0.4190	0.3919	0.9999	0.3036	0.2832	0.9999
R_Superior_colliculus_motor_related_MODIF	RSUC	0.1912	0.1695	0.9999	0.1912	0.2846	0.0310	0.4155	0.3410	0.0177	0.3151	0.2609	0.0880
R_ventricular_systems	RVS	0.1884	0.1728	0.9999	0.1884	0.4380	0.9999	0.6430	0.5677	0.9999	0.4814	0.4011	0.9999
R_Hypothalamus_MODIF	RHY	0.2266	0.1975	0.9991	0.2266	0.2614	0.0018	0.4249	0.3322	0.0004	0.3055	0.2352	0.0128
R_Inferior_colliculus_MODIF	RIC	0.2043	0.1811	0.9999	0.2043	0.3074	0.5491	0.4288	0.3768	0.5306	0.3212	0.2798	0.7075

R_Periaqueductal_gray	RPAG	0.2077	0.1789	0.9965	0.2077	0.2686	0.0007	0.4187	0.3206	0.0001	0.3082	0.2448	0.0066
R_Isocortex_MODIF	RICtx	0.1620	0.1520	0.9999	0.1620	0.3059	0.3806	0.4252	0.3697	0.3623	0.3340	0.2834	0.5428
R_Cortical_amygdalar_area_MODIF	RCOA	0.1522	0.1392	0.9999	0.1522	0.3126	0.0006	0.4609	0.3778	0.0032	0.3683	0.2898	0.0006
R_Olfactory_areas_MODIF	ROlfA	0.2076	0.1563	0.5671	0.2076	0.3128	0.2363	0.4594	0.3837	0.0141	0.3286	0.2865	0.8758
R_Pons	RP	0.2641	0.2594	0.9999	0.2641	0.2786	0.0123	0.4505	0.3707	0.0063	0.2976	0.2380	0.0417
R_Midbrain_reticular_nucleus	RRA	0.2516	0.2363	0.9999	0.2516	0.2666	0.0796	0.4162	0.3423	0.0201	0.2845	0.2333	0.2961
R_Nucleus_accumbens	RNA	0.1910	0.1722	0.9999	0.1910	0.3091	0.7721	0.4312	0.3818	0.6597	0.3279	0.2828	0.9206
R_fimbria	RF	0.5646	0.5181	0.0332	0.5646	0.3068	0.9999	0.5898	0.5120	0.0093	0.2248	0.2148	0.9999
R_Anterior_cingulate_area	RACA	0.2076	0.1481	0.1258	0.2076	0.2901	0.0104	0.4291	0.3408	0.0010	0.3240	0.2700	0.0858
R_Somatomotor_areas	RMO	0.1445	0.1246	0.9999	0.1445	0.2845	0.0086	0.4176	0.3412	0.0123	0.3356	0.2666	0.0160
R_Somatosensory_areas	RSS	0.1263	0.1287	0.9999	0.1263	0.3053	0.2711	0.4155	0.3653	0.6206	0.3446	0.2861	0.2116
R_Piriform_area	RPIR	0.1877	0.1598	0.9999	0.1877	0.3309	0.0342	0.4794	0.4085	0.0342	0.3645	0.3036	0.0684
R_Taenia_tecta_MODIF	RTT	0.2022	0.1733	0.9992	0.2022	0.3181	0.4206	0.4506	0.3888	0.1563	0.3344	0.2897	0.8104
R_Accessory_olfactory_bulb_glomerular_layer	RMOB_gl	0.1243	0.1280	0.9999	0.1243	0.3199	0.2611	0.4209	0.3691	0.5436	0.3471	0.2998	0.2280
R_Accessory_olfactory_bulb_granular_layer	RMOB_gr	0.2335	0.1786	0.0359	0.2335	0.3016	0.0047	0.4669	0.3647	0.0001	0.3307	0.2755	0.1347
R_Retrohippocampal_region_MODIF	RRHP	0.2454	0.2043	0.7859	0.2454	0.3099	0.2041	0.4601	0.3874	0.0247	0.3229	0.2779	0.7257
R_Entorhinal_area	REC	0.1692	0.1549	0.9999	0.1692	0.2937	0.0113	0.4419	0.3664	0.0146	0.3409	0.2701	0.0219
R_Field_CA1	RCA1	0.2113	0.2036	0.9999	0.2113	0.3167	0.1885	0.4562	0.3943	0.1540	0.3366	0.2838	0.3355
R_Field_CA3	RCA3	0.2123	0.2358	0.9999	0.2123	0.3368	0.7918	0.4680	0.4281	0.9736	0.3410	0.2955	0.7067
R_Dentate_gyrus	RDG	0.1849	0.1777	0.9999	0.1849	0.3446	0.6470	0.4677	0.4173	0.6148	0.3551	0.3128	0.7985
R_Field_CA2_MODIF	RCA2	0.2520	0.2303	0.9999	0.2520	0.3168	0.3491	0.4621	0.3952	0.0700	0.3193	0.2804	0.8402
R_Accessory_olfactory_bulb_mitral_layer	RMOB_mi	0.1596	0.1533	0.9999	0.1596	0.3176	0.3079	0.4307	0.3713	0.2224	0.3381	0.2944	0.5271
R_Striatum_MODIF	RSTR	0.2259	0.2114	0.9999	0.2259	0.2926	0.0074	0.4586	0.3742	0.0024	0.3267	0.2602	0.0344
R_Midbrain_MODIF	RMB	0.2753	0.2513	0.9997	0.2753	0.2761	0.1003	0.4350	0.3587	0.0127	0.2877	0.2399	0.4655
R_Medulla	RMY	0.2224	0.2029	0.9999	0.2224	0.2765	0.0006	0.4497	0.3514	0.0001	0.3159	0.2458	0.0058

S4. Streamlines for each mouse brain region.

ROI	WT Streamlines	Standard Dev	Cdkl5 Streamlines	Standard Dev	P value
LCC	65285	3995.279894	75917	12941.22852	0.038278
LCP	82432	8844.104053	83652	12593.7795	0.815427
LACOL	4365	1256.957296	3787	695.4461398	0.201
LPAL	41173	7705.429843	46215	6463.58659	0.130587
LIntC	9550	1746.887191	8489	2517.758088	0.315214
LTH	59339	4931.114037	53953	6747.963686	0.069172
LCB	108826	22980.95807	66878	13198.3389	0.00006
LSUC	46406	11684.08813	36310	5661.890005	0.018305
LVS	67853	9809.931757	68344	15959.52281	0.939136
LHY	50463	3818.833769	56984	7197.710587	0.031116
LIC	14530	4448.70221	21347	7078.626335	0.026718
LPAG	32330	6379.323811	30145	6807.125269	0.480629
LICtx	174405	20855.06861	166237	25302.27565	0.459465
LCOA	25019	9688.164971	12357	4286.85917	0.000812
LOlfA	42676	4635.305807	47664	12581.86841	0.300527
LP	39510	6618.039886	47258	14126.17339	0.167046
LRA	13433	3296.816126	13609	3498.795685	0.911438
LNA	16706	3819.413867	15621	3790.551939	0.539647
LF	6733	2273.820385	6172	2512.682805	0.618487
LACA	19623	3744.284788	24861	8357.975498	0.11549
LMO	60211	10379.41782	45025	11970.66331	0.009063
LSS	77158	9977.042605	65507	10856.27186	0.026023
LPIR	37562	8489.704286	24736	6510.520763	0.001236
LTT	37052	8915.538054	25738	5288.835709	0.002151
LMOB_gl	2282	954.3598827	1525	881.3879378	0.085334
LMOB_gr	1320	670.8842779	2058	1328.735409	0.165861
LRHP	27914	5963.512675	35271	6192.10548	0.016624
LEC	28597	5481.824507	35328	10531.17708	0.11539
LCA1	34262	767.9447525	35699	4645.232121	0.401185
LCA3	38487	3887.818182	35120	7480.187704	0.258999
LDG	43773	2895.137191	40924	9381.209438	0.419383
LCA2	8706	1945.913942	10096	2526.065404	0.205403
LMOB_mi	2468	998.9410107	1828	910.5074842	0.155633

LSTR	67066	6082.171552	71255	11014.64147	0.342345
LMB	61305	6611.208054	75691	12357.16823	0.007669
LMY	45447	10235.29133	52666	19058.15215	0.342103
RCC	52549	6807.610195	63496	16063.31415	0.087135
RCP	72791	8318.738087	70268	10817.0712	0.584323
RACOL	5032	692.1225635	4060	1108.946142	0.04105
RPAL	40485	5936.529024	44214	8940.860976	0.315312
RIntC	13224	3530.117835	7604	1643.359272	0.000134
RTH	57634	4625.66369	54026	7084.86668	0.22167
RCB	104041	16219.92288	71128	11574.71044	0.000047
RSUC	29475	9625.290556	36076	13901.63053	0.259244
RVS	58610	6372.611911	65234	16173.31311	0.287896
RHY	57267	8982.366086	51218	12974.81326	0.267708
RIC	17605	2595.361136	21132	9914.256199	0.342036
RPAG	19199	5404.746697	28056	5361.76381	0.002011
RICtx	156597	17432.62868	160236	30643.92741	0.765347
RCOA	29785	13433.80763	16293	5256.349822	0.005324
ROlfA	41348	9343.248345	56595	11306.93626	0.005474
RP	37555	4012.941778	47889	12429.38098	0.036716
RRA	11820	2057.535679	11411	2012.756831	0.664277
RNA	14824	3039.211129	15951	6150.599044	0.638329
RF	4690	899.4295017	9053	4430.028141	0.013887
RACA	15982	2210.107884	23415	4253.98742	0.000263
RMO	57888	9464.407819	50935	12268.94197	0.192873
RSS	72045	15827.24638	65342	18464.02568	0.411993
RPIR	54873	25740.93087	28947	9233.679433	0.004674
RTT	32070	7553.679141	31339	5816.167217	0.809513
RMOB_gl	1748	492.3958918	1794	800.3761994	0.88745
RMOB_gr	1249	624.5079778	1939	871.0797897	0.069811
RRHP	21385	6764.908441	27831	5110.57054	0.02575
REC	38899	13308.85758	52860	20291.29377	0.104724
RCA1	36200	2340.510918	38885	7622.729949	0.350244
RCA3	33449	3023.690838	35204	7576.028071	0.543987
RDG	45140	4618.346024	41984	11485.08129	0.472851
RCA2	8906	1443.399954	10134	2711.016867	0.258052
RMOB_mi	2165	377.1861988	2192	834.5988512	0.93132

RSTR	70501	9697.388514	70348	17606.96965	0.982418
RMB	62533	6475.959317	71774	15331.99068	0.12686
RMY	52982	11040.8449	59198	21963.31374	0.471075

S5. Adjust p values for group comparisons of pair-wised connectivity for the whole brain.

Region Connections	Whole Brain			
	Left Ipsilateral	Left Contralateral	Right Contralateral	Right Ipsilateral
Caudoputamen-corpus callosum	0.043424	-0.06279	-0.32737	-0.54632
anterior commissure olfactory limb-corpus callosum	0.065323	0	0	0.344478
pallidum -corpus callosum	0.874734	-0.01325	-0.97736	-0.25859
internal capsule-corpus callosum	-0.96814	0	0	-0.09702
Thalamus-corpus callosum	-0.2123	-0.01944	-0.15355	-0.00393
Cerebellum-corpus callosum	-0.09393	-0.00067	-0.10612	-0.01254
Superior colliculus-corpus callosum	-0.06532	-0.11337	-0.00903	-0.00873
ventricular systems-corpus callosum	0.206175	-0.00488	0.031169	-0.27258
Hypothalamus -corpus callosum	-0.81629	-0.00271	-0.93709	-0.00195
Inferior colliculus -corpus callosum	-0.03488	-0.01833	-0.12536	-0.00295
periaqueductal gray-corpus callosum	-0.01686	-0.0009	-0.0042	-0.00087
Isocortex -corpus callosum	0.572009	-0.47418	-0.03193	-0.19801
Cortical amygdalar area -corpus callosum	0.334363	-0.000027	-0.33777	0.544067
Olfactory areas -corpus callosum	-0.77488	-0.32024	0.838228	-0.00995
Pons-corpus callosum	-0.00073	-0.000075	-0.00404	-0.0011
Midbrain reticular nucleus-corpus callosum	-0.01778	0	-0.06397	-0.00124
Nucleus accumbens-corpus callosum	0.014936	0.96795	0.402522	0.038273
fimbria-corpus callosum	0.347511	0	0	0.237381
Anterior cingulate area-corpus callosum	-0.03053	0.890016	-0.03985	-0.0467
Somatomotor areas-corpus callosum	0.099083	0.060462	-0.86983	0.428281
Somatosensory areas-corpus callosum	0.142671	0.373839	0.605563	-0.31267
piriform area-corpus callosum	0.125299	0.896678	0.505582	0.2633
Taenia tecta -corpus callosum	0.018062	0.536398	0.077726	0.762914
Accessory olfactory bulb glomerular layer-corpus callosum	0	0	0	0
Accessory olfactory bulb granular layer-corpus callosum	0	0	0	0
Retrohippocampal region -corpus callosum	0.874734	-0.00273	-0.00404	-0.00398
Entorhinal area-corpus callosum	-0.02075	-0.00079	-0.00808	-0.1247
Field CA1-corpus callosum	0.240871	-0.07729	0.719436	-0.0467
Field CA3-corpus callosum	0.049219	-0.00062	-0.5058	-0.06444
Dentate gyrus-corpus callosum	0.511285	-0.00503	-0.89451	-0.05411
Field CA2 -corpus callosum	0.017851	-0.03202	-0.10575	-0.08275
Accessory olfactory bulb mitral layer-corpus callosum	0	0	0	0

Striatum -corpus callosum	0.150439	-0.00995	0.891522	-0.08214
Midbrain -corpus callosum	-0.00388	-0.00067	-0.00626	-0.00265
Medulla-corpus callosum	-0.00073	-0.000026	-0.04315	-0.00147
anterior commissure olfactory limb-Caudoputamen	0.006276	0	0	0.010723
pallidum -Caudoputamen	0.002602	-0.028361	0.842777	0.007538
internal capsule-Caudoputamen	0.040475	-0.02047	-0.505582	0.011103
Thalamus-Caudoputamen	0.020765	-0.000956	-0.114242	-0.860565
Cerebellum-Caudoputamen	-0.083122	-0.000182	-0.128671	-0.007538
Superior colliculus-Caudoputamen	-0.369061	-0.150228	-0.015039	-0.004068
ventricular systems-Caudoputamen	0.961314	-0.029382	0.692354	-0.655941
Hypothalamus -Caudoputamen	0.188489	-0.000895	0.744273	-0.00753
Inferior colliculus -Caudoputamen	-0.034769	-0.000873	-0.031169	-0.001208
periaqueductal gray-Caudoputamen	-0.070739	-0.00086	-0.00468	-0.000866
Isocortex -Caudoputamen	0.965014	-0.896678	-0.004204	-0.750299
Cortical amygdalar area -Caudoputamen	0.076879	0	0	0.060771
Olfactory areas -Caudoputamen	-0.960728	-0.249894	-0.664627	-0.348501
Pons-Caudoputamen	-0.001302	-0.000056	-0.00468	-0.000866
Midbrain reticular nucleus-Caudoputamen	-0.074859	-0.008293	-0.00593	-0.000866
Nucleus accumbens-Caudoputamen	0.009991	0.600434	-0.91361	0.065081
fimbria-Caudoputamen	0.04246	0	0	0.013885
Anterior cingulate area-Caudoputamen	-0.201664	-0.981985	-0.005787	-0.870062
Somatomotor areas-Caudoputamen	0.558885	0.53745	-0.257442	0.045337
Somatosensory areas-Caudoputamen	0.019443	0.580476	-0.215262	-0.965527
piriform area-Caudoputamen	0.078184	0.833869	0.71632	0.039511
Taenia tecta -Caudoputamen	0.02213	0.179953	0.29043	0.089813
Accessory olfactory bulb glomerular layer-Caudoputamen	0	0	0	0
Accessory olfactory bulb granular layer-Caudoputamen	0	0	0	0
Retrohippocampal region -Caudoputamen	-0.014681	-0.00046	-0.004044	-0.001346
Entorhinal area-Caudoputamen	-0.003033	-0.000326	-0.022719	-0.082347
Field CA1-Caudoputamen	-0.849965	-0.010282	-0.043154	-0.788491
Field CA3-Caudoputamen	0.001532	-0.001057	-0.127088	0.070533
Dentate gyrus-Caudoputamen	0.584996	-0.010282	-0.856004	0.515069
Field CA2 -Caudoputamen	0.18058	-0.507893	-0.020927	0.979117
Accessory olfactory bulb mitral layer-Caudoputamen	0	0	0	0
Striatum -Caudoputamen	0.001081	-0.25452	0.708717	0.261565
Midbrain -Caudoputamen	-0.007441	-0.000456	-0.006616	-0.000866

Medulla-Caudoputamen	-0.001302	-0.000056	-0.022719	-0.001299
pallidum -anterior commissure olfactory limb	-0.7809	0.003397	-0.605563	0.185799
internal capsule-anterior commissure olfactory limb	0	0	0	0
Thalamus-anterior commissure olfactory limb	0	0	0	0
Cerebellum-anterior commissure olfactory limb	0	0	0	0
Superior colliculus-anterior commissure olfactory limb	0	0	0	0
ventricular systems-anterior commissure olfactory limb	0.693148	0	0	0.747102
Hypothalamus -anterior commissure olfactory limb	0.038908	0.368161	0.187973	0.038408
Inferior colliculus -anterior commissure olfactory limb	0	0	0	0
periaqueductal gray-anterior commissure olfactory limb	0	0	0	0
Isocortex -anterior commissure olfactory limb	-0.88891	0.113367	0	-0.46812
Cortical amygdalar area -anterior commissure olfactory limb	0	0	0	0
Olfactory areas -anterior commissure olfactory limb	0.935149	-0.819066	-0.288288	-0.576715
Pons-anterior commissure olfactory limb	0	0	0	0
Midbrain reticular nucleus-anterior commissure olfactory limb	0	0	0	0
Nucleus accumbens-anterior commissure olfactory limb	0.100636	0	-0.029966	0.168385
fimbria-anterior commissure olfactory limb	0	0	0	0
Anterior cingulate area-anterior commissure olfactory limb	0	0	0	0
Somatomotor areas-anterior commissure olfactory limb	0	0	0	0
Somatosensory areas-anterior commissure olfactory limb	0	0	0	0
piriform area-anterior commissure olfactory limb	-0.039869	0	0	0.095313
Taenia tecta -anterior commissure olfactory limb	0.233817	0	0	-0.004213
Accessory olfactory bulb glomerular layer-anterior commissure olfactory limb	0	0	0	0
Accessory olfactory bulb granular layer-anterior commissure olfactory limb	0	0	0	0.540016
Retrohippocampal region -anterior commissure olfactory limb	0	0	0	0
Entorhinal area-anterior commissure olfactory limb	-0.014681	0	0	0
Field CA1-anterior commissure olfactory limb	0	0	0	0
Field CA3-anterior commissure olfactory limb	-0.39923	0	0	0
Dentate gyrus-anterior commissure olfactory limb	0	0	0	0
Field CA2 -anterior commissure olfactory limb	0	0	0	0
Accessory olfactory bulb mitral layer-anterior commissure olfactory limb	0	0	0	0
Striatum -anterior commissure olfactory limb	0.341189	0.240192	-0.327367	0.039511
Midbrain -anterior commissure olfactory limb	0	0	0	0
Medulla-anterior commissure olfactory limb	0	0	0	0

internal capsule-pallidum	0.546808	0	0	0.059256
Thalamus-pallidum	0.406194	0.96795	-0.601298	0.027405
Cerebellum-pallidum	-0.116701	-0.000873	-0.06984	-0.011309
Superior colliculus-pallidum	-0.085435	-0.553856	-0.025059	-0.008958
ventricular systems-pallidum	-0.014681	-0.39196	-0.243067	-0.10313
Hypothalamus -pallidum	0.523752	-0.01181	-0.934361	-0.144016
Inferior colliculus -pallidum	-0.04314	-0.087972	0	-0.001625
periaqueductal gray-pallidum	-0.014655	-0.002155	-0.092358	-0.001962
Isocortex -pallidum	-0.115741	-0.819066	-0.018705	-0.007538
Cortical amygdalar area -pallidum	-0.870264	0	0.369981	0.015318
Olfactory areas -pallidum	-0.427431	-0.570308	0.62709	-0.515516
Pons-pallidum	-0.003033	-0.000406	-0.020927	-0.001425
Midbrain reticular nucleus-pallidum	-0.006276	0	0	-0.001469
Nucleus accumbens-pallidum	-0.963004	-0.311973	-0.888496	-0.025741
fimbria-pallidum	-0.003033	-0.045538	0	-0.011502
AVA-pallidum	-0.014681	0	-0.005402	-0.818759
Somatomotor areas-pallidum	0.812256	0.597377	-0.327367	0.072086
Somatosensory areas-pallidum	0.219974	0.013577	-0.063968	-0.069794
piriform area-pallidum	0.290702	0.79032	0.91903	0.166798
Taenia tecta -pallidum	0.038553	0.181621	0.014025	0.237381
Accessory olfactory bulb glomerular layer-pallidum	0	0	0	0
Accessory olfactory bulb granular layer-pallidum	0	0	0	0
Retrohippocampal region -pallidum	-0.008964	-0.003665	-0.004044	-0.001337
Entorhinal area-pallidum	-0.001302	-0.00085	-0.029869	-0.003396
Field CA1-pallidum	-0.008123	-0.000588	-0.004204	-0.00753
Field CA3-pallidum	-0.048708	-0.002049	-0.005402	-0.015922
Dentate gyrus-pallidum	-0.961314	-0.053901	-0.014578	-0.828153
Field CA2 -pallidum	-0.009317	-0.003881	-0.004044	-0.003476
Accessory olfactory bulb mitral layer-pallidum	0	0	0	0
Striatum -pallidum	0.1054	-0.328523	0.275862	0.107899
Midbrain -pallidum	-0.005886	-0.000464	-0.004044	-0.003084
Medulla-pallidum	-0.005886	-0.000027	-0.241348	-0.003295
Thalamus-internal capsule	0.295821	-0.062789	-0.253627	0.079145
Cerebellum-internal capsule	0.014681	0	0	0.089691
Superior colliculus-internal capsule	0.965014	0	0	-0.001837

ventricular systems-internal capsule	-0.472855	0.981985	-0.18297	-0.792292
Hypothalamus -internal capsule	0.04246	-0.567403	0.069133	0.231961
Inferior colliculus -internal capsule	-0.034876	0	0	-0.013549
periaqueductal gray-internal capsule	-0.480362	0	0	-0.001425
Isocortex -internal capsule	-0.131579	0.152056	-0.273536	-0.234933
Cortical amygdalar area -internal capsule	0	0	0	0
Olfactory areas -internal capsule	-0.1054	0	0	0.0119
Pons-internal capsule	-0.056766	-0.06882	0	-0.001851
Midbrain reticular nucleus-internal capsule	0.823892	0	0	-0.085196
Nucleus accumbens-internal capsule	0	0	0	0
fimbria-internal capsule	0.233817	0	0	0
Anterior cingulate area-internal capsule	0	0	0	0
Somatomotor areas-internal capsule	0	0	0	0.001225
Somatosensory areas-internal capsule	0.171368	0	0	-0.836179
piriform area-internal capsule	0	0	0	0
Taenia tecta -internal capsule	0	0	0	0
Accessory olfactory bulb glomerular layer-internal capsule	0	0	0	0
Accessory olfactory bulb granular layer-internal capsule	0	0	0	0
Retrohippocampal region -internal capsule	0.88891	0	0	-0.011422
Entorhinal area-internal capsule	0	0	0	0
Field CA1-internal capsule	-0.424323	0	0	0.308887
Field CA3-internal capsule	0.014655	0	0	0.007538
Dentate gyrus-internal capsule	0.391328	0	0	0.005683
Field CA2 -internal capsule	0	0	0	0
Accessory olfactory bulb mitral layer-internal capsule	0	0	0	0
Striatum -internal capsule	0.030016	0	0	0.000898
Midbrain --internal capsule	-0.076879	-0.050635	-0.49621	-0.000866
Medulla-internal capsule	0	0	0	-0.056909
Cerebellum-Thalamus	0.006276	0.461447	0.003247	0.002491
Superior colliculus-Thalamus	0.341596	0.010078	-0.77473	-0.65594
ventricular systems-Thalamus	0.279326	0.947326	-0.04064	-0.1154
Hypothalamus -Thalamus	0.020215	-0.34389	0.006774	0.003151
Inferior colliculus -Thalamus	-0.01944	-0.00699	0.094199	0.450388
periaqueductal gray-Thalamus	0.641145	0.981985	-0.66463	-0.04831
Isocortex -Thalamus	-0.159	0.96795	-0.07773	-0.05926

Cortical amygdalar area -Thalamus	0.977013	0	0	0.013577
Olfactory areas -Thalamus	0.42563	0.274991	0.039048	0.031119
Pons-Thalamus	-0.07818	-0.00809	-0.06657	-0.00753
Midbrain reticular nucleus-Thalamus	0.88891	-0.34389	0.844714	-0.04122
Nucleus accumbens-Thalamus	0.048708	0	0.036547	0.098964
fimbria-Thalamus	0.295821	0	0	0.870062
Anterior cingulate area-Thalamus	0	0	0	-0.31196
Somatomotor areas-Thalamus	-0.1178	-0.63504	-0.18654	0.854537
Somatosensory areas-Thalamus	0.198676	0.096435	-0.02666	-0.05926
piriform area-Thalamus	0.871323	0.643298	0.813099	0.282744
Taenia tecta -Thalamus	0.012338	0.117032	0.074563	0.003644
Accessory olfactory bulb glomerular layer-Thalamus	0	0	0	0
Accessory olfactory bulb granular layer-Thalamus	0	0	0	0
Retrohippocampal region -Thalamus	-0.07818	-0.99378	-0.13485	-0.01933
Entorhinal area-Thalamus	-0.01633	-0.00829	-0.23501	-0.38882
Field CA1-Thalamus	-0.04099	-0.37722	0.745004	-0.06077
Field CA3-Thalamus	0.186374	-0.82161	-0.54934	0.190827
Dentate gyrus-Thalamus	-0.6854	0.819066	0.181131	0.019394
Field CA2 -Thalamus	-0.06607	-0.65244	-0.06453	-0.19953
Accessory olfactory bulb mitral layer-Thalamus	0	0	0	0
Striatum -Thalamus	0.007782	-0.31197	0.026537	0.003295
Midbrain -Thalamus	-0.27979	-0.22988	0.733657	-0.03951
Medulla-Thalamus	-0.01391	-0.00018	-0.21495	-0.00626
Superior colliculus-Cerebellum	0.008833	0.002615	0.691915	0.000898
ventricular systems-Cerebellum	0.091218	0.005731	0.839489	0.047883
Hypothalamus -Cerebellum	0.008123	0.407025	0.214951	0.002858
Inferior colliculus -Cerebellum	0.129424	0.059216	-0.40252	0.138113
periaqueductal gray-Cerebellum	0.021424	0.000895	-0.32529	0.112902
Isocortex -Cerebellum	0.049429	0.004994	-0.01788	0.045337
Cortical amygdalar area -Cerebellum	0.073312	0.020312	0	0.046224
Olfactory areas -Cerebellum	-0.0521	-0.10451	-0.02015	0.841135
Pons-Cerebellum	0.983778	-0.17347	-0.1246	-0.64295
Midbrain reticular nucleus-Cerebellum	0.010296	0.085947	-0.856	0.002936
Nucleus accumbens-Cerebellum	0	0	0	-0.09531
fimbria-Cerebellum	0	0	0	0

Anterior cingulate area-Cerebellum	0	0	0	-0.11481
Somatomotor areas-Cerebellum	-0.01468	-0.11584	-0.00727	-0.31196
Somatosensory areas-Cerebellum	0.796481	0.981985	-0.00673	-0.04982
piriform area-Cerebellum	0.965014	0	-0.63284	0.287694
Taenia tecta -Cerebellum	-0.04605	-0.17997	-0.00626	-0.03136
Accessory olfactory bulb glomerular layer-Cerebellum	0	0	0	0
Accessory olfactory bulb granular layer-Cerebellum	0	0	0	0
Retrohippocampal region -Cerebellum	0.037948	0.036022	-0.18778	0.013218
Entorhinal area-Cerebellum	0.006966	0.71889	-0.83767	0.038408
Field CA1-Cerebellum	0.459217	0.96795	-0.08312	0.074849
Field CA3-Cerebellum	0.001384	0.04245	-0.68808	0.001208
Dentate gyrus-Cerebellum	0.004965	0.031015	0.744175	0.000866
Field CA2 -Cerebellum	-0.24456	0	-0.13695	0.274103
Accessory olfactory bulb mitral layer-Cerebellum	0	0	0	0
Striatum -Cerebellum	-0.7637	-0.20021	-0.02015	0.965527
Midbrain -Cerebellum	0.017851	0.004419	-0.45926	0.001208
Medulla-Cerebellum	0.115741	-0.20264	0.063968	0.46434
ventricular systems-Superior colliculus	0.038908	0.445765	0.303819	-0.29133
Hypothalamus -Superior colliculus	0.148539	-0.17821	0.004847	-0.597
Inferior colliculus -Superior colliculus	0.340778	-0.02031	0.005402	0.011103
periaqueductal gray-Superior colliculus	0.738852	-0.07899	0.235005	-0.00293
Isocortex -Superior colliculus	0.186374	-0.62283	-0.68123	0.980568
Cortical amygdalar area -Superior colliculus	-0.96501	0	0	0.018546
Olfactory areas -Superior colliculus	-0.04099	-0.05843	-0.63153	-0.0464
Pons-Superior colliculus	-0.34146	-0.03184	0.166945	-0.05199
Midbrain reticular nucleus-Superior colliculus	-0.53142	-0.00369	0.271278	-0.00147
Nucleus accumbens-Superior colliculus	-0.17225	0	0	0
fimbria-Superior colliculus	0.921098	0	0	0
Anterior cingulate area-Superior colliculus	0	0	0	0
Somatomotor areas-Superior colliculus	-0.04048	-0.30192	-0.29252	-0.09531
Somatosensory areas-Superior colliculus	0.613886	-0.32024	-0.0779	-0.02461
piriform area-Superior colliculus	0	0	0	0.92004
Taenia tecta -Superior colliculus	-0.70843	-0.31727	-0.34436	-0.33827
Accessory olfactory bulb glomerular layer-Superior colliculus	0	0	0	0
Accessory olfactory bulb granular layer-Superior colliculus	0	0	0	0

Retrohippocampal region -Superior colliculus	0.260542	-0.19618	0.54934	-0.07332
Entorhinal area-Superior colliculus	-0.03042	-0.00279	0.304288	-0.32438
Field CA1-Superior colliculus	-0.89457	-0.08595	0.750675	-0.19258
Field CA3-Superior colliculus	0.657344	-0.02952	0.407779	-0.09169
Dentate gyrus-Superior colliculus	0.129424	-0.02763	0.032532	-0.80724
Field CA2 -Superior colliculus	0.985827	0	0.414157	-0.4674
Accessory olfactory bulb mitral layer-Superior colliculus	0	0	0	0
Striatum -Superior colliculus	-0.18648	-0.01013	0.585437	-0.01327
Midbrain -Superior colliculus	-0.81662	-0.03602	0.016338	-0.35421
Medulla-Superior colliculus	-0.02282	-0.00046	0.337767	-0.05241
Hypothalamus -ventricular systems	-0.468356	-0.003881	0.135943	-0.00147
Inferior colliculus -ventricular systems	-0.136981	-0.139249	0.75606	-0.038977
periaqueductal gray-ventricular systems	0.903983	-0.059216	-0.456043	-0.001242
Isocortex -ventricular systems	0.056158	0.113367	-0.057076	-0.107899
Cortical amygdalar area -ventricular systems	0.149617	-0.39196	0.66062	0.015249
Olfactory areas -ventricular systems	-0.477356	-0.619148	-0.268137	-0.571139
Pons-ventricular systems	-0.43327	-0.96795	0.703049	0.965527
Midbrain reticular nucleus-ventricular systems	0.657344	-0.029217	0.128671	-0.197117
Nucleus accumbens-ventricular systems	-0.890886	0.896678	-0.631528	0.548261
fimbria-ventricular systems	-0.12663	-0.008097	-0.063006	-0.072439
Anterior cingulate area-ventricular systems	-0.125325	0.286865	-0.006616	-0.797701
Somatomotor areas-ventricular systems	0.468356	0.065683	-0.243067	-0.553997
Somatosensory areas-ventricular systems	0.034769	0.012583	-0.130241	-0.515516
piriform area-ventricular systems	0.119401	0.685585	-0.674362	0.687187
Taenia tecta -ventricular systems	0.02213	0.023846	0.519852	0.860565
Accessory olfactory bulb glomerular layer-ventricular systems	0	0	0	0
Accessory olfactory bulb granular layer-ventricular systems	0	0	0	0
Retrohippocampal region -ventricular systems	-0.203779	-0.047996	-0.044004	-0.004462
Entorhinal area-ventricular systems	-0.194147	-0.020313	-0.043154	-0.354207
Field CA1-ventricular systems	-0.099051	-0.047919	-0.143631	-0.648925
Field CA3-ventricular systems	0.217886	-0.009946	-0.020398	-0.59004
Dentate gyrus-ventricular systems	-0.81369	-0.156689	-0.406199	-0.24518
Field CA2 -ventricular systems	-0.76764	-0.279997	-0.148122	-0.870062
Accessory olfactory bulb mitral layer-ventricular systems	0	0	0	0
Striatum -ventricular systems	-0.018452	-0.343131	-0.101767	-0.048314

Midbrain -ventricular systems	-0.074859	-0.032024	-0.839489	-0.056325
Medulla-ventricular systems	0.136981	-0.295264	0.004044	0.097016
Inferior colliculus -Hypothalamus	-0.06607	-0.021424	-0.662623	-0.333586
periaqueductal gray-Hypothalamus	0.961314	0.477847	-0.14028	-0.091498
Isocortex -Hypothalamus	-0.209443	-0.79032	-0.005402	-0.001425
Cortical amygdalar area -Hypothalamus	0.134268	-0.981985	0	0.016431
Olfactory areas -Hypothalamus	0.008964	0.967216	0.879283	0.393112
Pons-Hypothalamus	-0.038528	-0.023846	-0.188323	-0.012838
Midbrain reticular nucleus-Hypothalamus	0.412516	0.536398	0.774725	0.27853
Nucleus accumbens-Hypothalamus	0.91942	-0.852928	-0.020919	-0.008927
fimbria-Hypothalamus	-0.014795	-0.426524	0	-0.00124
Anterior cingulate area-Hypothalamus	0	0	0	-0.424469
Somatomotor areas-Hypothalamus	-0.870264	-0.910223	-0.345029	-0.965527
Somatosensory areas-Hypothalamus	0.216763	0.032415	-0.013541	-0.031119
piriform area-Hypothalamus	0.220123	0.746789	-0.036547	0.314442
Taenia tecta -Hypothalamus	0.008265	0.060462	0.130241	0.283638
Accessory olfactory bulb glomerular layer-Hypothalamus	0	0	0	0
Accessory olfactory bulb granular layer-Hypothalamus	0	0	0	0
Retrohippocampal region -Hypothalamus	-0.657344	-0.142546	-0.00468	-0.003295
Entorhinal area-Hypothalamus	-0.06607	-0.009162	-0.034471	-0.038273
Field CA1-Hypothalamus	-0.04246	-0.013791	-0.010219	-0.002858
Field CA3-Hypothalamus	-0.407891	-0.756503	-0.01171	-0.039511
Dentate gyrus-Hypothalamus	0.875482	-0.784386	-0.150198	-0.369704
Field CA2 -Hypothalamus	-0.017851	-0.005206	-0.004044	-0.005912
Accessory olfactory bulb mitral layer-Hypothalamus	0	0	0	0
Striatum -Hypothalamus	0.175137	-0.555306	-0.006733	0.463481
Midbrain -Hypothalamus	-0.693148	0.664916	0.579304	0.038519
Medulla-Hypothalamus	-0.014655	-0.000912	-0.114467	-0.021439
periaqueductal gray-Inferior colliculus	0.597136	0.44013	-0.23501	-0.39916
Isocortex -Inferior colliculus	-0.48303	0.184482	-0.04315	-0.33499
Cortical amygdalar area -Inferior colliculus	0	0	0	0
Olfactory areas -Inferior colliculus	0	0	0	0
Pons-Inferior colliculus	-0.01177	-0.00178	-0.04461	-0.00398
Midbrain reticular nucleus-Inferior colliculus	0.263552	0	0	0.762914

Nucleus accumbens-Inferior colliculus	0	0	0	0
fimbria-Inferior colliculus	0	0	0	0
Anterior cingulate area-Inferior colliculus	0	0	0	0
Somatomotor areas-Inferior colliculus	-0.0103	0	0	-0.01852
Somatosensory areas-Inferior colliculus	-0.05708	-0.80518	-0.14381	-0.0049
piriform area-Inferior colliculus	0	0	0	0
Taenia tecta -Inferior colliculus	0	0	0	0
Accessory olfactory bulb glomerular layer-Inferior colliculus	0	0	0	0
Accessory olfactory bulb granular layer-Inferior colliculus	0	0	0	0
Retrohippocampal region -Inferior colliculus	0.662842	0.555306	-0.23624	-0.597
Entorhinal area-Inferior colliculus	-0.02022	0	0	0.601488
Field CA1-Inferior colliculus	-0.18919	0	0	0.929153
Field CA3-Inferior colliculus	-0.33601	0	0	0.589164
Dentate gyrus-Inferior colliculus	-0.42563	0	-0.05318	0.073322
Field CA2 -Inferior colliculus	0	0	0	0
Accessory olfactory bulb mitral layer-Inferior colliculus	0	0	0	0
Striatum -Inferior colliculus	-0.05247	-0.2217	-0.00404	-0.06088
Midbrain -Inferior colliculus	-0.84997	0.162713	-0.43401	-0.37673
Medulla-Inferior colliculus	-0.00744	-0.00046	-0.06995	-0.00249
Isocortex -periaqueductal gray	-0.10682	-0.03453	-0.02506	-0.00348
Cortical amygdalar area -periaqueductal gray	0	0	0	0
Olfactory areas -periaqueductal gray	-0.00028	-0.00447	0	-0.0516
Pons-periaqueductal gray	0.008694	-0.14377	0.004204	0.122746
Midbrain reticular nucleus-periaqueductal gray	0.231324	-0.02836	0.083124	0.261202
Nucleus accumbens-periaqueductal gray	0	0	0	0
fimbria-periaqueductal gray	0	0	0	0
Anterior cingulate area-periaqueductal gray	0	0	0	0
Somatomotor areas-periaqueductal gray	-0.01468	-0.0505	0	-0.00393
Somatosensory areas-periaqueductal gray	-0.40941	-0.0569	-0.02015	-0.00364
piriform area-periaqueductal gray	0	0	0	0
Taenia tecta -periaqueductal gray	-0.07366	0	0	-0.21779
Accessory olfactory bulb glomerular layer-periaqueductal gray	0	0	0	0
Accessory olfactory bulb granular layer-periaqueductal gray	0	0	0	0
Retrohippocampal region -periaqueductal gray	0.889128	-0.02135	-0.0054	-0.02131
Entorhinal area-periaqueductal gray	-0.08338	-0.01287	0	-0.06491

Field CA1-periaqueductal gray	-0.04862	-0.07176	-0.07212	-0.06317
Field CA3-periaqueductal gray	0.081115	-0.61915	0.853724	-0.92004
Dentate gyrus-periaqueductal gray	0.129424	-0.27164	0.691468	-0.62809
Field CA2 -periaqueductal gray	0	0	0	-0.32613
Accessory olfactory bulb mitral layer-periaqueductal gray	0	0	0	0
Striatum -periaqueductal gray	-0.07366	-0.00619	-0.12209	-0.00407
Midbrain -periaqueductal gray	0.661041	-0.08636	0.083124	-0.2645
Medulla-periaqueductal gray	-0.76737	-0.0012	0.052953	-0.14005
Cortical amygdalar area -Isocortex	0.117802	-0.00049	0.181131	0.261202
Olfactory areas -Isocortex	-0.02213	-0.01361	0.585887	-0.04534
Pons-Isocortex	-0.03591	-0.00085	-0.02607	-0.00286
Midbrain reticular nucleus-Isocortex	-0.84975	-0.00829	-0.11447	-0.00143
Nucleus accumbens-Isocortex	-0.51129	-0.09386	-0.91903	-0.06446
fimbria-Ictx	-0.72975	-0.00087	-0.28829	0.136959
Anterior cingulate area-Isocortex	-0.43593	-0.70695	-0.12867	-0.04788
Somatomotor areas-Isocortex	0.291126	0.616639	-0.84471	0.022646
Somatosensory areas-Isocortex	0.04314	-0.63308	-0.46421	0.237381
piriform area-Isocortex	0.014805	-0.13735	0.531303	0.002858
Taenia tecta -Isocortex	-0.89089	-0.00273	0.157724	0.841473
Accessory olfactory bulb glomerular layer-Isocortex	0.74877	0	0	0.745697
Accessory olfactory bulb granular layer-Isocortex	-0.01234	0	0	-0.3636
Retrohippocampal region -Isocortex	0.009317	-0.00513	-0.18113	0.946317
Entorhinal area-Isocortex	-0.49291	-0.00111	-0.35256	0.292135
Field CA1-Isocortex	0.065323	-0.00583	0.323867	0.043107
Field CA3-Isocortex	0.037808	-0.00583	0.91903	0.201484
Dentate gyrus-Isocortex	0.06607	-0.02385	0.043154	0.051956
Field CA2 -Isocortex	0.139425	-0.01872	0.46239	0.114352
Accessory olfactory bulb mitral layer-Isocortex	-0.33601	0	0	0.944443
Striatum -Isocortex	-0.4935	-0.00394	0.725075	-0.03907
Midbrain -Isocortex	-0.09201	-0.00736	-0.06453	-0.00626
Medulla-Isocortex	-0.09995	-0.00041	-0.49004	-0.00906
Olfactory areas -Cortical amygdalar area	-0.80997	0	-0.00336	-0.58489
Pons-Cortical amygdalar area	0.406194	0	0	0
Midbrain reticular nucleus-Cortical amygdalar area	0	0	0	0

Nucleus accumbens-Cortical amygdalar area	-0.10682	0	0	0
fimbria-Cortical amygdalar area	-0.13135	0	0	0
Anterior cingulate area-Cortical amygdalar area	0	0	0	0
Somatomotor areas-Cortical amygdalar area	-0.23132	0	0	0.266347
Somatosensory areas-Cortical amygdalar area	0.099833	0	-0.06521	0.965527
piriform area-Cortical amygdalar area	0.432824	0	0	0.62694
Taenia tecta -Cortical amygdalar area	-0.55889	0.416356	0	-0.32613
Accessory olfactory bulb glomerular layer-Cortical amygdalar area	0	0	0	0
Accessory olfactory bulb granular layer-Cortical amygdalar area	0	0	0	0
Retrohippocampal region -Cortical amygdalar area	0.080848	0	0	-0.98146
Entorhinal area-Cortical amygdalar area	0.040275	0	0	0.001625
Field CA1-Cortical amygdalar area	0.099051	0	0	0.007538
Field CA3-Cortical amygdalar area	0.150446	0	0	0.003295
Dentate gyrus-Cortical amygdalar area	0.135483	0	0	0.013274
Field CA2 -Cortical amygdalar area	-0.55972	0	0	0.015249
Accessory olfactory bulb mitral layer-Cortical amygdalar area	0	0	0	0
Striatum -Cortical amygdalar area	0.667477	0.184482	-0.00626	0.06118
Midbrain -Cortical amygdalar area	0.525857	0	0	0.01834
Medulla-Cortical amygdalar area	0.424574	0	0	0
Pons-Olfactory areas	-0.0013	-0.00949	-0.00979	-0.00789
Midbrain reticular nucleus-Olfactory areas	0	0	0	0
Nucleus accumbens-Olfactory areas	-0.75491	-0.24019	-0.24307	-0.09169
fimbria-Olfactory areas	-0.03772	0	0	-0.03784
Anterior cingulate area-Olfactory areas	0.809734	0.96795	-0.02974	-0.05072
Somatomotor areas-Olfactory areas	0.073661	0.071885	0.728621	0.001815
Somatosensory areas-Olfactory areas	-0.82389	0.225921	-0.23501	-0.27853
piriform area-Olfactory areas	-0.00138	-0.28501	0.579304	-0.1709
Taenia tecta -Olfactory areas	0.849965	-0.64746	0.857952	-0.18288
Accessory olfactory bulb glomerular layer-Olfactory areas	0.514114	0	0	-0.83095
Accessory olfactory bulb granular layer-Olfactory areas	-0.67151	0	0	-0.48178
Retrohippocampal region -Olfactory areas	-0.01686	-0.01612	-0.00336	-0.00803
Entorhinal area-Olfactory areas	-0.00303	-0.00442	-0.08312	-0.29214
Field CA1-Olfactory areas	-0.00896	-0.00704	-0.02506	-0.06829
Field CA3-Olfactory areas	-0.05761	-0.06617	-0.02015	-0.22677
Dentate gyrus-Olfactory areas	-0.12942	-0.74979	-0.0575	-0.35421

Field CA2 -Olfactory areas	-0.01468	-0.17821	-0.14381	-0.28364
Accessory olfactory bulb mitral layer-Olfactory areas	0.377619	0	0	0.929153
Striatum -Olfactory areas	0.608522	0.521222	0.644787	-0.80724
Midbrain -Olfactory areas	-0.00141	-0.00388	-0.00336	-0.07771
Medulla-Olfactory areas	-0.0013	-0.00254	0	-0.05633
Midbrain reticular nucleus-Pons	0.01487	0.115842	0	0.27122
Nucleus accumbens-Pons	0	0	0	0
fimbria-Pons	0	0	0	0
Anterior cingulate area-Pons	0	0	0	0
Somatomotor areas-Pons	-0.01991	-0.02694	0	-0.00121
Somatosensory areas-Pons	-0.04291	-0.15206	-0.0042	-0.00121
piriform area-Pons	0	0	0	0
Taenia tecta -Pons	-0.00589	-0.00273	0	0
Accessory olfactory bulb glomerular layer-Pons	0	0	0	0
Accessory olfactory bulb granular layer-Pons	0	0	0	0
Retrohippocampal region -Pons	0.040475	0	-0.00213	-0.06564
Entorhinal area-Pons	0.850008	-0.02359	-0.08354	-0.29099
Field CA1-Pons	-0.77488	-0.23538	-0.01022	-0.27584
Field CA3-Pons	0.021424	0.320235	-0.24495	0.473919
Dentate gyrus-Pons	0.008265	0.415162	-0.05676	0.107618
Field CA2 -Pons	0	0	0	0
Accessory olfactory bulb mitral layer-Pons	0	0	0	0
Striatum -Pons	-0.00827	-0.00648	-0.00325	-0.00121
Midbrain -Pons	0.558078	0.018611	-0.06453	0.951367
Medulla-Pons	-0.43282	-0.00134	0.345029	-0.6541
Nucleus accumbens-Midbrain reticular nucleus	0	0	0	0
fimbria-Midbrain reticular nucleus	0	0	0	0
Anterior cingulate area-Midbrain reticular nucleus	0	0	0	0
Somatomotor areas-Midbrain reticular nucleus	0	0	0	0
Somatosensory areas-Midbrain reticular nucleus	0.78132	0	0	-0.02373
piriform area-Midbrain reticular nucleus	0	0	0	0
Taenia tecta -Midbrain reticular nucleus	0	0	0	0
Accessory olfactory bulb glomerular layer-Midbrain reticular nucleus	0	0	0	0
Accessory olfactory bulb granular layer-Midbrain reticular nucleus	0	0	0	0

Retrohippocampal region -Midbrain reticular nucleus	0.459217	0	0	-0.00121
Entorhinal area-Midbrain reticular nucleus	0	0	0	0
Field CA1-Midbrain reticular nucleus	0	0	0	-0.11547
Field CA3-Midbrain reticular nucleus	0.206175	0	0	0.095313
Dentate gyrus-Midbrain reticular nucleus	0.052472	0	0	-0.0115
Field CA2 -Midbrain reticular nucleus	0	0	0	0
Accessory olfactory bulb mitral layer-Midbrain reticular nucleus	0	0	0	0
Striatum -Midbrain reticular nucleus	-0.10069	-0.02836	0	-0.06899
Midbrain -Midbrain reticular nucleus	0.671513	0.084635	-0.23501	-0.19083
Medulla-Midbrain reticular nucleus	-0.37407	0	0	-0.01771
fimbria-Nucleus accumbens	-0.76764	0	0	-0.06039
Anterior cingulate area-Nucleus accumbens	0.978246	0	0	0
Somatomotor areas-Nucleus accumbens	0.183123	0	0	-0.35973
Somatosensory areas-Nucleus accumbens	0.664825	0	-0.75068	-0.04508
piriform area-Nucleus accumbens	-0.42743	0	0	0.668436
Taenia tecta -Nucleus accumbens	0.039869	0.479995	0.628989	0.032254
Accessory olfactory bulb glomerular layer-Nucleus accumbens	0	0	0	0
Accessory olfactory bulb granular layer-Nucleus accumbens	0	0	0	0
Retrohippocampal region -Nucleus accumbens	-0.04314	0	0	-0.0049
Entorhinal area-Nucleus accumbens	-0.001	-0.02265	0	-0.00407
Field CA1-Nucleus accumbens	-0.00827	0	0	-0.00407
Field CA3-Nucleus accumbens	-0.65994	0	0	-0.04832
Dentate gyrus-Nucleus accumbens	0.340778	0	0	-0.44558
Field CA2 -Nucleus accumbens	-0.02213	0	0	-0.554
Accessory olfactory bulb mitral layer-Nucleus accumbens	0	0	0	0
Striatum -Nucleus accumbens	0.15623	0.96649	0.8792	0.147959
Midbrain -Nucleus accumbens	-0.15882	0	0	-0.0467
Medulla-Nucleus accumbens	0	0	0	0
Anterior cingulate area-fimbria	0	0	0	0
Somatomotor areas-fimbria	0	0	0	0
Somatosensory areas-fimbria	0.150439	0	0	0.217785
piriform area-fimbria	0	0	0	0
Taenia tecta -fimbria	0.206782	0	0	-0.05199
Accessory olfactory bulb glomerular layer-fimbria	0	0	0	0

Accessory olfactory bulb granular layer-fimbria	0	0	0	0
Retrohippocampal region -fimbria	-0.00305	0	0	-0.01521
Entorhinal area-fimbria	-0.00073	0	0	0
Field CA1-fimbria	-0.04314	0	0	0.57434
Field CA3-fimbria	0.05246	-0.01325	-0.00979	0.470179
Dentate gyrus-fimbria	-0.97819	0	0	0.167187
Field CA2 -fimbria	-0.63562	0	0	-0.08981
Accessory olfactory bulb mitral layer-fimbria	0	0	0	0
Striatum -fimbria	-0.00303	-0.06046	-0.03946	-0.00626
Midbrain -fimbria	-0.00108	0	0	-0.06698
Medulla-fimbria	0	0	0	0
Somatomotor areas-Anterior cingulate area	0.009317	0.146905	0.327367	-0.95137
Somatosensory areas-Anterior cingulate area	-0.56108	-0.18448	0.235373	-0.64893
piriform area-Anterior cingulate area	-0.42563	0	0.124598	0.641581
Taenia tecta -Anterior cingulate area	0.64131	-0.05836	0.629669	-0.15177
Accessory olfactory bulb glomerular layer-Anterior cingulate area	0	0	0	0
Accessory olfactory bulb granular layer-Anterior cingulate area	0	0	0	0
Retrohippocampal region -Anterior cingulate area	0	0	0	-0.05926
Entorhinal area-Anterior cingulate area	-0.00932	-0.00284	0	-0.00789
Field CA1-Anterior cingulate area	0	0	0	0
Field CA3-Anterior cingulate area	0	0	0	-0.7977
Dentate gyrus-Anterior cingulate area	0	0	0	0.801122
Field CA2 -Anterior cingulate area	0.003033	0.935825	0.020154	0.718865
Accessory olfactory bulb mitral layer-Anterior cingulate area	0	0	0	0
Striatum -Anterior cingulate area	-0.585	-0.00062	0.579304	-0.68041
Midbrain -Anterior cingulate area	0	0	-0.00325	-0.00803
Medulla-Anterior cingulate area	0	0	0	0
Somatosensory areas-Somatomotor areas	-0.11705	-0.59738	0.047926	0.046226
piriform area-Somatomotor areas	0.295821	0	0.257442	0.005982
Taenia tecta -Somatomotor areas	0.110251	0.152056	0.029993	0.073322
Accessory olfactory bulb glomerular layer-Somatomotor areas	0.074859	0	0	0.002652
Accessory olfactory bulb granular layer-Somatomotor areas	0	0	0	0
Retrohippocampal region -Somatomotor areas	-0.01785	0	0	-0.01142
Entorhinal area-Somatomotor areas	-0.01468	0	0	-0.0169

Field CA1-Somatomotor areas	-0.16206	0	0	0.366081
Field CA3-Somatomotor areas	0.849745	0	0	0.717403
Dentate gyrus-Somatomotor areas	-0.8389	0	0	-0.82544
Field CA2 -Somatomotor areas	0.056766	0.179799	0.025059	0.642947
Accessory olfactory bulb mitral layer-Somatomotor areas	0.138594	0	0	0
Striatum -Somatomotor areas	0.332881	-0.46145	0.103435	0.070425
Midbrain -Somatomotor areas	-0.00528	-0.01861	-0.04549	-0.00308
Medulla-Somatomotor areas	-0.0013	0	0	-0.02436
piriform area-Somatosensory areas	0.020748	0.819066	0.058017	0.005287
Taenia tecta -Somatosensory areas	0.119401	0.685585	0.077901	0.453313
Accessory olfactory bulb glomerular layer-Somatosensory areas	0	0	0	0
Accessory olfactory bulb granular layer-Somatosensory areas	0	0	0	0
Retrohippocampal region -Somatosensory areas	0.254155	-0.00829	0	-0.0464
Entorhinal area-Somatosensory areas	-0.08312	-0.01112	-0.14501	-0.04775
Field CA1-Somatosensory areas	0.038908	-0.00178	0.212204	0.965527
Field CA3--Somatosensory areas	0.018193	-0.00321	0.530536	-0.96553
Dentate gyrus-Somatosensory areas	0.011756	-0.0376	0.143812	-0.36424
Field CA2 -Somatosensory areas	0.332881	0.104339	0.635868	0.114352
Accessory olfactory bulb mitral layer-Somatosensory areas	0	0	0	0
Striatum --Somatosensory areas	0.27461	-0.06086	0.059375	-0.1858
Midbrain --Somatosensory areas	-0.13277	-0.00178	-0.09644	-0.00717
Medulla-Somatosensory areas	-0.01468	-0.00062	0	-0.00598
Taenia tecta -piriform area	-0.0148	0.41051	0	-0.93863
Accessory olfactory bulb glomerular layer-piriform area	-0.27389	0	0	-0.33594
Accessory olfactory bulb granular layer-piriform area	0	0	0	0
Retrohippocampal region -piriform area	0.8277	0	0	-0.05691
Entorhinal area-piriform area	-0.2258	-0.01339	0	-0.47936
Field CA1-piriform area	0.209438	0	0	0.091697
Field CA3-piriform area	0.020748	0	0	0.047978
Dentate gyrus-piriform area	0.597136	-0.01861	0	0.283638
Field CA2 -piriform area	0.62268	0	0	0.281385
Accessory olfactory bulb mitral layer-piriform area	-0.00349	0	0	-0.94018
Striatum -piriform area	0.716142	0.852928	0.826939	0.166798
Midbrain -piriform area	0	0	0	-0.92915

Medulla-piriform area	0	0	0	0
Accessory olfactory bulb glomerular layer-Taenia tecta	0.113017	0	0	0.120042
Accessory olfactory bulb granular layer-Taenia tecta	0.438132	0	0	0.272584
Retrohippocampal region -Taenia tecta	-0.01481	-0.06279	-0.00404	-0.00591
Entorhinal area-Taenia tecta	-0.00303	-0.00195	-0.05938	-0.01142
Field CA1-Taenia tecta	-0.01176	-0.00388	-0.02931	-0.03852
Field CA3-Taenia tecta	0.678349	-0.49519	-0.08105	-0.30731
Dentate gyrus-Taenia tecta	0.30807	0.307082	-0.58589	0.973637
Field CA2 -Taenia tecta	0.88891	0.981985	-0.78475	-0.7207
Accessory olfactory bulb mitral layer-Taenia tecta	0.178863	0	0	0.157375
Striatum -Taenia tecta	0.025867	0.082566	0.106122	0.159605
Midbrain -Taenia tecta	-0.00863	-0.00178	-0.0054	-0.0623
Medulla-Taenia tecta	-0.00744	0	0	0
Accessory olfactory bulb granular layer-Accessory olfactory bulb glomerular layer	-0.14854	0	0	-0.03925
Retrohippocampal region -Accessory olfactory bulb glomerular layer	0	0	0	0
Entorhinal area-Accessory olfactory bulb glomerular layer	0	0	0	0
Field CA1-Accessory olfactory bulb glomerular layer	0	0	0	0
Field CA3-Accessory olfactory bulb glomerular layer	0	0	0	0
Dentate gyrus-Accessory olfactory bulb glomerular layer	0	0	0	0
Field CA2 -Accessory olfactory bulb glomerular layer	0	0	0	0
Accessory olfactory bulb mitral layer-Accessory olfactory bulb glomerular layer	0.336006	0	0	-0.25294
Striatum -Accessory olfactory bulb glomerular layer	0	0	0	0
Midbrain -Accessory olfactory bulb glomerular layer	0	0	0	0
Medulla-Accessory olfactory bulb glomerular layer	0	0	0	0
Retrohippocampal region -Accessory olfactory bulb granular layer	0	0	0	0
Entorhinal area-Accessory olfactory bulb granular layer	0	0	0	0
Field CA1-Accessory olfactory bulb granular layer	0	0	0	0
Field CA3-Accessory olfactory bulb granular layer	0	0	0	0
Dentate gyrus-Accessory olfactory bulb granular layer	0	0	0	0
Field CA2 -Accessory olfactory bulb granular layer	0	0	0	0
Accessory olfactory bulb mitral layer-Accessory olfactory bulb granular layer	-0.45604	0	0	0.965527
Striatum -Accessory olfactory bulb granular layer	0	0	0	0

Midbrain -Accessory olfactory bulb granular layer	0	0	0	0
Medulla-Accessory olfactory bulb granular layer	0	0	0	0
Entorhinal area-Retrohippocampal region	0.003809	-0.00105	-0.06458	0.648925
Field CA1-Retrohippocampal region	-0.56853	-0.000075	-0.02611	-0.16403
Field CA3-Retrohippocampal region	-0.01205	-0.00853	-0.05002	-0.0034
Dentate gyrus-Retrohippocampal region	-0.87473	-0.003	-0.0255	-0.01825
Field CA2 -Retrohippocampal region	-0.02213	-0.00853	-0.07456	-0.01592
Accessory olfactory bulb mitral layer-Retrohippocampal region	0	0	0	0
Striatum -Retrohippocampal region	-0.01494	-0.00073	-0.00636	-0.0049
Midbrain -Retrohippocampal region	-0.14634	-0.01432	-0.19257	-0.02242
Medulla-Retrohippocampal region	0.047474	-0.000026	0	-0.04761
Field CA1-Entorhinal area	0.724121	-0.00238	-0.00662	0.153477
Field CA3-Entorhinal area	-0.05371	-0.00205	-0.02423	-0.95137
Dentate gyrus-Entorhinal area	0.738852	-0.00246	-0.04315	0.051956
Field CA2 -Entorhinal area	-0.03488	-0.00736	-0.0292	0.940175
Accessory olfactory bulb mitral layer-Entorhinal area	0	0	0	0
Striatum -Entorhinal area	-0.00812	-0.02446	-0.00325	-0.09788
Midbrain -Entorhinal area	-0.00971	-0.22415	-0.00579	-0.36608
Medulla-Entorhinal area	0.40786	-0.00726	0.751331	-0.33827
Field CA3-Field CA1	-0.71614	-0.02123	-0.04315	-0.85899
Dentate gyrus-Field CA1	-0.1178	-0.61759	-0.60556	-0.94632
Field CA2 -Field CA1	0.806922	-0.00213	-0.01354	0.760857
Accessory olfactory bulb mitral layer-Field CA1	0	0	0	0
Striatum -Field CA1	-0.01468	-0.00262	-0.0042	-0.00523
Midbrain -Field CA1	-0.01062	-0.01861	-0.00468	-0.10649
Medulla-Field CA1	-0.35612	-0.02123	0	-0.76854
Dentate gyrus-Field CA3	0.788639	-0.05922	-0.13339	0.860565
Field CA2 -Field CA3	-0.8389	-0.02031	-0.02506	-0.36608
Accessory olfactory bulb mitral layer-Field CA3	0	0	0	0
Striatum -Field CA3	-0.23655	-0.00388	-0.01788	-0.06845
Midbrain -Field CA3	-0.88891	-0.2786	-0.62709	0.420251
Medulla-Field CA3	0.014398	-0.00135	0.109411	0.979117

Field CA2 -Dentate gyrus	-0.34496	-0.05419	-0.0139	0.951367
Accessory olfactory bulb mitral layer-Dentate gyrus	0	0	0	0
Striatum -Dentate gyrus	-0.96814	-0.02264	-0.04315	0.965527
Midbrain -Dentate gyrus	-0.3731	-0.42087	-0.17467	0.870062
Medulla-Dentate gyrus	0.032549	-0.00442	0.045484	0.745697
Accessory olfactory bulb mitral layer-Field CA2	0	0	0	0
Striatum -Field CA2	-0.08619	-0.00844	-0.13024	-0.01213
Midbrain -Field CA2	-0.00249	-0.00232	-0.06216	-0.29214
Medulla-Field CA2	0	0	0	0
Striatum -Accessory olfactory bulb mitral layer	0	0	0	0
Midbrain -Accessory olfactory bulb mitral layer	0	0	0	0
Medulla-Accessory olfactory bulb mitral layer	0	0	0	0
Midbrain -Striatum	-0.00744	-0.00474	-0.01171	-0.00822
Medulla-Striatum	-0.0026	-0.00018	-0.04928	-0.0049
Medulla-Midbrain	-0.09375	-0.00089	0.866937	-0.11471

S6. Adjust p values for group comparisons of pair-wised connectivity for Cerebellum.

Region Connections	Cerebellum			
	Left Ipsilateral	Left Contralateral	Right Contralateral	Right Ipsilateral
Caudoputamen-corpus callosum	0	0	0	0
anterior commissure olfactory limb-corpus callosum	0	0	0	0
pallidum -corpus callosum	0	0	0	0
internal capsule-corpus callosum	0	0	0	0
Thalamus-corpus callosum	0	0	0	0
Cerebellum-corpus callosum	-0.06081	-0.02595	0	0
Superior colliculus-corpus callosum	0	0	-0.03176	-0.01517
ventricular systems-corpus callosum	0	0	0	0
Hypothalamus -corpus callosum	0	0	0	0
Inferior colliculus -corpus callosum	0	0	0	0
periaqueductal gray-corpus callosum	0	0	0	0
Isocortex -corpus callosum	0	0	0	0
Cortical amygdalar area -corpus callosum	0	0	0	0
Olfactory areas -corpus callosum	0	0	0	0
Pons-corpus callosum	0	0	0	0
Midbrain reticular nucleus-corpus callosum	0	0	-0.03793	0
Nucleus accumbens-corpus callosum	0	0	0	0
fimbria-corpus callosum	0	0	0	0
Anterior cingulate area-corpus callosum	0	0	0	0
Somatomotor areas-corpus callosum	0	0	0	0
Somatosensory areas-corpus callosum	0	0	0	0
piriform area-corpus callosum	0	0	0	0
Taenia tecta -corpus callosum	0	0	0	0
Accessory olfactory bulb glomerular layer-corpus callosum	0	0	0	0
Accessory olfactory bulb granular layer-corpus callosum	0	0	0	0
Retrohippocampal region -corpus callosum	0	0	0	0
Entorhinal area-corpus callosum	0	0	0	0
Field CA1-corpus callosum	0	0	0	0
Field CA3-corpus callosum	0	0	0	0
Dentate gyrus-corpus callosum	0	0	0	0
Field CA2 -corpus callosum	0	0	0	0
Accessory olfactory bulb mitral layer-corpus callosum	0	0	0	0

Striatum -corpus callosum	0	0	0	0
Midbrain -corpus callosum	-0.06081	0	0	0
Medulla-corpus callosum	0	0	0	-0.38138
anterior commissure olfactory limb-Caudoputamen	0	0	0	0
pallidum -Caudoputamen	0	0	0	0
internal capsule-Caudoputamen	0	0	0	0
Thalamus-Caudoputamen	0	0	0	0
Cerebellum-Caudoputamen	-0.027145	-0.002473	0	0
Superior colliculus-Caudoputamen	0	0	-0.023894	-0.01538
ventricular systems-Caudoputamen	-0.020005	0	0	0
Hypothalamus -Caudoputamen	0	0	-0.085863	-0.030982
Inferior colliculus -Caudoputamen	0	0	0	0
periaqueductal gray-Caudoputamen	0	0	-0.205666	-0.196883
Isocortex -Caudoputamen	0	0	0	0
Cortical amygdalar area -Caudoputamen	0	0	0	0
Olfactory areas -Caudoputamen	0	0	0	0
Pons-Caudoputamen	-0.132746	0	0	0
Midbrain reticular nucleus-Caudoputamen	0	0	-0.003651	-0.01538
Nucleus accumbens-Caudoputamen	0	0	0	0
fimbria-Caudoputamen	0	0	0	0
Anterior cingulate area-Caudoputamen	0	0	0	0
Somatomotor areas-Caudoputamen	0	0	0	0
Somatosensory areas-Caudoputamen	0	0	0	0
piriform area-Caudoputamen	0	0	0	0
Taenia tecta -Caudoputamen	0	0	0	0
Accessory olfactory bulb glomerular layer-Caudoputamen	0	0	0	0
Accessory olfactory bulb granular layer-Caudoputamen	0	0	0	0
Retrohippocampal region -Caudoputamen	0	0	0	0
Entorhinal area-Caudoputamen	0	0	0	0
Field CA1-Caudoputamen	0	0	0	0
Field CA3-Caudoputamen	0	0	0	0
Dentate gyrus-Caudoputamen	0	0	0	0
Field CA2 -Caudoputamen	0	0	0	0
Accessory olfactory bulb mitral layer-Caudoputamen	0	0	0	0
Striatum -Caudoputamen	0	0	0	0
Midbrain -Caudoputamen	-0.074669	0	0	0

Medulla-Caudoputamen	-0.00236	0	-0.115285	-0.123312
pallidum -anterior commissure olfactory limb	0	0	0	0
internal capsule-anterior commissure olfactory limb	0	0	0	0
Thalamus-anterior commissure olfactory limb	0	0	0	0
Cerebellum-anterior commissure olfactory limb	0	0	0	0
Superior colliculus-anterior commissure olfactory limb	0	0	0	0
ventricular systems-anterior commissure olfactory limb	0	0	0	0
Hypothalamus -anterior commissure olfactory limb	0	0	0	0
Inferior colliculus -anterior commissure olfactory limb	0	0	0	0
periaqueductal gray-anterior commissure olfactory limb	0	0	0	0
Isocortex -anterior commissure olfactory limb	0	0	0	0
Cortical amygdalar area -anterior commissure olfactory limb	0	0	0	0
Olfactory areas -anterior commissure olfactory limb	0	0	0	0
Pons-anterior commissure olfactory limb	0	0	0	0
Midbrain reticular nucleus-anterior commissure olfactory limb	0	0	0	0
Nucleus accumbens-anterior commissure olfactory limb	0	0	0	0
fimbria-anterior commissure olfactory limb	0	0	0	0
Anterior cingulate area-anterior commissure olfactory limb	0	0	0	0
Somatomotor areas-anterior commissure olfactory limb	0	0	0	0
Somatosensory areas-anterior commissure olfactory limb	0	0	0	0
piriform area-anterior commissure olfactory limb	0	0	0	0
Taenia tecta -anterior commissure olfactory limb	0	0	0	0
Accessory olfactory bulb glomerular layer-anterior commissure olfactory limb	0	0	0	0
Accessory olfactory bulb granular layer-anterior commissure olfactory limb	0	0	0	0
Retrohippocampal region -anterior commissure olfactory limb	0	0	0	0
Entorhinal area-anterior commissure olfactory limb	0	0	0	0
Field CA1-anterior commissure olfactory limb	0	0	0	0
Field CA3-anterior commissure olfactory limb	0	0	0	0
Dentate gyrus-anterior commissure olfactory limb	0	0	0	0
Field CA2 -anterior commissure olfactory limb	0	0	0	0
Accessory olfactory bulb mitral layer-anterior commissure olfactory limb	0	0	0	0
Striatum -anterior commissure olfactory limb	0	0	0	0
Midbrain -anterior commissure olfactory limb	0	0	0	0
Medulla-anterior commissure olfactory limb	0	0	0	0

internal capsule-pallidum	0	0	0	0
Thalamus-pallidum	0	0	0	0
Cerebellum-pallidum	-0.188241	-0.025947	0	0
Superior colliculus-pallidum	0	0	-0.031758	-0.01538
ventricular systems-pallidum	0	0	0	0
Hypothalamus -pallidum	0	0	0	0
Inferior colliculus -pallidum	0	0	0	0
periaqueductal gray-pallidum	0	0	0	0
Isocortex -pallidum	0	0	0	0
Cortical amygdalar area -pallidum	0	0	0	0
Olfactory areas -pallidum	0	0	0	0
Pons-pallidum	0	0	0	0
Midbrain reticular nucleus-pallidum	0	0	0	0
Nucleus accumbens-pallidum	0	0	0	0
fimbria-pallidum	0	0	0	0
AVA-pallidum	0	0	0	0
Somatomotor areas-pallidum	0	0	0	0
Somatosensory areas-pallidum	0	0	0	0
piriform area-pallidum	0	0	0	0
Taenia tecta -pallidum	0	0	0	0
Accessory olfactory bulb glomerular layer-pallidum	0	0	0	0
Accessory olfactory bulb granular layer-pallidum	0	0	0	0
Retrohippocampal region -pallidum	0	0	0	0
Entorhinal area-pallidum	0	0	0	0
Field CA1-pallidum	0	0	0	0
Field CA3-pallidum	0	0	0	0
Dentate gyrus-pallidum	0	0	0	0
Field CA2 -pallidum	0	0	0	0
Accessory olfactory bulb mitral layer-pallidum	0	0	0	0
Striatum -pallidum	0	0	0	0
Midbrain -pallidum	0	0	0	0
Medulla-pallidum	0	0	0	0
Thalamus-internal capsule	0	0	0	0
Cerebellum-internal capsule	0.022949	0	0	0
Superior colliculus-internal capsule	0	0	0	-0.826487

ventricular systems-internal capsule	0	0	0	0
Hypothalamus -internal capsule	0	0	0	0
Inferior colliculus -internal capsule	0	0	0	0
periaqueductal gray-internal capsule	0	0	0	0
Isocortex -internal capsule	0	0	0	0
Cortical amygdalar area -internal capsule	0	0	0	0
Olfactory areas -internal capsule	0	0	0	0
Pons-internal capsule	0	0	0	0
Midbrain reticular nucleus-internal capsule	0	0	0	0
Nucleus accumbens-internal capsule	0	0	0	0
fimbria-internal capsule	0	0	0	0
Anterior cingulate area-internal capsule	0	0	0	0
Somatomotor areas-internal capsule	0	0	0	0
Somatosensory areas-internal capsule	0	0	0	0
piriform area-internal capsule	0	0	0	0
Taenia tecta -internal capsule	0	0	0	0
Accessory olfactory bulb glomerular layer-internal capsule	0	0	0	0
Accessory olfactory bulb granular layer-internal capsule	0	0	0	0
Retrohippocampal region -internal capsule	0	0	0	0
Entorhinal area-internal capsule	0	0	0	0
Field CA1-internal capsule	0	0	0	0
Field CA3-internal capsule	0	0	0	0
Dentate gyrus-internal capsule	0	0	0	0
Field CA2 -internal capsule	0	0	0	0
Accessory olfactory bulb mitral layer-internal capsule	0	0	0	0
Striatum -internal capsule	0	0	0	0
Midbrain --internal capsule	0	0	0	0
Medulla-internal capsule	0	0	0	0
Cerebellum-Thalamus	0.001161	0.045741	0	0
Superior colliculus-Thalamus	0	0	-0.8581	-0.9852
ventricular systems-Thalamus	0	0	0	0
Hypothalamus -Thalamus	0	0	-0.66338	0.756914
Inferior colliculus -Thalamus	0	0	0	0
periaqueductal gray-Thalamus	0	0	0	0
Isocortex -Thalamus	0	0	0	0

Cortical amygdalar area -Thalamus	0	0	0	0
Olfactory areas -Thalamus	0	0	0	0
Pons-Thalamus	0	0	0	0
Midbrain reticular nucleus-Thalamus	0	0	-0.77791	0
Nucleus accumbens-Thalamus	0	0	0	0
fimbria-Thalamus	0	0	0	0
Anterior cingulate area-Thalamus	0	0	0	0
Somatomotor areas-Thalamus	0	0	0	0
Somatosensory areas-Thalamus	0	0	0	0
piriform area-Thalamus	0	0	0	0
Taenia tecta -Thalamus	0	0	0	0
Accessory olfactory bulb glomerular layer-Thalamus	0	0	0	0
Accessory olfactory bulb granular layer-Thalamus	0	0	0	0
Retrohippocampal region -Thalamus	0	0	0	0
Entorhinal area-Thalamus	0	0	0	0
Field CA1-Thalamus	0	0	0	0
Field CA3-Thalamus	0	0	0	0
Dentate gyrus-Thalamus	0	0	0	0
Field CA2 -Thalamus	0	0	0	0
Accessory olfactory bulb mitral layer-Thalamus	0	0	0	0
Striatum -Thalamus	0	0	0	0
Midbrain -Thalamus	0	0	0	0
Medulla-Thalamus	-0.29323	-0.22666	0	0
Superior colliculus-Cerebellum	0.000765	0.002664	-0.21326	0
ventricular systems-Cerebellum	0.048973	0.22666	0.042511	0.008902
Hypothalamus -Cerebellum	0.00236	-0.97644	0.434629	0.381375
Inferior colliculus -Cerebellum	0.046733	0.1589	0.009616	0.015168
periaqueductal gray-Cerebellum	0.022949	0.1589	-0.8581	0.182515
Isocortex -Cerebellum	0.010578	0.976549	-0.97235	0.051188
Cortical amygdalar area -Cerebellum	0.007666	0.22666	-0.52198	0.54254
Olfactory areas -Cerebellum	-0.36572	-0.1128	0	-0.66124
Pons-Cerebellum	0.711091	-0.37433	0.615974	0.490793
Midbrain reticular nucleus-Cerebellum	0.00236	0.242913	-0.02975	-0.06932
Nucleus accumbens-Cerebellum	0	0	-0.52198	-0.57382
fimbria-Cerebellum	0	0	0	0

Anterior cingulate area-Cerebellum	0	0	0	0
Somatomotor areas-Cerebellum	-0.09775	-0.17335	0	0
Somatosensory areas-Cerebellum	0.617553	-0.17335	0	-0.01609
piriform area-Cerebellum	0.98021	0	-0.55081	-0.29172
Taenia tecta -Cerebellum	-0.65322	-0.22666	0	0
Accessory olfactory bulb glomerular layer-Cerebellum	0	0	0	-0.02589
Accessory olfactory bulb granular layer-Cerebellum	0	0	0	0
Retrohippocampal region -Cerebellum	0.006958	-0.8449	0	0
Entorhinal area-Cerebellum	0.0044	0.673503	0.75245	0.093043
Field CA1-Cerebellum	0.023544	-0.30134	-0.35611	0.668703
Field CA3-Cerebellum	0.000072	0.1589	-0.93818	0.137662
Dentate gyrus-Cerebellum	0.000096	0.242913	0.696955	0.015168
Field CA2 -Cerebellum	0	-0.01612	-0.79287	-0.71696
Accessory olfactory bulb mitral layer-Cerebellum	0	0	0	-0.638
Striatum -Cerebellum	-0.77345	-0.13379	0	0
Midbrain -Cerebellum	0.000377	0.154802	0.972348	0.476984
Medulla-Cerebellum	0.255801	-0.37433	0.042511	0.015168
ventricular systems-Superior colliculus	0.009355	0	0	0
Hypothalamus -Superior colliculus	0	0	0.006699	0.584998
Inferior colliculus -Superior colliculus	0.010723	0	0	0
periaqueductal gray-Superior colliculus	0	0	0.085863	0
Isocortex -Superior colliculus	0	0	0	0
Cortical amygdalar area -Superior colliculus	0	0	0	0
Olfactory areas -Superior colliculus	0	0	0	0
Pons-Superior colliculus	0.132746	0	0	0
Midbrain reticular nucleus-Superior colliculus	0	0	0.972348	0.565137
Nucleus accumbens-Superior colliculus	0	0	0	0
fimbria-Superior colliculus	0	0	0	0
Anterior cingulate area-Superior colliculus	0	0	0	0
Somatomotor areas-Superior colliculus	0	0	0	0
Somatosensory areas-Superior colliculus	0	0	0	0
piriform area-Superior colliculus	0	0	0	0
Taenia tecta -Superior colliculus	0	0	0	0
Accessory olfactory bulb glomerular layer-Superior colliculus	0	0	0	0
Accessory olfactory bulb granular layer-Superior colliculus	0	0	0	0

Retrohippocampal region -Superior colliculus	0	0	0	0
Entorhinal area-Superior colliculus	0	0	0	0
Field CA1-Superior colliculus	0	0	0	0
Field CA3-Superior colliculus	0	0	0	0
Dentate gyrus-Superior colliculus	0	0	0	0
Field CA2 -Superior colliculus	0	0	0	0
Accessory olfactory bulb mitral layer-Superior colliculus	0	0	0	0
Striatum -Superior colliculus	0	0	0	0
Midbrain -Superior colliculus	0.004339	0	0	0
Medulla-Superior colliculus	-0.25871	0	0.003651	0
Hypothalamus -ventricular systems	-0.974131	-0.724343	-0.320883	0
Inferior colliculus -ventricular systems	-0.960334	0.769579	0.241009	0.381375
periaqueductal gray-ventricular systems	0.103434	0.242913	0.792867	0.137662
Isocortex -ventricular systems	0.28192	-0.761728	0.891321	-0.656162
Cortical amygdalar area -ventricular systems	0	0	0.891321	-0.44922
Olfactory areas -ventricular systems	0	0	0	0
Pons-ventricular systems	0.20812	0.254785	0	0
Midbrain reticular nucleus-ventricular systems	0	0	-0.115285	-0.087389
Nucleus accumbens-ventricular systems	0	0	0	0
fimbria-ventricular systems	0	0	0	0
Anterior cingulate area-ventricular systems	0	0	0	0
Somatomotor areas-ventricular systems	0	0	0	0
Somatosensory areas-ventricular systems	0	0	0	0
piriform area-ventricular systems	0	0	0	0
Taenia tecta -ventricular systems	0	0	0	0
Accessory olfactory bulb glomerular layer-ventricular systems	0	0	0	0
Accessory olfactory bulb granular layer-ventricular systems	0	0	0	0
Retrohippocampal region -ventricular systems	0.008953	0	0	0
Entorhinal area-ventricular systems	0.010578	0	0	0
Field CA1-ventricular systems	0	0	0	-0.758269
Field CA3-ventricular systems	0	0	0	0
Dentate gyrus-ventricular systems	0.000765	0	0	0
Field CA2 -ventricular systems	0	0	0	0.571267
Accessory olfactory bulb mitral layer-ventricular systems	0	0	0	0
Striatum -ventricular systems	0	-0.1646	0	0

Midbrain -ventricular systems	0.210738	-0.596728	0	0.381375
Medulla-ventricular systems	0.382206	-0.622092	0.009616	0.01538
Inferior colliculus -Hypothalamus	0	0	0	0
periaqueductal gray-Hypothalamus	0	0	0	0
Isocortex -Hypothalamus	0	0	0	0
Cortical amygdalar area -Hypothalamus	0	0	0	0
Olfactory areas -Hypothalamus	0	0	0	0
Pons-Hypothalamus	-0.409806	0	0	0
Midbrain reticular nucleus-Hypothalamus	0	0	-0.065172	-0.656162
Nucleus accumbens-Hypothalamus	0	0	0	0
fimbria-Hypothalamus	0	0	0	0
Anterior cingulate area-Hypothalamus	0	0	0	0
Somatomotor areas-Hypothalamus	0	0	0	0
Somatosensory areas-Hypothalamus	0	0	0	0
piriform area-Hypothalamus	0	0	0	0
Taenia tecta -Hypothalamus	0	0	0	0
Accessory olfactory bulb glomerular layer-Hypothalamus	0	0	0	0
Accessory olfactory bulb granular layer-Hypothalamus	0	0	0	0
Retrohippocampal region -Hypothalamus	0	0	0	0
Entorhinal area-Hypothalamus	0	0	0	0
Field CA1-Hypothalamus	0	0	0	0
Field CA3-Hypothalamus	0	0	0	0
Dentate gyrus-Hypothalamus	0	0	0	0
Field CA2 -Hypothalamus	0	0	0	0
Accessory olfactory bulb mitral layer-Hypothalamus	0	0	0	0
Striatum -Hypothalamus	0	0	0	0
Midbrain -Hypothalamus	0.661312	0.062932	0	0
Medulla-Hypothalamus	-0.142502	-0.529216	0	0.661239
periaqueductal gray-Inferior colliculus	0.195155	0.242913	-0.77586	0
Isocortex -Inferior colliculus	0.481732	0.63871	0	0.123312
Cortical amygdalar area -Inferior colliculus	0	0	0	0.381375
Olfactory areas -Inferior colliculus	0	0	0	0
Pons-Inferior colliculus	-0.09983	-0.11755	0	0
Midbrain reticular nucleus-Inferior colliculus	0	0	-0.00962	-0.23291

Nucleus accumbens-Inferior colliculus	0	0	0	0
fimbria-Inferior colliculus	0	0	0	0
Anterior cingulate area-Inferior colliculus	0	0	0	0
Somatomotor areas-Inferior colliculus	0	0	0	0
Somatosensory areas-Inferior colliculus	0	0	0	0
piriform area-Inferior colliculus	0	0	0	0
Taenia tecta -Inferior colliculus	0	0	0	0
Accessory olfactory bulb glomerular layer-Inferior colliculus	0	0	0	0
Accessory olfactory bulb granular layer-Inferior colliculus	0	0	0	0
Retrohippocampal region -Inferior colliculus	0	0	0	0
Entorhinal area-Inferior colliculus	0	0	0	0
Field CA1-Inferior colliculus	0	0	0	0
Field CA3-Inferior colliculus	0	0	0	0
Dentate gyrus-Inferior colliculus	0	0	0	0.476984
Field CA2 -Inferior colliculus	0	0	0	0.912441
Accessory olfactory bulb mitral layer-Inferior colliculus	0	0	0	0
Striatum -Inferior colliculus	0	0	0	0
Midbrain -Inferior colliculus	0.010578	0.117554	0	0
Medulla-Inferior colliculus	-0.01072	-0.0706	-0.52394	-0.48086
Isocortex -periaqueductal gray	-0.98021	0	0	0
Cortical amygdalar area -periaqueductal gray	0	0	0	0
Olfactory areas -periaqueductal gray	0	0	0	0
Pons-periaqueductal gray	0.077956	-0.63871	0	0
Midbrain reticular nucleus-periaqueductal gray	0	0	-0.34146	-0.03975
Nucleus accumbens-periaqueductal gray	0	0	0	0
fimbria-periaqueductal gray	0	0	0	0
Anterior cingulate area-periaqueductal gray	0	0	0	0
Somatomotor areas-periaqueductal gray	0	0	0	0
Somatosensory areas-periaqueductal gray	0	0	0	0
piriform area-periaqueductal gray	0	0	0	0
Taenia tecta -periaqueductal gray	0	0	0	0
Accessory olfactory bulb glomerular layer-periaqueductal gray	0	0	0	0
Accessory olfactory bulb granular layer-periaqueductal gray	0	0	0	0
Retrohippocampal region -periaqueductal gray	0	0	0	0
Entorhinal area-periaqueductal gray	0	0	0	0

Field CA1-periaqueductal gray	0	0	0	0
Field CA3-periaqueductal gray	0	0	0	0
Dentate gyrus-periaqueductal gray	0	0	0	0
Field CA2 -periaqueductal gray	0	0	0	0
Accessory olfactory bulb mitral layer-periaqueductal gray	0	0	0	0
Striatum -periaqueductal gray	0	0	0	0
Midbrain -periaqueductal gray	0.02061	0.110446	0	0
Medulla-periaqueductal gray	-0.09775	-0.0706	0.053372	0.783346
Cortical amygdalar area -Isocortex	0	0	0	0
Olfactory areas -Isocortex	0	0	0	0
Pons-Isocortex	-0.6053	0	0	0
Midbrain reticular nucleus-Isocortex	0	0	-0.02975	-0.38197
Nucleus accumbens-Isocortex	0	0	0	0
fimbria-Ictx	0	0	0	0
Anterior cingulate area-Isocortex	0	0	0	0
Somatomotor areas-Isocortex	0	0	0	0
Somatosensory areas-Isocortex	0	0	0	0
piriform area-Isocortex	0	0	0	0
Taenia tecta -Isocortex	0	0	0	0
Accessory olfactory bulb glomerular layer-Isocortex	0	0	0	0
Accessory olfactory bulb granular layer-Isocortex	0	0	0	0
Retrohippocampal region -Isocortex	0	0	0	0
Entorhinal area-Isocortex	0	0	0	0
Field CA1-Isocortex	0	0	0	0
Field CA3-Isocortex	0	0	0	0
Dentate gyrus-Isocortex	0	0	0	0
Field CA2 -Isocortex	0	0	0	0
Accessory olfactory bulb mitral layer-Isocortex	0	0	0	0
Striatum -Isocortex	0	0	0	0
Midbrain -Isocortex	0.412798	0.63871	0	0
Medulla-Isocortex	-0.73461	0	0.779173	0.716964
Olfactory areas -Cortical amygdalar area	0	0	0	0
Pons-Cortical amygdalar area	0	0	0	0
Midbrain reticular nucleus-Cortical amygdalar area	0	0	0	0

Nucleus accumbens-Cortical amygdalar area	0	0	0	0
fimbria-Cortical amygdalar area	0	0	0	0
Anterior cingulate area-Cortical amygdalar area	0	0	0	0
Somatomotor areas-Cortical amygdalar area	0	0	0	0
Somatosensory areas-Cortical amygdalar area	0	0	0	0
piriform area-Cortical amygdalar area	0	0	0	0
Taenia tecta -Cortical amygdalar area	0	0	0	0
Accessory olfactory bulb glomerular layer-Cortical amygdalar area	0	0	0	0
Accessory olfactory bulb granular layer-Cortical amygdalar area	0	0	0	0
Retrohippocampal region -Cortical amygdalar area	0	0	0	0
Entorhinal area-Cortical amygdalar area	0	0	0	0
Field CA1-Cortical amygdalar area	0	0	0	0
Field CA3-Cortical amygdalar area	0	0	0	0
Dentate gyrus-Cortical amygdalar area	0	0	0	0
Field CA2 -Cortical amygdalar area	0	0	0	0
Accessory olfactory bulb mitral layer-Cortical amygdalar area	0	0	0	0
Striatum -Cortical amygdalar area	0	0	0	0
Midbrain -Cortical amygdalar area	0	0	0	0
Medulla-Cortical amygdalar area	0	0	0	0
Pons-Olfactory areas	0	0	0	0
Midbrain reticular nucleus-Olfactory areas	0	0	0	0
Nucleus accumbens-Olfactory areas	0	0	0	0
fimbria-Olfactory areas	0	0	0	0
Anterior cingulate area-Olfactory areas	0	0	0	0
Somatomotor areas-Olfactory areas	0	0	0	0
Somatosensory areas-Olfactory areas	0	0	0	0
piriform area-Olfactory areas	0	0	0	0
Taenia tecta -Olfactory areas	0	0	0	0
Accessory olfactory bulb glomerular layer-Olfactory areas	0	0	0	0
Accessory olfactory bulb granular layer-Olfactory areas	0	0	0	0
Retrohippocampal region -Olfactory areas	0	0	0	0
Entorhinal area-Olfactory areas	0	0	0	0
Field CA1-Olfactory areas	0	0	0	0
Field CA3-Olfactory areas	0	0	0	0
Dentate gyrus-Olfactory areas	0	0	0	0

Field CA2 -Olfactory areas	0	0	0	0
Accessory olfactory bulb mitral layer-Olfactory areas	0	0	0	0
Striatum -Olfactory areas	0	0	0	0
Midbrain -Olfactory areas	0	0	0	0
Medulla-Olfactory areas	0	0	0	0
Midbrain reticular nucleus-Pons	0	0	-0.22775	0
Nucleus accumbens-Pons	0	0	0	0
fimbria-Pons	0	0	0	0
Anterior cingulate area-Pons	0	0	0	0
Somatomotor areas-Pons	0	0	0	0
Somatosensory areas-Pons	0	0	0	0
piriform area-Pons	0	0	0	0
Taenia tecta -Pons	0	0	0	0
Accessory olfactory bulb glomerular layer-Pons	0	0	0	0
Accessory olfactory bulb granular layer-Pons	0	0	0	0
Retrohippocampal region -Pons	0.010578	0	0	0
Entorhinal area-Pons	0.000765	0	0	0
Field CA1-Pons	0.065283	0	0	-0.38138
Field CA3-Pons	0	0	0	0
Dentate gyrus-Pons	0.004339	0.769579	0	0
Field CA2 -Pons	0	0	0	0.417547
Accessory olfactory bulb mitral layer-Pons	0	0	0	0
Striatum -Pons	-0.52217	-0.13379	0	0
Midbrain -Pons	-0.74402	-0.21251	0.779173	0.769863
Medulla-Pons	-0.32027	-0.02595	-0.79287	-0.47698
Nucleus accumbens-Midbrain reticular nucleus	0	0	0	0
fimbria-Midbrain reticular nucleus	0	0	0	0
Anterior cingulate area-Midbrain reticular nucleus	0	0	0	0
Somatomotor areas-Midbrain reticular nucleus	0	0	0	0
Somatosensory areas-Midbrain reticular nucleus	0	0	0	0
piriform area-Midbrain reticular nucleus	0	0	0	0
Taenia tecta -Midbrain reticular nucleus	0	0	0	0
Accessory olfactory bulb glomerular layer-Midbrain reticular nucleus	0	0	0	0
Accessory olfactory bulb granular layer-Midbrain reticular nucleus	0	0	0	0

Retrohippocampal region -Midbrain reticular nucleus	0	0	0	0
Entorhinal area-Midbrain reticular nucleus	0	0	0	0
Field CA1-Midbrain reticular nucleus	0	0	0	0
Field CA3-Midbrain reticular nucleus	0	0	0	0
Dentate gyrus-Midbrain reticular nucleus	0	0	0	0
Field CA2 -Midbrain reticular nucleus	0	0	0	0
Accessory olfactory bulb mitral layer-Midbrain reticular nucleus	0	0	0	0
Striatum -Midbrain reticular nucleus	0	0	0	0
Midbrain -Midbrain reticular nucleus	0	0	0	0
Medulla-Midbrain reticular nucleus	0	0	0	0
fimbria-Nucleus accumbens	0	0	0	0
Anterior cingulate area-Nucleus accumbens	0	0	0	0
Somatomotor areas-Nucleus accumbens	0	0	0	0
Somatosensory areas-Nucleus accumbens	0	0	0	0
piriform area-Nucleus accumbens	0	0	0	0
Taenia tecta -Nucleus accumbens	0	0	0	0
Accessory olfactory bulb glomerular layer-Nucleus accumbens	0	0	0	0
Accessory olfactory bulb granular layer-Nucleus accumbens	0	0	0	0
Retrohippocampal region -Nucleus accumbens	0	0	0	0
Entorhinal area-Nucleus accumbens	0	0	0	0
Field CA1-Nucleus accumbens	0	0	0	0
Field CA3-Nucleus accumbens	0	0	0	0
Dentate gyrus-Nucleus accumbens	0	0	0	0
Field CA2 -Nucleus accumbens	0	0	0	0
Accessory olfactory bulb mitral layer-Nucleus accumbens	0	0	0	0
Striatum -Nucleus accumbens	0	0	0	0
Midbrain -Nucleus accumbens	0	0	0	0
Medulla-Nucleus accumbens	0	0	0	0
Anterior cingulate area-fimbria	0	0	0	0
Somatomotor areas-fimbria	0	0	0	0
Somatosensory areas-fimbria	0	0	0	0
piriform area-fimbria	0	0	0	0
Taenia tecta -fimbria	0	0	0	0
Accessory olfactory bulb glomerular layer-fimbria	0	0	0	0

Accessory olfactory bulb granular layer-fimbria	0	0	0	0
Retrohippocampal region -fimbria	0	0	0	0
Entorhinal area-fimbria	0	0	0	0
Field CA1-fimbria	0	0	0	0
Field CA3-fimbria	0	0	0	0
Dentate gyrus-fimbria	0	0	0	0
Field CA2 -fimbria	0	0	0	0
Accessory olfactory bulb mitral layer-fimbria	0	0	0	0
Striatum -fimbria	0	0	0	0
Midbrain -fimbria	0	0	0	0
Medulla-fimbria	0	0	0	0
Somatomotor areas-Anterior cingulate area	0	0	0	0
Somatosensory areas-Anterior cingulate area	0	0	0	0
piriform area-Anterior cingulate area	0	0	0	0
Taenia tecta -Anterior cingulate area	0	0	0	0
Accessory olfactory bulb glomerular layer-Anterior cingulate area	0	0	0	0
Accessory olfactory bulb granular layer-Anterior cingulate area	0	0	0	0
Retrohippocampal region -Anterior cingulate area	0	0	0	0
Entorhinal area-Anterior cingulate area	0	0	0	0
Field CA1-Anterior cingulate area	0	0	0	0
Field CA3-Anterior cingulate area	0	0	0	0
Dentate gyrus-Anterior cingulate area	0	0	0	0
Field CA2 -Anterior cingulate area	0	0	0	0
Accessory olfactory bulb mitral layer-Anterior cingulate area	0	0	0	0
Striatum -Anterior cingulate area	0	0	0	0
Midbrain -Anterior cingulate area	0	0	0	0
Medulla-Anterior cingulate area	0	0	0	0
Somatosensory areas-Somatomotor areas	0	0	0	0
piriform area-Somatomotor areas	0	0	0	0
Taenia tecta -Somatomotor areas	0	0	0	0
Accessory olfactory bulb glomerular layer-Somatomotor areas	0	0	0	0
Accessory olfactory bulb granular layer-Somatomotor areas	0	0	0	0
Retrohippocampal region -Somatomotor areas	0	0	0	0
Entorhinal area-Somatomotor areas	0	0	0	0

Field CA1-Somatomotor areas	0	0	0	0
Field CA3-Somatomotor areas	0	0	0	0
Dentate gyrus-Somatomotor areas	0	0	0	0
Field CA2 -Somatomotor areas	0	0	0	0
Accessory olfactory bulb mitral layer-Somatomotor areas	0	0	0	0
Striatum -Somatomotor areas	0	0	0	0
Midbrain -Somatomotor areas	0	0	0	0
Medulla-Somatomotor areas	0	0	0	0
piriform area-Somatosensory areas	0	0	0	0
Taenia tecta -Somatosensory areas	0	0	0	0
Accessory olfactory bulb glomerular layer-Somatosensory areas	0	0	0	0
Accessory olfactory bulb granular layer-Somatosensory areas	0	0	0	0
Retrohippocampal region -Somatosensory areas	0	0	0	0
Entorhinal area-Somatosensory areas	0	0	0	0
Field CA1-Somatosensory areas	0	0	0	0
Field CA3--Somatosensory areas	0	0	0	0
Dentate gyrus-Somatosensory areas	0	0	0	0
Field CA2 -Somatosensory areas	0	0	0	0
Accessory olfactory bulb mitral layer-Somatosensory areas	0	0	0	0
Striatum --Somatosensory areas	0	0	0	0
Midbrain --Somatosensory areas	0	0	0	0
Medulla-Somatosensory areas	0	0	0	0
Taenia tecta -piriform area	0	0	0	0
Accessory olfactory bulb glomerular layer-piriform area	0	0	0	0
Accessory olfactory bulb granular layer-piriform area	0	0	0	0
Retrohippocampal region -piriform area	0	0	0	0
Entorhinal area-piriform area	0	0	0	0
Field CA1-piriform area	0	0	0	0
Field CA3-piriform area	0	0	0	0
Dentate gyrus-piriform area	0	0	0	0
Field CA2 -piriform area	0	0	0	0
Accessory olfactory bulb mitral layer-piriform area	0	0	0	0
Striatum -piriform area	0	0	0	0
Midbrain -piriform area	0	0	0	0

Medulla-piriform area	0	0	0	0
Accessory olfactory bulb glomerular layer-Taenia tecta	0	0	0	0
Accessory olfactory bulb granular layer-Taenia tecta	0	0	0	0
Retrohippocampal region -Taenia tecta	0	0	0	0
Entorhinal area-Taenia tecta	0	0	0	0
Field CA1-Taenia tecta	0	0	0	0
Field CA3-Taenia tecta	0	0	0	0
Dentate gyrus-Taenia tecta	0	0	0	0
Field CA2 -Taenia tecta	0	0	0	0
Accessory olfactory bulb mitral layer-Taenia tecta	0	0	0	0
Striatum -Taenia tecta	0	0	0	0
Midbrain -Taenia tecta	0	0	0	0
Medulla-Taenia tecta	0	0	0	0
Accessory olfactory bulb granular layer-Accessory olfactory bulb glomerular layer	0	0	0	0
Retrohippocampal region -Accessory olfactory bulb glomerular layer	0	0	0	0
Entorhinal area-Accessory olfactory bulb glomerular layer	0	0	0	0
Field CA1-Accessory olfactory bulb glomerular layer	0	0	0	0
Field CA3-Accessory olfactory bulb glomerular layer	0	0	0	0
Dentate gyrus-Accessory olfactory bulb glomerular layer	0	0	0	0
Field CA2 -Accessory olfactory bulb glomerular layer	0	0	0	0
Accessory olfactory bulb mitral layer-Accessory olfactory bulb glomerular layer	0	0	0	0
Striatum -Accessory olfactory bulb glomerular layer	0	0	0	0
Midbrain -Accessory olfactory bulb glomerular layer	0	0	0	0
Medulla-Accessory olfactory bulb glomerular layer	0	0	0	0
Retrohippocampal region -Accessory olfactory bulb granular layer	0	0	0	0
Entorhinal area-Accessory olfactory bulb granular layer	0	0	0	0
Field CA1-Accessory olfactory bulb granular layer	0	0	0	0
Field CA3-Accessory olfactory bulb granular layer	0	0	0	0
Dentate gyrus-Accessory olfactory bulb granular layer	0	0	0	0
Field CA2 -Accessory olfactory bulb granular layer	0	0	0	0
Accessory olfactory bulb mitral layer-Accessory olfactory bulb granular layer	0	0	0	0
Striatum -Accessory olfactory bulb granular layer	0	0	0	0

Midbrain -Accessory olfactory bulb granular layer	0	0	0	0
Medulla-Accessory olfactory bulb granular layer	0	0	0	0
Entorhinal area-Retrohippocampal region	0	0	0	0
Field CA1-Retrohippocampal region	0	0	0	0
Field CA3-Retrohippocampal region	0	0	0	0
Dentate gyrus-Retrohippocampal region	0	0	0	0
Field CA2 -Retrohippocampal region	0	0	0	0
Accessory olfactory bulb mitral layer-Retrohippocampal region	0	0	0	0
Striatum -Retrohippocampal region	0	0	0	0
Midbrain -Retrohippocampal region	0	0	0	0
Medulla-Retrohippocampal region	0.063743	0	0	0
Field CA1-Entorhinal area	0	0	0	0
Field CA3-Entorhinal area	0	0	0	0
Dentate gyrus-Entorhinal area	0	0	0	0
Field CA2 -Entorhinal area	0	0	0	0
Accessory olfactory bulb mitral layer-Entorhinal area	0	0	0	0
Striatum -Entorhinal area	0	0	0	0
Midbrain -Entorhinal area	0	0	0	0
Medulla-Entorhinal area	0.074669	0	0	0
Field CA3-Field CA1	0	0	0	0
Dentate gyrus-Field CA1	0	0	0	0
Field CA2 -Field CA1	0	0	0	0
Accessory olfactory bulb mitral layer-Field CA1	0	0	0	0
Striatum -Field CA1	0	0	0	0
Midbrain -Field CA1	0	0	0	0
Medulla-Field CA1	0	0	0	0
Dentate gyrus-Field CA3	0	0	0	0
Field CA2 -Field CA3	0	0	0	0
Accessory olfactory bulb mitral layer-Field CA3	0	0	0	0
Striatum -Field CA3	0	0	0	0
Midbrain -Field CA3	0	0	0	0
Medulla-Field CA3	0	0	0	0.573824

Field CA2 -Dentate gyrus	0	0	0	0
Accessory olfactory bulb mitral layer-Dentate gyrus	0	0	0	0
Striatum -Dentate gyrus	0	0	0	0
Midbrain -Dentate gyrus	0.013274	0	0	0
Medulla-Dentate gyrus	0.064825	0	0	0.441032
Accessory olfactory bulb mitral layer-Field CA2	0	0	0	0
Striatum -Field CA2	0	0	0	0
Midbrain -Field CA2	0	0	0	0
Medulla-Field CA2	0	0	0	0
Striatum -Accessory olfactory bulb mitral layer	0	0	0	0
Midbrain -Accessory olfactory bulb mitral layer	0	0	0	0
Medulla-Accessory olfactory bulb mitral layer	0	0	0	0
Midbrain -Striatum	0	0	0	0
Medulla-Striatum	-0.21068	0	0	0
Medulla-Midbrain	-0.0179	-0.04639	0.972348	0

S7. Adjust p values for group comparisons of pair-wised connectivity for Superior Colliculus.

Region Connections	Superior Colliculus			
	Left Ipsilateral	Left Contralateral	Right Contralateral	Right Ipsilateral
Caudoputamen-corpus callosum	-0.00258	-0.10327	-0.01461	-0.08308
anterior commissure olfactory limb-corpus callosum	0	0	0	0
pallidum -corpus callosum	-0.06774	0	-0.29146	0
internal capsule-corpus callosum	0	0	0	0
Thalamus-corpus callosum	-0.01059	-0.18617	-0.04114	-0.06749
Cerebellum-corpus callosum	-0.45741	-0.92738	-0.75131	0.646539
Superior colliculus-corpus callosum	-0.02234	-0.19339	-0.0643	-0.0229
ventricular systems-corpus callosum	-0.13706	-0.20135	-0.04114	-0.08668
Hypothalamus -corpus callosum	-0.02234	-0.51493	-0.18357	-0.11769
Inferior colliculus -corpus callosum	-0.01059	-0.30051	-0.26682	-0.04293
periaqueductal gray-corpus callosum	-0.00318	-0.07638	-0.01461	-0.015
Isocortex -corpus callosum	-0.03264	-0.19339	-0.04979	-0.07154
Cortical amygdalar area -corpus callosum	0	0	0	0
Olfactory areas -corpus callosum	0	0	0	0
Pons-corpus callosum	-0.25416	0	-0.06649	-0.11614
Midbrain reticular nucleus-corpus callosum	-0.00258	-0.19339	0	-0.05632
Nucleus accumbens-corpus callosum	0	0	0	0
fimbria-corpus callosum	0	0	0	0
Anterior cingulate area-corpus callosum	0	0	0	0
Somatomotor areas-corpus callosum	0	0	0	0
Somatosensory areas-corpus callosum	-0.03991	0	0	0
piriform area-corpus callosum	0	0	0	0
Taenia tecta -corpus callosum	0	0	0	0
Accessory olfactory bulb glomerular layer-corpus callosum	0	0	0	0
Accessory olfactory bulb granular layer-corpus callosum	0	0	0	0
Retrohippocampal region -corpus callosum	-0.02336	-0.27306	0	-0.42557
Entorhinal area-corpus callosum	0	0	0	0
Field CA1-corpus callosum	-0.23784	0	-0.56359	0
Field CA3-corpus callosum	-0.12006	0	0	0
Dentate gyrus-corpus callosum	-0.03264	-0.30051	-0.68108	0
Field CA2 -corpus callosum	0	0	0	0
Accessory olfactory bulb mitral layer-corpus callosum	0	0	0	0

Striatum -corpus callosum	-0.00258	-0.30255	-0.08065	-0.1307
Midbrain -corpus callosum	-0.00258	-0.03666	-0.04114	-0.015
Medulla-corpus callosum	-0.02144	0	-0.18595	-0.15565
anterior commissure olfactory limb-Caudoputamen	0	0	0	0
pallidum -Caudoputamen	-0.06825	0	-0.237782	0
internal capsule-Caudoputamen	0	0	0	0
Thalamus-Caudoputamen	-0.033548	-0.063364	-0.117324	-0.014998
Cerebellum-Caudoputamen	-0.266354	-0.239526	0.931668	0.540995
Superior colliculus-Caudoputamen	-0.074251	-0.076383	-0.095329	-0.032413
ventricular systems-Caudoputamen	-0.182232	-0.063364	-0.200453	-0.015347
Hypothalamus -Caudoputamen	-0.049979	-0.141612	-0.224292	-0.014998
Inferior colliculus -Caudoputamen	-0.00593	-0.057414	-0.291461	-0.056316
periaqueductal gray-Caudoputamen	-0.011497	-0.0146	-0.014609	-0.014791
Isocortex -Caudoputamen	-0.056052	-0.063364	-0.064297	-0.037167
Cortical amygdalar area -Caudoputamen	0	0	0	0
Olfactory areas -Caudoputamen	-0.112881	0	-0.291461	0
Pons-Caudoputamen	-0.069772	-0.300514	-0.066487	-0.021955
Midbrain reticular nucleus-Caudoputamen	-0.003183	-0.063364	0	-0.062923
Nucleus accumbens-Caudoputamen	0	0	0	0
fimbria-Caudoputamen	0	0	0	0
Anterior cingulate area-Caudoputamen	0	0	0	0
Somatomotor areas-Caudoputamen	-0.166282	0	0	0
Somatosensory areas-Caudoputamen	-0.02417	-0.19339	-0.291461	0
piriform area-Caudoputamen	0	0	0	0
Taenia tecta -Caudoputamen	0	0	0	0
Accessory olfactory bulb glomerular layer-Caudoputamen	0	0	0	0
Accessory olfactory bulb granular layer-Caudoputamen	0	0	0	0
Retrohippocampal region -Caudoputamen	-0.014177	-0.260263	-0.291461	-0.075497
Entorhinal area-Caudoputamen	-0.193063	0	-0.572821	0
Field CA1-Caudoputamen	-0.361054	0	-0.29927	-0.072997
Field CA3-Caudoputamen	-0.043783	0	-0.200453	-0.099133
Dentate gyrus-Caudoputamen	-0.01066	-0.19339	-0.394188	-0.114074
Field CA2 -Caudoputamen	0	0	0	0
Accessory olfactory bulb mitral layer-Caudoputamen	0	0	0	0
Striatum -Caudoputamen	-0.003183	-0.083389	-0.049785	-0.037427
Midbrain -Caudoputamen	-0.003183	-0.012636	-0.066487	-0.020125

Medulla-Caudoputamen	-0.114914	-0.178949	-0.291461	-0.119232
pallidum -anterior commissure olfactory limb	0	0	0	0
internal capsule-anterior commissure olfactory limb	0	0	0	0
Thalamus-anterior commissure olfactory limb	0	0	0	0
Cerebellum-anterior commissure olfactory limb	0	0	0	0
Superior colliculus-anterior commissure olfactory limb	0	0	0	0
ventricular systems-anterior commissure olfactory limb	0	0	0	0
Hypothalamus -anterior commissure olfactory limb	0	0	0	0
Inferior colliculus -anterior commissure olfactory limb	0	0	0	0
periaqueductal gray-anterior commissure olfactory limb	0	0	0	0
Isocortex -anterior commissure olfactory limb	0	0	0	0
Cortical amygdalar area -anterior commissure olfactory limb	0	0	0	0
Olfactory areas -anterior commissure olfactory limb	0	0	0	0
Pons-anterior commissure olfactory limb	0	0	0	0
Midbrain reticular nucleus-anterior commissure olfactory limb	0	0	0	0
Nucleus accumbens-anterior commissure olfactory limb	0	0	0	0
fimbria-anterior commissure olfactory limb	0	0	0	0
Anterior cingulate area-anterior commissure olfactory limb	0	0	0	0
Somatomotor areas-anterior commissure olfactory limb	0	0	0	0
Somatosensory areas-anterior commissure olfactory limb	0	0	0	0
piriform area-anterior commissure olfactory limb	0	0	0	0
Taenia tecta -anterior commissure olfactory limb	0	0	0	0
Accessory olfactory bulb glomerular layer-anterior commissure olfactory limb	0	0	0	0
Accessory olfactory bulb granular layer-anterior commissure olfactory limb	0	0	0	0
Retrohippocampal region -anterior commissure olfactory limb	0	0	0	0
Entorhinal area-anterior commissure olfactory limb	0	0	0	0
Field CA1-anterior commissure olfactory limb	0	0	0	0
Field CA3-anterior commissure olfactory limb	0	0	0	0
Dentate gyrus-anterior commissure olfactory limb	0	0	0	0
Field CA2 -anterior commissure olfactory limb	0	0	0	0
Accessory olfactory bulb mitral layer-anterior commissure olfactory limb	0	0	0	0
Striatum -anterior commissure olfactory limb	0	0	0	0
Midbrain -anterior commissure olfactory limb	0	0	0	0
Medulla-anterior commissure olfactory limb	0	0	0	0

internal capsule-pallidum	0	0	0	0
Thalamus-pallidum	-0.125608	-0.178949	-0.346607	-0.047419
Cerebellum-pallidum	0	0	-0.819498	0
Superior colliculus-pallidum	-0.020193	-0.54014	-0.185948	-0.022896
ventricular systems-pallidum	-0.094279	-0.423883	0	-0.075497
Hypothalamus -pallidum	0	0	0	0
Inferior colliculus -pallidum	-0.032644	-0.19339	-0.427763	-0.078112
periaqueductal gray-pallidum	-0.085645	-0.19339	-0.076266	-0.015347
Isocortex -pallidum	-0.11568	-0.168771	-0.368352	-0.071535
Cortical amygdalar area -pallidum	0	0	0	0
Olfactory areas -pallidum	0	0	0	0
Pons-pallidum	0	0	0	0
Midbrain reticular nucleus-pallidum	-0.203646	0	0	0
Nucleus accumbens-pallidum	0	0	0	0
fimbria-pallidum	0	0	0	0
AVA-pallidum	0	0	0	0
Somatomotor areas-pallidum	0	0	0	0
Somatosensory areas-pallidum	0	0	0	0
piriform area-pallidum	0	0	0	0
Taenia tecta -pallidum	0	0	0	0
Accessory olfactory bulb glomerular layer-pallidum	0	0	0	0
Accessory olfactory bulb granular layer-pallidum	0	0	0	0
Retrohippocampal region -pallidum	0	0	0	0
Entorhinal area-pallidum	0	0	0	0
Field CA1-pallidum	0	0	0	0
Field CA3-pallidum	0	0	0	0
Dentate gyrus-pallidum	-0.351799	0	0	0
Field CA2 -pallidum	0	0	0	0
Accessory olfactory bulb mitral layer-pallidum	0	0	0	0
Striatum -pallidum	-0.156823	0	0	0
Midbrain -pallidum	-0.007945	-0.037863	-0.27814	-0.015347
Medulla-pallidum	0	0	0	-0.610656
Thalamus-internal capsule	0	0	0	0
Cerebellum-internal capsule	0	0	0	0
Superior colliculus-internal capsule	-0.161168	0.284031	-0.589374	-0.06328

ventricular systems-internal capsule	0	0	0	0
Hypothalamus -internal capsule	0	0	0	0
Inferior colliculus -internal capsule	-0.02336	0	0	0
periaqueductal gray-internal capsule	-0.155878	0	0	0
Isocortex -internal capsule	-0.547985	0	0	0
Cortical amygdalar area -internal capsule	0	0	0	0
Olfactory areas -internal capsule	0	0	0	0
Pons-internal capsule	0	0	0	0
Midbrain reticular nucleus-internal capsule	0	0	0	0
Nucleus accumbens-internal capsule	0	0	0	0
fimbria-internal capsule	0	0	0	0
Anterior cingulate area-internal capsule	0	0	0	0
Somatomotor areas-internal capsule	0	0	0	0
Somatosensory areas-internal capsule	0	0	0	0
piriform area-internal capsule	0	0	0	0
Taenia tecta -internal capsule	0	0	0	0
Accessory olfactory bulb glomerular layer-internal capsule	0	0	0	0
Accessory olfactory bulb granular layer-internal capsule	0	0	0	0
Retrohippocampal region -internal capsule	0	0	0	0
Entorhinal area-internal capsule	0	0	0	0
Field CA1-internal capsule	0	0	0	0
Field CA3-internal capsule	0	0	0	0
Dentate gyrus-internal capsule	0	0	0	0
Field CA2 -internal capsule	0	0	0	0
Accessory olfactory bulb mitral layer-internal capsule	0	0	0	0
Striatum -internal capsule	0	0	0	0
Midbrain --internal capsule	-0.00593	0.961825	-0.751309	-0.068438
Medulla-internal capsule	0	0	0	0
Cerebellum-Thalamus	0.033059	0.254748	0.128959	0.022896
Superior colliculus-Thalamus	0.399652	0.076383	0.931668	-0.50728
ventricular systems-Thalamus	0.221505	0.316794	0.758832	-0.78993
Hypothalamus -Thalamus	0.661328	0.240735	0.066487	0.881295
Inferior colliculus -Thalamus	-0.0669	-0.33172	0.807735	0.959097
periaqueductal gray-Thalamus	-0.58579	-0.91247	-0.23778	-0.00399
Isocortex -Thalamus	0.571044	-0.83798	-0.7802	-0.96379

Cortical amygdalar area -Thalamus	0	0	0	0
Olfactory areas -Thalamus	-0.69952	0	-0.29146	-0.03743
Pons-Thalamus	-0.09868	-0.46985	-0.31132	-0.16758
Midbrain reticular nucleus-Thalamus	-0.18737	-0.19339	0.513799	-0.0458
Nucleus accumbens-Thalamus	0	0	0	0
fimbria-Thalamus	0	0	0	0
Anterior cingulate area-Thalamus	0	0	0	0
Somatomotor areas-Thalamus	-0.18841	0	0	-0.07811
Somatosensory areas-Thalamus	0.843811	-0.458	-0.82227	-0.04341
piriform area-Thalamus	0	0	0	0
Taenia tecta -Thalamus	0	0	0	-0.08255
Accessory olfactory bulb glomerular layer-Thalamus	0	0	0	0
Accessory olfactory bulb granular layer-Thalamus	0	0	0	0
Retrohippocampal region -Thalamus	-0.69647	-0.71385	0.904004	0.722201
Entorhinal area-Thalamus	0	-0.87621	-0.47419	-0.52399
Field CA1-Thalamus	0.751773	0.449972	-0.52183	-0.51274
Field CA3-Thalamus	0.814509	0.58865	-0.57282	-0.51274
Dentate gyrus-Thalamus	-0.36393	0.423883	0.292747	-0.57208
Field CA2 -Thalamus	0	0	0	0
Accessory olfactory bulb mitral layer-Thalamus	0	0	0	0
Striatum -Thalamus	-0.25416	-0.19339	-0.45815	-0.16151
Midbrain -Thalamus	-0.58523	-0.42388	0.822274	-0.57611
Medulla-Thalamus	-0.01231	-0.17895	-0.46339	-0.21734
Superior colliculus-Cerebellum	0.010587	0.016222	0.559371	0.014998
ventricular systems-Cerebellum	0.06825	0.254748	0.419698	0.564949
Hypothalamus -Cerebellum	0.043783	0.168771	0.045648	0.022896
Inferior colliculus -Cerebellum	0.021625	0.355524	0.169568	0.014998
periaqueductal gray-Cerebellum	0.03014	0.316794	0.185948	0.04367
Isocortex -Cerebellum	0.040994	0.144877	-0.95486	0.123827
Cortical amygdalar area -Cerebellum	0	0	0	0
Olfactory areas -Cerebellum	0	0	0	-0.8813
Pons-Cerebellum	0.696473	0.331723	0.939767	0.999576
Midbrain reticular nucleus-Cerebellum	0.310723	0.300514	0	0
Nucleus accumbens-Cerebellum	0	0	0	0
fimbria-Cerebellum	0	0	0	0

Anterior cingulate area-Cerebellum	0	0	0	0
Somatomotor areas-Cerebellum	0	-0.55143	0	0
Somatosensory areas-Cerebellum	0.78533	0.821207	0.822274	0.963788
piriform area-Cerebellum	0	0	0	0
Taenia tecta -Cerebellum	0	0	0	0
Accessory olfactory bulb glomerular layer-Cerebellum	0	0	0	0
Accessory olfactory bulb granular layer-Cerebellum	0	0	0	0
Retrohippocampal region -Cerebellum	0.102883	0.440497	-0.55865	0.401161
Entorhinal area-Cerebellum	0	0.589819	-0.82227	0.20983
Field CA1-Cerebellum	0.057372	0.422045	0	0.481761
Field CA3-Cerebellum	0.06774	0.331723	-0.82227	0.196734
Dentate gyrus-Cerebellum	0.016795	0.339112	0.394188	0.037427
Field CA2 -Cerebellum	0	0	0	0
Accessory olfactory bulb mitral layer-Cerebellum	0	0	0	0
Striatum -Cerebellum	-0.54799	0.926892	0.95829	0.51274
Midbrain -Cerebellum	0.017793	0.012636	0.642717	0.042979
Medulla-Cerebellum	-0.41608	-0.36494	-0.83928	0.821468
ventricular systems-Superior colliculus	0.139355	0.739298	0.237782	-0.84577
Hypothalamus -Superior colliculus	0.153019	-0.30051	0.024213	-0.26193
Inferior colliculus -Superior colliculus	0.426682	-0.5505	0.045648	0.051542
periaqueductal gray-Superior colliculus	-0.01418	-0.23365	-0.88536	-0.19714
Isocortex -Superior colliculus	0.03014	-0.32505	0.839279	0.967478
Cortical amygdalar area -Superior colliculus	0.161168	-0.91885	0.291461	0.942624
Olfactory areas -Superior colliculus	-0.11568	-0.10443	-0.18701	-0.01479
Pons-Superior colliculus	-0.60145	-0.30051	-0.98164	-0.07934
Midbrain reticular nucleus-Superior colliculus	-0.00258	-0.08348	0.291461	-0.06843
Nucleus accumbens-Superior colliculus	-0.98877	-0.32505	-0.29146	-0.03743
fimbria-Superior colliculus	-0.97159	-0.42388	-0.16415	-0.01479
Anterior cingulate area-Superior colliculus	-0.01982	-0.03786	-0.04114	-0.06328
Somatomotor areas-Superior colliculus	-0.0812	-0.19339	-0.24657	-0.04341
Somatosensory areas-Superior colliculus	0.817726	-0.33863	-0.26056	-0.08668
piriform area-Superior colliculus	-0.16627	-0.24239	0.291461	-0.96379
Taenia tecta -Superior colliculus	0.298525	-0.28948	-0.34895	-0.01688
Accessory olfactory bulb glomerular layer-Superior colliculus	0	0	0	0
Accessory olfactory bulb granular layer-Superior colliculus	0	0	0	0

Retrohippocampal region -Superior colliculus	0.642987	-0.11593	0.784582	-0.35265
Entorhinal area-Superior colliculus	-0.09868	-0.19339	-0.75131	-0.18401
Field CA1-Superior colliculus	-0.84381	-0.19339	0.839279	-0.16888
Field CA3-Superior colliculus	-0.63154	-0.42388	0.981642	-0.30296
Dentate gyrus-Superior colliculus	0.254158	-0.46633	0.301985	-0.87479
Field CA2 -Superior colliculus	0.59836	-0.13839	0.237782	-0.1196
Accessory olfactory bulb mitral layer-Superior colliculus	0	0	0	0
Striatum -Superior colliculus	-0.05079	-0.10327	-0.82227	-0.03241
Midbrain -Superior colliculus	-0.06857	-0.17895	0.291461	-0.65287
Medulla-Superior colliculus	-0.05482	-0.05741	-0.48422	-0.11769
Hypothalamus -ventricular systems	0.166562	-0.812518	0.080648	0.853991
Inferior colliculus -ventricular systems	-0.351799	-0.308729	0.291461	-0.789931
periaqueductal gray-ventricular systems	-0.404699	-0.211267	-0.291461	-0.022896
Isocortex -ventricular systems	0.040994	0.943742	0.822274	-0.664959
Cortical amygdalar area -ventricular systems	0	0	0	0.369565
Olfactory areas -ventricular systems	-0.166282	-0.201099	-0.041135	-0.020125
Pons-ventricular systems	0.547985	-0.440497	-0.414462	-0.037427
Midbrain reticular nucleus-ventricular systems	-0.182776	-0.178949	0.456976	-0.291189
Nucleus accumbens-ventricular systems	0	0	0	0
fimbria-ventricular systems	0	0	0	0
Anterior cingulate area-ventricular systems	0	0	0	0
Somatomotor areas-ventricular systems	-0.186173	-0.300514	0	-0.05283
Somatosensory areas-ventricular systems	0.703009	0.837975	-0.484831	-0.372745
piriform area-ventricular systems	0	0	0	0
Taenia tecta -ventricular systems	0.898903	0	0	-0.056316
Accessory olfactory bulb glomerular layer-ventricular systems	0	0	0	0
Accessory olfactory bulb granular layer-ventricular systems	0	0	0	0
Retrohippocampal region -ventricular systems	0.989235	-0.178949	-0.483423	-0.574465
Entorhinal area-ventricular systems	0.654072	-0.4979	-0.572821	-0.413065
Field CA1-ventricular systems	0.642987	0.857184	-0.934551	-0.400838
Field CA3-ventricular systems	0.668697	0.423883	-0.742524	-0.119599
Dentate gyrus-ventricular systems	0.332008	0.961825	0.822274	-0.409674
Field CA2 -ventricular systems	0	0	0	0
Accessory olfactory bulb mitral layer-ventricular systems	0	0	0	0
Striatum -ventricular systems	-0.265888	-0.423883	-0.316632	-0.090875

Midbrain -ventricular systems	-0.266782	-0.11365	0.291461	-0.181734
Medulla-ventricular systems	-0.161348	-0.057414	-0.572821	-0.14065
Inferior colliculus -Hypothalamus	-0.011851	-0.201099	-0.661563	-0.507277
periaqueductal gray-Hypothalamus	-0.210438	0.537292	-0.301985	-0.074218
Isocortex -Hypothalamus	-0.490653	0.521851	-0.291461	-0.652874
Cortical amygdalar area -Hypothalamus	0	0	0	0
Olfactory areas -Hypothalamus	0	0	0	0
Pons-Hypothalamus	-0.457411	0	0	-0.297981
Midbrain reticular nucleus-Hypothalamus	-0.01066	-0.729583	0	-0.544776
Nucleus accumbens-Hypothalamus	0	0	0	0
fimbria-Hypothalamus	0	0	0	0
Anterior cingulate area-Hypothalamus	0	0	0	0
Somatomotor areas-Hypothalamus	0	0	0	0
Somatosensory areas-Hypothalamus	-0.696473	0.895441	0	-0.29166
piriform area-Hypothalamus	0	0	0	0
Taenia tecta -Hypothalamus	0	0	0	0
Accessory olfactory bulb glomerular layer-Hypothalamus	0	0	0	0
Accessory olfactory bulb granular layer-Hypothalamus	0	0	0	0
Retrohippocampal region -Hypothalamus	-0.098679	-0.289477	0	-0.811514
Entorhinal area-Hypothalamus	0	0	0	0.87316
Field CA1-Hypothalamus	0	0	-0.68108	-0.796283
Field CA3-Hypothalamus	0.798454	0.243212	0	-0.942624
Dentate gyrus-Hypothalamus	0.661328	0.4979	0.741503	0.959097
Field CA2 -Hypothalamus	0	0	0	0
Accessory olfactory bulb mitral layer-Hypothalamus	0	0	0	0
Striatum -Hypothalamus	-0.199455	-0.843059	-0.68108	-0.386962
Midbrain -Hypothalamus	-0.00986	-0.720051	-0.291461	-0.205571
Medulla-Hypothalamus	-0.195065	0	-0.581237	-0.648866
periaqueductal gray-Inferior colliculus	-0.15588	-0.91885	-0.61801	-0.83366
Isocortex -Inferior colliculus	-0.62432	0.083389	-0.36562	-0.89279
Cortical amygdalar area -Inferior colliculus	0	0	0	0
Olfactory areas -Inferior colliculus	0	0	0	-0.84234
Pons-Inferior colliculus	-0.05738	-0.23976	-0.36562	-0.01535
Midbrain reticular nucleus-Inferior colliculus	-0.02336	0.868184	0	-0.15396

Nucleus accumbens-Inferior colliculus	0	0	0	0
fimbria-Inferior colliculus	0	0	0	0
Anterior cingulate area-Inferior colliculus	0	0	0	0
Somatomotor areas-Inferior colliculus	-0.06977	-0.30051	0	-0.15565
Somatosensory areas-Inferior colliculus	-0.11568	-0.91168	0	0
piriform area-Inferior colliculus	0	0	0	0
Taenia tecta -Inferior colliculus	0	0	0	0
Accessory olfactory bulb glomerular layer-Inferior colliculus	0	0	0	0
Accessory olfactory bulb granular layer-Inferior colliculus	0	0	0	0
Retrohippocampal region -Inferior colliculus	0.969187	0.542765	0	-0.83366
Entorhinal area-Inferior colliculus	0	-0.91885	0	0.385712
Field CA1-Inferior colliculus	-0.19946	-0.96183	0	-0.68996
Field CA3-Inferior colliculus	-0.41608	0.4979	-0.53733	-0.6584
Dentate gyrus-Inferior colliculus	-0.8336	-0.99953	-0.45748	0.083082
Field CA2 -Inferior colliculus	0	0	0	0
Accessory olfactory bulb mitral layer-Inferior colliculus	0	0	0	0
Striatum -Inferior colliculus	-0.03264	-0.53861	-0.21981	-0.25902
Midbrain -Inferior colliculus	-0.0893	0.170171	-0.58124	-0.5428
Medulla-Inferior colliculus	-0.02102	-0.14161	-0.32296	-0.05127
Isocortex -periaqueductal gray	-0.21044	-0.18617	-0.29146	-0.06328
Cortical amygdalar area -periaqueductal gray	0	0	0	0
Olfactory areas -periaqueductal gray	-0.13936	0	-0.04979	-0.01535
Pons-periaqueductal gray	-0.17461	-0.19339	-0.79748	-0.01598
Midbrain reticular nucleus-periaqueductal gray	-0.08797	-0.01264	-0.95829	-0.03743
Nucleus accumbens-periaqueductal gray	0	0	0	0
fimbria-periaqueductal gray	0	0	0	0
Anterior cingulate area-periaqueductal gray	0	0	0	0
Somatomotor areas-periaqueductal gray	-0.09868	0	0	-0.015
Somatosensory areas-periaqueductal gray	-0.16628	-0.14988	-0.46745	-0.03743
piriform area-periaqueductal gray	0	0	0	0
Taenia tecta -periaqueductal gray	0	0	0	-0.29798
Accessory olfactory bulb glomerular layer-periaqueductal gray	0	0	0	0
Accessory olfactory bulb granular layer-periaqueductal gray	0	0	0	0
Retrohippocampal region -periaqueductal gray	-0.43172	-0.08339	-0.29927	-0.02717
Entorhinal area-periaqueductal gray	-0.58187	0	-0.09575	-0.0458

Field CA1-periaqueductal gray	-0.59836	-0.35138	-0.82227	-0.01479
Field CA3-periaqueductal gray	-0.84706	-0.91885	-0.46745	-0.21719
Dentate gyrus-periaqueductal gray	-0.65549	-0.44555	-0.98164	-0.13771
Field CA2 -periaqueductal gray	0	0	0	0
Accessory olfactory bulb mitral layer-periaqueductal gray	0	0	0	0
Striatum -periaqueductal gray	-0.02066	-0.11693	-0.18595	-0.00846
Midbrain -periaqueductal gray	-0.00593	-0.03786	-0.36835	-0.00826
Medulla-periaqueductal gray	-0.01138	-0.06336	-0.21778	-0.08308
Cortical amygdalar area -Isocortex	0.017793	0	0.403653	-0.81706
Olfactory areas -Isocortex	-0.18182	-0.17895	-0.04979	-0.0136
Pons-Isocortex	-0.64299	-0.19339	-0.18701	-0.06749
Midbrain reticular nucleus-Isocortex	-0.01138	-0.15889	0.68108	-0.01535
Nucleus accumbens-Isocortex	0	0	0	-0.51274
fimbria-Ictx	0	0	0	0
Anterior cingulate area-Isocortex	-0.02336	-0.08484	0	-0.02395
Somatomotor areas-Isocortex	-0.24997	-0.40055	-0.48483	-0.12394
Somatosensory areas-Isocortex	-0.98312	-0.30051	-0.29146	-0.35904
piriform area-Isocortex	-0.86741	0	0	-0.8813
Taenia tecta -Isocortex	0.689123	0	-0.14634	-0.1455
Accessory olfactory bulb glomerular layer-Isocortex	0	0	0	0
Accessory olfactory bulb granular layer-Isocortex	0	0	0	0
Retrohippocampal region -Isocortex	0.11568	-0.22176	-0.31132	0.702543
Entorhinal area-Isocortex	-0.21044	-0.31152	-0.13335	-0.03749
Field CA1-Isocortex	0.914811	-0.82729	-0.69832	-0.39887
Field CA3-Isocortex	-0.78354	-0.23976	-0.58937	-0.39744
Dentate gyrus-Isocortex	0.063874	-0.30051	0.822274	0.967478
Field CA2 -Isocortex	-0.82319	0	0.288044	-0.4933
Accessory olfactory bulb mitral layer-Isocortex	0	0	0	0
Striatum -Isocortex	-0.0812	-0.09471	-0.18595	-0.03743
Midbrain -Isocortex	-0.21953	-0.03786	-0.64051	-0.34789
Medulla-Isocortex	-0.04998	-0.19339	-0.29146	-0.20983
Olfactory areas -Cortical amygdalar area	0	0	0	0
Pons-Cortical amygdalar area	0	0	0	0
Midbrain reticular nucleus-Cortical amygdalar area	0	0	0	0

Nucleus accumbens-Cortical amygdalar area	0	0	0	0
fimbria-Cortical amygdalar area	0	0	0	0
Anterior cingulate area-Cortical amygdalar area	0	0	0	0
Somatomotor areas-Cortical amygdalar area	0	0	0	0
Somatosensory areas-Cortical amygdalar area	0	0	0	0
piriform area-Cortical amygdalar area	0	0	0	0
Taenia tecta -Cortical amygdalar area	0	0	0	0
Accessory olfactory bulb glomerular layer-Cortical amygdalar area	0	0	0	0
Accessory olfactory bulb granular layer-Cortical amygdalar area	0	0	0	0
Retrohippocampal region -Cortical amygdalar area	0	0	0	0
Entorhinal area-Cortical amygdalar area	0	0	0	0
Field CA1-Cortical amygdalar area	0	0	0	0
Field CA3-Cortical amygdalar area	0	0	0	0
Dentate gyrus-Cortical amygdalar area	0	0	0	0
Field CA2 -Cortical amygdalar area	0	0	0	0
Accessory olfactory bulb mitral layer-Cortical amygdalar area	0	0	0	0
Striatum -Cortical amygdalar area	0	0	0	0
Midbrain -Cortical amygdalar area	0.989235	-0.84031	0	0.519832
Medulla-Cortical amygdalar area	0	0	0	0
Pons-Olfactory areas	0	0	0	0
Midbrain reticular nucleus-Olfactory areas	0	0	0	0
Nucleus accumbens-Olfactory areas	0	0	0	0
fimbria-Olfactory areas	0	0	0	0
Anterior cingulate area-Olfactory areas	0	0	0	0
Somatomotor areas-Olfactory areas	0	0	0	0
Somatosensory areas-Olfactory areas	0	-0.19339	0	0
piriform area-Olfactory areas	0	0	0	0
Taenia tecta -Olfactory areas	0	0	0	0
Accessory olfactory bulb glomerular layer-Olfactory areas	0	0	0	0
Accessory olfactory bulb granular layer-Olfactory areas	0	0	0	0
Retrohippocampal region -Olfactory areas	0	0	0	0
Entorhinal area-Olfactory areas	0	0	0	0
Field CA1-Olfactory areas	0	0	0	0
Field CA3-Olfactory areas	0	0	0	0
Dentate gyrus-Olfactory areas	0	0	0	0

Field CA2 -Olfactory areas	0	0	0	0
Accessory olfactory bulb mitral layer-Olfactory areas	0	0	0	0
Striatum -Olfactory areas	0	0	0	0
Midbrain -Olfactory areas	-0.00593	-0.02345	-0.79748	-0.01479
Medulla-Olfactory areas	0	0	0	0
Midbrain reticular nucleus-Pons	0	0	0	0
Nucleus accumbens-Pons	0	0	0	0
fimbria-Pons	0	0	0	0
Anterior cingulate area-Pons	0	0	0	0
Somatomotor areas-Pons	0	0	0	0
Somatosensory areas-Pons	0	0	0	0
piriform area-Pons	0	0	0	0
Taenia tecta -Pons	0	0	0	0
Accessory olfactory bulb glomerular layer-Pons	0	0	0	0
Accessory olfactory bulb granular layer-Pons	0	0	0	0
Retrohippocampal region -Pons	-0.49065	0	0	0
Entorhinal area-Pons	0	0	0	0
Field CA1-Pons	0	-0.85718	0	0
Field CA3-Pons	0	0	0	0
Dentate gyrus-Pons	0	0	0	0
Field CA2 -Pons	0	0	0	0
Accessory olfactory bulb mitral layer-Pons	0	0	0	0
Striatum -Pons	0	0	0	0
Midbrain -Pons	-0.03264	-0.05741	-0.0643	-0.01479
Medulla-Pons	0	0	0	0
Nucleus accumbens-Midbrain reticular nucleus	0	0	0	0
fimbria-Midbrain reticular nucleus	0	0	0	0
Anterior cingulate area-Midbrain reticular nucleus	0	0	0	0
Somatomotor areas-Midbrain reticular nucleus	0	0	0	0
Somatosensory areas-Midbrain reticular nucleus	0	0	0	0
piriform area-Midbrain reticular nucleus	0	0	0	0
Taenia tecta -Midbrain reticular nucleus	0	0	0	0
Accessory olfactory bulb glomerular layer-Midbrain reticular nucleus	0	0	0	0
Accessory olfactory bulb granular layer-Midbrain reticular nucleus	0	0	0	0

Retrohippocampal region -Midbrain reticular nucleus	-0.0186	0	0	-0.37707
Entorhinal area-Midbrain reticular nucleus	0	0	0	0
Field CA1-Midbrain reticular nucleus	0	0	0	0
Field CA3-Midbrain reticular nucleus	-0.16135	0	0	0
Dentate gyrus-Midbrain reticular nucleus	-0.10573	0	0	-0.04293
Field CA2 -Midbrain reticular nucleus	0	0	0	0
Accessory olfactory bulb mitral layer-Midbrain reticular nucleus	0	0	0	0
Striatum -Midbrain reticular nucleus	-0.01982	0	0	0
Midbrain -Midbrain reticular nucleus	-0.00411	0.284031	-0.23778	-0.24008
Medulla-Midbrain reticular nucleus	0	0	0	0
fimbria-Nucleus accumbens	0	0	0	0
Anterior cingulate area-Nucleus accumbens	0	0	0	0
Somatomotor areas-Nucleus accumbens	0	0	0	0
Somatosensory areas-Nucleus accumbens	0	0	0	0
piriform area-Nucleus accumbens	0	0	0	0
Taenia tecta -Nucleus accumbens	0	0	0	0
Accessory olfactory bulb glomerular layer-Nucleus accumbens	0	0	0	0
Accessory olfactory bulb granular layer-Nucleus accumbens	0	0	0	0
Retrohippocampal region -Nucleus accumbens	0	0	0	0
Entorhinal area-Nucleus accumbens	0	0	0	0
Field CA1-Nucleus accumbens	0	0	0	0
Field CA3-Nucleus accumbens	0	0	0	0
Dentate gyrus-Nucleus accumbens	0	0	0	0
Field CA2 -Nucleus accumbens	0	0	0	0
Accessory olfactory bulb mitral layer-Nucleus accumbens	0	0	0	0
Striatum -Nucleus accumbens	0	0	0	0
Midbrain -Nucleus accumbens	0	0	0	0
Medulla-Nucleus accumbens	0	0	0	0
Anterior cingulate area-fimbria	0	0	0	0
Somatomotor areas-fimbria	0	0	0	0
Somatosensory areas-fimbria	0	0	0	0
piriform area-fimbria	0	0	0	0
Taenia tecta -fimbria	0	0	0	0
Accessory olfactory bulb glomerular layer-fimbria	0	0	0	0

Accessory olfactory bulb granular layer-fimbria	0	0	0	0
Retrohippocampal region -fimbria	0	0	0	0
Entorhinal area-fimbria	0	0	0	0
Field CA1-fimbria	0	0	0	0
Field CA3-fimbria	0	0	0	0
Dentate gyrus-fimbria	0	0	0	0
Field CA2 -fimbria	0	0	0	0
Accessory olfactory bulb mitral layer-fimbria	0	0	0	0
Striatum -fimbria	0	0	0	0
Midbrain -fimbria	0	0	0	-0.06328
Medulla-fimbria	0	0	0	0
Somatomotor areas-Anterior cingulate area	0	0	0	0
Somatosensory areas-Anterior cingulate area	0	0	0	0
piriform area-Anterior cingulate area	0	0	0	0
Taenia tecta -Anterior cingulate area	0	0	0	0
Accessory olfactory bulb glomerular layer-Anterior cingulate area	0	0	0	0
Accessory olfactory bulb granular layer-Anterior cingulate area	0	0	0	0
Retrohippocampal region -Anterior cingulate area	0	0	0	0
Entorhinal area-Anterior cingulate area	0	0	0	0
Field CA1-Anterior cingulate area	0	0	0	0
Field CA3-Anterior cingulate area	0	0	0	0
Dentate gyrus-Anterior cingulate area	0	0	0	0
Field CA2 -Anterior cingulate area	0	0	0	0
Accessory olfactory bulb mitral layer-Anterior cingulate area	0	0	0	0
Striatum -Anterior cingulate area	0	0	0	0
Midbrain -Anterior cingulate area	-0.00258	-0.03786	-0.12896	-0.08308
Medulla-Anterior cingulate area	0	0	0	0
Somatosensory areas-Somatomotor areas	0	0	0	0
piriform area-Somatomotor areas	0	0	0	0
Taenia tecta -Somatomotor areas	0	0	0	0
Accessory olfactory bulb glomerular layer-Somatomotor areas	0	0	0	0
Accessory olfactory bulb granular layer-Somatomotor areas	0	0	0	0
Retrohippocampal region -Somatomotor areas	0	0	0	0
Entorhinal area-Somatomotor areas	0	0	0	0

Field CA1-Somatomotor areas	0	0	0	0
Field CA3-Somatomotor areas	0	0	0	0
Dentate gyrus-Somatomotor areas	0	0	0	0
Field CA2 -Somatomotor areas	0	0	0	0
Accessory olfactory bulb mitral layer-Somatomotor areas	0	0	0	0
Striatum -Somatomotor areas	0	0	0	0
Midbrain -Somatomotor areas	-0.00258	-0.05741	-0.46745	-0.02013
Medulla-Somatomotor areas	0	0	0	0
piriform area-Somatosensory areas	0	0	0	0
Taenia tecta -Somatosensory areas	0	0	0	0
Accessory olfactory bulb glomerular layer-Somatosensory areas	0	0	0	0
Accessory olfactory bulb granular layer-Somatosensory areas	0	0	0	0
Retrohippocampal region -Somatosensory areas	-0.89639	0	-0.82227	0
Entorhinal area-Somatosensory areas	0	0	-0.29146	0
Field CA1-Somatosensory areas	0	0	0.631455	0
Field CA3--Somatosensory areas	0	0	-0.70814	0
Dentate gyrus-Somatosensory areas	-0.8336	0	0.742524	-0.78993
Field CA2 -Somatosensory areas	0	0	0	0
Accessory olfactory bulb mitral layer-Somatosensory areas	0	0	0	0
Striatum --Somatosensory areas	-0.25416	0	-0.48342	0
Midbrain --Somatosensory areas	-0.08565	-0.03786	-0.48342	-0.12196
Medulla-Somatosensory areas	0	0	0	0
Taenia tecta -piriform area	0	0	0	0
Accessory olfactory bulb glomerular layer-piriform area	0	0	0	0
Accessory olfactory bulb granular layer-piriform area	0	0	0	0
Retrohippocampal region -piriform area	0	0	0	0
Entorhinal area-piriform area	0	0	0	0
Field CA1-piriform area	0	0	0	0
Field CA3-piriform area	0	0	0	0
Dentate gyrus-piriform area	0	0	0	0
Field CA2 -piriform area	0	0	0	0
Accessory olfactory bulb mitral layer-piriform area	0	0	0	0
Striatum -piriform area	0	0	0	0
Midbrain -piriform area	-0.01369	0	0	0

Medulla-piriform area	0	0	0	0
Accessory olfactory bulb glomerular layer-Taenia tecta	0	0	0	0
Accessory olfactory bulb granular layer-Taenia tecta	0	0	0	0
Retrohippocampal region -Taenia tecta	0	0	0	0
Entorhinal area-Taenia tecta	0	0	0	0
Field CA1-Taenia tecta	0	0	0	0
Field CA3-Taenia tecta	0	0	0	0
Dentate gyrus-Taenia tecta	0	0	0	0
Field CA2 -Taenia tecta	0	0	0	0
Accessory olfactory bulb mitral layer-Taenia tecta	0	0	0	0
Striatum -Taenia tecta	0	0	0	0
Midbrain -Taenia tecta	-0.11288	-0.17425	-0.79748	-0.015
Medulla-Taenia tecta	0	0	0	0
Accessory olfactory bulb granular layer-Accessory olfactory bulb glomerular layer	0	0	0	0
Retrohippocampal region -Accessory olfactory bulb glomerular layer	0	0	0	0
Entorhinal area-Accessory olfactory bulb glomerular layer	0	0	0	0
Field CA1-Accessory olfactory bulb glomerular layer	0	0	0	0
Field CA3-Accessory olfactory bulb glomerular layer	0	0	0	0
Dentate gyrus-Accessory olfactory bulb glomerular layer	0	0	0	0
Field CA2 -Accessory olfactory bulb glomerular layer	0	0	0	0
Accessory olfactory bulb mitral layer-Accessory olfactory bulb glomerular layer	0	0	0	0
Striatum -Accessory olfactory bulb glomerular layer	0	0	0	0
Midbrain -Accessory olfactory bulb glomerular layer	0	0	0	0
Medulla-Accessory olfactory bulb glomerular layer	0	0	0	0
Retrohippocampal region -Accessory olfactory bulb granular layer	0	0	0	0
Entorhinal area-Accessory olfactory bulb granular layer	0	0	0	0
Field CA1-Accessory olfactory bulb granular layer	0	0	0	0
Field CA3-Accessory olfactory bulb granular layer	0	0	0	0
Dentate gyrus-Accessory olfactory bulb granular layer	0	0	0	0
Field CA2 -Accessory olfactory bulb granular layer	0	0	0	0
Accessory olfactory bulb mitral layer-Accessory olfactory bulb granular layer	0	0	0	0
Striatum -Accessory olfactory bulb granular layer	0	0	0	0

Midbrain -Accessory olfactory bulb granular layer	0	0	0	0
Medulla-Accessory olfactory bulb granular layer	0	0	0	0
Entorhinal area-Retrohippocampal region	0	0	0	0
Field CA1-Retrohippocampal region	0	0	0	0
Field CA3-Retrohippocampal region	0.696473	0	-0.83928	0
Dentate gyrus-Retrohippocampal region	0.182847	0	-0.99486	0.51274
Field CA2 -Retrohippocampal region	0	0	0	0
Accessory olfactory bulb mitral layer-Retrohippocampal region	0	0	0	0
Striatum -Retrohippocampal region	-0.16656	0	0	0
Midbrain -Retrohippocampal region	-0.04466	-0.17895	-0.35998	-0.04367
Medulla-Retrohippocampal region	0	0	-0.46745	0
Field CA1-Entorhinal area	0	0	0	0
Field CA3-Entorhinal area	0	0	0	0
Dentate gyrus-Entorhinal area	0	0	0	0
Field CA2 -Entorhinal area	0	0	0	0
Accessory olfactory bulb mitral layer-Entorhinal area	0	0	0	0
Striatum -Entorhinal area	0	0	0	0
Midbrain -Entorhinal area	-0.00534	-0.17017	-0.20952	-0.07771
Medulla-Entorhinal area	0	0	0	0
Field CA3-Field CA1	0	0	0	0
Dentate gyrus-Field CA1	0	0	0	0
Field CA2 -Field CA1	0	0	0	0
Accessory olfactory bulb mitral layer-Field CA1	0	0	0	0
Striatum -Field CA1	0	0	0	0
Midbrain -Field CA1	-0.13401	-0.34819	-0.58937	-0.05795
Medulla-Field CA1	0	0	0	0
Dentate gyrus-Field CA3	0	-0.86669	0	0
Field CA2 -Field CA3	0	0	0	0
Accessory olfactory bulb mitral layer-Field CA3	0	0	0	0
Striatum -Field CA3	0	0	0	0
Midbrain -Field CA3	-0.42014	-0.12889	0.893607	-0.13139
Medulla-Field CA3	0	0	0	0

Field CA2 -Dentate gyrus	0	0	0	0
Accessory olfactory bulb mitral layer-Dentate gyrus	0	0	0	0
Striatum -Dentate gyrus	-0.31072	0	-0.29146	-0.51983
Midbrain -Dentate gyrus	-0.16135	-0.51493	-0.48342	-0.09452
Medulla-Dentate gyrus	0	0	-0.52183	0
Accessory olfactory bulb mitral layer-Field CA2	0	0	0	0
Striatum -Field CA2	0	0	0	0
Midbrain -Field CA2	-0.02894	-0.14089	-0.92877	-0.07422
Medulla-Field CA2	0	0	0	0
Striatum -Accessory olfactory bulb mitral layer	0	0	0	0
Midbrain -Accessory olfactory bulb mitral layer	0	0	0	0
Medulla-Accessory olfactory bulb mitral layer	0	0	0	0
Midbrain -Striatum	-0.00449	-0.05655	-0.28802	-0.04298
Medulla-Striatum	-0.16117	0	-0.23778	-0.16146
Medulla-Midbrain	-0.00318	-0.05741	-0.06649	-0.02013

S8. Adjust p values for group comparisons of pair-wised connectivity for Hippocampus.

Region Connections	Hippocampus			
	Left Ipsilateral	Left Contralateral	Right Contralateral	Right Ipsilateral
Caudoputamen-corpus callosum	-0.82317	-0.16254	-0.0286	0.280144
anterior commissure olfactory limb-corpus callosum	0	0	0	0
pallidum -corpus callosum	-0.0136	-0.00813	-0.0111	-0.11707
internal capsule-corpus callosum	-0.08887	0	-0.32151	0.666789
Thalamus-corpus callosum	-0.01586	-0.48422	-0.05423	-0.19189
Cerebellum-corpus callosum	-0.15104	-0.12399	-0.03585	-0.05338
Superior colliculus-corpus callosum	-0.21244	-0.23083	-0.01335	-0.11841
ventricular systems-corpus callosum	0.133296	-0.91075	-0.17618	0.119686
Hypothalamus -corpus callosum	-0.01006	-0.02482	-0.01982	-0.06513
Inferior colliculus -corpus callosum	-0.02808	-0.48659	-0.19745	-0.15723
periaqueductal gray-corpus callosum	-0.00196	-0.05465	-0.00269	-0.02897
Isocortex -corpus callosum	0.138853	-0.26814	-0.01283	0.332891
Cortical amygdalar area -corpus callosum	0.215703	-0.06776	-0.00567	-0.8135
Olfactory areas -corpus callosum	-0.05232	-0.13907	-0.04979	-0.06011
Pons-corpus callosum	-0.0039	-0.01388	-0.00269	-0.00519
Midbrain reticular nucleus-corpus callosum	-0.05698	0	0	-0.56104
Nucleus accumbens-corpus callosum	-0.03386	-0.08335	-0.12714	-0.03368
fimbria-corpus callosum	0.180288	-0.05636	-0.00135	0.112922
Anterior cingulate area-corpus callosum	-0.05418	-0.09032	-0.89321	0.682714
Somatomotor areas-corpus callosum	-0.86157	0.301283	-0.79824	0.666789
Somatosensory areas-corpus callosum	0.026225	0.439656	-0.4013	0.257233
piriform area-corpus callosum	0.22466	-0.53048	-0.86657	0.40127
Taenia tecta -corpus callosum	-0.40041	0.974713	-0.09299	-0.53957
Accessory olfactory bulb glomerular layer-corpus callosum	0	0	0	0
Accessory olfactory bulb granular layer-corpus callosum	0	0	0	0
Retrohippocampal region -corpus callosum	-0.20382	-0.00767	-0.00549	-0.03398
Entorhinal area-corpus callosum	0.928871	-0.00767	-0.00508	-0.33742
Field CA1-corpus callosum	0.852543	-0.39153	-0.36226	0.937736
Field CA3-corpus callosum	0.421709	-0.02381	-0.0111	0.785383
Dentate gyrus-corpus callosum	-0.38906	-0.06839	-0.0845	-0.7211
Field CA2 -corpus callosum	0.491758	-0.07369	-0.01982	0.640328
Accessory olfactory bulb mitral layer-corpus callosum	0	0	0	0

Striatum -corpus callosum	-0.01074	-0.01229	-0.00729	-0.4544
Midbrain -corpus callosum	-0.00532	-0.01198	-0.0064	-0.02341
Medulla-corpus callosum	-0.01006	-0.01027	-0.00873	-0.0079
anterior commissure olfactory limb-Caudoputamen	0	0	0	0
pallidum -Caudoputamen	-0.133647	-0.042921	-0.011828	-0.191625
internal capsule-Caudoputamen	0.338112	0	0	0
Thalamus-Caudoputamen	0.018921	-0.743198	-0.341023	0.10072
Cerebellum-Caudoputamen	-0.304444	-0.03379	-0.206628	-0.134901
Superior colliculus-Caudoputamen	0.78988	0.705242	-0.165057	-0.192103
ventricular systems-Caudoputamen	0.013595	-0.525805	-0.351901	0.080157
Hypothalamus -Caudoputamen	-0.486266	-0.007666	-0.403444	-0.060113
Inferior colliculus -Caudoputamen	-0.091083	0	-0.123665	-0.192755
periaqueductal gray-Caudoputamen	-0.922694	-0.010492	-0.006773	-0.058526
Isocortex -Caudoputamen	0.754398	-0.805178	-0.026973	0.271579
Cortical amygdalar area -Caudoputamen	0.180295	0	-0.080744	0.884139
Olfactory areas -Caudoputamen	-0.905274	-0.303993	-0.426967	-0.090994
Pons-Caudoputamen	-0.009851	-0.01424	-0.008728	-0.005194
Midbrain reticular nucleus-Caudoputamen	0.138853	0	0	0
Nucleus accumbens-Caudoputamen	0.43909	0	0	-0.168648
fimbria-Caudoputamen	0.01661	0	0	0.066259
Anterior cingulate area-Caudoputamen	-0.113849	-0.082965	-0.125661	-0.980782
Somatomotor areas-Caudoputamen	-0.360966	-0.635634	-0.34039	0.636045
Somatosensory areas-Caudoputamen	0.046345	-0.80543	-0.113919	0.069459
piriform area-Caudoputamen	0.68301	0.801197	0.603127	0.232075
Taenia tecta -Caudoputamen	0.159202	-0.981071	-0.863597	-0.232075
Accessory olfactory bulb glomerular layer-Caudoputamen	0	0	0	0
Accessory olfactory bulb granular layer-Caudoputamen	0	0	0	0
Retrohippocampal region -Caudoputamen	-0.007397	-0.007666	-0.006773	-0.005959
Entorhinal area-Caudoputamen	-0.088419	-0.010271	-0.006773	-0.04963
Field CA1-Caudoputamen	-0.365056	-0.139074	-0.01982	0.682714
Field CA3-Caudoputamen	0.01691	-0.027509	-0.06287	0.013948
Dentate gyrus-Caudoputamen	-0.824087	-0.103887	-0.154384	0.568315
Field CA2 -Caudoputamen	0.325898	-0.084479	-0.026973	0.687326
Accessory olfactory bulb mitral layer-Caudoputamen	0	0	0	0
Striatum -Caudoputamen	-0.119569	-0.023767	-0.049792	-0.276883
Midbrain -Caudoputamen	-0.21382	-0.007666	-0.01982	-0.019809

Medulla-Caudoputamen	-0.013595	-0.007666	-0.061098	-0.007393
pallidum -anterior commissure olfactory limb	0	0	0	0
internal capsule-anterior commissure olfactory limb	0	0	0	0
Thalamus-anterior commissure olfactory limb	0	0	0	0
Cerebellum-anterior commissure olfactory limb	0	0	0	0
Superior colliculus-anterior commissure olfactory limb	0	0	0	0
ventricular systems-anterior commissure olfactory limb	0	0	0	0
Hypothalamus -anterior commissure olfactory limb	0	0	0	0
Inferior colliculus -anterior commissure olfactory limb	0	0	0	0
periaqueductal gray-anterior commissure olfactory limb	0	0	0	0
Isocortex -anterior commissure olfactory limb	-0.418412	-0.854117	0	-0.023414
Cortical amygdalar area -anterior commissure olfactory limb	0	0	0	0
Olfactory areas -anterior commissure olfactory limb	0	0	0	0
Pons-anterior commissure olfactory limb	0	0	0	0
Midbrain reticular nucleus-anterior commissure olfactory limb	0	0	0	0
Nucleus accumbens-anterior commissure olfactory limb	0	0	0	0
fimbria-anterior commissure olfactory limb	0	0	0	0
Anterior cingulate area-anterior commissure olfactory limb	0	0	0	0
Somatomotor areas-anterior commissure olfactory limb	0	0	0	0
Somatosensory areas-anterior commissure olfactory limb	0	0	0	0
piriform area-anterior commissure olfactory limb	0	0	0	0
Taenia tecta -anterior commissure olfactory limb	0	0	0	0
Accessory olfactory bulb glomerular layer-anterior commissure olfactory limb	0	0	0	0
Accessory olfactory bulb granular layer-anterior commissure olfactory limb	0	0	0	0
Retrohippocampal region -anterior commissure olfactory limb	-0.164639	0	0	-0.144871
Entorhinal area-anterior commissure olfactory limb	0	0	0	0
Field CA1-anterior commissure olfactory limb	-0.155619	0	0	-0.112922
Field CA3-anterior commissure olfactory limb	-0.660625	0	0	-0.023414
Dentate gyrus-anterior commissure olfactory limb	-0.392937	0	0	-0.235354
Field CA2 -anterior commissure olfactory limb	0	0	0	0
Accessory olfactory bulb mitral layer-anterior commissure olfactory limb	0	0	0	0
Striatum -anterior commissure olfactory limb	0	0	0	0
Midbrain -anterior commissure olfactory limb	0	0	0	0
Medulla-anterior commissure olfactory limb	0	0	0	0

internal capsule-pallidum	0	0	0	0
Thalamus-pallidum	-0.418412	-0.079139	-0.054353	-0.505718
Cerebellum-pallidum	-0.048058	-0.121074	-0.092986	-0.040228
Superior colliculus-pallidum	-0.050295	-0.642683	-0.256548	-0.028969
ventricular systems-pallidum	-0.166922	-0.174272	-0.006773	-0.237562
Hypothalamus -pallidum	-0.057938	-0.03379	-0.032724	-0.053384
Inferior colliculus -pallidum	0	0	0	-0.040416
periaqueductal gray-pallidum	-0.119431	-0.122891	0	0
Isocortex -pallidum	-0.10107	-0.218615	-0.029544	-0.0079
Cortical amygdalar area -pallidum	0.20136	0	0	-0.455702
Olfactory areas -pallidum	-0.0546	-0.074967	-0.02886	-0.028969
Pons-pallidum	-0.026208	-0.06318	-0.293934	-0.01527
Midbrain reticular nucleus-pallidum	0	0	0	0
Nucleus accumbens-pallidum	0	0	0	-0.198732
fimbria-pallidum	0.056978	-0.570461	0	-0.445433
AVA-pallidum	0	0	0	-0.454026
Somatomotor areas-pallidum	0	-0.286435	-0.136215	-0.198732
Somatosensory areas-pallidum	-0.599145	-0.079139	-0.200703	-0.981408
piriform area-pallidum	-0.204885	-0.260766	-0.320267	-0.943926
Taenia tecta -pallidum	-0.093119	-0.054962	-0.049792	-0.040693
Accessory olfactory bulb glomerular layer-pallidum	0	0	0	0
Accessory olfactory bulb granular layer-pallidum	0	0	0	0
Retrohippocampal region -pallidum	-0.013595	-0.007666	-0.011096	-0.013948
Entorhinal area-pallidum	-0.007397	-0.011094	-0.014376	-0.005194
Field CA1-pallidum	-0.009851	-0.007666	-0.00685	-0.024665
Field CA3-pallidum	-0.052319	-0.017562	-0.007287	-0.049997
Dentate gyrus-pallidum	-0.138853	-0.039026	-0.01982	-0.343715
Field CA2 -pallidum	-0.0043	-0.007666	-0.006399	-0.018979
Accessory olfactory bulb mitral layer-pallidum	0	0	0	0
Striatum -pallidum	-0.034809	-0.008162	-0.008728	-0.060113
Midbrain -pallidum	-0.0043	-0.008133	-0.025218	-0.009086
Medulla-pallidum	0	0	-0.292074	-0.191625
Thalamus-internal capsule	0.338112	0	0	0.005959
Cerebellum-internal capsule	0.346031	0	0	0.135478
Superior colliculus-internal capsule	0.78988	0	0	0

ventricular systems-internal capsule	0.016083	0	0	0.033524
Hypothalamus -internal capsule	0.495239	0	0	0.01527
Inferior colliculus -internal capsule	0	0	0	0
periaqueductal gray-internal capsule	0	0	0	0
Isocortex -internal capsule	-0.433366	0.790812	-0.267656	-0.924605
Cortical amygdalar area -internal capsule	0	0	0	0
Olfactory areas -internal capsule	0	0	0	0
Pons-internal capsule	0	0	0	-0.269516
Midbrain reticular nucleus-internal capsule	0	0	0	0
Nucleus accumbens-internal capsule	0	0	0	0
fimbria-internal capsule	0	0	0	0
Anterior cingulate area-internal capsule	0	0	0	0
Somatomotor areas-internal capsule	0	0	0	0
Somatosensory areas-internal capsule	0.062308	0.805178	0	0
piriform area-internal capsule	0	0	0	0
Taenia tecta -internal capsule	0	0	0	0
Accessory olfactory bulb glomerular layer-internal capsule	0	0	0	0
Accessory olfactory bulb granular layer-internal capsule	0	0	0	0
Retrohippocampal region -internal capsule	-0.180288	0	0	-0.023414
Entorhinal area-internal capsule	-0.366688	0	0	-0.665239
Field CA1-internal capsule	-0.84535	0	0	0.595245
Field CA3-internal capsule	0.022456	0	-0.433307	0.004914
Dentate gyrus-internal capsule	0.61544	0	-0.937016	0.005496
Field CA2 -internal capsule	-0.769347	0	0	0.251543
Accessory olfactory bulb mitral layer-internal capsule	0	0	0	0
Striatum -internal capsule	0.347869	0	0	0.251543
Midbrain --internal capsule	0	0	0	0.74689
Medulla-internal capsule	0	0	0	0
Cerebellum-Thalamus	0.349012	0.253538	0.542092	0.032464
Superior colliculus-Thalamus	0.920108	0.010271	0.452602	0.532935
ventricular systems-Thalamus	0.159202	-0.53759	0.947619	0.050772
Hypothalamus -Thalamus	-0.34787	-0.16246	0.864533	-0.29663
Inferior colliculus -Thalamus	-0.15842	0	0.43404	0.10573
periaqueductal gray-Thalamus	-0.04383	-0.64042	-0.01342	-0.38787
Isocortex -Thalamus	-0.81508	-0.4501	-0.44318	-0.18352

Cortical amygdalar area -Thalamus	0.045953	0.363023	0.924052	0.643693
Olfactory areas -Thalamus	-0.13365	-0.0805	-0.05474	-0.07336
Pons-Thalamus	-0.02623	-0.11519	-0.0093	-0.32512
Midbrain reticular nucleus-Thalamus	-0.1592	0	0	0.923635
Nucleus accumbens-Thalamus	-0.7544	0	0	-0.13182
fimbria-Thalamus	0.07087	-0.21693	-0.20555	0.339394
Anterior cingulate area-Thalamus	-0.66063	-0.03379	0	-0.26002
Somatomotor areas-Thalamus	-0.19913	-0.41018	0.936276	-0.19873
Somatosensory areas-Thalamus	0.07087	0.866605	-0.7783	-0.23535
piriform area-Thalamus	-0.82803	-0.66069	0.52666	0.376936
Taenia tecta -Thalamus	0.84535	-0.42542	-0.37776	-0.04289
Accessory olfactory bulb glomerular layer-Thalamus	0	0	0	0
Accessory olfactory bulb granular layer-Thalamus	0	0	0	0
Retrohippocampal region -Thalamus	-0.08937	-0.39078	-0.33471	-0.02158
Entorhinal area-Thalamus	-0.0136	-0.01388	-0.05435	-0.40216
Field CA1-Thalamus	-0.06857	0.953546	0.840362	-0.04072
Field CA3-Thalamus	0.857625	0.974713	-0.66383	0.354091
Dentate gyrus-Thalamus	-0.13709	0.439656	0.174611	0.967138
Field CA2 -Thalamus	-0.02409	-0.39924	-0.66618	-0.26756
Accessory olfactory bulb mitral layer-Thalamus	0	0	0	0
Striatum -Thalamus	-0.19543	-0.03929	-0.27892	-0.74096
Midbrain -Thalamus	-0.0039	-0.19732	-0.42697	0.48999
Medulla-Thalamus	-0.03114	-0.39864	-0.13983	-0.37694
Superior colliculus-Cerebellum	0.016083	0.017055	0.784213	0.785383
ventricular systems-Cerebellum	0.259162	-0.69314	0.724614	-0.56104
Hypothalamus -Cerebellum	0.045953	-0.33162	0.35936	0.021578
Inferior colliculus -Cerebellum	0.957084	0	0.787852	0.140359
periaqueductal gray-Cerebellum	0.418412	0.526738	0.914381	-0.56911
Isocortex -Cerebellum	0.151042	-0.8012	-0.48991	0.335076
Cortical amygdalar area -Cerebellum	0.001964	0.007651	0.097287	0.066065
Olfactory areas -Cerebellum	-0.11943	-0.08822	-0.14617	-0.14755
Pons-Cerebellum	0.520647	0.48659	-0.87046	0.964928
Midbrain reticular nucleus-Cerebellum	0	0	0	0
Nucleus accumbens-Cerebellum	-0.23541	0	0	-0.19163
fimbria-Cerebellum	0	0	0	-0.14352

Anterior cingulate area-Cerebellum	0	0	0	-0.08852
Somatomotor areas-Cerebellum	-0.11957	-0.17562	-0.35121	-0.1349
Somatosensory areas-Cerebellum	-0.78988	-0.64268	-0.21702	-0.0361
piriform area-Cerebellum	-0.59164	-0.26154	0	0.880806
Taenia tecta -Cerebellum	-0.4787	-0.52624	-0.0121	-0.00433
Accessory olfactory bulb glomerular layer-Cerebellum	0	0	0	0
Accessory olfactory bulb granular layer-Cerebellum	0	0	0	0
Retrohippocampal region -Cerebellum	0.019472	-0.98358	-0.49457	0.926274
Entorhinal area-Cerebellum	0.037436	-0.29876	-0.7783	0.016036
Field CA1-Cerebellum	0.038926	-0.49261	-0.37609	-0.94393
Field CA3-Cerebellum	0.000521	0.093259	0.446327	0.028969
Dentate gyrus-Cerebellum	0.000521	0.137651	0.246624	0.023414
Field CA2 -Cerebellum	-0.65719	-0.00767	-0.17775	-0.72247
Accessory olfactory bulb mitral layer-Cerebellum	0	0	0	0
Striatum -Cerebellum	-0.22392	-0.10341	-0.05474	-0.19189
Midbrain -Cerebellum	0.204885	0.648721	0.012496	0.028969
Medulla-Cerebellum	0	0	0.79379	0.600512
ventricular systems-Superior colliculus	0.176089	-0.91075	0.005665	-0.89772
Hypothalamus -Superior colliculus	0.316374	-0.94214	0.014537	-0.45403
Inferior colliculus -Superior colliculus	-0.48246	0	0.113919	-0.40733
periaqueductal gray-Superior colliculus	0.961961	0.854117	0.788018	-0.19794
Isocortex -Superior colliculus	0.117453	-0.28397	0.666184	-0.63492
Cortical amygdalar area -Superior colliculus	0.271504	0	0.097287	0.626157
Olfactory areas -Superior colliculus	-0.14439	-0.09789	-0.05435	-0.00908
Pons-Superior colliculus	0.78988	-0.39735	-0.43945	-0.10429
Midbrain reticular nucleus-Superior colliculus	-0.48246	0	0	-0.31917
Nucleus accumbens-Superior colliculus	0.657188	0	0	-0.18219
fimbria-Superior colliculus	0.438981	0	-0.41471	-0.63492
Anterior cingulate area-Superior colliculus	0	-0.00765	0	-0.05157
Somatomotor areas-Superior colliculus	-0.54237	-0.12334	0.914381	-0.06011
Somatosensory areas-Superior colliculus	0.105898	-0.49261	-0.1383	-0.24313
piriform area-Superior colliculus	-0.18029	0	0.167392	-0.57847
Taenia tecta -Superior colliculus	0.861572	-0.42485	-0.50748	-0.13973
Accessory olfactory bulb glomerular layer-Superior colliculus	0	0	0	0
Accessory olfactory bulb granular layer-Superior colliculus	0	0	0	0

Retrohippocampal region -Superior colliculus	-0.20783	-0.03366	0.935687	-0.02341
Entorhinal area-Superior colliculus	-0.11345	-0.2392	0.875326	-0.26068
Field CA1-Superior colliculus	-0.96292	-0.07017	0.951979	-0.35184
Field CA3-Superior colliculus	-0.75342	-0.61486	0.734958	-0.46469
Dentate gyrus-Superior colliculus	0.389055	-0.61593	0.134354	-0.92364
Field CA2 -Superior colliculus	0.500366	-0.02552	0.666184	-0.53333
Accessory olfactory bulb mitral layer-Superior colliculus	0	0	0	0
Striatum -Superior colliculus	-0.04211	-0.13259	-0.66179	-0.04242
Midbrain -Superior colliculus	-0.18029	-0.30399	0.18781	-0.19276
Medulla-Superior colliculus	-0.55573	-0.3406	0.890552	-0.82951
Hypothalamus -ventricular systems	-0.810003	-0.175623	0.371126	-0.320069
Inferior colliculus -ventricular systems	-0.329154	-0.173645	-0.817345	-0.841442
periaqueductal gray-ventricular systems	-0.769963	-0.435985	-0.301604	-0.276883
Isocortex -ventricular systems	0.146767	-0.875125	-0.341969	0.884139
Cortical amygdalar area -ventricular systems	0.102381	0.958637	-0.768622	0.352913
Olfactory areas -ventricular systems	-0.791632	-0.098948	-0.028471	-0.178145
Pons-ventricular systems	-0.054126	-0.023374	-0.013345	-0.010783
Midbrain reticular nucleus-ventricular systems	0.572938	-0.128494	0	-0.9441
Nucleus accumbens-ventricular systems	-0.754398	-0.046776	-0.087021	-0.046466
fimbria-ventricular systems	0.425072	-0.017332	-0.221857	-0.93774
Anterior cingulate area-ventricular systems	-0.346031	-0.027251	-0.292074	0.634918
Somatomotor areas-ventricular systems	-0.548538	-0.764217	-0.188188	0.912761
Somatosensory areas-ventricular systems	0.09568	-0.983581	-0.160767	0.178427
piriform area-ventricular systems	0.07087	-0.097892	0.86909	0.276883
Taenia tecta -ventricular systems	0.35804	0.977249	-0.146709	-0.076636
Accessory olfactory bulb glomerular layer-ventricular systems	0	0	0	0
Accessory olfactory bulb granular layer-ventricular systems	0	0	0	0
Retrohippocampal region -ventricular systems	-0.048058	-0.023374	-0.021792	-0.028969
Entorhinal area-ventricular systems	-0.184792	-0.013878	-0.035264	-0.354091
Field CA1-ventricular systems	-0.104048	-0.056355	-0.147371	-0.806277
Field CA3-ventricular systems	0.961961	-0.014401	-0.087021	0.436579
Dentate gyrus-ventricular systems	-0.264708	-0.123009	-0.461047	-0.588122
Field CA2 -ventricular systems	-0.066618	-0.008109	-0.307156	0.436371
Accessory olfactory bulb mitral layer-ventricular systems	0	0	0	0
Striatum -ventricular systems	-0.101371	-0.007651	-0.184896	-0.368886

Midbrain -ventricular systems	-0.204885	-0.063609	0.54131	-0.70828
Medulla-ventricular systems	-0.057387	-0.05382	-0.054784	-0.064149
Inferior colliculus -Hypothalamus	-0.861572	0	-0.837102	0.997396
periaqueductal gray-Hypothalamus	-0.405958	0.670508	-0.208706	0.583017
Isocortex -Hypothalamus	-0.155619	-0.385895	-0.020958	-0.024665
Cortical amygdalar area -Hypothalamus	0.087849	0.009482	-0.134354	0.182189
Olfactory areas -Hypothalamus	-0.056978	-0.097892	-0.024419	-0.021578
Pons-Hypothalamus	-0.046372	-0.705242	-0.106734	-0.177315
Midbrain reticular nucleus-Hypothalamus	0	0	0	0
Nucleus accumbens-Hypothalamus	-0.4426	0	0	0
fimbria-Hypothalamus	-0.478702	-0.84836	0	-0.040693
Anterior cingulate area-Hypothalamus	0	0	0	0
Somatomotor areas-Hypothalamus	-0.100892	-0.164132	-0.176456	-0.262012
Somatosensory areas-Hypothalamus	-0.396962	-0.457976	-0.021595	-0.028969
piriform area-Hypothalamus	-0.324744	-0.434643	0	0.899192
Taenia tecta -Hypothalamus	-0.055208	-0.659327	-0.033273	-0.15723
Accessory olfactory bulb glomerular layer-Hypothalamus	0	0	0	0
Accessory olfactory bulb granular layer-Hypothalamus	0	0	0	0
Retrohippocampal region -Hypothalamus	-0.04211	-0.144672	-0.007287	-0.022967
Entorhinal area-Hypothalamus	-0.07087	-0.090322	-0.00269	-0.021578
Field CA1-Hypothalamus	-0.014264	-0.097892	-0.007979	-0.037736
Field CA3-Hypothalamus	-0.27539	-0.750081	-0.025053	-0.191625
Dentate gyrus-Hypothalamus	-0.43909	-0.836557	-0.130532	-0.569609
Field CA2 -Hypothalamus	-0.017565	-0.038525	-0.007287	-0.042417
Accessory olfactory bulb mitral layer-Hypothalamus	0	0	0	0
Striatum -Hypothalamus	-0.205688	-0.044957	-0.013423	-0.277193
Midbrain -Hypothalamus	-0.113446	-0.575573	-0.160767	0.295834
Medulla-Hypothalamus	-0.271504	0	-0.160767	-0.55312
periaqueductal gray-Inferior colliculus	0	0	0	0
Isocortex -Inferior colliculus	-0.29954	0.997062	-0.21794	-0.83009
Cortical amygdalar area -Inferior colliculus	0	0	0	0.175903
Olfactory areas -Inferior colliculus	0	0	0	-0.1486
Pons-Inferior colliculus	0	0	0	0
Midbrain reticular nucleus-Inferior colliculus	0	0	0	0

Nucleus accumbens-Inferior colliculus	0	0	0	0
fimbria-Inferior colliculus	0	0	0	0
Anterior cingulate area-Inferior colliculus	0	0	0	0
Somatomotor areas-Inferior colliculus	0	0	0	-0.08857
Somatosensory areas-Inferior colliculus	-0.33281	-0.66746	0	-0.10573
piriform area-Inferior colliculus	0	0	0	0.7211
Taenia tecta -Inferior colliculus	0	0	0	0
Accessory olfactory bulb glomerular layer-Inferior colliculus	0	0	0	0
Accessory olfactory bulb granular layer-Inferior colliculus	0	0	0	0
Retrohippocampal region -Inferior colliculus	-0.02409	-0.88286	0	-0.82992
Entorhinal area-Inferior colliculus	-0.07087	0	-0.30129	0.665999
Field CA1-Inferior colliculus	-0.20489	-0.58186	0	0.83022
Field CA3-Inferior colliculus	-0.46959	0.646609	-0.40551	0.709069
Dentate gyrus-Inferior colliculus	-0.23714	-0.66069	-0.43404	0.183518
Field CA2 -Inferior colliculus	0	0	0	-0.81949
Accessory olfactory bulb mitral layer-Inferior colliculus	0	0	0	0
Striatum -Inferior colliculus	-0.00813	-0.06549	-0.11767	-0.24848
Midbrain -Inferior colliculus	-0.20783	-0.78498	0	-0.64241
Medulla-Inferior colliculus	0	0	0	-0.58703
Isocortex -periaqueductal gray	-0.58294	-0.08229	-0.1383	-0.06011
Cortical amygdalar area -periaqueductal gray	0.037922	0	0	0.347813
Olfactory areas -periaqueductal gray	-0.01586	-0.18184	-0.0286	-0.00302
Pons-periaqueductal gray	0	0	0	0
Midbrain reticular nucleus-periaqueductal gray	0	0	0	0
Nucleus accumbens-periaqueductal gray	0	0	0	0
fimbria-periaqueductal gray	0	0	0	0
Anterior cingulate area-periaqueductal gray	0	0	0	0
Somatomotor areas-periaqueductal gray	0	-0.12849	0	0
Somatosensory areas-periaqueductal gray	-0.74897	-0.09435	-0.05435	-0.01768
piriform area-periaqueductal gray	0	0	0	0
Taenia tecta -periaqueductal gray	0	0	0	0
Accessory olfactory bulb glomerular layer-periaqueductal gray	0	0	0	0
Accessory olfactory bulb granular layer-periaqueductal gray	0	0	0	0
Retrohippocampal region -periaqueductal gray	-0.31926	-0.01049	-0.22186	-0.0079
Entorhinal area-periaqueductal gray	-0.13365	-0.01398	-0.04319	-0.03847

Field CA1-periaqueductal gray	-0.28172	-0.03703	-0.2178	-0.0372
Field CA3-periaqueductal gray	0.145099	-0.48659	0.637814	-0.4989
Dentate gyrus-periaqueductal gray	0.124807	-0.20014	0.461047	-0.90556
Field CA2 -periaqueductal gray	-0.04383	-0.01555	-0.22302	-0.27158
Accessory olfactory bulb mitral layer-periaqueductal gray	0	0	0	0
Striatum -periaqueductal gray	-0.02319	-0.04641	-0.10767	-0.00588
Midbrain -periaqueductal gray	-0.24334	-0.33983	0.106375	0.177315
Medulla-periaqueductal gray	0	0	-0.36226	0
Cortical amygdalar area -Isocortex	0.050295	-0.16974	-0.14162	0.923635
Olfactory areas -Isocortex	-0.35919	-0.02857	-0.04997	-0.01618
Pons-Isocortex	-0.14677	-0.02458	-0.01941	-0.02906
Midbrain reticular nucleus-Isocortex	0.703702	-0.32274	-0.16739	-0.33453
Nucleus accumbens-Isocortex	-0.19164	-0.02751	-0.43532	-0.01103
fimbria-Ictx	0.120852	-0.03379	-0.01069	-0.81949
Anterior cingulate area-Isocortex	-0.93897	-0.06776	-0.78421	-0.04404
Somatomotor areas-Isocortex	0.143054	-0.88286	0.520029	0.83022
Somatosensory areas-Isocortex	0.026208	-0.38964	-0.81735	-0.99633
piriform area-Isocortex	0.037436	-0.30048	0.666184	0.227518
Taenia tecta -Isocortex	0.822129	-0.90679	-0.21626	-0.21185
Accessory olfactory bulb glomerular layer-Isocortex	0	0	0	0
Accessory olfactory bulb granular layer-Isocortex	0	0	0	0
Retrohippocampal region -Isocortex	-0.78988	-0.01071	-0.01643	0.943926
Entorhinal area-Isocortex	0.418412	-0.00813	-0.10516	0.337853
Field CA1-Isocortex	0.097072	-0.02993	-0.73958	0.095473
Field CA3-Isocortex	0.055208	-0.02482	-0.23112	0.251543
Dentate gyrus-Isocortex	0.086854	-0.05636	0.947619	0.060113
Field CA2 -Isocortex	0.243343	-0.01229	-0.87046	0.156435
Accessory olfactory bulb mitral layer-Isocortex	0	0	0	0
Striatum -Isocortex	-0.23846	-0.01889	-0.25482	-0.0307
Midbrain -Isocortex	-0.02932	-0.03807	-0.05684	-0.04964
Medulla-Isocortex	-0.48246	-0.02751	-0.13435	-0.06925
Olfactory areas -Cortical amygdalar area	0.666448	-0.01049	0	-0.07336
Pons-Cortical amygdalar area	0.001964	0	0	0.337853
Midbrain reticular nucleus-Cortical amygdalar area	0	0	0	0

Nucleus accumbens-Cortical amygdalar area	0	0	0	0
fimbria-Cortical amygdalar area	0.674726	0	0	-0.01768
Anterior cingulate area-Cortical amygdalar area	0	0	0	0
Somatomotor areas-Cortical amygdalar area	0	0	0	-0.27358
Somatosensory areas-Cortical amygdalar area	0.062608	-0.66069	0	-0.70907
piriform area-Cortical amygdalar area	0.021798	0	0	0.271993
Taenia tecta -Cortical amygdalar area	0.123871	-0.52674	0	-0.01898
Accessory olfactory bulb glomerular layer-Cortical amygdalar area	0	0	0	0
Accessory olfactory bulb granular layer-Cortical amygdalar area	0	0	0	0
Retrohippocampal region -Cortical amygdalar area	0.108831	-0.05224	0	0.144158
Entorhinal area-Cortical amygdalar area	0.001964	-0.14498	-0.11081	0.083676
Field CA1-Cortical amygdalar area	0.010369	-0.08429	0	0.177315
Field CA3-Cortical amygdalar area	0.013601	-0.46045	0	0.263669
Dentate gyrus-Cortical amygdalar area	0.016462	-0.91075	-0.72337	0.175768
Field CA2 -Cortical amygdalar area	0.800667	0	0	0.433047
Accessory olfactory bulb mitral layer-Cortical amygdalar area	0	0	0	0
Striatum -Cortical amygdalar area	0.144394	-0.00765	-0.26766	0.634918
Midbrain -Cortical amygdalar area	0.047827	0.384504	0.312395	0.066524
Medulla-Cortical amygdalar area	0	0	0	0.274214
Pons-Olfactory areas	-0.27116	-0.10201	-0.20064	-0.00588
Midbrain reticular nucleus-Olfactory areas	0	0	0	0
Nucleus accumbens-Olfactory areas	0	0	0	-0.16467
fimbria-Olfactory areas	0.567609	-0.94008	0	-0.03368
Anterior cingulate area-Olfactory areas	-0.98451	-0.01424	0	-0.07704
Somatomotor areas-Olfactory areas	-0.83927	-0.71236	-0.91286	-0.02897
Somatosensory areas-Olfactory areas	0.965309	-0.07392	-0.87046	-0.13425
piriform area-Olfactory areas	-0.24334	-0.28934	0.866574	-0.26308
Taenia tecta -Olfactory areas	-0.37559	-0.13406	-0.1091	-0.00519
Accessory olfactory bulb glomerular layer-Olfactory areas	0	0	0	0
Accessory olfactory bulb granular layer-Olfactory areas	0	0	0	0
Retrohippocampal region -Olfactory areas	-0.01048	-0.00767	-0.00787	-0.03877
Entorhinal area-Olfactory areas	-0.14439	-0.02552	-0.01982	-0.00946
Field CA1-Olfactory areas	-0.02409	-0.01149	-0.00344	-0.02897
Field CA3-Olfactory areas	-0.94589	-0.1731	-0.10516	-0.06011
Dentate gyrus-Olfactory areas	-0.1059	-0.0805	-0.11722	-0.12106

Field CA2 -Olfactory areas	-0.04595	-0.00117	-0.01941	-0.02218
Accessory olfactory bulb mitral layer-Olfactory areas	0	0	0	0
Striatum -Olfactory areas	-0.1592	-0.01686	-0.06269	-0.01981
Midbrain -Olfactory areas	-0.02245	-0.01229	-0.03648	-0.00351
Medulla-Olfactory areas	-0.60284	-0.05224	-0.22865	-0.14736
Midbrain reticular nucleus-Pons	0	0	0	0
Nucleus accumbens-Pons	0	0	0	0
fimbria-Pons	0	0	0	0
Anterior cingulate area-Pons	0	0	0	0
Somatomotor areas-Pons	-0.15564	-0.10129	0	-0.04548
Somatosensory areas-Pons	-0.02248	-0.01398	-0.08702	-0.01981
piriform area-Pons	0	0	0	0
Taenia tecta -Pons	0	0	0	-0.13461
Accessory olfactory bulb glomerular layer-Pons	0	0	0	0
Accessory olfactory bulb granular layer-Pons	0	0	0	0
Retrohippocampal region -Pons	-0.96442	-0.01323	-0.04816	-0.27688
Entorhinal area-Pons	-0.45823	-0.07516	-0.01367	-0.02855
Field CA1-Pons	-0.64466	-0.01388	-0.05474	-0.27421
Field CA3-Pons	0.046372	0.83715	-0.42697	0.934421
Dentate gyrus-Pons	0.22392	-0.3073	-0.30969	0.26871
Field CA2 -Pons	-0.11117	-0.00765	0	-0.13946
Accessory olfactory bulb mitral layer-Pons	0	0	0	0
Striatum -Pons	-0.02246	-0.01132	-0.0064	-0.00351
Midbrain -Pons	0.970086	-0.57538	0.01982	0.049997
Medulla-Pons	0	0	0	0
Nucleus accumbens-Midbrain reticular nucleus	0	0	0	0
fimbria-Midbrain reticular nucleus	0	0	0	0
Anterior cingulate area-Midbrain reticular nucleus	0	0	0	0
Somatomotor areas-Midbrain reticular nucleus	0	0	0	0
Somatosensory areas-Midbrain reticular nucleus	0.045953	0	0	0
piriform area-Midbrain reticular nucleus	0	0	0	0
Taenia tecta -Midbrain reticular nucleus	0	0	0	0
Accessory olfactory bulb glomerular layer-Midbrain reticular nucleus	0	0	0	0
Accessory olfactory bulb granular layer-Midbrain reticular nucleus	0	0	0	0

Retrohippocampal region -Midbrain reticular nucleus	-0.17609	0	0	-0.19812
Entorhinal area-Midbrain reticular nucleus	0.965309	0	0	-0.07341
Field CA1-Midbrain reticular nucleus	-0.12604	0	0	-0.37694
Field CA3-Midbrain reticular nucleus	0.089962	-0.80596	-0.52061	0.023414
Dentate gyrus-Midbrain reticular nucleus	-0.94766	-0.57557	-0.78802	0.150259
Field CA2 -Midbrain reticular nucleus	0	0	0	0
Accessory olfactory bulb mitral layer-Midbrain reticular nucleus	0	0	0	0
Striatum -Midbrain reticular nucleus	-0.2715	0	0	0
Midbrain -Midbrain reticular nucleus	-0.22953	0	0	0
Medulla-Midbrain reticular nucleus	0	0	0	0
fimbria-Nucleus accumbens	0	0	0	0
Anterior cingulate area-Nucleus accumbens	0	0	0	0
Somatomotor areas-Nucleus accumbens	0	0	0	0
Somatosensory areas-Nucleus accumbens	0.471199	-0.33678	-0.76862	-0.03246
piriform area-Nucleus accumbens	0	0	0	0
Taenia tecta -Nucleus accumbens	0	0	0	0
Accessory olfactory bulb glomerular layer-Nucleus accumbens	0	0	0	0
Accessory olfactory bulb granular layer-Nucleus accumbens	0	0	0	0
Retrohippocampal region -Nucleus accumbens	-0.02245	-0.20398	0	-0.06415
Entorhinal area-Nucleus accumbens	-0.05234	-0.23658	-0.05435	-0.00519
Field CA1-Nucleus accumbens	-0.0218	-0.05636	-0.10767	-0.0339
Field CA3-Nucleus accumbens	0.495239	-0.33461	-0.31453	-0.04069
Dentate gyrus-Nucleus accumbens	-0.68618	-0.87513	-0.29908	-0.10608
Field CA2 -Nucleus accumbens	-0.17609	0	0	-0.02467
Accessory olfactory bulb mitral layer-Nucleus accumbens	0	0	0	0
Striatum -Nucleus accumbens	-0.12942	-0.38028	0	-0.06796
Midbrain -Nucleus accumbens	0	0	0	-0.09547
Medulla-Nucleus accumbens	0	0	0	0
Anterior cingulate area-fimbria	0	0	0	0
Somatomotor areas-fimbria	0	0	0	0
Somatosensory areas-fimbria	0.025942	-0.22535	0	0.258823
piriform area-fimbria	0.159911	0	0	0.614993
Taenia tecta -fimbria	0.07087	0	0	-0.19735
Accessory olfactory bulb glomerular layer-fimbria	0	0	0	0

Accessory olfactory bulb granular layer-fimbria	0	0	0	0
Retrohippocampal region -fimbria	-0.02582	0	0	-0.01331
Entorhinal area-fimbria	-0.08485	0	0	-0.00302
Field CA1-fimbria	-0.02505	-0.01911	-0.00682	-0.98078
Field CA3-fimbria	0.166341	-0.00811	-0.03425	0.40127
Dentate gyrus-fimbria	0.682152	-0.04387	-0.0611	-0.88081
Field CA2 -fimbria	-0.15059	0	0	-0.27745
Accessory olfactory bulb mitral layer-fimbria	0	0	0	0
Striatum -fimbria	0.657188	-0.0347	-0.42697	-0.28585
Midbrain -fimbria	-0.54413	0	0	-0.08852
Medulla-fimbria	0	0	0	0
Somatomotor areas-Anterior cingulate area	0.007397	0.023767	0.279297	0.611035
Somatosensory areas-Anterior cingulate area	0.098554	0.758976	-0.52812	-0.99215
piriform area-Anterior cingulate area	0	0	0	0
Taenia tecta -Anterior cingulate area	0.478702	-0.34883	-0.72461	-0.32574
Accessory olfactory bulb glomerular layer-Anterior cingulate area	0	0	0	0
Accessory olfactory bulb granular layer-Anterior cingulate area	0	0	0	0
Retrohippocampal region -Anterior cingulate area	-0.04806	0	0	-0.02198
Entorhinal area-Anterior cingulate area	0	0	-0.00873	-0.02845
Field CA1-Anterior cingulate area	-0.09885	0	-0.00549	-0.28388
Field CA3-Anterior cingulate area	-0.48553	0	-0.08492	-0.78438
Dentate gyrus-Anterior cingulate area	-0.02146	-0.03379	-0.0129	-0.36003
Field CA2 -Anterior cingulate area	0.016436	0.140846	0.062261	0.309909
Accessory olfactory bulb mitral layer-Anterior cingulate area	0	0	0	0
Striatum -Anterior cingulate area	-0.04806	-0.08448	-0.10134	-0.14487
Midbrain -Anterior cingulate area	0	0	-0.01069	-0.03246
Medulla-Anterior cingulate area	0	0	0	0
Somatosensory areas-Somatomotor areas	0.701203	-0.42695	0.153722	0.533331
piriform area-Somatomotor areas	0	0	0.314527	0.874379
Taenia tecta -Somatomotor areas	0.229531	0.171757	0.541092	0
Accessory olfactory bulb glomerular layer-Somatomotor areas	0	0	0	0
Accessory olfactory bulb granular layer-Somatomotor areas	0	0	0	0
Retrohippocampal region -Somatomotor areas	-0.02505	0	-0.29207	-0.01631
Entorhinal area-Somatomotor areas	-0.07087	0	-0.2178	-0.11272

Field CA1-Somatomotor areas	-0.1158	-0.42251	-0.39232	-0.69499
Field CA3-Somatomotor areas	0.941209	-0.53759	-0.66618	-0.89384
Dentate gyrus-Somatomotor areas	-0.09631	-0.42251	-0.89178	-0.79879
Field CA2 -Somatomotor areas	0.019635	-0.95351	0.028597	-0.89919
Accessory olfactory bulb mitral layer-Somatomotor areas	0	0	0	0
Striatum -Somatomotor areas	-0.18029	-0.21795	-0.71374	-0.66581
Midbrain -Somatomotor areas	-0.03114	-0.06571	-0.2007	-0.02158
Medulla-Somatomotor areas	0	0	-0.25041	-0.08636
piriform area-Somatosensory areas	0.158422	0.953546	0.908183	0.200622
Taenia tecta -Somatosensory areas	0.490055	0.435006	-0.75007	-0.54942
Accessory olfactory bulb glomerular layer-Somatosensory areas	0	0	0	0
Accessory olfactory bulb granular layer-Somatosensory areas	0	0	0	0
Retrohippocampal region -Somatosensory areas	0.830305	-0.03592	-0.05435	-0.11167
Entorhinal area-Somatosensory areas	-0.35572	-0.00833	-0.13193	-0.06626
Field CA1-Somatosensory areas	0.037416	-0.02857	0.157807	0.665999
Field CA3--Somatosensory areas	0.014013	-0.05766	-0.52812	0.860114
Dentate gyrus-Somatosensory areas	0.093617	-0.12107	0.426967	-0.86326
Field CA2 -Somatosensory areas	0.045953	-0.13588	0.220744	-0.77921
Accessory olfactory bulb mitral layer-Somatosensory areas	0	0	0	0
Striatum --Somatosensory areas	-0.7286	-0.15279	-0.3258	-0.74689
Midbrain --Somatosensory areas	-0.20783	-0.00767	-0.05903	-0.05479
Medulla-Somatosensory areas	-0.01123	0	-0.05903	-0.03246
Taenia tecta -piriform area	-0.19913	0	-0.57892	-0.84389
Accessory olfactory bulb glomerular layer-piriform area	0	0	0	0
Accessory olfactory bulb granular layer-piriform area	0	0	0	0
Retrohippocampal region -piriform area	-0.16855	0	-0.20064	0.821952
Entorhinal area-piriform area	-0.97198	-0.95355	-0.10134	0.640045
Field CA1-piriform area	0.056602	-0.90679	-0.05684	0.268638
Field CA3-piriform area	0.181279	-0.91075	-0.2611	0.156435
Dentate gyrus-piriform area	-0.22874	-0.85604	-0.16077	0.122265
Field CA2 -piriform area	0.598648	0	-0.08309	0.160479
Accessory olfactory bulb mitral layer-piriform area	0	0	0	0
Striatum -piriform area	-0.48553	-0.77131	-0.2793	0.732633
Midbrain -piriform area	-0.07087	-0.7432	0	-0.88414

Medulla-piriform area	0	0	0	0
Accessory olfactory bulb glomerular layer-Taenia tecta	0	0	0	0
Accessory olfactory bulb granular layer-Taenia tecta	0	0	0	0
Retrohippocampal region -Taenia tecta	-0.04211	-0.00813	-0.03566	-0.02317
Entorhinal area-Taenia tecta	-0.02463	-0.00813	-0.05501	-0.00739
Field CA1-Taenia tecta	-0.03191	-0.00816	-0.13622	-0.08352
Field CA3-Taenia tecta	0.271504	-0.18365	-0.58085	-0.24564
Dentate gyrus-Taenia tecta	-0.95701	-0.59136	0.936276	-0.48463
Field CA2 -Taenia tecta	-0.13709	-0.0103	-0.11703	-0.14178
Accessory olfactory bulb mitral layer-Taenia tecta	0	0	0	0
Striatum -Taenia tecta	-0.84535	-0.02751	-0.13622	-0.02158
Midbrain -Taenia tecta	-0.16249	-0.01363	-0.10008	-0.03774
Medulla-Taenia tecta	0	0	0	0
Accessory olfactory bulb granular layer-Accessory olfactory bulb glomerular layer	0	0	0	0
Retrohippocampal region -Accessory olfactory bulb glomerular layer	0	0	0	0
Entorhinal area-Accessory olfactory bulb glomerular layer	0	0	0	0
Field CA1-Accessory olfactory bulb glomerular layer	0	0	0	0
Field CA3-Accessory olfactory bulb glomerular layer	0	0	0	0
Dentate gyrus-Accessory olfactory bulb glomerular layer	0	0	0	0
Field CA2 -Accessory olfactory bulb glomerular layer	0	0	0	0
Accessory olfactory bulb mitral layer-Accessory olfactory bulb glomerular layer	0	0	0	0
Striatum -Accessory olfactory bulb glomerular layer	0	0	0	0
Midbrain -Accessory olfactory bulb glomerular layer	0	0	0	0
Medulla-Accessory olfactory bulb glomerular layer	0	0	0	0
Retrohippocampal region -Accessory olfactory bulb granular layer	0	0	0	0
Entorhinal area-Accessory olfactory bulb granular layer	0	0	0	0
Field CA1-Accessory olfactory bulb granular layer	0	0	0	0
Field CA3-Accessory olfactory bulb granular layer	0	0	0	0
Dentate gyrus-Accessory olfactory bulb granular layer	0	0	0	0
Field CA2 -Accessory olfactory bulb granular layer	0	0	0	0
Accessory olfactory bulb mitral layer-Accessory olfactory bulb granular layer	0	0	0	0
Striatum -Accessory olfactory bulb granular layer	0	0	0	0

Midbrain -Accessory olfactory bulb granular layer	0	0	0	0
Medulla-Accessory olfactory bulb granular layer	0	0	0	0
Entorhinal area-Retrohippocampal region	-0.41403	-0.01477	-0.00621	-0.70236
Field CA1-Retrohippocampal region	-0.40509	-0.00767	-0.0125	-0.08872
Field CA3-Retrohippocampal region	-0.01971	-0.05224	-0.02442	-0.01331
Dentate gyrus-Retrohippocampal region	-0.41841	-0.04496	-0.04474	-0.0361
Field CA2 -Retrohippocampal region	-0.01892	-0.00813	-0.01367	-0.10876
Accessory olfactory bulb mitral layer-Retrohippocampal region	0	0	0	0
Striatum -Retrohippocampal region	-0.01074	-0.00767	-0.00729	-0.0322
Midbrain -Retrohippocampal region	-0.02136	-0.03586	-0.09781	-0.01331
Medulla-Retrohippocampal region	0.80617	-0.16254	-0.23507	-0.48351
Field CA1-Entorhinal area	0.392937	-0.00813	-0.00677	0.561039
Field CA3-Entorhinal area	-0.36219	-0.00813	-0.0064	-0.86011
Dentate gyrus-Entorhinal area	0.916069	-0.03379	-0.0064	0.073411
Field CA2 -Entorhinal area	-0.11345	-0.00767	-0.01982	-0.88414
Accessory olfactory bulb mitral layer-Entorhinal area	0	0	0	0
Striatum -Entorhinal area	-0.06396	-0.00776	-0.0093	-0.10912
Midbrain -Entorhinal area	-0.01123	-0.03792	-0.06686	-0.08916
Medulla-Entorhinal area	-0.50937	-0.10201	-0.33455	-0.53333
Field CA3-Field CA1	-0.18029	-0.07171	-0.03749	0.899192
Dentate gyrus-Field CA1	-0.39392	-0.98358	-0.30724	-0.25725
Field CA2 -Field CA1	0.971982	-0.02751	-0.15349	0.521246
Accessory olfactory bulb mitral layer-Field CA1	0	0	0	0
Striatum -Field CA1	-0.0043	-0.00767	-0.00682	-0.05502
Midbrain -Field CA1	-0.02594	-0.03703	-0.12646	-0.03368
Medulla-Field CA1	-0.69326	-0.21819	-0.31733	-0.19189
Dentate gyrus-Field CA3	0.828032	-0.19732	-0.1659	-0.9086
Field CA2 -Field CA3	-0.20489	-0.0144	-0.01849	-0.92627
Accessory olfactory bulb mitral layer-Field CA3	0	0	0	0
Striatum -Field CA3	-0.09312	-0.01027	-0.01982	-0.37694
Midbrain -Field CA3	0.857821	-0.3845	0.683063	0.325124
Medulla-Field CA3	0.029316	-0.14368	0.405017	-0.46804

Field CA2 -Dentate gyrus	-0.75456	-0.02835	-0.18185	-0.30792
Accessory olfactory bulb mitral layer-Dentate gyrus	0	0	0	0
Striatum -Dentate gyrus	-0.0668	-0.02903	-0.0611	-0.8135
Midbrain -Dentate gyrus	-0.22874	-0.52624	-0.8371	0.95293
Medulla-Dentate gyrus	0.105238	-0.21521	0.647097	0.67737
Accessory olfactory bulb mitral layer-Field CA2	0	0	0	0
Striatum -Field CA2	-0.00522	-0.00767	-0.01982	-0.06011
Midbrain -Field CA2	-0.0332	-0.02751	-0.01283	-0.24573
Medulla-Field CA2	0	0	-0.09022	-0.08852
Striatum -Accessory olfactory bulb mitral layer	0	0	0	0
Midbrain -Accessory olfactory bulb mitral layer	0	0	0	0
Medulla-Accessory olfactory bulb mitral layer	0	0	0	0
Midbrain -Striatum	-0.01511	-0.00813	-0.01941	-0.043
Medulla-Striatum	-0.00985	-0.01229	-0.01367	-0.03142
Medulla-Midbrain	-0.68341	-0.76123	-0.17989	0.211849

S9. Adjust p values for group comparisons of pair-wised connectivity for Thalamus.

Region Connections	Thalamus			
	Left Ipsilateral	Left Contralateral	Right Contralateral	Right Ipsilateral
Caudoputamen-corpus callosum	-0.01201	-0.04366	-0.02357	0.265413
anterior commissure olfactory limb-corpus callosum	0	0	0	0
pallidum -corpus callosum	-0.16065	-0.08913	-0.07771	-0.20803
internal capsule-corpus callosum	0.392045	-0.322	-0.24367	-0.4013
Thalamus-corpus callosum	-0.00905	-0.322	-0.03988	-0.08416
Cerebellum-corpus callosum	-0.07602	-0.04136	-0.03484	-0.09786
Superior colliculus-corpus callosum	-0.02755	-0.40876	-0.03271	-0.03313
ventricular systems-corpus callosum	-0.03145	-0.12672	-0.03714	-0.35434
Hypothalamus -corpus callosum	-0.04055	-0.02538	-0.05227	-0.03338
Inferior colliculus -corpus callosum	-0.00416	-0.0606	-0.04967	-0.01061
periaqueductal gray-corpus callosum	-0.00168	-0.01844	-0.00878	-0.00885
Isocortex -corpus callosum	-0.00545	-0.12628	-0.01335	-0.20053
Cortical amygdalar area -corpus callosum	0.763793	0	0	-0.8759
Olfactory areas -corpus callosum	-0.56003	-0.20394	-0.15331	-0.87286
Pons-corpus callosum	-0.00198	-0.00918	-0.00221	-0.00548
Midbrain reticular nucleus-corpus callosum	-0.00355	-0.04384	-0.06049	-0.01819
Nucleus accumbens-corpus callosum	0.53227	0	-0.03057	0
fimbria-corpus callosum	0.697278	0	-0.14419	-0.93491
Anterior cingulate area-corpus callosum	0	0	0	-0.05405
Somatomotor areas-corpus callosum	-0.09704	-0.57474	-0.05027	-0.47576
Somatosensory areas-corpus callosum	-0.14522	-0.32463	-0.04049	-0.20772
piriform area-corpus callosum	0	0	0	0.441483
Taenia tecta -corpus callosum	0.116989	0.513602	-0.15331	-0.52234
Accessory olfactory bulb glomerular layer-corpus callosum	0	0	0	0
Accessory olfactory bulb granular layer-corpus callosum	0	0	0	0
Retrohippocampal region -corpus callosum	-0.00833	-0.01844	-0.01938	-0.00548
Entorhinal area-corpus callosum	-0.02711	-0.05096	-0.00746	-0.07072
Field CA1-corpus callosum	-0.01746	-0.02538	-0.01992	-0.01472
Field CA3-corpus callosum	0.198407	-0.04758	-0.03714	-0.764
Dentate gyrus-corpus callosum	-0.60265	-0.12715	-0.02592	-0.55845
Field CA2 -corpus callosum	-0.07726	-0.17364	-0.02592	-0.11971
Accessory olfactory bulb mitral layer-corpus callosum	0	0	0	0

Striatum -corpus callosum	-0.02996	-0.08972	-0.05811	0.435246
Midbrain -corpus callosum	-0.00198	-0.01163	-0.00746	-0.00548
Medulla-corpus callosum	-0.00242	-0.02538	-0.01335	-0.01962
anterior commissure olfactory limb-Caudoputamen	0	0	0	0
pallidum -Caudoputamen	0.462316	-0.025379	-0.130138	-0.726345
internal capsule-Caudoputamen	0.064128	-0.048534	-0.103423	0.365581
Thalamus-Caudoputamen	0.233515	-0.409211	-0.013351	0.570509
Cerebellum-Caudoputamen	-0.172882	-0.017426	-0.076885	-0.164586
Superior colliculus-Caudoputamen	-0.13209	-0.090298	-0.053995	-0.066172
ventricular systems-Caudoputamen	0.9871	-0.281664	-0.308782	0.338159
Hypothalamus -Caudoputamen	-0.838256	-0.013111	-0.764969	-0.067105
Inferior colliculus -Caudoputamen	-0.003971	-0.013111	-0.079412	-0.05386
periaqueductal gray-Caudoputamen	-0.019129	-0.011633	-0.008782	-0.006659
Isocortex -Caudoputamen	-0.089245	-0.087715	-0.011296	-0.764004
Cortical amygdalar area -Caudoputamen	0.265485	0	-0.870566	0.654417
Olfactory areas -Caudoputamen	0.131729	0.958977	-0.981427	0.145561
Pons-Caudoputamen	-0.001679	-0.011633	-0.002208	-0.000459
Midbrain reticular nucleus-Caudoputamen	-0.031053	-0.014447	-0.013351	-0.018107
Nucleus accumbens-Caudoputamen	0.096323	0.664906	-0.671313	-0.839338
fimbria-Caudoputamen	0.013653	-0.032217	-0.173842	0.145561
Anterior cingulate area-Caudoputamen	-0.024206	-0.012819	-0.059114	0.86131
Somatomotor areas-Caudoputamen	-0.163404	-0.529969	-0.062731	-0.704425
Somatosensory areas-Caudoputamen	0.36878	-0.814435	-0.060489	-0.709602
piriform area-Caudoputamen	-0.473738	0.816217	0.631704	0.70592
Taenia tecta -Caudoputamen	0.004199	0.044723	0.399624	0.273575
Accessory olfactory bulb glomerular layer-Caudoputamen	0	0	0	0
Accessory olfactory bulb granular layer-Caudoputamen	0	0	0	0
Retrohippocampal region -Caudoputamen	-0.029963	-0.011633	-0.01193	-0.018107
Entorhinal area-Caudoputamen	-0.171815	-0.013111	-0.01576	-0.03313
Field CA1-Caudoputamen	-0.403146	-0.160504	-0.01381	-0.337483
Field CA3-Caudoputamen	0.003602	-0.04398	-0.101897	0.018994
Dentate gyrus-Caudoputamen	0.054582	-0.142733	-0.293432	0.014722
Field CA2 -Caudoputamen	-0.44523	-0.813283	-0.076885	-0.385799
Accessory olfactory bulb mitral layer-Caudoputamen	0	0	0	0
Striatum -Caudoputamen	0.382833	-0.07414	-0.236811	0.154929
Midbrain -Caudoputamen	-0.017379	-0.009621	-0.013351	-0.004054

Medulla-Caudoputamen	-0.00378	-0.011633	-0.009558	-0.007118
pallidum -anterior commissure olfactory limb	0	0	0	0
internal capsule-anterior commissure olfactory limb	0	0	0	0
Thalamus-anterior commissure olfactory limb	0.091958	0.028947	0	0.01941
Cerebellum-anterior commissure olfactory limb	0	0	0	0
Superior colliculus-anterior commissure olfactory limb	0	0	0	0
ventricular systems-anterior commissure olfactory limb	0.782681	0	0.529344	-0.533981
Hypothalamus -anterior commissure olfactory limb	0.372068	0	0	0
Inferior colliculus -anterior commissure olfactory limb	0	0	0	0
periaqueductal gray-anterior commissure olfactory limb	0	0	-0.34969	0
Isocortex -anterior commissure olfactory limb	0	0	0	0
Cortical amygdalar area -anterior commissure olfactory limb	0	0	0	0
Olfactory areas -anterior commissure olfactory limb	0	0	0	0
Pons-anterior commissure olfactory limb	0	0	0	0
Midbrain reticular nucleus-anterior commissure olfactory limb	0	0	0	0
Nucleus accumbens-anterior commissure olfactory limb	0	0	0	0
fimbria-anterior commissure olfactory limb	0	0	0	0
Anterior cingulate area-anterior commissure olfactory limb	0	0	0	0
Somatomotor areas-anterior commissure olfactory limb	0	0	0	0
Somatosensory areas-anterior commissure olfactory limb	0	0	0	0
piriform area-anterior commissure olfactory limb	0	0	0	0
Taenia tecta -anterior commissure olfactory limb	0	0	0	0
Accessory olfactory bulb glomerular layer-anterior commissure olfactory limb	0	0	0	0
Accessory olfactory bulb granular layer-anterior commissure olfactory limb	0	0	0	0
Retrohippocampal region -anterior commissure olfactory limb	0	0	0	0
Entorhinal area-anterior commissure olfactory limb	0	0	0	0
Field CA1-anterior commissure olfactory limb	0	0	0	0
Field CA3-anterior commissure olfactory limb	0.925011	0	0	0
Dentate gyrus-anterior commissure olfactory limb	0.366928	0	0	0.721266
Field CA2 -anterior commissure olfactory limb	0	0	0	0
Accessory olfactory bulb mitral layer-anterior commissure olfactory limb	0	0	0	0
Striatum -anterior commissure olfactory limb	0	0	0	0
Midbrain -anterior commissure olfactory limb	0	0	0	0
Medulla-anterior commissure olfactory limb	0	0	0	0

internal capsule-pallidum	0.066378	-0.324625	-0.562774	0.328442
Thalamus-pallidum	0.63858	0.292082	-0.339303	0.12608
Cerebellum-pallidum	-0.316398	-0.069543	-0.103779	-0.034706
Superior colliculus-pallidum	-0.13148	0.940223	-0.142224	-0.05228
ventricular systems-pallidum	-0.800176	0.318348	-0.196087	0.260769
Hypothalamus -pallidum	-0.545533	-0.025379	-0.444454	-0.265413
Inferior colliculus -pallidum	-0.014262	-0.045328	-0.567457	-0.032465
periaqueductal gray-pallidum	-0.017379	-0.046848	-0.023569	-0.011299
Isocortex -pallidum	-0.281911	-0.215303	-0.042542	-0.054054
Cortical amygdalar area -pallidum	0.228443	0	0	-0.532327
Olfactory areas -pallidum	0.762367	-0.928122	-0.589602	-0.478972
Pons-pallidum	-0.005682	-0.016361	-0.009558	-0.000023
Midbrain reticular nucleus-pallidum	-0.00841	-0.136444	-0.079522	-0.008784
Nucleus accumbens-pallidum	0.029963	0	0	-0.490594
fimbria-pallidum	0.947707	0.922997	0	-0.020028
AVA-pallidum	-0.096235	0	0	0
Somatomotor areas-pallidum	0	-0.660772	0	-0.506607
Somatosensory areas-pallidum	0.163404	0.826095	-0.567457	0.922808
piriform area-pallidum	0.660328	-0.468127	0	-0.827337
Taenia tecta -pallidum	0.017379	0.209012	0.794936	0.654397
Accessory olfactory bulb glomerular layer-pallidum	0	0	0	0
Accessory olfactory bulb granular layer-pallidum	0	0	0	0
Retrohippocampal region -pallidum	-0.096235	-0.055079	-0.042211	-0.01673
Entorhinal area-pallidum	-0.024206	-0.045328	-0.052466	-0.005539
Field CA1-pallidum	-0.194014	-0.025379	-0.036026	-0.026606
Field CA3-pallidum	0.75867	-0.088886	-0.033849	-0.118319
Dentate gyrus-pallidum	0.015377	-0.626056	-0.067978	0.133624
Field CA2 -pallidum	-0.028021	-0.135371	-0.040803	-0.023822
Accessory olfactory bulb mitral layer-pallidum	0	0	0	0
Striatum -pallidum	0.88664	-0.235197	-0.250693	-0.359897
Midbrain -pallidum	-0.004199	-0.014447	-0.021051	-0.010612
Medulla-pallidum	-0.03582	-0.019207	-0.152089	-0.040151
Thalamus-internal capsule	-0.757388	0.883796	-0.30135	0.02985
Cerebellum-internal capsule	0.017379	0.480721	-0.649973	0.090386
Superior colliculus-internal capsule	-0.499668	0.254502	-0.582099	-0.294468

ventricular systems-internal capsule	0.485411	0.510953	-0.157312	-0.552905
Hypothalamus -internal capsule	-0.072405	-0.519492	0.152398	0.095544
Inferior colliculus -internal capsule	-0.004156	0	0.532587	-0.49749
periaqueductal gray-internal capsule	-0.007629	-0.972704	-0.216635	-0.032437
Isocortex -internal capsule	-0.387775	-0.822202	-0.405041	-0.506607
Cortical amygdalar area -internal capsule	0	0	0	0
Olfactory areas -internal capsule	0	0	0	0.570509
Pons-internal capsule	-0.00378	0	0	-0.007182
Midbrain reticular nucleus-internal capsule	-0.031446	0	0	-0.433956
Nucleus accumbens-internal capsule	0	0	0	0
fimbria-internal capsule	0	0	0	0.670233
Anterior cingulate area-internal capsule	0	0	0	0
Somatomotor areas-internal capsule	0	0	0	-0.060784
Somatosensory areas-internal capsule	0.029081	0.871578	0	-0.764393
piriform area-internal capsule	0	0	0	0
Taenia tecta -internal capsule	0.09491	0	0	0
Accessory olfactory bulb glomerular layer-internal capsule	0	0	0	0
Accessory olfactory bulb granular layer-internal capsule	0	0	0	0
Retrohippocampal region -internal capsule	-0.904805	0	0	-0.132916
Entorhinal area-internal capsule	0.924745	0	0	0.570509
Field CA1-internal capsule	0.157174	0	0	-0.874949
Field CA3-internal capsule	0.003602	0	-0.532587	0.005479
Dentate gyrus-internal capsule	0.253288	0	-0.838038	0.003287
Field CA2 -internal capsule	0.226248	0	0	0
Accessory olfactory bulb mitral layer-internal capsule	0	0	0	0
Striatum -internal capsule	0.142905	-0.879213	-0.911562	0.145561
Midbrain --internal capsule	-0.009052	-0.814435	-0.181154	-0.102531
Medulla-internal capsule	-0.021958	0	-0.064606	0
Cerebellum-Thalamus	0.007531	0.925903	0.216635	0.014127
Superior colliculus-Thalamus	0.17753	0.822892	-0.63004	-0.67023
ventricular systems-Thalamus	0.766837	-0.38322	-0.07168	-0.31071
Hypothalamus -Thalamus	0.017455	-0.2967	0.019612	0.024207
Inferior colliculus -Thalamus	-0.02421	-0.17437	0.927199	0.984499
periaqueductal gray-Thalamus	0.285462	-0.82151	-0.15322	-0.01819
Isocortex -Thalamus	-0.1005	-0.21822	-0.15331	-0.08652

Cortical amygdalar area -Thalamus	0.069498	0.370605	0.547019	0.338159
Olfactory areas -Thalamus	0.042368	0.872113	0.040803	0.802643
Pons-Thalamus	-0.04541	-0.03885	-0.02048	-0.00807
Midbrain reticular nucleus-Thalamus	-0.66158	-0.32475	-0.54584	-0.09554
Nucleus accumbens-Thalamus	0.013653	0.839611	0.079412	0.310713
fimbria-Thalamus	0.346952	-0.20332	-0.02458	-0.68493
Anterior cingulate area-Thalamus	-0.04237	-0.06646	-0.00813	-0.02802
Somatomotor areas-Thalamus	-0.13024	-0.11208	-0.26586	-0.15169
Somatosensory areas-Thalamus	0.184499	-0.44024	-0.38767	-0.02985
piriform area-Thalamus	-0.89279	-0.93002	0.309065	0.337143
Taenia tecta -Thalamus	0.001679	0.308778	0.038518	0.196306
Accessory olfactory bulb glomerular layer-Thalamus	0	0	0	0
Accessory olfactory bulb granular layer-Thalamus	0	0	0	0
Retrohippocampal region -Thalamus	-0.44523	-0.42894	-0.22897	-0.02332
Entorhinal area-Thalamus	-0.02462	-0.02538	-0.03484	-0.08082
Field CA1-Thalamus	-0.08687	-0.4352	0.630037	-0.02601
Field CA3-Thalamus	0.172969	-0.39874	-0.34592	0.792217
Dentate gyrus-Thalamus	0.604477	-0.5705	0.190967	0.124372
Field CA2 -Thalamus	-0.0191	-0.66942	-0.94666	-0.11801
Accessory olfactory bulb mitral layer-Thalamus	0	0	0	0
Striatum -Thalamus	0.009951	-0.21132	0.111397	0.00807
Midbrain -Thalamus	-0.2332	-0.2967	-0.58593	-0.02037
Medulla-Thalamus	-0.00877	-0.01163	-0.03852	-0.02462
Superior colliculus-Cerebellum	0.019322	0.013111	0.840197	0.522344
ventricular systems-Cerebellum	0.322093	0.891241	-0.7047	0.853841
Hypothalamus -Cerebellum	0.004527	0.93658	0.052266	0.0535
Inferior colliculus -Cerebellum	-0.63496	-0.651	0.684524	0.041455
periaqueductal gray-Cerebellum	0.288593	0.606243	-0.68475	0.710001
Isocortex -Cerebellum	-0.7259	-0.51464	-0.34592	-0.40642
Cortical amygdalar area -Cerebellum	0.007629	0	0.376834	0.041455
Olfactory areas -Cerebellum	-0.44257	-0.20394	-0.26186	-0.54678
Pons-Cerebellum	0.826945	0.60265	-0.78729	-0.83335
Midbrain reticular nucleus-Cerebellum	0.073346	0.257392	0	0.014349
Nucleus accumbens-Cerebellum	-0.35448	0	0	0
fimbria-Cerebellum	0	-0.1605	0	-0.01819

Anterior cingulate area-Cerebellum	-0.00841	-0.0566	-0.03164	-0.06398
Somatomotor areas-Cerebellum	-0.02802	-0.17437	-0.1019	-0.06387
Somatosensory areas-Cerebellum	0.844425	-0.49725	-0.16237	-0.01811
piriform area-Cerebellum	0.358278	-0.96492	0.347878	0.275395
Taenia tecta -Cerebellum	0.63858	0.42952	-0.15322	-0.2754
Accessory olfactory bulb glomerular layer-Cerebellum	0	0	0	0
Accessory olfactory bulb granular layer-Cerebellum	0	0	0	0
Retrohippocampal region -Cerebellum	0.872861	-0.27498	-0.34523	0.833699
Entorhinal area-Cerebellum	-0.42818	-0.34699	-0.47235	0.17098
Field CA1-Cerebellum	0.462316	-0.81444	-0.35974	0.170742
Field CA3-Cerebellum	0.004199	0.595574	-0.52429	0.00626
Dentate gyrus-Cerebellum	0.002415	0.602966	0.383485	0.00626
Field CA2 -Cerebellum	-0.53227	-0.07748	0	-0.92281
Accessory olfactory bulb mitral layer-Cerebellum	0	0	0	0
Striatum -Cerebellum	0.228443	-0.47775	-0.2621	-0.75621
Midbrain -Cerebellum	0.040551	0.390895	0.799676	0.050346
Medulla-Cerebellum	-0.54333	-0.96823	-0.58593	0.37967
ventricular systems-Superior colliculus	0.09053	0.958977	0.059114	-0.51445
Hypothalamus -Superior colliculus	0.157159	-0.67894	0.009558	-0.70281
Inferior colliculus -Superior colliculus	-0.10602	-0.39442	0.072426	-0.33714
periaqueductal gray-Superior colliculus	0.421095	0.958319	0.999178	-0.1092
Isocortex -Superior colliculus	-0.94569	-0.21065	0.911562	-0.20415
Cortical amygdalar area -Superior colliculus	0.209661	-0.68287	0.334889	0.984499
Olfactory areas -Superior colliculus	-0.62695	-0.25014	-0.21664	-0.04396
Pons-Superior colliculus	-0.37352	-0.3193	-0.19403	-0.02137
Midbrain reticular nucleus-Superior colliculus	-0.73198	-0.4459	0.033849	-0.05405
Nucleus accumbens-Superior colliculus	0.889715	0	-0.13166	-0.33748
fimbria-Superior colliculus	-0.90032	-0.58103	0	-0.00798
Anterior cingulate area-Superior colliculus	-0.01319	-0.02039	-0.03385	-0.05435
Somatomotor areas-Superior colliculus	-0.05672	-0.21156	-0.18538	-0.04729
Somatosensory areas-Superior colliculus	0.441165	-0.30187	-0.04794	-0.05435
piriform area-Superior colliculus	-0.63858	-0.2333	0.134867	0.745329
Taenia tecta -Superior colliculus	0.064128	-0.48757	-0.54702	-0.09554
Accessory olfactory bulb glomerular layer-Superior colliculus	0	0	0	0
Accessory olfactory bulb granular layer-Superior colliculus	0	0	0	0

Retrohippocampal region -Superior colliculus	-0.0586	-0.0885	0.30135	-0.03885
Entorhinal area-Superior colliculus	-0.13173	-0.32463	0.981427	-0.15169
Field CA1-Superior colliculus	-0.75739	-0.17278	0.7047	-0.1638
Field CA3-Superior colliculus	-0.83826	-0.50674	-0.80784	-0.28966
Dentate gyrus-Superior colliculus	0.326872	-0.45786	0.062875	-0.5552
Field CA2 -Superior colliculus	0.63858	-0.27498	0.369078	-0.31679
Accessory olfactory bulb mitral layer-Superior colliculus	0	0	0	0
Striatum -Superior colliculus	-0.11357	-0.06954	0.52429	-0.16247
Midbrain -Superior colliculus	-0.27385	-0.36724	0.076885	-0.08082
Medulla-Superior colliculus	-0.07335	-0.06354	-0.50971	-0.40642
Hypothalamus -ventricular systems	-0.04541	-0.048534	-0.099501	-0.008784
Inferior colliculus -ventricular systems	-0.027728	-0.04729	-0.30135	-0.730552
periaqueductal gray-ventricular systems	-0.543331	-0.377082	-0.131756	-0.01819
Isocortex -ventricular systems	-0.24018	-0.221777	-0.059114	-0.130886
Cortical amygdalar area -ventricular systems	0.307471	-0.678936	-0.910319	-0.985335
Olfactory areas -ventricular systems	0.871763	-0.75988	-0.726344	0.956108
Pons-ventricular systems	-0.028745	-0.024309	-0.01193	-0.01819
Midbrain reticular nucleus-ventricular systems	-0.872688	-0.439627	-0.22352	-0.014127
Nucleus accumbens-ventricular systems	0.525415	-0.459115	0.531301	0.651041
fimbria-ventricular systems	-0.727845	-0.025379	-0.23775	0.878895
Anterior cingulate area-ventricular systems	-0.013099	-0.013111	-0.01193	-0.023446
Somatomotor areas-ventricular systems	-0.160647	-0.263235	-0.189933	-0.408592
Somatosensory areas-ventricular systems	0.731975	-0.735735	-0.163029	-0.130422
piriform area-ventricular systems	-0.361098	-0.54941	0.365789	0.66001
Taenia tecta -ventricular systems	0.007629	0.025379	0.189933	0.761829
Accessory olfactory bulb glomerular layer-ventricular systems	0	0	0	0
Accessory olfactory bulb granular layer-ventricular systems	0	0	0	0
Retrohippocampal region -ventricular systems	-0.008343	-0.03761	-0.023569	-0.01819
Entorhinal area-ventricular systems	-0.018223	-0.025379	-0.062731	-0.11023
Field CA1-ventricular systems	-0.049982	-0.045328	-0.030076	-0.014127
Field CA3-ventricular systems	-0.194014	-0.036831	-0.10851	-0.208033
Dentate gyrus-ventricular systems	0.841871	-0.274976	-0.238487	-0.337143
Field CA2 -ventricular systems	-0.03543	-0.025379	-0.034839	-0.207718
Accessory olfactory bulb mitral layer-ventricular systems	0	0	0	0
Striatum -ventricular systems	-0.394809	-0.063539	0.387674	0.307831

Midbrain -ventricular systems	-0.108408	-0.014447	-0.052266	-0.00807
Medulla-ventricular systems	-0.017379	-0.013111	-0.059114	-0.03313
Inferior colliculus -Hypothalamus	-0.056721	-0.76064	-0.73001	-0.557039
periaqueductal gray-Hypothalamus	-0.119094	0.370504	-0.096413	-0.142444
Isocortex -Hypothalamus	-0.077227	-0.588757	-0.040803	-0.027926
Cortical amygdalar area -Hypothalamus	0.04171	0.02089	-0.142224	0.258161
Olfactory areas -Hypothalamus	-0.568265	0.67464	-0.060489	-0.836554
Pons-Hypothalamus	-0.023083	-0.112084	-0.014516	-0.01819
Midbrain reticular nucleus-Hypothalamus	-0.998895	0.039683	-0.345916	0.196306
Nucleus accumbens-Hypothalamus	0.432309	0.18433	-0.474094	-0.520063
fimbria-Hypothalamus	-0.435931	-0.411888	-0.018688	-0.003673
Anterior cingulate area-Hypothalamus	-0.007629	-0.130216	-0.009558	-0.01819
Somatomotor areas-Hypothalamus	-0.012817	-0.327421	-0.073752	-0.063871
Somatosensory areas-Hypothalamus	0.296188	0.553287	-0.059695	-0.052738
piriform area-Hypothalamus	0.869265	0.558159	0.760722	0.316787
Taenia tecta -Hypothalamus	0.166817	0.014283	0.7047	0.530581
Accessory olfactory bulb glomerular layer-Hypothalamus	0	0	0	0
Accessory olfactory bulb granular layer-Hypothalamus	0	0	0	0
Retrohippocampal region -Hypothalamus	-0.766212	-0.729439	-0.038518	-0.021231
Entorhinal area-Hypothalamus	-0.403146	-0.581608	-0.013351	-0.026009
Field CA1-Hypothalamus	-0.075357	-0.364227	-0.045059	-0.042276
Field CA3-Hypothalamus	0.6761	0.459115	-0.079412	-0.063871
Dentate gyrus-Hypothalamus	0.501655	0.665434	-0.228868	-0.667986
Field CA2 -Hypothalamus	-0.126181	-0.529457	-0.1229	-0.027554
Accessory olfactory bulb mitral layer-Hypothalamus	0	0	0	0
Striatum -Hypothalamus	-0.092028	-0.983107	-0.113219	0.788454
Midbrain -Hypothalamus	-0.216608	0.141091	-0.158114	-0.783288
Medulla-Hypothalamus	-0.019129	-0.042156	-0.030076	-0.086125
periaqueductal gray-Inferior colliculus	-0.27378	-0.98311	-0.22949	-0.08295
Isocortex -Inferior colliculus	-0.05837	-0.52946	-0.09704	-0.19375
Cortical amygdalar area -Inferior colliculus	0	0	0	0.060537
Olfactory areas -Inferior colliculus	-0.08687	0	0	-0.30082
Pons-Inferior colliculus	-0.37255	-0.26978	0	-0.09786
Midbrain reticular nucleus-Inferior colliculus	-0.23737	0	0	-0.31792

Nucleus accumbens-Inferior colliculus	0	0	0	0
fimbria-Inferior colliculus	0	0	0	0
Anterior cingulate area-Inferior colliculus	0	0	0	0
Somatomotor areas-Inferior colliculus	-0.02755	-0.31459	-0.11692	-0.04461
Somatosensory areas-Inferior colliculus	-0.01558	-0.75298	-0.16948	-0.02147
piriform area-Inferior colliculus	0	0	0	0
Taenia tecta -Inferior colliculus	0	0	0	-0.67493
Accessory olfactory bulb glomerular layer-Inferior colliculus	0	0	0	0
Accessory olfactory bulb granular layer-Inferior colliculus	0	0	0	0
Retrohippocampal region -Inferior colliculus	-0.06518	-0.41169	0	-0.4998
Entorhinal area-Inferior colliculus	-0.04839	0	-0.15331	0.570509
Field CA1-Inferior colliculus	-0.09624	-0.67894	-0.5821	-0.83978
Field CA3-Inferior colliculus	-0.27385	0.588281	-0.30135	0.222919
Dentate gyrus-Inferior colliculus	-0.19152	-0.86789	-0.38738	0.012479
Field CA2 -Inferior colliculus	0	0	0	0
Accessory olfactory bulb mitral layer-Inferior colliculus	0	0	0	0
Striatum -Inferior colliculus	-0.02609	-0.28247	-0.0929	-0.6544
Midbrain -Inferior colliculus	-0.02663	-0.58828	-0.07771	-0.18179
Medulla-Inferior colliculus	0	-0.25397	0	-0.67013
Isocortex -periaqueductal gray	-0.02333	-0.02538	-0.03128	-0.04015
Cortical amygdalar area -periaqueductal gray	0.247398	0	0.716568	-0.52338
Olfactory areas -periaqueductal gray	-0.03379	-0.04853	-0.02458	-0.01716
Pons-periaqueductal gray	-0.21184	-0.04472	-0.06273	-0.00807
Midbrain reticular nucleus-periaqueductal gray	-0.31408	-0.02538	0.162374	-0.26077
Nucleus accumbens-periaqueductal gray	0.985507	-0.41622	0	-0.33714
fimbria-periaqueductal gray	-0.11873	-0.01163	0	-0.00626
Anterior cingulate area-periaqueductal gray	-0.00198	0	-0.01991	-0.01811
Somatomotor areas-periaqueductal gray	-0.00242	-0.01476	-0.02014	-0.01819
Somatosensory areas-periaqueductal gray	-0.15167	-0.04393	-0.03057	-0.01413
piriform area-periaqueductal gray	-0.10712	0	0.582099	0.52338
Taenia tecta -periaqueductal gray	0.833408	-0.17858	-0.42485	-0.09746
Accessory olfactory bulb glomerular layer-periaqueductal gray	0	0	0	0
Accessory olfactory bulb granular layer-periaqueductal gray	0	0	0	0
Retrohippocampal region -periaqueductal gray	-0.12873	-0.02324	-0.12416	-0.01163
Entorhinal area-periaqueductal gray	-0.03114	-0.01163	-0.02357	-0.01819

Field CA1-periaqueductal gray	-0.35828	-0.0885	-0.2023	-0.02499
Field CA3-periaqueductal gray	0.394809	-0.08545	-0.15731	-0.05803
Dentate gyrus-periaqueductal gray	0.425776	-0.10871	0.80784	-0.33288
Field CA2 -periaqueductal gray	-0.20662	-0.02831	-0.13166	-0.37793
Accessory olfactory bulb mitral layer-periaqueductal gray	0	0	0	0
Striatum -periaqueductal gray	-0.00753	-0.02335	-0.05227	-0.01261
Midbrain -periaqueductal gray	-0.10689	-0.01311	-0.45784	-0.0051
Medulla-periaqueductal gray	-0.02996	-0.03359	-0.05528	-0.04015
Cortical amygdalar area -Isocortex	0.035966	-0.57399	0.603734	-0.88467
Olfactory areas -Isocortex	0.975007	-0.11208	-0.5821	-0.02884
Pons-Isocortex	-0.01444	-0.02757	-0.0113	-0.01413
Midbrain reticular nucleus-Isocortex	-0.10292	-0.02538	-0.07513	-0.03153
Nucleus accumbens-Isocortex	0.872861	-0.43828	-0.43037	-0.14244
fimbria-Ictx	-0.90481	-0.01163	-0.00475	-0.0677
Anterior cingulate area-Isocortex	-0.01319	-0.01163	-0.01335	-0.01819
Somatomotor areas-Isocortex	-0.28546	-0.40921	-0.25971	-0.15169
Somatosensory areas-Isocortex	0.864162	-0.60265	-0.14911	-0.16085
piriform area-Isocortex	0.71917	-0.66077	0.415206	0.131178
Taenia tecta -Isocortex	0.09491	-0.95898	-0.60373	-0.28966
Accessory olfactory bulb glomerular layer-Isocortex	0	0	0	0
Accessory olfactory bulb granular layer-Isocortex	0	0	0	0
Retrohippocampal region -Isocortex	-0.03379	-0.02538	-0.03385	-0.0098
Entorhinal area-Isocortex	-0.13173	-0.02538	-0.11692	-0.63577
Field CA1-Isocortex	-0.15926	-0.03199	-0.04681	-0.29328
Field CA3-Isocortex	0.866655	-0.07873	-0.18115	-0.78845
Dentate gyrus-Isocortex	0.72577	-0.0962	-0.5313	0.891092
Field CA2 -Isocortex	-0.11909	-0.02435	-0.11692	-0.40364
Accessory olfactory bulb mitral layer-Isocortex	0	0	0	0
Striatum -Isocortex	-0.39481	-0.02431	-0.24367	-0.36102
Midbrain -Isocortex	-0.00568	-0.02335	-0.02458	-0.01472
Medulla-Isocortex	-0.01201	-0.02431	-0.03385	-0.02003
Olfactory areas -Cortical amygdalar area	0	-0.58551	0	-0.64106
Pons-Cortical amygdalar area	0	0	0	0
Midbrain reticular nucleus-Cortical amygdalar area	0	0	0	0

Nucleus accumbens-Cortical amygdalar area	0	0	0	0
fimbria-Cortical amygdalar area	0	0	0	0
Anterior cingulate area-Cortical amygdalar area	0	0	0	0
Somatomotor areas-Cortical amygdalar area	0	0	0	0
Somatosensory areas-Cortical amygdalar area	0.018189	0.80436	0	-0.41533
piriform area-Cortical amygdalar area	0	0	0	0
Taenia tecta -Cortical amygdalar area	0	0	0	0
Accessory olfactory bulb glomerular layer-Cortical amygdalar area	0	0	0	0
Accessory olfactory bulb granular layer-Cortical amygdalar area	0	0	0	0
Retrohippocampal region -Cortical amygdalar area	0.20723	0	0	-0.1552
Entorhinal area-Cortical amygdalar area	0	0	0	-0.43396
Field CA1-Cortical amygdalar area	0.421221	0	0	-0.07256
Field CA3-Cortical amygdalar area	0.085516	0	0	-0.67493
Dentate gyrus-Cortical amygdalar area	0.036656	-0.34765	0	0.759618
Field CA2 -Cortical amygdalar area	0	0	0	0
Accessory olfactory bulb mitral layer-Cortical amygdalar area	0	0	0	0
Striatum -Cortical amygdalar area	0.129397	-0.30032	0.716568	0.46629
Midbrain -Cortical amygdalar area	0.194014	-0.79385	0.630976	0.339171
Medulla-Cortical amygdalar area	0	0	0	0
Pons-Olfactory areas	-0.01214	-0.03803	-0.01335	-0.01843
Midbrain reticular nucleus-Olfactory areas	-0.9871	0	0	-0.33656
Nucleus accumbens-Olfactory areas	0.382833	0	0	0
fimbria-Olfactory areas	0	0	0	0
Anterior cingulate area-Olfactory areas	0	0	0	0
Somatomotor areas-Olfactory areas	0	-0.86789	0	-0.17519
Somatosensory areas-Olfactory areas	0.024206	0.427324	-0.64016	-0.50661
piriform area-Olfactory areas	0	0	0	0
Taenia tecta -Olfactory areas	0.259947	0.043765	0.327017	0.479951
Accessory olfactory bulb glomerular layer-Olfactory areas	0	0	0	0
Accessory olfactory bulb granular layer-Olfactory areas	0	0	0	0
Retrohippocampal region -Olfactory areas	-0.00905	-0.01163	-0.05227	-0.00548
Entorhinal area-Olfactory areas	-0.1683	-0.06038	-0.06049	-0.01061
Field CA1-Olfactory areas	-0.07335	-0.1605	-0.02592	-0.01061
Field CA3-Olfactory areas	0.130241	-0.55796	-0.11826	-0.25816
Dentate gyrus-Olfactory areas	0.216679	0.925903	-0.87057	0.882006

Field CA2 -Olfactory areas	0	0	0	-0.11471
Accessory olfactory bulb mitral layer-Olfactory areas	0	0	0	0
Striatum -Olfactory areas	-0.49368	0.958977	0.095977	0.873693
Midbrain -Olfactory areas	-0.02875	-0.01163	-0.04602	-0.02001
Medulla-Olfactory areas	-0.22036	-0.1605	-0.10984	-0.20319
Midbrain reticular nucleus-Pons	-0.01365	0.968227	0	-0.30323
Nucleus accumbens-Pons	0	0	0	0
fimbria-Pons	0	0	0	0
Anterior cingulate area-Pons	0	0	0	0
Somatomotor areas-Pons	-0.01746	-0.04519	-0.07015	-0.00718
Somatosensory areas-Pons	-0.02802	-0.02538	-0.07168	-0.01819
piriform area-Pons	0	0	0	0
Taenia tecta -Pons	0	0	0	-0.00807
Accessory olfactory bulb glomerular layer-Pons	0	0	0	0
Accessory olfactory bulb granular layer-Pons	0	0	0	0
Retrohippocampal region -Pons	-0.03316	-0.01163	-0.07941	-0.03338
Entorhinal area-Pons	-0.12194	-0.32742	-0.02048	-0.02003
Field CA1-Pons	-0.04055	-0.01539	-0.06273	-0.06167
Field CA3-Pons	-0.22625	-0.02847	-0.0929	-0.02918
Dentate gyrus-Pons	-0.11909	-0.02538	-0.21238	-0.17519
Field CA2 -Pons	-0.06881	0	0	0
Accessory olfactory bulb mitral layer-Pons	0	0	0	0
Striatum -Pons	-0.03145	-0.01163	-0.00746	-0.00037
Midbrain -Pons	-0.07241	-0.04472	-0.12472	-0.02884
Medulla-Pons	-0.41943	-0.16531	-0.40763	-0.33816
Nucleus accumbens-Midbrain reticular nucleus	0	0	0	0
fimbria-Midbrain reticular nucleus	0	0	0	0
Anterior cingulate area-Midbrain reticular nucleus	0	0	0	0
Somatomotor areas-Midbrain reticular nucleus	-0.05126	0	0	-0.03313
Somatosensory areas-Midbrain reticular nucleus	-0.67278	-0.05396	-0.10241	-0.15166
piriform area-Midbrain reticular nucleus	0	0	0	0
Taenia tecta -Midbrain reticular nucleus	0.465481	0	0	0
Accessory olfactory bulb glomerular layer-Midbrain reticular nucleus	0	0	0	0
Accessory olfactory bulb granular layer-Midbrain reticular nucleus	0	0	0	0

Retrohippocampal region -Midbrain reticular nucleus	-0.29102	-0.29193	0	-0.16984
Entorhinal area-Midbrain reticular nucleus	0	0	-0.11051	-0.02605
Field CA1-Midbrain reticular nucleus	-0.42818	-0.52118	0	-0.0278
Field CA3-Midbrain reticular nucleus	0.096235	-0.17364	-0.01335	0.040151
Dentate gyrus-Midbrain reticular nucleus	0.325997	-0.42774	-0.21664	0.057872
Field CA2 -Midbrain reticular nucleus	-0.07252	0	0	-0.60659
Accessory olfactory bulb mitral layer-Midbrain reticular nucleus	0	0	0	0
Striatum -Midbrain reticular nucleus	-0.2332	-0.17437	-0.03603	-0.08114
Midbrain -Midbrain reticular nucleus	-0.00242	0.968227	-0.74425	-0.36102
Medulla-Midbrain reticular nucleus	-0.13173	0	-0.47235	0
fimbria-Nucleus accumbens	0	0	0	0
Anterior cingulate area-Nucleus accumbens	0	0	0	0
Somatomotor areas-Nucleus accumbens	0	0	0	0
Somatosensory areas-Nucleus accumbens	0.03582	0	0	0
piriform area-Nucleus accumbens	0	0	0	0
Taenia tecta -Nucleus accumbens	0	0	0	0
Accessory olfactory bulb glomerular layer-Nucleus accumbens	0	0	0	0
Accessory olfactory bulb granular layer-Nucleus accumbens	0	0	0	0
Retrohippocampal region -Nucleus accumbens	-0.3238	0	0	-0.33714
Entorhinal area-Nucleus accumbens	-0.75739	0	0	-0.14208
Field CA1-Nucleus accumbens	-0.57778	0	0	-0.05766
Field CA3-Nucleus accumbens	0.036656	-0.80907	-0.97803	-0.12202
Dentate gyrus-Nucleus accumbens	0.01958	0.729612	0.911562	-0.78329
Field CA2 -Nucleus accumbens	0	0	0	0
Accessory olfactory bulb mitral layer-Nucleus accumbens	0	0	0	0
Striatum -Nucleus accumbens	0.42818	0.909975	0.053995	-0.97093
Midbrain -Nucleus accumbens	-0.12618	0	-0.0946	-0.10275
Medulla-Nucleus accumbens	0	0	0	0
Anterior cingulate area-fimbria	0	0	0	0
Somatomotor areas-fimbria	0	0	0	0
Somatosensory areas-fimbria	0	0	0	0
piriform area-fimbria	0	0	0	0
Taenia tecta -fimbria	0	0	0	0
Accessory olfactory bulb glomerular layer-fimbria	0	0	0	0

Accessory olfactory bulb granular layer-fimbria	0	0	0	0
Retrohippocampal region -fimbria	-0.02421	0	0	-0.00807
Entorhinal area-fimbria	0	0	0	-0.00885
Field CA1-fimbria	-0.60732	0	-0.03484	-0.25816
Field CA3-fimbria	0.009424	-0.02335	-0.03714	0.083422
Dentate gyrus-fimbria	0.031458	-0.01535	-0.12654	0.337143
Field CA2 -fimbria	0	0	0	0
Accessory olfactory bulb mitral layer-fimbria	0	0	0	0
Striatum -fimbria	0.342781	-0.06189	-0.66699	-0.41751
Midbrain -fimbria	-0.14291	-0.01163	-0.01381	-0.00329
Medulla-fimbria	0	0	0	0
Somatomotor areas-Anterior cingulate area	0	0	0	0
Somatosensory areas-Anterior cingulate area	-0.01983	-0.06027	-0.03008	0
piriform area-Anterior cingulate area	0	0	0	0
Taenia tecta -Anterior cingulate area	0	0	0	0
Accessory olfactory bulb glomerular layer-Anterior cingulate area	0	0	0	0
Accessory olfactory bulb granular layer-Anterior cingulate area	0	0	0	0
Retrohippocampal region -Anterior cingulate area	0	0	0	0
Entorhinal area-Anterior cingulate area	0	0	0	0
Field CA1-Anterior cingulate area	0	0	0	0
Field CA3-Anterior cingulate area	-0.46004	0	0	-0.23007
Dentate gyrus-Anterior cingulate area	-0.02267	-0.1782	-0.12654	-0.32844
Field CA2 -Anterior cingulate area	0	0	0	0
Accessory olfactory bulb mitral layer-Anterior cingulate area	0	0	0	0
Striatum -Anterior cingulate area	-0.01201	0	0	-0.22956
Midbrain -Anterior cingulate area	-0.00242	-0.01311	-0.00888	-0.02541
Medulla-Anterior cingulate area	0	0	0	0
Somatosensory areas-Somatomotor areas	-0.76754	-0.3909	-0.32715	-0.30839
piriform area-Somatomotor areas	0	0	0	0.962433
Taenia tecta -Somatomotor areas	0	0	0	0
Accessory olfactory bulb glomerular layer-Somatomotor areas	0	0	0	0
Accessory olfactory bulb granular layer-Somatomotor areas	0	0	0	0
Retrohippocampal region -Somatomotor areas	-0.03836	-0.47536	-0.16237	-0.03636
Entorhinal area-Somatomotor areas	-0.15716	0	-0.40763	-0.20772

Field CA1-Somatomotor areas	-0.34979	0	-0.22949	-0.07072
Field CA3-Somatomotor areas	0.987639	-0.77279	0.992965	-0.81836
Dentate gyrus-Somatomotor areas	-0.38762	-0.49784	0.614275	-0.67493
Field CA2 -Somatomotor areas	0	0	0	0
Accessory olfactory bulb mitral layer-Somatomotor areas	0	0	0	0
Striatum -Somatomotor areas	-0.45789	-0.2352	0.713	-0.86627
Midbrain -Somatomotor areas	-0.0036	-0.01311	-0.03128	-0.01819
Medulla-Somatomotor areas	-0.09804	-0.04393	-0.25688	-0.01435
piriform area-Somatosensory areas	0.095217	0	0	0
Taenia tecta -Somatosensory areas	0.002415	0	0.198984	0.912116
Accessory olfactory bulb glomerular layer-Somatosensory areas	0	0	0	0
Accessory olfactory bulb granular layer-Somatosensory areas	0	0	0	0
Retrohippocampal region -Somatosensory areas	-0.22625	-0.17364	-0.4525	-0.02003
Entorhinal area-Somatosensory areas	-0.7259	-0.1605	-0.36002	-0.14244
Field CA1-Somatosensory areas	0.394809	-0.14126	-0.30239	-0.18777
Field CA3--Somatosensory areas	0.00378	-0.12672	-0.74204	-0.65104
Dentate gyrus-Somatosensory areas	0.021958	-0.46813	0.345916	-0.36843
Field CA2 -Somatosensory areas	-0.45238	0	-0.09976	-0.46629
Accessory olfactory bulb mitral layer-Somatosensory areas	0	0	0	0
Striatum --Somatosensory areas	0.042368	-0.52946	0.440806	0.354335
Midbrain --Somatosensory areas	-0.04839	-0.01219	-0.05911	-0.00548
Medulla-Somatosensory areas	-0.02104	-0.04366	-0.11051	-0.05228
Taenia tecta -piriform area	0	0	0	0
Accessory olfactory bulb glomerular layer-piriform area	0	0	0	0
Accessory olfactory bulb granular layer-piriform area	0	0	0	0
Retrohippocampal region -piriform area	-0.52542	0	0	-0.81739
Entorhinal area-piriform area	0	0	-0.15731	0.412926
Field CA1-piriform area	-0.41943	0	0	-0.95611
Field CA3-piriform area	0.358278	0	0.885483	0.328442
Dentate gyrus-piriform area	0.949331	-0.98311	-0.80784	0.207523
Field CA2 -piriform area	0	0	0	0
Accessory olfactory bulb mitral layer-piriform area	0	0	0	0
Striatum -piriform area	-0.78949	0.872113	0.856979	0.570509
Midbrain -piriform area	-0.01913	0.91226	-0.05612	0.478972

Medulla-piriform area	0	0	0	0
Accessory olfactory bulb glomerular layer-Taenia tecta	0	0	0	0
Accessory olfactory bulb granular layer-Taenia tecta	0	0	0	0
Retrohippocampal region -Taenia tecta	-0.15492	-0.08592	-0.15342	-0.04545
Entorhinal area-Taenia tecta	0	-0.02538	-0.34788	-0.04007
Field CA1-Taenia tecta	-0.94762	-0.13022	-0.41476	-0.22077
Field CA3-Taenia tecta	0.017379	0.573987	0.672186	-0.96748
Dentate gyrus-Taenia tecta	0.004199	0.529969	0.189933	0.289657
Field CA2 -Taenia tecta	0	0	0	0
Accessory olfactory bulb mitral layer-Taenia tecta	0	0	0	0
Striatum -Taenia tecta	0.134092	-0.87921	0.008133	0.24652
Midbrain -Taenia tecta	-0.35505	-0.01163	-0.30186	-0.04015
Medulla-Taenia tecta	0	0	-0.79968	-0.09583
Accessory olfactory bulb granular layer-Accessory olfactory bulb glomerular layer	0	0	0	0
Retrohippocampal region -Accessory olfactory bulb glomerular layer	0	0	0	0
Entorhinal area-Accessory olfactory bulb glomerular layer	0	0	0	0
Field CA1-Accessory olfactory bulb glomerular layer	0	0	0	0
Field CA3-Accessory olfactory bulb glomerular layer	0	0	0	0
Dentate gyrus-Accessory olfactory bulb glomerular layer	0	0	0	0
Field CA2 -Accessory olfactory bulb glomerular layer	0	0	0	0
Accessory olfactory bulb mitral layer-Accessory olfactory bulb glomerular layer	0	0	0	0
Striatum -Accessory olfactory bulb glomerular layer	0	0	0	0
Midbrain -Accessory olfactory bulb glomerular layer	0	0	0	0
Medulla-Accessory olfactory bulb glomerular layer	0	0	0	0
Retrohippocampal region -Accessory olfactory bulb granular layer	0	0	0	0
Entorhinal area-Accessory olfactory bulb granular layer	0	0	0	0
Field CA1-Accessory olfactory bulb granular layer	0	0	0	0
Field CA3-Accessory olfactory bulb granular layer	0	0	0	0
Dentate gyrus-Accessory olfactory bulb granular layer	0	0	0	0
Field CA2 -Accessory olfactory bulb granular layer	0	0	0	0
Accessory olfactory bulb mitral layer-Accessory olfactory bulb granular layer	0	0	0	0
Striatum -Accessory olfactory bulb granular layer	0	0	0	0

Midbrain -Accessory olfactory bulb granular layer	0	0	0	0
Medulla-Accessory olfactory bulb granular layer	0	0	0	0
Entorhinal area-Retrohippocampal region	-0.00956	-0.42732	-0.05911	-0.0531
Field CA1-Retrohippocampal region	-0.0131	-0.01163	-0.03259	-0.00367
Field CA3-Retrohippocampal region	-0.34948	-0.04533	-0.04729	-0.01366
Dentate gyrus-Retrohippocampal region	0.677858	-0.04519	-0.06461	-0.01941
Field CA2 -Retrohippocampal region	-0.0049	-0.02538	-0.11236	-0.02884
Accessory olfactory bulb mitral layer-Retrohippocampal region	0	0	0	0
Striatum -Retrohippocampal region	-0.04064	-0.01163	-0.02357	-0.01413
Midbrain -Retrohippocampal region	-0.00763	-0.04472	-0.0435	-0.00807
Medulla-Retrohippocampal region	-0.03814	-0.12672	-0.15331	-0.11199
Field CA1-Entorhinal area	-0.0329	-0.01311	-0.03128	-0.10275
Field CA3-Entorhinal area	-0.1499	-0.02431	-0.01335	-0.2754
Dentate gyrus-Entorhinal area	-0.3272	-0.02914	-0.01335	-0.8655
Field CA2 -Entorhinal area	0	0	0	-0.36558
Accessory olfactory bulb mitral layer-Entorhinal area	0	0	0	0
Striatum -Entorhinal area	-0.04328	-0.02538	-0.03385	-0.03029
Midbrain -Entorhinal area	-0.01305	-0.05613	-0.06831	-0.02499
Medulla-Entorhinal area	-0.1683	-0.12715	-0.14078	-0.21652
Field CA3-Field CA1	0.670741	-0.04519	-0.02098	-0.34153
Dentate gyrus-Field CA1	0.872861	-0.33509	-0.0408	-0.16836
Field CA2 -Field CA1	-0.15492	-0.02538	-0.06049	-0.0677
Accessory olfactory bulb mitral layer-Field CA1	0	0	0	0
Striatum -Field CA1	-0.07726	-0.01311	-0.03484	-0.06598
Midbrain -Field CA1	-0.03268	-0.03915	-0.1174	-0.01413
Medulla-Field CA1	-0.04247	-0.07442	-0.25086	-0.03636
Dentate gyrus-Field CA3	0.014592	-0.06954	-0.2023	0.230072
Field CA2 -Field CA3	0.158864	-0.02832	-0.04082	-0.52234
Accessory olfactory bulb mitral layer-Field CA3	0	0	0	0
Striatum -Field CA3	0.226248	-0.02538	-0.14078	0.232552
Midbrain -Field CA3	-0.78747	-0.04685	-0.23681	-0.47378
Medulla-Field CA3	-0.28835	-0.27737	-0.30135	-0.02038

Field CA2 -Dentate gyrus	-0.84443	-0.09558	-0.15002	-0.36108
Accessory olfactory bulb mitral layer-Dentate gyrus	0	0	0	0
Striatum -Dentate gyrus	0.007531	-0.0438	-0.3909	0.025841
Midbrain -Dentate gyrus	-0.67229	-0.49754	-0.16028	-0.48117
Medulla-Dentate gyrus	-0.21839	-0.40921	-0.15867	-0.43186
Accessory olfactory bulb mitral layer-Field CA2	0	0	0	0
Striatum -Field CA2	-0.00833	-0.01163	-0.32278	-0.175
Midbrain -Field CA2	-0.04237	-0.03992	-0.05227	-0.04222
Medulla-Field CA2	0	0	0	0
Striatum -Accessory olfactory bulb mitral layer	0	0	0	0
Midbrain -Accessory olfactory bulb mitral layer	0	0	0	0
Medulla-Accessory olfactory bulb mitral layer	0	0	0	0
Midbrain -Striatum	-0.00355	-0.01292	-0.03813	-0.04015
Medulla-Striatum	-0.02528	-0.01163	-0.03852	-0.01472
Medulla-Midbrain	-0.02175	-0.07462	-0.05911	-0.08284

S10. Adjust p values for group comparisons of pair-wised connectivity for Entorhinal Cortex.

Region Connections	Entorhinal Cortex			
	Left Ipsilateral	Left Contralateral	Right Contralateral	Right Ipsilateral
Caudoputamen-corpus callosum	0	-0.00551	-0.08372	-0.00015
anterior commissure olfactory limb-corpus callosum	0	0	0	0
pallidum -corpus callosum	-0.01487	0	0	-0.02834
internal capsule-corpus callosum	0	0	0	0
Thalamus-corpus callosum	0	0	0	0
Cerebellum-corpus callosum	-0.24238	0	-0.05661	-0.03785
Superior colliculus-corpus callosum	0	0	0	0
ventricular systems-corpus callosum	-0.00515	0	-0.01081	-0.00172
Hypothalamus -corpus callosum	0	0	0	0
Inferior colliculus -corpus callosum	0	0	0	0
periaqueductal gray-corpus callosum	0	0	0	0
Isocortex -corpus callosum	-0.04825	-0.0024	-0.00528	-0.02137
Cortical amygdalar area -corpus callosum	-0.96806	0	-0.00144	-0.93902
Olfactory areas -corpus callosum	-0.24238	0	-0.05661	-0.02949
Pons-corpus callosum	0	0	0	0
Midbrain reticular nucleus-corpus callosum	0	0	0	0
Nucleus accumbens-corpus callosum	0	0	0	-0.12528
fimbria-corpus callosum	0	0	0	0
Anterior cingulate area-corpus callosum	0	0	0	0
Somatomotor areas-corpus callosum	0	0	0	0
Somatosensory areas-corpus callosum	0	0	0	0
piriform area-corpus callosum	0.769652	-0.07341	-0.21422	0.440474
Taenia tecta -corpus callosum	0	0	-0.05661	-0.01457
Accessory olfactory bulb glomerular layer-corpus callosum	0	0	0	0
Accessory olfactory bulb granular layer-corpus callosum	0	0	0	0
Retrohippocampal region -corpus callosum	-0.01456	-0.00551	-0.02923	-0.09549
Entorhinal area-corpus callosum	-0.04574	-0.00259	-0.0045	-0.05156
Field CA1-corpus callosum	-0.07082	0	-0.02189	-0.48592
Field CA3-corpus callosum	0	0	0	-0.01614
Dentate gyrus-corpus callosum	-0.00753	0	-0.11196	-0.02742
Field CA2 -corpus callosum	0	0	0	0
Accessory olfactory bulb mitral layer-corpus callosum	0	0	0	0

Striatum -corpus callosum	-0.00373	-0.01498	-0.03263	-0.0109
Midbrain -corpus callosum	0	0	0	0
Medulla-corpus callosum	0	0	0	0
anterior commissure olfactory limb-Caudoputamen	0	0	0	0
pallidum -Caudoputamen	0	0	0	0
internal capsule-Caudoputamen	0	0	0	0
Thalamus-Caudoputamen	0	0	0	0
Cerebellum-Caudoputamen	-0.576842	0	0	-0.014001
Superior colliculus-Caudoputamen	0	0	0	0
ventricular systems-Caudoputamen	0	0	0	-0.054371
Hypothalamus -Caudoputamen	0	0	0	0
Inferior colliculus -Caudoputamen	0	0	0	0
periaqueductal gray-Caudoputamen	0	0	0	0
Isocortex -Caudoputamen	-0.05586	-0.002397	-0.001444	-0.03056
Cortical amygdalar area -Caudoputamen	-0.12553	0	0	-0.138735
Olfactory areas -Caudoputamen	0	0	-0.404806	-0.035781
Pons-Caudoputamen	0	0	0	0
Midbrain reticular nucleus-Caudoputamen	0	0	0	0
Nucleus accumbens-Caudoputamen	0	0	0	0
fimbria-Caudoputamen	0	0	0	0
Anterior cingulate area-Caudoputamen	0	0	0	0
Somatomotor areas-Caudoputamen	0	0	0	0
Somatosensory areas-Caudoputamen	0	0	0	0
piriform area-Caudoputamen	-0.567076	0	0	0.121744
Taenia tecta -Caudoputamen	0	0	0	0
Accessory olfactory bulb glomerular layer-Caudoputamen	0	0	0	0
Accessory olfactory bulb granular layer-Caudoputamen	0	0	0	0
Retrohippocampal region -Caudoputamen	-0.005153	-0.233186	0	-0.003265
Entorhinal area-Caudoputamen	-0.061256	-0.011206	-0.008298	-0.024624
Field CA1-Caudoputamen	0	0	0	-0.010728
Field CA3-Caudoputamen	0	0	0	0
Dentate gyrus-Caudoputamen	-0.501138	0	-0.092759	-0.031772
Field CA2 -Caudoputamen	0	0	0	0
Accessory olfactory bulb mitral layer-Caudoputamen	0	0	0	0
Striatum -Caudoputamen	0	0	-0.167824	-0.094096
Midbrain -Caudoputamen	0	0	0	0

Medulla-Caudoputamen	0	0	0	0
pallidum -anterior commissure olfactory limb	0	0	0	0
internal capsule-anterior commissure olfactory limb	0	0	0	0
Thalamus-anterior commissure olfactory limb	0	0	0	0
Cerebellum-anterior commissure olfactory limb	0	0	0	0
Superior colliculus-anterior commissure olfactory limb	0	0	0	0
ventricular systems-anterior commissure olfactory limb	0	0	0	0
Hypothalamus -anterior commissure olfactory limb	0	0	0	0
Inferior colliculus -anterior commissure olfactory limb	0	0	0	0
periaqueductal gray-anterior commissure olfactory limb	0	0	0	0
Isocortex -anterior commissure olfactory limb	0	0	0	0
Cortical amygdalar area -anterior commissure olfactory limb	0	0	0	0
Olfactory areas -anterior commissure olfactory limb	0	0	0	0
Pons-anterior commissure olfactory limb	0	0	0	0
Midbrain reticular nucleus-anterior commissure olfactory limb	0	0	0	0
Nucleus accumbens-anterior commissure olfactory limb	0	0	0	0
fimbria-anterior commissure olfactory limb	0	0	0	0
Anterior cingulate area-anterior commissure olfactory limb	0	0	0	0
Somatomotor areas-anterior commissure olfactory limb	0	0	0	0
Somatosensory areas-anterior commissure olfactory limb	0	0	0	0
piriform area-anterior commissure olfactory limb	0	0	0	0
Taenia tecta -anterior commissure olfactory limb	0	0	0	0
Accessory olfactory bulb glomerular layer-anterior commissure olfactory limb	0	0	0	0
Accessory olfactory bulb granular layer-anterior commissure olfactory limb	0	0	0	0
Retrohippocampal region -anterior commissure olfactory limb	0	0	0	0
Entorhinal area-anterior commissure olfactory limb	-0.071254	0	0	-0.040839
Field CA1-anterior commissure olfactory limb	0	0	0	0
Field CA3-anterior commissure olfactory limb	0	0	0	0
Dentate gyrus-anterior commissure olfactory limb	0	0	0	0
Field CA2 -anterior commissure olfactory limb	0	0	0	0
Accessory olfactory bulb mitral layer-anterior commissure olfactory limb	0	0	0	0
Striatum -anterior commissure olfactory limb	0	0	0	0
Midbrain -anterior commissure olfactory limb	0	0	0	0
Medulla-anterior commissure olfactory limb	0	0	0	0

internal capsule-pallidum	0	0	0	0
Thalamus-pallidum	0	0	0	0
Cerebellum-pallidum	0	0	0	-0.002439
Superior colliculus-pallidum	0	0	0	0
ventricular systems-pallidum	0	0	0	0
Hypothalamus -pallidum	0	0	0	0
Inferior colliculus -pallidum	0	0	0	0
periaqueductal gray-pallidum	0	0	0	0
Isocortex -pallidum	-0.004757	-0.002397	-0.032839	-0.00127
Cortical amygdalar area -pallidum	0	0	0	-0.01457
Olfactory areas -pallidum	0	0	0	-0.225686
Pons-pallidum	0	0	0	0
Midbrain reticular nucleus-pallidum	0	0	0	0
Nucleus accumbens-pallidum	0	0	0	0
fimbria-pallidum	0	0	0	0
AVA-pallidum	0	0	0	0
Somatomotor areas-pallidum	0	0	0	0
Somatosensory areas-pallidum	0	0	0	0
piriform area-pallidum	-0.163696	0	0	0
Taenia tecta -pallidum	0	0	0	0
Accessory olfactory bulb glomerular layer-pallidum	0	0	0	0
Accessory olfactory bulb granular layer-pallidum	0	0	0	0
Retrohippocampal region -pallidum	-0.000815	0	0	-0.031916
Entorhinal area-pallidum	-0.001656	-0.003688	-0.00555	-0.001162
Field CA1-pallidum	0	0	0	-0.043333
Field CA3-pallidum	0	0	0	0
Dentate gyrus-pallidum	-0.064982	0	0	-0.206217
Field CA2 -pallidum	0	0	0	0
Accessory olfactory bulb mitral layer-pallidum	0	0	0	0
Striatum -pallidum	0	0	0	-0.125275
Midbrain -pallidum	0	0	0	0
Medulla-pallidum	0	0	0	0
Thalamus-internal capsule	0	0	0	0
Cerebellum-internal capsule	0	0	0	0
Superior colliculus-internal capsule	0	0	0	0

ventricular systems-internal capsule	0	0	0	0
Hypothalamus -internal capsule	0	0	0	0
Inferior colliculus -internal capsule	0	0	0	0
periaqueductal gray-internal capsule	0	0	0	0
Isocortex -internal capsule	0	0	0	0
Cortical amygdalar area -internal capsule	0	0	0	0
Olfactory areas -internal capsule	0	0	0	0
Pons-internal capsule	0	0	0	0
Midbrain reticular nucleus-internal capsule	0	0	0	0
Nucleus accumbens-internal capsule	0	0	0	0
fimbria-internal capsule	0	0	0	0
Anterior cingulate area-internal capsule	0	0	0	0
Somatomotor areas-internal capsule	0	0	0	0
Somatosensory areas-internal capsule	0	0	0	0
piriform area-internal capsule	0	0	0	0
Taenia tecta -internal capsule	0	0	0	0
Accessory olfactory bulb glomerular layer-internal capsule	0	0	0	0
Accessory olfactory bulb granular layer-internal capsule	0	0	0	0
Retrohippocampal region -internal capsule	0	0	0	0
Entorhinal area-internal capsule	-0.496319	0	0	-0.153504
Field CA1-internal capsule	0	0	0	0
Field CA3-internal capsule	0	0	0	0
Dentate gyrus-internal capsule	0	0	0	0
Field CA2 -internal capsule	0	0	0	0
Accessory olfactory bulb mitral layer-internal capsule	0	0	0	0
Striatum -internal capsule	0	0	0	0
Midbrain --internal capsule	0	0	0	0
Medulla-internal capsule	0	0	0	0
Cerebellum-Thalamus	-0.19389	-0.94932	0	-0.26869
Superior colliculus-Thalamus	0	0	0	0
ventricular systems-Thalamus	0	0	0	-0.03953
Hypothalamus -Thalamus	0	0	0	0
Inferior colliculus -Thalamus	0	0	0	0
periaqueductal gray-Thalamus	0	0	0	0
Isocortex -Thalamus	-0.00476	-0.01882	-0.23096	-0.12528

Cortical amygdalar area -Thalamus	-0.06498	0	0	-0.03483
Olfactory areas -Thalamus	0	-0.16675	0	-0.02603
Pons-Thalamus	0	0	0	0
Midbrain reticular nucleus-Thalamus	0	0	0	0
Nucleus accumbens-Thalamus	0	0	0	0
fimbria-Thalamus	0	0	0	0
Anterior cingulate area-Thalamus	0	0	0	0
Somatomotor areas-Thalamus	0	0	0	0
Somatosensory areas-Thalamus	0	0	0	0
piriform area-Thalamus	-0.11815	0	0	-0.70237
Taenia tecta -Thalamus	0	0	0	-0.09966
Accessory olfactory bulb glomerular layer-Thalamus	0	0	0	0
Accessory olfactory bulb granular layer-Thalamus	0	0	0	0
Retrohippocampal region -Thalamus	-0.05987	0	0	-0.26869
Entorhinal area-Thalamus	-0.0133	-0.02048	-0.05661	-0.43111
Field CA1-Thalamus	0	0	0	0
Field CA3-Thalamus	0	0	0	0
Dentate gyrus-Thalamus	0	0	0	-0.36028
Field CA2 -Thalamus	0	0	0	0
Accessory olfactory bulb mitral layer-Thalamus	0	0	0	0
Striatum -Thalamus	0	0	0	-0.10029
Midbrain -Thalamus	0	0	0	0
Medulla-Thalamus	0	0	0	0
Superior colliculus-Cerebellum	0.867171	0.501521	0	-0.88797
ventricular systems-Cerebellum	-0.49632	-0.11196	-0.63947	-0.05162
Hypothalamus -Cerebellum	-0.84971	-0.09903	0	-0.00755
Inferior colliculus -Cerebellum	0	0	0	0
periaqueductal gray-Cerebellum	0	0	0	0
Isocortex -Cerebellum	0.002033	-0.10797	0.687528	0.485915
Cortical amygdalar area -Cerebellum	0.205179	-0.4449	0.467879	0.871954
Olfactory areas -Cerebellum	-0.50006	0	-0.48101	-0.09563
Pons-Cerebellum	0	0	0	0
Midbrain reticular nucleus-Cerebellum	0	0	0	0
Nucleus accumbens-Cerebellum	0	0	0	0
fimbria-Cerebellum	0	0	0	0

Anterior cingulate area-Cerebellum	0	0	0	0
Somatomotor areas-Cerebellum	-0.10671	0	0	0
Somatosensory areas-Cerebellum	-0.4274	-0.11196	0	-0.00202
piriform area-Cerebellum	-0.60252	-0.80097	0	-0.59997
Taenia tecta -Cerebellum	0	0	0	-0.00417
Accessory olfactory bulb glomerular layer-Cerebellum	0	0	0	0
Accessory olfactory bulb granular layer-Cerebellum	0	0	0	0
Retrohippocampal region -Cerebellum	0.034741	0.582903	0.450584	0.221757
Entorhinal area-Cerebellum	0.023448	-0.97455	-0.48927	0.18595
Field CA1-Cerebellum	0.242384	-0.44856	-0.60296	-0.2044
Field CA3-Cerebellum	0.242384	0	0	-0.28641
Dentate gyrus-Cerebellum	0.034741	0.405532	0.828707	-0.91169
Field CA2 -Cerebellum	0	0	0	-0.10321
Accessory olfactory bulb mitral layer-Cerebellum	0	0	0	0
Striatum -Cerebellum	-0.25653	-0.06558	-0.19088	-0.01308
Midbrain -Cerebellum	-0.31038	0.882357	0	0.441825
Medulla-Cerebellum	0.152721	0	0	0.235506
ventricular systems-Superior colliculus	0	0	0	0
Hypothalamus -Superior colliculus	0	0	0	0
Inferior colliculus -Superior colliculus	0	0	0	0
periaqueductal gray-Superior colliculus	0	0	0	0
Isocortex -Superior colliculus	-0.03663	-0.0233	-0.40481	-0.04271
Cortical amygdalar area -Superior colliculus	-0.1392	0	0	-0.03569
Olfactory areas -Superior colliculus	0	0	0	-0.0357
Pons-Superior colliculus	0	0	0	0
Midbrain reticular nucleus-Superior colliculus	0	0	0	0
Nucleus accumbens-Superior colliculus	0	0	0	0
fimbria-Superior colliculus	0	0	0	0
Anterior cingulate area-Superior colliculus	0	0	0	0
Somatomotor areas-Superior colliculus	0	0	0	0
Somatosensory areas-Superior colliculus	0	0	0	0
piriform area-Superior colliculus	-0.0984	0	0	0
Taenia tecta -Superior colliculus	0	0	0	0
Accessory olfactory bulb glomerular layer-Superior colliculus	0	0	0	0
Accessory olfactory bulb granular layer-Superior colliculus	0	0	0	0

Retrohippocampal region -Superior colliculus	-0.10671	0	0.058082	-0.20837
Entorhinal area-Superior colliculus	-0.09025	-0.0935	-0.76394	-0.21747
Field CA1-Superior colliculus	0	0	0	0
Field CA3-Superior colliculus	0	0	0	0
Dentate gyrus-Superior colliculus	0	0	0	0
Field CA2 -Superior colliculus	0	0	0	0
Accessory olfactory bulb mitral layer-Superior colliculus	0	0	0	0
Striatum -Superior colliculus	0	0	0	-0.06393
Midbrain -Superior colliculus	0	0	0	0
Medulla-Superior colliculus	0	0	0	0
Hypothalamus -ventricular systems	0	-0.175836	0	0
Inferior colliculus -ventricular systems	0	0	0	0
periaqueductal gray-ventricular systems	0	0	0	0
Isocortex -ventricular systems	-0.069294	-0.003325	-0.010809	-0.026132
Cortical amygdalar area -ventricular systems	-0.566311	-0.098805	-0.025867	0.817559
Olfactory areas -ventricular systems	-0.187111	-0.093502	-0.190881	-0.017361
Pons-ventricular systems	0	0	0	0
Midbrain reticular nucleus-ventricular systems	0	0	0	0
Nucleus accumbens-ventricular systems	0	0	0	0
fimbria-ventricular systems	0	0	0	0
Anterior cingulate area-ventricular systems	0	0	0	0
Somatomotor areas-ventricular systems	0	0	0	0
Somatosensory areas-ventricular systems	0	-0.048247	0	0
piriform area-ventricular systems	0.94982	-0.045741	0	0.24215
Taenia tecta -ventricular systems	0	0	0	-0.023313
Accessory olfactory bulb glomerular layer-ventricular systems	0	0	0	0
Accessory olfactory bulb granular layer-ventricular systems	0	0	0	0
Retrohippocampal region -ventricular systems	-0.02193	-0.003325	-0.041981	-0.051563
Entorhinal area-ventricular systems	-0.169651	-0.015332	-0.028871	-0.222985
Field CA1-ventricular systems	-0.61168	0	0	-0.03056
Field CA3-ventricular systems	0	0	0	-0.18595
Dentate gyrus-ventricular systems	-0.949045	-0.233186	-0.057431	-0.026034
Field CA2 -ventricular systems	0	0	0	0
Accessory olfactory bulb mitral layer-ventricular systems	0	0	0	0
Striatum -ventricular systems	-0.005153	-0.002397	0	-0.161533

Midbrain -ventricular systems	0	0	0	0
Medulla-ventricular systems	0	0	0	0
Inferior colliculus -Hypothalamus	0	0	0	0
periaqueductal gray-Hypothalamus	0	0	0	0
Isocortex -Hypothalamus	-0.030616	-0.020483	-0.011784	-0.037849
Cortical amygdalar area -Hypothalamus	0	0	0	0
Olfactory areas -Hypothalamus	0	0	0	-0.204396
Pons-Hypothalamus	0	0	0	0
Midbrain reticular nucleus-Hypothalamus	0	0	0	0
Nucleus accumbens-Hypothalamus	0	0	0	0
fimbria-Hypothalamus	0	0	0	0
Anterior cingulate area-Hypothalamus	0	0	0	0
Somatomotor areas-Hypothalamus	0	0	0	0
Somatosensory areas-Hypothalamus	0	0	0	0
piriform area-Hypothalamus	0	-0.401491	0	0
Taenia tecta -Hypothalamus	0	0	0	0
Accessory olfactory bulb glomerular layer-Hypothalamus	0	0	0	0
Accessory olfactory bulb granular layer-Hypothalamus	0	0	0	0
Retrohippocampal region -Hypothalamus	-0.044346	-0.401491	-0.041752	-0.140424
Entorhinal area-Hypothalamus	-0.163696	-0.109529	-0.003061	-0.017361
Field CA1-Hypothalamus	0	0	0	0
Field CA3-Hypothalamus	0	0	0	0
Dentate gyrus-Hypothalamus	0	0	0	0
Field CA2 -Hypothalamus	0	0	0	0
Accessory olfactory bulb mitral layer-Hypothalamus	0	0	0	0
Striatum -Hypothalamus	0	0	0	-0.225686
Midbrain -Hypothalamus	0	0	0	0
Medulla-Hypothalamus	0	0	0	0
periaqueductal gray-Inferior colliculus	0	0	0	0
Isocortex -Inferior colliculus	0	0	0	0.504182
Cortical amygdalar area -Inferior colliculus	0	0	0	-0.45058
Olfactory areas -Inferior colliculus	0	0	0	0
Pons-Inferior colliculus	0	0	0	0
Midbrain reticular nucleus-Inferior colliculus	0	0	0	0

Nucleus accumbens-Inferior colliculus	0	0	0	0
fimbria-Inferior colliculus	0	0	0	0
Anterior cingulate area-Inferior colliculus	0	0	0	0
Somatomotor areas-Inferior colliculus	0	0	0	0
Somatosensory areas-Inferior colliculus	0	0	0	0
piriform area-Inferior colliculus	0	0	0	0
Taenia tecta -Inferior colliculus	0	0	0	0
Accessory olfactory bulb glomerular layer-Inferior colliculus	0	0	0	0
Accessory olfactory bulb granular layer-Inferior colliculus	0	0	0	0
Retrohippocampal region -Inferior colliculus	0	0	0	0
Entorhinal area-Inferior colliculus	-0.08626	-0.40149	-0.23624	0.676647
Field CA1-Inferior colliculus	0	0	0	0
Field CA3-Inferior colliculus	0	0	0	0
Dentate gyrus-Inferior colliculus	0	0	0	0
Field CA2 -Inferior colliculus	0	0	0	0
Accessory olfactory bulb mitral layer-Inferior colliculus	0	0	0	0
Striatum -Inferior colliculus	0	0	0	0
Midbrain -Inferior colliculus	0	0	0	0
Medulla-Inferior colliculus	0	0	0	0
Isocortex -periaqueductal gray	-0.00835	-0.02156	-0.2952	-0.03578
Cortical amygdalar area -periaqueductal gray	0	0	0	0
Olfactory areas -periaqueductal gray	0	0	0	0
Pons-periaqueductal gray	0	0	0	0
Midbrain reticular nucleus-periaqueductal gray	0	0	0	0
Nucleus accumbens-periaqueductal gray	0	0	0	0
fimbria-periaqueductal gray	0	0	0	0
Anterior cingulate area-periaqueductal gray	0	0	0	0
Somatomotor areas-periaqueductal gray	0	0	0	0
Somatosensory areas-periaqueductal gray	0	0	0	0
piriform area-periaqueductal gray	0	0	0	0
Taenia tecta -periaqueductal gray	0	0	0	0
Accessory olfactory bulb glomerular layer-periaqueductal gray	0	0	0	0
Accessory olfactory bulb granular layer-periaqueductal gray	0	0	0	0
Retrohippocampal region -periaqueductal gray	0	0	0	0
Entorhinal area-periaqueductal gray	-0.24238	-0.05224	-0.05715	-0.01308

Field CA1-periaqueductal gray	0	0	0	0
Field CA3-periaqueductal gray	0	0	0	0
Dentate gyrus-periaqueductal gray	0	0	0	0
Field CA2 -periaqueductal gray	0	0	0	0
Accessory olfactory bulb mitral layer-periaqueductal gray	0	0	0	0
Striatum -periaqueductal gray	0	0	0	0
Midbrain -periaqueductal gray	0	0	0	0
Medulla-periaqueductal gray	0	0	0	0
Cortical amygdalar area -Isocortex	0.494528	-0.07545	-0.00144	-0.99359
Olfactory areas -Isocortex	-0.50006	-0.02784	-0.00592	-0.01308
Pons-Isocortex	-0.2662	-0.51537	-0.2669	-0.15056
Midbrain reticular nucleus-Isocortex	0	0	0	0
Nucleus accumbens-Isocortex	-0.04825	-0.10459	-0.10598	-0.00417
fimbria-Ictx	0	0	0	0
Anterior cingulate area-Isocortex	0	0	-0.00144	-0.00187
Somatomotor areas-Isocortex	-0.01199	-0.0216	-0.00528	-0.014
Somatosensory areas-Isocortex	-0.08611	-0.03372	-0.00161	-0.16153
piriform area-Isocortex	0.008353	-0.02048	-0.40481	0.035781
Taenia tecta -Isocortex	-0.03235	-0.09903	-0.00161	-0.00293
Accessory olfactory bulb glomerular layer-Isocortex	0	0	0	0
Accessory olfactory bulb granular layer-Isocortex	0	0	0	0
Retrohippocampal region -Isocortex	-0.04825	-0.02561	-0.02587	0.90933
Entorhinal area-Isocortex	-0.90567	-0.00637	-0.03151	0.750491
Field CA1-Isocortex	-0.61815	-0.06558	-0.00163	0.920606
Field CA3-Isocortex	-0.04739	-0.08695	-0.01081	-0.12973
Dentate gyrus-Isocortex	-0.01978	-0.10797	-0.01371	-0.70512
Field CA2 -Isocortex	-0.00198	0	-0.01081	-0.04347
Accessory olfactory bulb mitral layer-Isocortex	0	0	0	0
Striatum -Isocortex	-0.05586	-0.01129	-0.00163	-0.00172
Midbrain -Isocortex	-0.00515	-0.72916	-0.00182	-0.02462
Medulla-Isocortex	0.328326	-0.29765	-0.20404	-0.22176
Olfactory areas -Cortical amygdalar area	-0.24238	0	0	-0.04357
Pons-Cortical amygdalar area	0.077665	0	0	0.643531
Midbrain reticular nucleus-Cortical amygdalar area	0	0	0	0

Nucleus accumbens-Cortical amygdalar area	0	0	0	0
fimbria-Cortical amygdalar area	0	0	0	0
Anterior cingulate area-Cortical amygdalar area	0	0	0	0
Somatomotor areas-Cortical amygdalar area	0	0	0	0
Somatosensory areas-Cortical amygdalar area	0.874041	-0.06161	0	-0.63106
piriform area-Cortical amygdalar area	-0.12834	0	0	0.774519
Taenia tecta -Cortical amygdalar area	0	0	0	-0.00493
Accessory olfactory bulb glomerular layer-Cortical amygdalar area	0	0	0	0
Accessory olfactory bulb granular layer-Cortical amygdalar area	0	0	0	0
Retrohippocampal region -Cortical amygdalar area	0.501138	0	0	0.052874
Entorhinal area-Cortical amygdalar area	0.003727	-0.01121	-0.07987	0.024624
Field CA1-Cortical amygdalar area	0.403317	0	0	0.612474
Field CA3-Cortical amygdalar area	-0.80709	0	0	-0.69
Dentate gyrus-Cortical amygdalar area	0.733883	0	0	0.222985
Field CA2 -Cortical amygdalar area	0	0	0	-0.22569
Accessory olfactory bulb mitral layer-Cortical amygdalar area	0	0	0	0
Striatum -Cortical amygdalar area	-0.01989	-0.00418	0	-0.22176
Midbrain -Cortical amygdalar area	-0.01929	-0.79853	0	-0.16153
Medulla-Cortical amygdalar area	0	0	0	0.817559
Pons-Olfactory areas	0	0	0	0
Midbrain reticular nucleus-Olfactory areas	0	0	0	0
Nucleus accumbens-Olfactory areas	0	0	0	0
fimbria-Olfactory areas	0	0	0	0
Anterior cingulate area-Olfactory areas	0	0	0	0
Somatomotor areas-Olfactory areas	0	0	0	-0.01115
Somatosensory areas-Olfactory areas	0	-0.05218	0	-0.07239
piriform area-Olfactory areas	-0.07496	-0.39469	0	-0.00035
Taenia tecta -Olfactory areas	0	0	0	-0.00417
Accessory olfactory bulb glomerular layer-Olfactory areas	0	0	0	0
Accessory olfactory bulb granular layer-Olfactory areas	0	0	0	0
Retrohippocampal region -Olfactory areas	-0.00476	-0.09903	-0.04039	-0.21145
Entorhinal area-Olfactory areas	-0.06419	-0.04359	-0.01081	-0.02603
Field CA1-Olfactory areas	-0.49632	-0.43417	0	-0.21635
Field CA3-Olfactory areas	0	0	0	-0.11448
Dentate gyrus-Olfactory areas	-0.05138	-0.14257	-0.05655	-0.15056

Field CA2 -Olfactory areas	0	0	0	0
Accessory olfactory bulb mitral layer-Olfactory areas	0	0	0	0
Striatum -Olfactory areas	0	-0.23319	0	-0.02462
Midbrain -Olfactory areas	0	-0.51537	0	-0.014
Medulla-Olfactory areas	0	0	0	0
Midbrain reticular nucleus-Pons	0	0	0	0
Nucleus accumbens-Pons	0	0	0	0
fimbria-Pons	0	0	0	0
Anterior cingulate area-Pons	0	0	0	0
Somatomotor areas-Pons	0	0	0	0
Somatosensory areas-Pons	0	0	0	0
piriform area-Pons	0	0	0	0
Taenia tecta -Pons	0	0	0	0
Accessory olfactory bulb glomerular layer-Pons	0	0	0	0
Accessory olfactory bulb granular layer-Pons	0	0	0	0
Retrohippocampal region -Pons	0.520226	0	0	0
Entorhinal area-Pons	0.755996	-0.01377	-0.04039	-0.04972
Field CA1-Pons	0	0	0	0
Field CA3-Pons	0	0	0	0
Dentate gyrus-Pons	0	0	0	0
Field CA2 -Pons	0	0	0	0
Accessory olfactory bulb mitral layer-Pons	0	0	0	0
Striatum -Pons	0	0	0	0
Midbrain -Pons	0	0	0	0
Medulla-Pons	0	0	0	0
Nucleus accumbens-Midbrain reticular nucleus	0	0	0	0
fimbria-Midbrain reticular nucleus	0	0	0	0
Anterior cingulate area-Midbrain reticular nucleus	0	0	0	0
Somatomotor areas-Midbrain reticular nucleus	0	0	0	0
Somatosensory areas-Midbrain reticular nucleus	0	0	0	0
piriform area-Midbrain reticular nucleus	0	0	0	0
Taenia tecta -Midbrain reticular nucleus	0	0	0	0
Accessory olfactory bulb glomerular layer-Midbrain reticular nucleus	0	0	0	0
Accessory olfactory bulb granular layer-Midbrain reticular nucleus	0	0	0	0

Retrohippocampal region -Midbrain reticular nucleus	0	0	0	0
Entorhinal area-Midbrain reticular nucleus	0.80709	0	0	-0.05437
Field CA1-Midbrain reticular nucleus	0	0	0	0
Field CA3-Midbrain reticular nucleus	0	0	0	0
Dentate gyrus-Midbrain reticular nucleus	0	0	0	0
Field CA2 -Midbrain reticular nucleus	0	0	0	0
Accessory olfactory bulb mitral layer-Midbrain reticular nucleus	0	0	0	0
Striatum -Midbrain reticular nucleus	0	0	0	0
Midbrain -Midbrain reticular nucleus	0	0	0	0
Medulla-Midbrain reticular nucleus	0	0	0	0
fimbria-Nucleus accumbens	0	0	0	0
Anterior cingulate area-Nucleus accumbens	0	0	0	0
Somatomotor areas-Nucleus accumbens	0	0	0	0
Somatosensory areas-Nucleus accumbens	0	0	0	0
piriform area-Nucleus accumbens	0	0	0	0
Taenia tecta -Nucleus accumbens	0	0	0	0
Accessory olfactory bulb glomerular layer-Nucleus accumbens	0	0	0	0
Accessory olfactory bulb granular layer-Nucleus accumbens	0	0	0	0
Retrohippocampal region -Nucleus accumbens	-0.04739	0	0	-0.29332
Entorhinal area-Nucleus accumbens	-0.00198	-0.10459	-0.02587	-0.00127
Field CA1-Nucleus accumbens	0	0	0	0
Field CA3-Nucleus accumbens	0	0	0	0
Dentate gyrus-Nucleus accumbens	0	0	0	-0.44906
Field CA2 -Nucleus accumbens	0	0	0	0
Accessory olfactory bulb mitral layer-Nucleus accumbens	0	0	0	0
Striatum -Nucleus accumbens	0	0	0	0
Midbrain -Nucleus accumbens	0	0	0	0
Medulla-Nucleus accumbens	0	0	0	0
Anterior cingulate area-fimbria	0	0	0	0
Somatomotor areas-fimbria	0	0	0	0
Somatosensory areas-fimbria	0	0	0	0
piriform area-fimbria	0	0	0	0
Taenia tecta -fimbria	0	0	0	0
Accessory olfactory bulb glomerular layer-fimbria	0	0	0	0

Accessory olfactory bulb granular layer-fimbria	0	0	0	0
Retrohippocampal region -fimbria	0	0	0	0
Entorhinal area-fimbria	-0.06498	0	0	-0.00401
Field CA1-fimbria	0	0	0	0
Field CA3-fimbria	0	0	0	0
Dentate gyrus-fimbria	0	0	0	0
Field CA2 -fimbria	0	0	0	0
Accessory olfactory bulb mitral layer-fimbria	0	0	0	0
Striatum -fimbria	0	0	0	0
Midbrain -fimbria	0	0	0	0
Medulla-fimbria	0	0	0	0
Somatomotor areas-Anterior cingulate area	0	0	0	0
Somatosensory areas-Anterior cingulate area	0	0	0	0
piriform area-Anterior cingulate area	0	0	0	0
Taenia tecta -Anterior cingulate area	0	0	0	0
Accessory olfactory bulb glomerular layer-Anterior cingulate area	0	0	0	0
Accessory olfactory bulb granular layer-Anterior cingulate area	0	0	0	0
Retrohippocampal region -Anterior cingulate area	0	0	0	0
Entorhinal area-Anterior cingulate area	0	-0.00034	-0.00163	-0.00035
Field CA1-Anterior cingulate area	0	0	0	0
Field CA3-Anterior cingulate area	0	0	0	0
Dentate gyrus-Anterior cingulate area	0	0	0	0
Field CA2 -Anterior cingulate area	0	0	0	0
Accessory olfactory bulb mitral layer-Anterior cingulate area	0	0	0	0
Striatum -Anterior cingulate area	0	0	0	0
Midbrain -Anterior cingulate area	0	0	0	0
Medulla-Anterior cingulate area	0	0	0	0
Somatosensory areas-Somatomotor areas	0	0	0	0
piriform area-Somatomotor areas	-0.07125	0	0	0
Taenia tecta -Somatomotor areas	0	0	0	0
Accessory olfactory bulb glomerular layer-Somatomotor areas	0	0	0	0
Accessory olfactory bulb granular layer-Somatomotor areas	0	0	0	0
Retrohippocampal region -Somatomotor areas	-0.00515	0	0	-0.01941
Entorhinal area-Somatomotor areas	-0.000033	-0.0087	-0.07646	-0.01614

Field CA1-Somatomotor areas	0	0	0	0
Field CA3-Somatomotor areas	0	0	0	0
Dentate gyrus-Somatomotor areas	0	0	0	0
Field CA2 -Somatomotor areas	0	0	0	0
Accessory olfactory bulb mitral layer-Somatomotor areas	0	0	0	0
Striatum -Somatomotor areas	0	0	0	-0.00116
Midbrain -Somatomotor areas	0	0	0	0
Medulla-Somatomotor areas	0	0	0	0
piriform area-Somatosensory areas	0.030616	0	0	0.07239
Taenia tecta -Somatosensory areas	0	0	0	0
Accessory olfactory bulb glomerular layer-Somatosensory areas	0	0	0	0
Accessory olfactory bulb granular layer-Somatosensory areas	0	0	0	0
Retrohippocampal region -Somatosensory areas	-0.05138	0	0	0
Entorhinal area-Somatosensory areas	-0.03474	-0.0024	-0.05587	-0.05156
Field CA1-Somatosensory areas	0	0	0	0
Field CA3--Somatosensory areas	0	0	0	0
Dentate gyrus-Somatosensory areas	0	0	0	0
Field CA2 -Somatosensory areas	0	0	0	0
Accessory olfactory bulb mitral layer-Somatosensory areas	0	0	0	0
Striatum --Somatosensory areas	0	0	0	-0.00172
Midbrain --Somatosensory areas	0	0	0	0
Medulla-Somatosensory areas	0	0	0	0
Taenia tecta -piriform area	0	0	0	-0.01073
Accessory olfactory bulb glomerular layer-piriform area	0	0	0	0
Accessory olfactory bulb granular layer-piriform area	0	0	0	0
Retrohippocampal region -piriform area	-0.2447	0	-0.13816	0.440821
Entorhinal area-piriform area	-0.10671	-0.58915	-0.05348	-0.64353
Field CA1-piriform area	0.132047	0	-0.01937	0.131745
Field CA3-piriform area	-0.34443	0	0	0.119301
Dentate gyrus-piriform area	-0.31648	0	-0.09844	0.06055
Field CA2 -piriform area	-0.26936	0	0	0.05293
Accessory olfactory bulb mitral layer-piriform area	0	0	0	0
Striatum -piriform area	-0.01456	0	-0.15707	-0.70512
Midbrain -piriform area	-0.02313	0	0	0

Medulla-piriform area	0	0	0	0
Accessory olfactory bulb glomerular layer-Taenia tecta	0	0	0	0
Accessory olfactory bulb granular layer-Taenia tecta	0	0	0	0
Retrohippocampal region -Taenia tecta	-0.06929	-0.08695	0	-0.04333
Entorhinal area-Taenia tecta	-0.00198	-0.00369	-0.02762	-0.01115
Field CA1-Taenia tecta	0	0	0	-0.03578
Field CA3-Taenia tecta	0	0	0	0
Dentate gyrus-Taenia tecta	0	0	0	-0.20837
Field CA2 -Taenia tecta	0	0	0	0
Accessory olfactory bulb mitral layer-Taenia tecta	0	0	0	0
Striatum -Taenia tecta	0	-0.11378	0	0
Midbrain -Taenia tecta	0	0	0	0
Medulla-Taenia tecta	0	0	0	0
Accessory olfactory bulb granular layer-Accessory olfactory bulb glomerular layer	0	0	0	0
Retrohippocampal region -Accessory olfactory bulb glomerular layer	0	0	0	0
Entorhinal area-Accessory olfactory bulb glomerular layer	0	0	0	-0.2822
Field CA1-Accessory olfactory bulb glomerular layer	0	0	0	0
Field CA3-Accessory olfactory bulb glomerular layer	0	0	0	0
Dentate gyrus-Accessory olfactory bulb glomerular layer	0	0	0	0
Field CA2 -Accessory olfactory bulb glomerular layer	0	0	0	0
Accessory olfactory bulb mitral layer-Accessory olfactory bulb glomerular layer	0	0	0	0
Striatum -Accessory olfactory bulb glomerular layer	0	0	0	0
Midbrain -Accessory olfactory bulb glomerular layer	0	0	0	0
Medulla-Accessory olfactory bulb glomerular layer	0	0	0	0
Retrohippocampal region -Accessory olfactory bulb granular layer	0	0	0	0
Entorhinal area-Accessory olfactory bulb granular layer	0	0	0	-0.07601
Field CA1-Accessory olfactory bulb granular layer	0	0	0	0
Field CA3-Accessory olfactory bulb granular layer	0	0	0	0
Dentate gyrus-Accessory olfactory bulb granular layer	0	0	0	0
Field CA2 -Accessory olfactory bulb granular layer	0	0	0	0
Accessory olfactory bulb mitral layer-Accessory olfactory bulb granular layer	0	0	0	0
Striatum -Accessory olfactory bulb granular layer	0	0	0	0

Midbrain -Accessory olfactory bulb granular layer	0	0	0	0
Medulla-Accessory olfactory bulb granular layer	0	0	0	0
Entorhinal area-Retrohippocampal region	0.048246	-0.01533	-0.01098	0.062422
Field CA1-Retrohippocampal region	0.499804	0	-0.31545	0.409291
Field CA3-Retrohippocampal region	-0.82067	0	0	-0.62847
Dentate gyrus-Retrohippocampal region	0.148018	-0.61875	0	0.119301
Field CA2 -Retrohippocampal region	0	0	0	-0.92061
Accessory olfactory bulb mitral layer-Retrohippocampal region	0	0	0	0
Striatum -Retrohippocampal region	-0.00373	-0.10459	-0.01178	-0.21747
Midbrain -Retrohippocampal region	-0.13205	0.613098	0	0.643531
Medulla-Retrohippocampal region	0	0	0.602959	0
Field CA1-Entorhinal area	0.136571	-0.00369	-0.00163	0.200091
Field CA3-Entorhinal area	-0.56631	-0.02048	-0.0045	-0.87617
Dentate gyrus-Entorhinal area	0.536548	-0.0935	-0.00176	0.040839
Field CA2 -Entorhinal area	-0.04124	-0.00369	-0.01652	-0.87617
Accessory olfactory bulb mitral layer-Entorhinal area	0	0	0	-0.12528
Striatum -Entorhinal area	-0.02236	-0.00374	-0.00643	-0.05156
Midbrain -Entorhinal area	-0.00835	-0.15526	-0.1393	-0.36028
Medulla-Entorhinal area	0.269358	-0.10657	-0.51992	-0.18019
Field CA3-Field CA1	0	0	0	-0.47251
Dentate gyrus-Field CA1	0.198254	0	0	-0.16686
Field CA2 -Field CA1	0	0	0	0
Accessory olfactory bulb mitral layer-Field CA1	0	0	0	0
Striatum -Field CA1	-0.02193	0	-0.05587	-0.01614
Midbrain -Field CA1	0	0	0	0
Medulla-Field CA1	0	0	0	0
Dentate gyrus-Field CA3	0	0	0	-0.37989
Field CA2 -Field CA3	0	0	0	0
Accessory olfactory bulb mitral layer-Field CA3	0	0	0	0
Striatum -Field CA3	-0.12746	0	0	-0.10029
Midbrain -Field CA3	0	0	0	0
Medulla-Field CA3	0	0	0	0

Field CA2 -Dentate gyrus	0	0	0	0
Accessory olfactory bulb mitral layer-Dentate gyrus	0	0	0	0
Striatum -Dentate gyrus	-0.04662	-0.07341	0	-0.09012
Midbrain -Dentate gyrus	0	0	0	-0.29543
Medulla-Dentate gyrus	0	0	0	0
Accessory olfactory bulb mitral layer-Field CA2	0	0	0	0
Striatum -Field CA2	0	0	0	0
Midbrain -Field CA2	0	0	0	0
Medulla-Field CA2	0	0	0	0
Striatum -Accessory olfactory bulb mitral layer	0	0	0	0
Midbrain -Accessory olfactory bulb mitral layer	0	0	0	0
Medulla-Accessory olfactory bulb mitral layer	0	0	0	0
Midbrain -Striatum	0	0	0	-0.0357
Medulla-Striatum	0	0	0	0
Medulla-Midbrain	0	0	0	0

S11. Adjust p values for group comparisons of pair-wised connectivity for Hypothalamus.

Region Connections	Hypothalamus			
	Left Ipsilateral	Left Contralateral	Right Contralateral	Right Ipsilateral
Caudoputamen-corpus callosum	-0.0018	-0.00394	-0.00323	-0.07303
anterior commissure olfactory limb-corpus callosum	0	0	0	0
pallidum -corpus callosum	-0.37586	-0.01869	-0.00959	-0.0454
internal capsule-corpus callosum	-0.33521	-0.32178	-0.0692	-0.03071
Thalamus-corpus callosum	-0.02367	-0.02032	-0.01481	-0.25197
Cerebellum-corpus callosum	-0.00254	-0.00365	-0.00481	-0.00402
Superior colliculus-corpus callosum	-0.00438	-0.07381	-0.00683	-0.00499
ventricular systems-corpus callosum	-0.00542	-0.00499	-0.0078	-0.00671
Hypothalamus -corpus callosum	-0.00892	-0.00737	-0.02061	-0.10224
Inferior colliculus -corpus callosum	-0.00137	-0.04613	-0.00955	-0.00275
periaqueductal gray-corpus callosum	-0.00116	-0.00435	-0.00241	-0.00201
Isocortex -corpus callosum	-0.00172	-0.01634	-0.00336	-0.00775
Cortical amygdalar area -corpus callosum	0.48304	0	-0.1185	0.465205
Olfactory areas -corpus callosum	0.664336	-0.04214	-0.09755	0.689279
Pons-corpus callosum	-0.00116	-0.00263	-0.00283	-0.00156
Midbrain reticular nucleus-corpus callosum	-0.00048	-0.00887	-0.04016	-0.00511
Nucleus accumbens-corpus callosum	-0.16975	-0.06686	0	-0.06159
fimbria-corpus callosum	0	0	0	0
Anterior cingulate area-corpus callosum	0	0	0	0
Somatomotor areas-corpus callosum	-0.09106	-0.35647	0	0
Somatosensory areas-corpus callosum	-0.00761	-0.14137	-0.09755	-0.11124
piriform area-corpus callosum	0	0	0	0
Taenia tecta -corpus callosum	-0.05531	-0.62366	-0.0383	0.897278
Accessory olfactory bulb glomerular layer-corpus callosum	0	0	0	0
Accessory olfactory bulb granular layer-corpus callosum	0	0	0	0
Retrohippocampal region -corpus callosum	-0.00156	-0.08025	-0.0078	-0.01863
Entorhinal area-corpus callosum	-0.04528	0	-0.00336	-0.00499
Field CA1-corpus callosum	-0.00116	-0.01772	-0.01299	-0.01818
Field CA3-corpus callosum	-0.00521	-0.02686	-0.00368	-0.00585
Dentate gyrus-corpus callosum	-0.00552	-0.02528	-0.00336	-0.04672
Field CA2 -corpus callosum	0	0	0	0
Accessory olfactory bulb mitral layer-corpus callosum	0	0	0	0

Striatum -corpus callosum	-0.97078	-0.15045	-0.03347	0.758428
Midbrain -corpus callosum	-0.00116	-0.00263	-0.00241	-0.00086
Medulla-corpus callosum	-0.00156	-0.00435	-0.0078	-0.01789
anterior commissure olfactory limb-Caudoputamen	0	0	0.187791	0
pallidum -Caudoputamen	0.003906	-0.008509	-0.566328	-0.199659
internal capsule-Caudoputamen	0.852107	-0.686026	-0.141509	-0.134196
Thalamus-Caudoputamen	-0.420955	-0.065858	-0.048887	0.685066
Cerebellum-Caudoputamen	-0.011487	-0.002632	-0.00708	-0.010912
Superior colliculus-Caudoputamen	-0.025365	-0.018803	-0.026835	-0.018184
ventricular systems-Caudoputamen	-0.054449	-0.006745	-0.058132	-0.006116
Hypothalamus -Caudoputamen	0.65523	-0.002632	-0.594018	-0.636527
Inferior colliculus -Caudoputamen	-0.005772	-0.00365	-0.011891	-0.0095
periaqueductal gray-Caudoputamen	-0.002715	-0.004373	-0.002826	-0.002753
Isocortex -Caudoputamen	-0.012037	-0.006295	-0.00336	-0.008284
Cortical amygdalar area -Caudoputamen	0.205008	0	-0.873969	0.321007
Olfactory areas -Caudoputamen	0.265569	-0.176582	-0.118464	0.497894
Pons-Caudoputamen	-0.001314	-0.00365	-0.002622	-0.000857
Midbrain reticular nucleus-Caudoputamen	-0.002541	-0.008384	-0.010206	-0.0095
Nucleus accumbens-Caudoputamen	0.310407	-0.485144	-0.065176	-0.450705
fimbria-Caudoputamen	0	0	0	0
Anterior cingulate area-Caudoputamen	0	0	0	0
Somatomotor areas-Caudoputamen	-0.025435	-0.112865	-0.034466	-0.153586
Somatosensory areas-Caudoputamen	-0.060468	-0.037784	-0.03629	-0.105305
piriform area-Caudoputamen	0.784195	-0.081227	-0.632641	0
Taenia tecta -Caudoputamen	0.259406	0.071982	-0.408153	0.373902
Accessory olfactory bulb glomerular layer-Caudoputamen	0	0	0	0
Accessory olfactory bulb granular layer-Caudoputamen	0	0	0	0
Retrohippocampal region -Caudoputamen	-0.008922	-0.004345	-0.010149	-0.002064
Entorhinal area-Caudoputamen	-0.059114	-0.009333	-0.003289	-0.002387
Field CA1-Caudoputamen	-0.002648	-0.004659	-0.00708	-0.006809
Field CA3-Caudoputamen	-0.047901	-0.00365	-0.004089	-0.006809
Dentate gyrus-Caudoputamen	-0.025435	-0.00365	-0.007804	-0.055274
Field CA2 -Caudoputamen	-0.012214	0	-0.040723	0
Accessory olfactory bulb mitral layer-Caudoputamen	0	0	0	0
Striatum -Caudoputamen	0.042322	-0.026862	-0.611958	0.178077
Midbrain -Caudoputamen	-0.002446	-0.002632	-0.003547	-0.001595

Medulla-Caudoputamen	-0.001314	-0.00365	-0.00446	-0.003669
pallidum -anterior commissure olfactory limb	0.080734	0.004345	-0.179459	0.096847
internal capsule-anterior commissure olfactory limb	0	0	0	0
Thalamus-anterior commissure olfactory limb	0.205008	0	0	0
Cerebellum-anterior commissure olfactory limb	0	0	0	0
Superior colliculus-anterior commissure olfactory limb	0	0	0	0
ventricular systems-anterior commissure olfactory limb	0.85552	0.287262	-0.934105	-0.28742
Hypothalamus -anterior commissure olfactory limb	0.006594	0.18327	0.179459	0.098959
Inferior colliculus -anterior commissure olfactory limb	0	0	0	0
periaqueductal gray-anterior commissure olfactory limb	0	0	0	0
Isocortex -anterior commissure olfactory limb	-0.319326	-0.726227	0	-0.727028
Cortical amygdalar area -anterior commissure olfactory limb	0	0	0	0
Olfactory areas -anterior commissure olfactory limb	0	-0.096498	-0.754221	0
Pons-anterior commissure olfactory limb	0	0	0	0
Midbrain reticular nucleus-anterior commissure olfactory limb	0	0	0	0
Nucleus accumbens-anterior commissure olfactory limb	0.030684	-0.187805	-0.08281	0.009414
fimbria-anterior commissure olfactory limb	0	0	0	0
Anterior cingulate area-anterior commissure olfactory limb	0	0	0	0
Somatomotor areas-anterior commissure olfactory limb	0	0	0	0
Somatosensory areas-anterior commissure olfactory limb	0	0	0	0
piriform area-anterior commissure olfactory limb	0	0	0	0
Taenia tecta -anterior commissure olfactory limb	0	0	0.183381	0
Accessory olfactory bulb glomerular layer-anterior commissure olfactory limb	0	0	0	0
Accessory olfactory bulb granular layer-anterior commissure olfactory limb	0	0	0	0
Retrohippocampal region -anterior commissure olfactory limb	0	0	0	0
Entorhinal area-anterior commissure olfactory limb	0	0	0	0
Field CA1-anterior commissure olfactory limb	0	0	0	0
Field CA3-anterior commissure olfactory limb	-0.181612	0	0	0
Dentate gyrus-anterior commissure olfactory limb	0	0	0	0
Field CA2 -anterior commissure olfactory limb	0	0	0	0
Accessory olfactory bulb mitral layer-anterior commissure olfactory limb	0	0	0	0
Striatum -anterior commissure olfactory limb	0.013294	0.117056	-0.592995	0.013654
Midbrain -anterior commissure olfactory limb	0	-0.244073	0	-0.012365
Medulla-anterior commissure olfactory limb	0	0	0	0

internal capsule-pallidum	0.291937	-0.45596	-0.211597	0.092749
Thalamus-pallidum	0.070698	0.904843	-0.566328	0.092749
Cerebellum-pallidum	-0.103793	-0.005978	-0.013545	-0.007096
Superior colliculus-pallidum	-0.038443	-0.513824	-0.060091	-0.030956
ventricular systems-pallidum	-0.102163	-0.189611	-0.024664	-0.002311
Hypothalamus -pallidum	0.696618	-0.030483	-0.570279	-0.535928
Inferior colliculus -pallidum	-0.013294	-0.067668	-0.065176	-0.009445
periaqueductal gray-pallidum	-0.005951	-0.02492	-0.006856	-0.0095
Isocortex -pallidum	-0.013294	-0.029967	-0.020112	-0.005578
Cortical amygdalar area -pallidum	0.030554	0	-0.961161	0.361053
Olfactory areas -pallidum	0.020527	-0.104385	0.292706	0.019395
Pons-pallidum	-0.002541	-0.00365	-0.00336	-0.001595
Midbrain reticular nucleus-pallidum	-0.005772	-0.019629	-0.066577	-0.005853
Nucleus accumbens-pallidum	-0.793007	-0.117056	-0.050298	-0.496074
fimbria-pallidum	-0.047796	0	-0.067215	-0.003279
AVA-pallidum	-0.132346	0	0	0
Somatomotor areas-pallidum	-0.784195	-0.771576	-0.781441	-0.092749
Somatosensory areas-pallidum	0.228716	-0.137652	-0.457094	-0.189529
piriform area-pallidum	0.046199	-0.177393	-0.040413	-0.912362
Taenia tecta -pallidum	0.004479	0.070396	0.040515	0.08994
Accessory olfactory bulb glomerular layer-pallidum	0	0	0	0
Accessory olfactory bulb granular layer-pallidum	0	0	0	0
Retrohippocampal region -pallidum	-0.014012	-0.013899	-0.061387	-0.003279
Entorhinal area-pallidum	-0.005457	-0.018478	-0.01011	-0.001977
Field CA1-pallidum	-0.008887	-0.008317	-0.007804	-0.009719
Field CA3-pallidum	-0.122474	-0.008317	-0.006856	-0.004533
Dentate gyrus-pallidum	-0.021203	-0.033794	-0.016362	-0.018556
Field CA2 -pallidum	-0.019453	-0.040626	-0.0113	-0.002374
Accessory olfactory bulb mitral layer-pallidum	0	0	0	0
Striatum -pallidum	0.021213	0.88566	0.018667	0.013015
Midbrain -pallidum	-0.002715	-0.004757	-0.005386	-0.002014
Medulla-pallidum	-0.00563	-0.004937	-0.008992	-0.003726
Thalamus-internal capsule	0.028207	0.023175	0.874121	0.002461
Cerebellum-internal capsule	0.045284	-0.821185	-0.423928	0.043935
Superior colliculus-internal capsule	0.878575	0.024692	0.400686	-0.100238

ventricular systems-internal capsule	0.127806	-0.442363	-0.381272	-0.088717
Hypothalamus -internal capsule	0.202406	-0.427801	0.020838	0.092749
Inferior colliculus -internal capsule	-0.002446	0	0	-0.023237
periaqueductal gray-internal capsule	-0.005477	0.977714	-0.133641	-0.059647
Isocortex -internal capsule	-0.190555	-0.202268	-0.123946	-0.044525
Cortical amygdalar area -internal capsule	0	0	0	0
Olfactory areas -internal capsule	0.578136	0	0	0.018184
Pons-internal capsule	-0.002446	-0.420752	-0.247386	-0.001977
Midbrain reticular nucleus-internal capsule	-0.254254	0	0	-0.992157
Nucleus accumbens-internal capsule	0	0	0	0
fimbria-internal capsule	0	0	0	0
Anterior cingulate area-internal capsule	0	0	0	0
Somatomotor areas-internal capsule	0	0	0	0
Somatosensory areas-internal capsule	0.31032	0	0	-0.45867
piriform area-internal capsule	0	0	0	0
Taenia tecta -internal capsule	0	0	0	0
Accessory olfactory bulb glomerular layer-internal capsule	0	0	0	0
Accessory olfactory bulb granular layer-internal capsule	0	0	0	0
Retrohippocampal region -internal capsule	0.979253	0	0	-0.128379
Entorhinal area-internal capsule	0	0	0	0
Field CA1-internal capsule	0.469673	0	0	-0.566453
Field CA3-internal capsule	0.067108	0	-0.260262	-0.310511
Dentate gyrus-internal capsule	0.494534	0	0	0.636527
Field CA2 -internal capsule	0	0	0	0
Accessory olfactory bulb mitral layer-internal capsule	0	0	0	0
Striatum -internal capsule	0.125032	-0.98061	-0.436572	0.012716
Midbrain --internal capsule	-0.068322	-0.68534	-0.551389	-0.164549
Medulla-internal capsule	-0.016898	0	-0.272404	0
Cerebellum-Thalamus	0.005951	-0.87303	0.674309	0.269022
Superior colliculus-Thalamus	0.319965	0.00365	-0.28129	-0.17186
ventricular systems-Thalamus	0.088729	-0.39867	-0.08057	-0.04741
Hypothalamus -Thalamus	0.011978	-0.7519	0.008728	0.006085
Inferior colliculus -Thalamus	-0.04093	-0.19236	0.983316	-0.68928
periaqueductal gray-Thalamus	-0.04514	0.751904	-0.01817	-0.02682
Isocortex -Thalamus	-0.04552	-0.11999	-0.05742	-0.03071

Cortical amygdalar area -Thalamus	0.148777	0	0.713841	0.026824
Olfactory areas -Thalamus	-0.61785	-0.64734	0.331737	0.149345
Pons-Thalamus	-0.01824	-0.01712	-0.01189	-0.00328
Midbrain reticular nucleus-Thalamus	-0.88465	-0.5812	0.90683	0.815245
Nucleus accumbens-Thalamus	-0.64584	-0.53485	0.968145	-0.90191
fimbria-Thalamus	-0.08515	0	-0.04072	-0.00297
Anterior cingulate area-Thalamus	-0.01625	-0.066	0	-0.09098
Somatomotor areas-Thalamus	-0.10565	-0.47662	-0.71364	-0.14954
Somatosensory areas-Thalamus	0.316396	-0.29829	-0.40557	-0.14034
piriform area-Thalamus	-0.51398	0.94981	0.328719	0.08994
Taenia tecta -Thalamus	0.07394	0.183718	0.093576	0.288054
Accessory olfactory bulb glomerular layer-Thalamus	0	0	0	0
Accessory olfactory bulb granular layer-Thalamus	0	0	0	0
Retrohippocampal region -Thalamus	-0.50102	-0.05003	-0.07258	-0.00699
Entorhinal area-Thalamus	-0.21128	-0.02686	-0.00419	-0.02418
Field CA1-Thalamus	-0.14123	-0.02867	-0.09027	-0.0137
Field CA3-Thalamus	0.076571	-0.42466	-0.05638	-0.33103
Dentate gyrus-Thalamus	0.634272	-0.44845	-0.33704	0.989439
Field CA2 -Thalamus	-0.09887	-0.19287	-0.0862	-0.01111
Accessory olfactory bulb mitral layer-Thalamus	0	0	0	0
Striatum -Thalamus	0.696618	-0.48514	0.570279	0.02597
Midbrain -Thalamus	-0.12781	-0.37593	-0.28133	-0.0095
Medulla-Thalamus	-0.02561	-0.00704	-0.01832	-0.00499
Superior colliculus-Cerebellum	0.015987	0.023185	-0.38127	-0.14351
ventricular systems-Cerebellum	0.386295	-0.11937	-0.35677	-0.06897
Hypothalamus -Cerebellum	0.005772	-0.40694	0.006834	0.018626
Inferior colliculus -Cerebellum	-0.37932	-0.55564	0.921696	-0.97649
periaqueductal gray-Cerebellum	0.959462	-0.6536	-0.36102	-0.14452
Isocortex -Cerebellum	-0.04993	-0.02851	-0.01047	-0.0095
Cortical amygdalar area -Cerebellum	0.004849	0.08856	-0.9217	0.154099
Olfactory areas -Cerebellum	-0.10579	-0.03778	-0.03928	-0.18282
Pons-Cerebellum	-0.17318	-0.38596	-0.26026	-0.26775
Midbrain reticular nucleus-Cerebellum	0.85552	-0.96622	0	-0.59883
Nucleus accumbens-Cerebellum	-0.08073	-0.15963	-0.08158	-0.11558
fimbria-Cerebellum	0	0	0	0

Anterior cingulate area-Cerebellum	-0.00245	-0.11999	-0.01299	-0.0181
Somatomotor areas-Cerebellum	-0.00892	-0.101	-0.01189	-0.00581
Somatosensory areas-Cerebellum	-0.95214	-0.03589	-0.03306	-0.00451
piriform area-Cerebellum	-0.71304	-0.07823	0.605853	0.179178
Taenia tecta -Cerebellum	-0.19167	-0.24407	-0.06232	-0.02682
Accessory olfactory bulb glomerular layer-Cerebellum	0	0	0	0
Accessory olfactory bulb granular layer-Cerebellum	0	0	0	0
Retrohippocampal region -Cerebellum	-0.08977	-0.15045	-0.03379	-0.0363
Entorhinal area-Cerebellum	-0.12374	-0.0139	-0.01355	-0.02357
Field CA1-Cerebellum	-0.09737	-0.04063	-0.01907	-0.01683
Field CA3-Cerebellum	0.004379	-0.37593	-0.06345	0.25225
Dentate gyrus-Cerebellum	0.001163	-0.62422	-0.32872	0.0223
Field CA2 -Cerebellum	-0.00948	-0.00494	0	-0.08994
Accessory olfactory bulb mitral layer-Cerebellum	0	0	0	0
Striatum -Cerebellum	-0.52609	-0.07185	-0.07348	-0.72515
Midbrain -Cerebellum	0.011464	-0.68388	0.707165	0.707506
Medulla-Cerebellum	-0.13537	-0.20382	-0.15749	-0.16154
ventricular systems-Superior colliculus	0.098173	-0.1841	-0.65104	-0.02682
Hypothalamus -Superior colliculus	0.016898	-0.4775	0.00336	-0.89728
Inferior colliculus -Superior colliculus	-0.7842	0	0.165365	-0.17804
periaqueductal gray-Superior colliculus	0.302146	-0.19875	0.064029	-0.05159
Isocortex -Superior colliculus	-0.0172	-0.01553	-0.03928	-0.03391
Cortical amygdalar area -Superior colliculus	0.039906	0	0.468141	-0.98921
Olfactory areas -Superior colliculus	-0.15277	-0.06114	-0.23641	-0.07881
Pons-Superior colliculus	-0.2346	-0.28774	-0.11413	-0.01365
Midbrain reticular nucleus-Superior colliculus	0.793007	0	0.098376	-0.11124
Nucleus accumbens-Superior colliculus	-0.52437	-0.5135	-0.26844	-0.44291
fimbria-Superior colliculus	0	0	0	-0.02522
Anterior cingulate area-Superior colliculus	0	0	-0.02969	0
Somatomotor areas-Superior colliculus	-0.04036	-0.07848	-0.23742	-0.04703
Somatosensory areas-Superior colliculus	-0.1867	-0.12845	-0.09027	-0.09828
piriform area-Superior colliculus	0.965228	0	0.065176	0.231532
Taenia tecta -Superior colliculus	0.611921	-0.33458	-0.40871	-0.07826
Accessory olfactory bulb glomerular layer-Superior colliculus	0	0	0	0
Accessory olfactory bulb granular layer-Superior colliculus	0	0	0	0

Retrohippocampal region -Superior colliculus	-0.04882	-0.10634	-0.40319	-0.03309
Entorhinal area-Superior colliculus	-0.08481	-0.19875	-0.1283	-0.01897
Field CA1-Superior colliculus	-0.0685	-0.05779	-0.67431	-0.03759
Field CA3-Superior colliculus	0.338349	-0.2476	-0.33814	-0.05876
Dentate gyrus-Superior colliculus	0.843536	-0.07055	0.291937	-0.18054
Field CA2 -Superior colliculus	0	-0.14476	0	-0.14469
Accessory olfactory bulb mitral layer-Superior colliculus	0	0	0	0
Striatum -Superior colliculus	-0.08873	-0.02978	0.605853	-0.10024
Midbrain -Superior colliculus	0.291937	-0.17853	0.020357	-0.02847
Medulla-Superior colliculus	-0.02695	-0.06188	-0.1031	-0.03886
Hypothalamus -ventricular systems	-0.045515	-0.02492	-0.138236	-0.008284
Inferior colliculus -ventricular systems	-0.024679	-0.033794	-0.018667	-0.008203
periaqueductal gray-ventricular systems	-0.334957	-0.067447	-0.022194	-0.002753
Isocortex -ventricular systems	-0.008506	-0.007964	-0.010468	-0.004016
Cortical amygdalar area -ventricular systems	0.376598	-0.333445	-0.773551	-0.594144
Olfactory areas -ventricular systems	-0.13357	-0.183718	-0.271826	-0.430029
Pons-ventricular systems	-0.015987	-0.012073	-0.003363	-0.002753
Midbrain reticular nucleus-ventricular systems	0.57302	-0.247598	0.669257	-0.005826
Nucleus accumbens-ventricular systems	-0.256782	-0.181981	-0.093576	-0.024362
fimbria-ventricular systems	-0.003976	-0.007403	-0.005959	-0.000857
Anterior cingulate area-ventricular systems	-0.005951	-0.020943	-0.007804	-0.023959
Somatomotor areas-ventricular systems	-0.050667	-0.065998	-0.033789	-0.019099
Somatosensory areas-ventricular systems	-0.149369	-0.074966	-0.051247	-0.024162
piriform area-ventricular systems	-0.35647	-0.077159	0.91994	0.951199
Taenia tecta -ventricular systems	0.255348	0.78214	0.906734	-0.245282
Accessory olfactory bulb glomerular layer-ventricular systems	0	0	0	0
Accessory olfactory bulb granular layer-ventricular systems	0	0	0	0
Retrohippocampal region -ventricular systems	-0.01824	-0.012073	-0.003547	-0.002014
Entorhinal area-ventricular systems	-0.081876	-0.015633	-0.003547	-0.002014
Field CA1-ventricular systems	-0.008922	-0.00365	-0.00336	-0.004509
Field CA3-ventricular systems	-0.038587	-0.015526	-0.011891	-0.007881
Dentate gyrus-ventricular systems	-0.089707	-0.024692	-0.059041	-0.019395
Field CA2 -ventricular systems	-0.004453	-0.018803	-0.01686	-0.019254
Accessory olfactory bulb mitral layer-ventricular systems	0	0	0	0
Striatum -ventricular systems	-0.016307	-0.026133	-0.234186	-0.075833

Midbrain -ventricular systems	-0.379324	-0.035891	-0.020609	-0.002374
Medulla-ventricular systems	-0.006328	-0.004994	-0.010149	-0.0095
Inferior colliculus -Hypothalamus	-0.036414	-0.143973	-0.216247	-0.267973
periaqueductal gray-Hypothalamus	-0.208832	0.127745	-0.06282	-0.184456
Isocortex -Hypothalamus	-0.005017	-0.136543	-0.011303	-0.01452
Cortical amygdalar area -Hypothalamus	0.048818	0.049067	-0.128898	0.030583
Olfactory areas -Hypothalamus	0.055311	-0.821451	0.876941	0.053118
Pons-Hypothalamus	-0.021766	-0.391131	-0.010206	-0.006985
Midbrain reticular nucleus-Hypothalamus	0.960515	0.073812	-0.669839	0.033001
Nucleus accumbens-Hypothalamus	-0.324799	-0.364555	-0.033789	-0.148601
fimbria-Hypothalamus	-0.010444	-0.128451	-0.00368	-0.001661
Anterior cingulate area-Hypothalamus	-0.001422	-0.045065	-0.002622	-0.011238
Somatomotor areas-Hypothalamus	-0.067108	-0.581201	-0.051247	-0.061138
Somatosensory areas-Hypothalamus	0.634272	-0.769437	-0.024307	-0.061138
piriform area-Hypothalamus	0.326282	-0.716426	-0.773551	0.113713
Taenia tecta -Hypothalamus	0.008887	0.093607	0.132542	0.128379
Accessory olfactory bulb glomerular layer-Hypothalamus	0	0	0	0
Accessory olfactory bulb granular layer-Hypothalamus	0	0	0	0
Retrohippocampal region -Hypothalamus	-0.0961	-0.247598	-0.003531	-0.004457
Entorhinal area-Hypothalamus	-0.097373	-0.336266	-0.002622	-0.005853
Field CA1-Hypothalamus	-0.002446	-0.060275	-0.005979	-0.0095
Field CA3-Hypothalamus	-0.110435	-0.642436	-0.011891	-0.014074
Dentate gyrus-Hypothalamus	-0.272624	-0.649637	-0.043035	-0.154099
Field CA2 -Hypothalamus	-0.004859	-0.053449	-0.004089	-0.008764
Accessory olfactory bulb mitral layer-Hypothalamus	0	0	0	0
Striatum -Hypothalamus	0.621191	-0.183718	-0.052249	0.245723
Midbrain -Hypothalamus	-0.634272	0.070396	-0.118464	0.067007
Medulla-Hypothalamus	-0.009037	-0.035891	-0.007804	-0.008284
periaqueductal gray-Inferior colliculus	-0.37735	-0.53485	0	-0.04105
Isocortex -Inferior colliculus	-0.01141	-0.14951	-0.05427	-0.01496
Cortical amygdalar area -Inferior colliculus	0	0	0	0
Olfactory areas -Inferior colliculus	-0.03055	-0.55545	-0.17781	-0.12121
Pons-Inferior colliculus	-0.19167	-0.28836	0	-0.11103
Midbrain reticular nucleus-Inferior colliculus	0	0	0	0

Nucleus accumbens-Inferior colliculus	0	0	0	0
fimbria-Inferior colliculus	0	0	0	0
Anterior cingulate area-Inferior colliculus	0	0	0	0
Somatomotor areas-Inferior colliculus	-0.01746	0	0	-0.01818
Somatosensory areas-Inferior colliculus	-0.00616	-0.48857	0	-0.05325
piriform area-Inferior colliculus	0	0	0	0
Taenia tecta -Inferior colliculus	0	0	0	0
Accessory olfactory bulb glomerular layer-Inferior colliculus	0	0	0	0
Accessory olfactory bulb granular layer-Inferior colliculus	0	0	0	0
Retrohippocampal region -Inferior colliculus	-0.33722	-0.82145	0	-0.08852
Entorhinal area-Inferior colliculus	-0.10642	0	0	-0.11872
Field CA1-Inferior colliculus	-0.46414	0	0	-0.10554
Field CA3-Inferior colliculus	0	0	0	-0.09302
Dentate gyrus-Inferior colliculus	-0.38646	-0.94951	-0.66926	-0.9927
Field CA2 -Inferior colliculus	0	0	0	0
Accessory olfactory bulb mitral layer-Inferior colliculus	0	0	0	0
Striatum -Inferior colliculus	-0.00548	-0.0662	-0.06518	-0.10728
Midbrain -Inferior colliculus	-0.03733	-0.68631	-0.0358	-0.06693
Medulla-Inferior colliculus	0	0	0	-0.23504
Isocortex -periaqueductal gray	-0.00272	-0.00365	-0.00708	-0.00402
Cortical amygdalar area -periaqueductal gray	0	0	0	-0.14469
Olfactory areas -periaqueductal gray	-0.00254	-0.02044	-0.00992	-0.01856
Pons-periaqueductal gray	-0.10961	-0.06323	-0.07289	-0.00954
Midbrain reticular nucleus-periaqueductal gray	-0.79301	-0.13128	0	-0.09656
Nucleus accumbens-periaqueductal gray	-0.06375	-0.29829	-0.09027	-0.4373
fimbria-periaqueductal gray	0	0	0	-0.0026
Anterior cingulate area-periaqueductal gray	-0.00116	-0.00365	-0.00792	-0.00699
Somatomotor areas-periaqueductal gray	-0.00137	-0.03316	-0.01047	-0.0095
Somatosensory areas-periaqueductal gray	-0.00265	-0.00466	-0.01026	-0.01335
piriform area-periaqueductal gray	0	-0.13735	0	0.45867
Taenia tecta -periaqueductal gray	-0.16654	-0.07175	-0.07686	-0.05876
Accessory olfactory bulb glomerular layer-periaqueductal gray	0	0	0	0
Accessory olfactory bulb granular layer-periaqueductal gray	0	0	0	0
Retrohippocampal region -periaqueductal gray	-0.00889	-0.00435	-0.00708	-0.00463
Entorhinal area-periaqueductal gray	-0.00383	-0.01276	-0.00708	-0.00201

Field CA1-periaqueductal gray	-0.00438	-0.00365	-0.03885	-0.00206
Field CA3-periaqueductal gray	-0.12269	-0.00448	-0.07497	-0.01283
Dentate gyrus-periaqueductal gray	-0.05911	-0.0063	-0.07659	-0.02682
Field CA2 -periaqueductal gray	-0.00254	-0.00263	-0.06518	-0.04794
Accessory olfactory bulb mitral layer-periaqueductal gray	0	0	0	0
Striatum -periaqueductal gray	-0.00889	-0.01642	-0.06596	-0.02787
Midbrain -periaqueductal gray	-0.67923	-0.04063	-0.85897	-0.02682
Medulla-periaqueductal gray	-0.05511	-0.08653	-0.09759	-0.04672
Cortical amygdalar area -Isocortex	0.335205	-0.49742	-0.47698	-0.02445
Olfactory areas -Isocortex	-0.06238	-0.01634	-0.07869	-0.02255
Pons-Isocortex	-0.00245	-0.00605	-0.00336	-0.00201
Midbrain reticular nucleus-Isocortex	-0.00116	-0.00494	-0.00522	-0.01914
Nucleus accumbens-Isocortex	-0.0144	-0.02094	-0.04458	-0.03777
fimbria-Ictx	-0.0242	0	-0.00336	-0.00021
Anterior cingulate area-Isocortex	-0.02821	-0.02094	-0.01866	-0.01126
Somatomotor areas-Isocortex	-0.0206	-0.1742	-0.09755	-0.04672
Somatosensory areas-Isocortex	-0.05206	-0.02025	-0.07792	-0.03605
piriform area-Isocortex	-0.18034	-0.15672	-0.78474	-0.57873
Taenia tecta -Isocortex	-0.46306	-0.53853	-0.06282	-0.03941
Accessory olfactory bulb glomerular layer-Isocortex	0	0	0	0
Accessory olfactory bulb granular layer-Isocortex	0	0	0	0
Retrohippocampal region -Isocortex	-0.01627	-0.00804	-0.00409	-0.00201
Entorhinal area-Isocortex	-0.0253	-0.0117	-0.00992	-0.00402
Field CA1-Isocortex	-0.00577	-0.00598	-0.02312	-0.01496
Field CA3-Isocortex	-0.13812	-0.01642	-0.0113	-0.01407
Dentate gyrus-Isocortex	-0.47564	-0.01471	-0.12805	-0.04304
Field CA2 -Isocortex	-0.00865	-0.03589	-0.00992	-0.01407
Accessory olfactory bulb mitral layer-Isocortex	0	0	0	0
Striatum -Isocortex	-0.01134	-0.02608	-0.08714	-0.00945
Midbrain -Isocortex	-0.00254	-0.00494	-0.00708	-0.00206
Medulla-Isocortex	-0.0025	-0.00499	-0.00708	-0.00395
Olfactory areas -Cortical amygdalar area	0.843536	-0.73244	0	0.462769
Pons-Cortical amygdalar area	0	0	0	-0.0016
Midbrain reticular nucleus-Cortical amygdalar area	0	0	0	0

Nucleus accumbens-Cortical amygdalar area	0	0	0	-0.19385
fimbria-Cortical amygdalar area	0	0	0	-0.0095
Anterior cingulate area-Cortical amygdalar area	0	0	0	0
Somatomotor areas-Cortical amygdalar area	0	0	0	0
Somatosensory areas-Cortical amygdalar area	0.052061	0.671437	0	-0.53593
piriform area-Cortical amygdalar area	0	0	0	0
Taenia tecta -Cortical amygdalar area	0	0.750462	0	0.325304
Accessory olfactory bulb glomerular layer-Cortical amygdalar area	0	0	0	0
Accessory olfactory bulb granular layer-Cortical amygdalar area	0	0	0	0
Retrohippocampal region -Cortical amygdalar area	0.641349	0	0	0
Entorhinal area-Cortical amygdalar area	0	0	0	0
Field CA1-Cortical amygdalar area	0	0	0	-0.02682
Field CA3-Cortical amygdalar area	-0.51626	-0.24407	0	-0.04703
Dentate gyrus-Cortical amygdalar area	0.386295	-0.30208	0	-0.42937
Field CA2 -Cortical amygdalar area	0	0	0	0
Accessory olfactory bulb mitral layer-Cortical amygdalar area	0	0	0	0
Striatum -Cortical amygdalar area	0.414534	-0.90484	0.421679	0.451188
Midbrain -Cortical amygdalar area	0.179252	-0.36456	-0.84462	0.373854
Medulla-Cortical amygdalar area	0	0	0	0
Pons-Olfactory areas	-0.00649	-0.00624	-0.00336	-0.00206
Midbrain reticular nucleus-Olfactory areas	0	0	0	-0.25635
Nucleus accumbens-Olfactory areas	0.27402	-0.02896	-0.02576	0.617879
fimbria-Olfactory areas	0	0	-0.01501	0
Anterior cingulate area-Olfactory areas	0	0	0	0
Somatomotor areas-Olfactory areas	0	0.489966	-0.41819	-0.16154
Somatosensory areas-Olfactory areas	0.410598	-0.53485	-0.2534	-0.14452
piriform area-Olfactory areas	0	0.870632	0.961161	0
Taenia tecta -Olfactory areas	0.017425	0.095325	-0.91994	0.012365
Accessory olfactory bulb glomerular layer-Olfactory areas	0	0	0	0
Accessory olfactory bulb granular layer-Olfactory areas	0	0	0	0
Retrohippocampal region -Olfactory areas	-0.0028	-0.00624	-0.03362	-0.00304
Entorhinal area-Olfactory areas	-0.04514	-0.066	-0.01232	-0.00671
Field CA1-Olfactory areas	-0.00595	-0.01237	-0.04043	-0.04198
Field CA3-Olfactory areas	-0.13446	-0.02209	-0.01469	-0.00727
Dentate gyrus-Olfactory areas	-0.03629	-0.01462	-0.06754	-0.38087

Field CA2 -Olfactory areas	0	0	0	0
Accessory olfactory bulb mitral layer-Olfactory areas	0	0	0	0
Striatum -Olfactory areas	0.040984	0.759795	0.475453	0.055274
Midbrain -Olfactory areas	-0.00745	-0.00365	-0.01047	-0.00451
Medulla-Olfactory areas	-0.01417	-0.0226	-0.0275	-0.0095
Midbrain reticular nucleus-Pons	-0.17811	0	-0.59402	-0.00945
Nucleus accumbens-Pons	-0.03176	-0.03905	-0.07258	-0.03587
fimbria-Pons	-0.00865	0	0	-0.02847
Anterior cingulate area-Pons	-0.0076	0	0	-0.00451
Somatomotor areas-Pons	-0.01044	-0.01948	-0.02304	-0.00201
Somatosensory areas-Pons	-0.0025	-0.00365	-0.01383	-0.00206
piriform area-Pons	-0.07048	0	0	0
Taenia tecta -Pons	-0.01716	-0.02692	-0.01635	-0.00198
Accessory olfactory bulb glomerular layer-Pons	0	0	0	0
Accessory olfactory bulb granular layer-Pons	0	0	0	0
Retrohippocampal region -Pons	-0.00994	-0.00365	-0.0078	-0.0095
Entorhinal area-Pons	-0.01458	-0.01739	-0.00683	-0.00237
Field CA1-Pons	-0.00448	-0.00494	-0.03108	-0.0143
Field CA3-Pons	-0.03374	-0.00499	-0.01271	-0.01485
Dentate gyrus-Pons	-0.0212	-0.01968	-0.06726	-0.03135
Field CA2 -Pons	-0.01425	-0.00365	0	-0.10311
Accessory olfactory bulb mitral layer-Pons	0	0	0	0
Striatum -Pons	-0.00595	-0.00435	-0.00336	-0.00176
Midbrain -Pons	-0.02177	-0.03551	-0.02466	-0.00446
Medulla-Pons	-0.17811	-0.12338	-0.30227	-0.19057
Nucleus accumbens-Midbrain reticular nucleus	0	0	0	0
fimbria-Midbrain reticular nucleus	0	0	0	0
Anterior cingulate area-Midbrain reticular nucleus	0	0	0	0
Somatomotor areas-Midbrain reticular nucleus	-0.0217	0	0	0
Somatosensory areas-Midbrain reticular nucleus	-0.05571	-0.07716	-0.04965	0
piriform area-Midbrain reticular nucleus	0	0	0	0
Taenia tecta -Midbrain reticular nucleus	0	0	0	0
Accessory olfactory bulb glomerular layer-Midbrain reticular nucleus	0	0	0	0
Accessory olfactory bulb granular layer-Midbrain reticular nucleus	0	0	0	0

Retrohippocampal region -Midbrain reticular nucleus	-0.29359	0	0	-0.26196
Entorhinal area-Midbrain reticular nucleus	0	0	0	0
Field CA1-Midbrain reticular nucleus	-0.01385	0	0	-0.00323
Field CA3-Midbrain reticular nucleus	-0.5229	0	0	-0.9416
Dentate gyrus-Midbrain reticular nucleus	-0.78421	0	-0.38127	-0.91378
Field CA2 -Midbrain reticular nucleus	0	0	0	0
Accessory olfactory bulb mitral layer-Midbrain reticular nucleus	0	0	0	0
Striatum -Midbrain reticular nucleus	-0.03198	-0.16266	-0.01749	-0.53575
Midbrain -Midbrain reticular nucleus	-0.01047	0.746988	0.421285	-0.1541
Medulla-Midbrain reticular nucleus	0	0	-0.23419	-0.11622
fimbria-Nucleus accumbens	0	0	0	0
Anterior cingulate area-Nucleus accumbens	0	0	0	0
Somatomotor areas-Nucleus accumbens	0	0	0	0
Somatosensory areas-Nucleus accumbens	-0.41453	-0.20227	0	-0.35599
piriform area-Nucleus accumbens	0	0	0	0
Taenia tecta -Nucleus accumbens	0.074391	0	0.874121	0
Accessory olfactory bulb glomerular layer-Nucleus accumbens	0	0	0	0
Accessory olfactory bulb granular layer-Nucleus accumbens	0	0	0	0
Retrohippocampal region -Nucleus accumbens	-0.00878	0	-0.031	-0.0392
Entorhinal area-Nucleus accumbens	-0.01417	0	-0.02648	-0.01365
Field CA1-Nucleus accumbens	0	0	0	-0.10672
Field CA3-Nucleus accumbens	-0.08437	0	-0.19641	-0.09275
Dentate gyrus-Nucleus accumbens	-0.00485	0	-0.25878	-0.14548
Field CA2 -Nucleus accumbens	0	0	0	0
Accessory olfactory bulb mitral layer-Nucleus accumbens	0	0	0	0
Striatum -Nucleus accumbens	0.019002	-0.03905	-0.45709	0.03886
Midbrain -Nucleus accumbens	-0.01141	-0.04895	-0.0113	-0.01282
Medulla-Nucleus accumbens	-0.03352	0	-0.06913	-0.0926
Anterior cingulate area-fimbria	0	0	0	0
Somatomotor areas-fimbria	0	0	0	0
Somatosensory areas-fimbria	0	0	0	0
piriform area-fimbria	0	0	0	0
Taenia tecta -fimbria	0	0	0	0
Accessory olfactory bulb glomerular layer-fimbria	0	0	0	0

Accessory olfactory bulb granular layer-fimbria	0	0	0	0
Retrohippocampal region -fimbria	0	0	0	0
Entorhinal area-fimbria	0	0	0	-0.00572
Field CA1-fimbria	0	0	0	0
Field CA3-fimbria	0	0	0	-0.00237
Dentate gyrus-fimbria	0	0	0	-0.00237
Field CA2 -fimbria	0	0	0	0
Accessory olfactory bulb mitral layer-fimbria	0	0	0	0
Striatum -fimbria	-0.01359	0	0	-0.00021
Midbrain -fimbria	-0.00889	-0.00365	-0.00992	-0.00216
Medulla-fimbria	0	0	0	-0.14051
Somatomotor areas-Anterior cingulate area	0	0	0	0
Somatosensory areas-Anterior cingulate area	0	0	0	0
piriform area-Anterior cingulate area	0	0	0	0
Taenia tecta -Anterior cingulate area	0	0	0	0
Accessory olfactory bulb glomerular layer-Anterior cingulate area	0	0	0	0
Accessory olfactory bulb granular layer-Anterior cingulate area	0	0	0	0
Retrohippocampal region -Anterior cingulate area	0	0	0	0
Entorhinal area-Anterior cingulate area	0	0	0	0
Field CA1-Anterior cingulate area	0	0	0	0
Field CA3-Anterior cingulate area	0	0	0	0
Dentate gyrus-Anterior cingulate area	0	0	0	0
Field CA2 -Anterior cingulate area	0	0	0	0
Accessory olfactory bulb mitral layer-Anterior cingulate area	0	0	0	0
Striatum -Anterior cingulate area	-0.08515	-0.00494	0	-0.0016
Midbrain -Anterior cingulate area	-0.00131	-0.00499	-0.00241	-0.01097
Medulla-Anterior cingulate area	0	0	-0.03347	0
Somatosensory areas-Somatomotor areas	-0.69681	-0.24407	0	0
piriform area-Somatomotor areas	0	0	0	0
Taenia tecta -Somatomotor areas	0	0	0	0
Accessory olfactory bulb glomerular layer-Somatomotor areas	0	0	0	0
Accessory olfactory bulb granular layer-Somatomotor areas	0	0	0	0
Retrohippocampal region -Somatomotor areas	-0.08515	0	0	-0.10024
Entorhinal area-Somatomotor areas	0	0	-0.06989	-0.04741

Field CA1-Somatomotor areas	0	0	0	0
Field CA3-Somatomotor areas	0	0	-0.11268	0
Dentate gyrus-Somatomotor areas	-0.12154	0	-0.36183	-0.05422
Field CA2 -Somatomotor areas	0	0	0	0
Accessory olfactory bulb mitral layer-Somatomotor areas	0	0	0	0
Striatum -Somatomotor areas	-0.36502	-0.7598	0.874121	-0.06548
Midbrain -Somatomotor areas	-0.00172	-0.0063	-0.0113	-0.00402
Medulla-Somatomotor areas	-0.01765	-0.02026	-0.09838	-0.0143
piriform area-Somatosensory areas	0	0	0	0
Taenia tecta -Somatosensory areas	0.293585	0	-0.56536	0.861291
Accessory olfactory bulb glomerular layer-Somatosensory areas	0	0	0	0
Accessory olfactory bulb granular layer-Somatosensory areas	0	0	0	0
Retrohippocampal region -Somatosensory areas	-0.15113	-0.44582	-0.08933	-0.09889
Entorhinal area-Somatosensory areas	-0.64692	-0.35085	-0.06913	-0.13803
Field CA1-Somatosensory areas	-0.1024	-0.14379	-0.27346	-0.1944
Field CA3--Somatosensory areas	-0.20219	-0.17853	-0.09755	-0.35582
Dentate gyrus-Somatosensory areas	-0.37977	-0.09548	-0.42168	-0.27376
Field CA2 -Somatosensory areas	0	0	0	0
Accessory olfactory bulb mitral layer-Somatosensory areas	0	0	0	0
Striatum --Somatosensory areas	0.091655	-0.22603	-0.59402	-0.58713
Midbrain --Somatosensory areas	-0.00553	-0.00851	-0.0078	-0.00237
Medulla-Somatosensory areas	-0.00577	-0.01661	-0.01384	-0.02562
Taenia tecta -piriform area	0	0	0	0
Accessory olfactory bulb glomerular layer-piriform area	0	0	0	0
Accessory olfactory bulb granular layer-piriform area	0	0	0	0
Retrohippocampal region -piriform area	0	0	0	0
Entorhinal area-piriform area	0	0	-0.02984	0
Field CA1-piriform area	0	0	0	0
Field CA3-piriform area	0	0	0	-0.32025
Dentate gyrus-piriform area	0	-0.82145	0	-0.80868
Field CA2 -piriform area	0	0	0	0
Accessory olfactory bulb mitral layer-piriform area	0	0	0	0
Striatum -piriform area	0.293585	-0.4926	-0.56065	0.882734
Midbrain -piriform area	-0.02307	-0.99361	-0.03379	0.420645

Medulla-piriform area	0	0	-0.06282	0
Accessory olfactory bulb glomerular layer-Taenia tecta	0	0	0	0
Accessory olfactory bulb granular layer-Taenia tecta	0	0	0	0
Retrohippocampal region -Taenia tecta	-0.30235	-0.28716	-0.20572	-0.08646
Entorhinal area-Taenia tecta	0	0	-0.09027	0
Field CA1-Taenia tecta	-0.09106	-0.0691	0	-0.07674
Field CA3-Taenia tecta	-0.50453	-0.03324	-0.48041	-0.02255
Dentate gyrus-Taenia tecta	-0.20631	-0.16591	0.921696	-0.09336
Field CA2 -Taenia tecta	0	0	0	0
Accessory olfactory bulb mitral layer-Taenia tecta	0	0	0	0
Striatum -Taenia tecta	0.00207	0.045805	0.007948	0.005856
Midbrain -Taenia tecta	-0.0206	-0.00365	-0.06282	-0.00538
Medulla-Taenia tecta	-0.07094	0	-0.06911	-0.00828
Accessory olfactory bulb granular layer-Accessory olfactory bulb glomerular layer	0	0	0	0
Retrohippocampal region -Accessory olfactory bulb glomerular layer	0	0	0	0
Entorhinal area-Accessory olfactory bulb glomerular layer	0	0	0	0
Field CA1-Accessory olfactory bulb glomerular layer	0	0	0	0
Field CA3-Accessory olfactory bulb glomerular layer	0	0	0	0
Dentate gyrus-Accessory olfactory bulb glomerular layer	0	0	0	0
Field CA2 -Accessory olfactory bulb glomerular layer	0	0	0	0
Accessory olfactory bulb mitral layer-Accessory olfactory bulb glomerular layer	0	0	0	0
Striatum -Accessory olfactory bulb glomerular layer	0	0	0	0
Midbrain -Accessory olfactory bulb glomerular layer	0	0	0	0
Medulla-Accessory olfactory bulb glomerular layer	0	0	0	0
Retrohippocampal region -Accessory olfactory bulb granular layer	0	0	0	0
Entorhinal area-Accessory olfactory bulb granular layer	0	0	0	0
Field CA1-Accessory olfactory bulb granular layer	0	0	0	0
Field CA3-Accessory olfactory bulb granular layer	0	0	0	0
Dentate gyrus-Accessory olfactory bulb granular layer	0	0	0	0
Field CA2 -Accessory olfactory bulb granular layer	0	0	0	0
Accessory olfactory bulb mitral layer-Accessory olfactory bulb granular layer	0	0	0	0
Striatum -Accessory olfactory bulb granular layer	0	0	0	0

Midbrain -Accessory olfactory bulb granular layer	0	0	0	0
Medulla-Accessory olfactory bulb granular layer	0	0	0	0
Entorhinal area-Retrohippocampal region	-0.00971	0	-0.02684	-0.01399
Field CA1-Retrohippocampal region	-0.00534	-0.00263	-0.05813	-0.01302
Field CA3-Retrohippocampal region	-0.40718	-0.03226	-0.01707	-0.01265
Dentate gyrus-Retrohippocampal region	-0.87453	-0.04513	-0.09681	-0.01691
Field CA2 -Retrohippocampal region	0	0	0	0
Accessory olfactory bulb mitral layer-Retrohippocampal region	0	0	0	0
Striatum -Retrohippocampal region	-0.01141	-0.00378	-0.01047	-0.00275
Midbrain -Retrohippocampal region	-0.02391	-0.00378	-0.00795	-0.00201
Medulla-Retrohippocampal region	-0.0206	-0.13491	-0.09027	-0.08402
Field CA1-Entorhinal area	0	-0.01222	0	-0.02372
Field CA3-Entorhinal area	-0.42956	-0.00413	-0.12805	-0.0207
Dentate gyrus-Entorhinal area	-0.60816	-0.03625	-0.10125	-0.00945
Field CA2 -Entorhinal area	0	0	0	0
Accessory olfactory bulb mitral layer-Entorhinal area	0	0	0	0
Striatum -Entorhinal area	-0.01417	-0.00898	-0.03447	-0.00395
Midbrain -Entorhinal area	-0.00485	-0.00365	-0.00323	-0.0016
Medulla-Entorhinal area	-0.0462	-0.01276	-0.05291	-0.04741
Field CA3-Field CA1	-0.04528	-0.09361	-0.01205	-0.02357
Dentate gyrus-Field CA1	-0.30741	-0.07381	-0.03186	-0.03766
Field CA2 -Field CA1	0	0	0	0
Accessory olfactory bulb mitral layer-Field CA1	0	0	0	0
Striatum -Field CA1	-0.00245	-0.00538	-0.00336	-0.00671
Midbrain -Field CA1	-0.00585	-0.00499	-0.00353	-0.00201
Medulla-Field CA1	-0.02561	-0.05149	-0.13006	-0.0363
Dentate gyrus-Field CA3	0.876069	-0.04793	-0.11532	-0.07886
Field CA2 -Field CA3	0	-0.09361	-0.08594	-0.12121
Accessory olfactory bulb mitral layer-Field CA3	0	0	0	0
Striatum -Field CA3	-0.01627	-0.0133	-0.07258	-0.00806
Midbrain -Field CA3	-0.08312	-0.00804	-0.01823	-0.02324
Medulla-Field CA3	-0.10416	-0.03906	-0.1041	-0.01282

Field CA2 -Dentate gyrus	0	0	-0.14859	-0.14112
Accessory olfactory bulb mitral layer-Dentate gyrus	0	0	0	0
Striatum -Dentate gyrus	-0.03055	-0.03905	-0.06282	-0.09391
Midbrain -Dentate gyrus	-0.05531	-0.09517	-0.01823	-0.23193
Medulla-Dentate gyrus	-0.03404	-0.09361	-0.0692	-0.0554
Accessory olfactory bulb mitral layer-Field CA2	0	0	0	0
Striatum -Field CA2	-0.01718	-0.00598	-0.45709	-0.01349
Midbrain -Field CA2	-0.00301	-0.00832	-0.00596	-0.0062
Medulla-Field CA2	0	0	-0.06518	-0.09974
Striatum -Accessory olfactory bulb mitral layer	0	0	0	0
Midbrain -Accessory olfactory bulb mitral layer	0	0	0	0
Medulla-Accessory olfactory bulb mitral layer	0	0	0	0
Midbrain -Striatum	-0.00595	-0.00704	-0.01191	-0.01376
Medulla-Striatum	-0.00438	-0.00365	-0.0078	-0.00247
Medulla-Midbrain	-0.00865	-0.02581	-0.0232	-0.00538

S12. Adjust p values for group comparisons of pair-wised connectivity for Medulla.

Region Connections	Medulla			
	Left Ipsilateral	Left Contralateral	Right Contralateral	Right Ipsilateral
Caudoputamen-corpus callosum	-0.04262	0	0	0
anterior commissure olfactory limb-corpus callosum	0	0	0	0
pallidum -corpus callosum	0	0	0	0
internal capsule-corpus callosum	0	0	0	0
Thalamus-corpus callosum	0	0	0	0
Cerebellum-corpus callosum	-0.01209	-0.00735	-0.0241	-0.03279
Superior colliculus-corpus callosum	0	0	0	0
ventricular systems-corpus callosum	0	0	-0.06675	0
Hypothalamus -corpus callosum	0	0	0	0
Inferior colliculus -corpus callosum	0	0	0	0
periaqueductal gray-corpus callosum	0	0	0	0
Isocortex -corpus callosum	0	0	0	0
Cortical amygdalar area -corpus callosum	0	0	0	0
Olfactory areas -corpus callosum	0	0	0	0
Pons-corpus callosum	-0.00308	-0.01261	-0.03812	-0.19131
Midbrain reticular nucleus-corpus callosum	0	0	0	0
Nucleus accumbens-corpus callosum	0	0	0	0
fimbria-corpus callosum	0	0	0	0
Anterior cingulate area-corpus callosum	0	0	0	0
Somatomotor areas-corpus callosum	0	0	0	0
Somatosensory areas-corpus callosum	0	0	0	0
piriform area-corpus callosum	0	0	0	0
Taenia tecta -corpus callosum	0	0	0	0
Accessory olfactory bulb glomerular layer-corpus callosum	0	0	0	0
Accessory olfactory bulb granular layer-corpus callosum	0	0	0	0
Retrohippocampal region -corpus callosum	0	0	0	0
Entorhinal area-corpus callosum	0	0	0	0
Field CA1-corpus callosum	0	0	0	0
Field CA3-corpus callosum	0	0	0	0
Dentate gyrus-corpus callosum	0	0	0	0
Field CA2 -corpus callosum	0	0	0	0
Accessory olfactory bulb mitral layer-corpus callosum	0	0	0	0

Striatum -corpus callosum	0	0	0	0
Midbrain -corpus callosum	0	0	0	0
Medulla-corpus callosum	-0.00015	-0.00094	-0.00755	-0.00558
anterior commissure olfactory limb-Caudoputamen	0	0	0	0
pallidum -Caudoputamen	0	0	0	0
internal capsule-Caudoputamen	0	0	0	0
Thalamus-Caudoputamen	0	0	0	0
Cerebellum-Caudoputamen	-0.004009	-0.002639	-0.019353	-0.005578
Superior colliculus-Caudoputamen	0	0	0	0
ventricular systems-Caudoputamen	-0.007023	-0.004616	-0.030278	-0.012886
Hypothalamus -Caudoputamen	0	0	0	0
Inferior colliculus -Caudoputamen	0	0	-0.111499	0
periaqueductal gray-Caudoputamen	0	0	0	0
Isocortex -Caudoputamen	-0.212923	0	-0.111499	0
Cortical amygdalar area -Caudoputamen	0	0	0	0
Olfactory areas -Caudoputamen	0	0	0	0
Pons-Caudoputamen	-0.001346	-0.021165	-0.005681	-0.011169
Midbrain reticular nucleus-Caudoputamen	0	0	0	0
Nucleus accumbens-Caudoputamen	0	0	0	0
fimbria-Caudoputamen	0	0	0	0
Anterior cingulate area-Caudoputamen	0	0	0	0
Somatomotor areas-Caudoputamen	0	0	0	0
Somatosensory areas-Caudoputamen	0	0	0	0
piriform area-Caudoputamen	0	0	0	0
Taenia tecta -Caudoputamen	0	0	0	0
Accessory olfactory bulb glomerular layer-Caudoputamen	0	0	0	0
Accessory olfactory bulb granular layer-Caudoputamen	0	0	0	0
Retrohippocampal region -Caudoputamen	0	0	0	0
Entorhinal area-Caudoputamen	0	0	0	0
Field CA1-Caudoputamen	0	0	0	0
Field CA3-Caudoputamen	0	0	0	0
Dentate gyrus-Caudoputamen	0	0	0	0
Field CA2 -Caudoputamen	0	0	0	0
Accessory olfactory bulb mitral layer-Caudoputamen	0	0	0	0
Striatum -Caudoputamen	0	0	0	0
Midbrain -Caudoputamen	-0.120964	0	-0.027313	0

Medulla-Caudoputamen	-0.001253	-0.000935	-0.008218	-0.005578
pallidum -anterior commissure olfactory limb	0	0	0	0
internal capsule-anterior commissure olfactory limb	0	0	0	0
Thalamus-anterior commissure olfactory limb	0	0	0	0
Cerebellum-anterior commissure olfactory limb	0	0	0	0
Superior colliculus-anterior commissure olfactory limb	0	0	0	0
ventricular systems-anterior commissure olfactory limb	0	0	0	0
Hypothalamus -anterior commissure olfactory limb	0	0	0	0
Inferior colliculus -anterior commissure olfactory limb	0	0	0	0
periaqueductal gray-anterior commissure olfactory limb	0	0	0	0
Isocortex -anterior commissure olfactory limb	0	0	0	0
Cortical amygdalar area -anterior commissure olfactory limb	0	0	0	0
Olfactory areas -anterior commissure olfactory limb	0	0	0	0
Pons-anterior commissure olfactory limb	0	0	0	0
Midbrain reticular nucleus-anterior commissure olfactory limb	0	0	0	0
Nucleus accumbens-anterior commissure olfactory limb	0	0	0	0
fimbria-anterior commissure olfactory limb	0	0	0	0
Anterior cingulate area-anterior commissure olfactory limb	0	0	0	0
Somatomotor areas-anterior commissure olfactory limb	0	0	0	0
Somatosensory areas-anterior commissure olfactory limb	0	0	0	0
piriform area-anterior commissure olfactory limb	0	0	0	0
Taenia tecta -anterior commissure olfactory limb	0	0	0	0
Accessory olfactory bulb glomerular layer-anterior commissure olfactory limb	0	0	0	0
Accessory olfactory bulb granular layer-anterior commissure olfactory limb	0	0	0	0
Retrohippocampal region -anterior commissure olfactory limb	0	0	0	0
Entorhinal area-anterior commissure olfactory limb	0	0	0	0
Field CA1-anterior commissure olfactory limb	0	0	0	0
Field CA3-anterior commissure olfactory limb	0	0	0	0
Dentate gyrus-anterior commissure olfactory limb	0	0	0	0
Field CA2 -anterior commissure olfactory limb	0	0	0	0
Accessory olfactory bulb mitral layer-anterior commissure olfactory limb	0	0	0	0
Striatum -anterior commissure olfactory limb	0	0	0	0
Midbrain -anterior commissure olfactory limb	0	0	0	0
Medulla-anterior commissure olfactory limb	0	0	0	0

internal capsule-pallidum	0	0	0	0
Thalamus-pallidum	0	0	0	0
Cerebellum-pallidum	-0.049642	-0.072795	-0.104297	-0.015726
Superior colliculus-pallidum	0	0	0	0
ventricular systems-pallidum	0	0	-0.083037	-0.058426
Hypothalamus -pallidum	0	0	0	0
Inferior colliculus -pallidum	0	0	0	0
periaqueductal gray-pallidum	0	0	0	0
Isocortex -pallidum	0	0	0	0
Cortical amygdalar area -pallidum	0	0	0	0
Olfactory areas -pallidum	0	0	0	0
Pons-pallidum	-0.092711	-0.021165	-0.076029	-0.191305
Midbrain reticular nucleus-pallidum	0	0	0	0
Nucleus accumbens-pallidum	0	0	0	0
fimbria-pallidum	0	0	0	0
AVA-pallidum	0	0	0	0
Somatomotor areas-pallidum	0	0	0	0
Somatosensory areas-pallidum	0	0	0	0
piriform area-pallidum	0	0	0	0
Taenia tecta -pallidum	0	0	0	0
Accessory olfactory bulb glomerular layer-pallidum	0	0	0	0
Accessory olfactory bulb granular layer-pallidum	0	0	0	0
Retrohippocampal region -pallidum	0	0	0	0
Entorhinal area-pallidum	0	0	0	0
Field CA1-pallidum	0	0	0	0
Field CA3-pallidum	0	0	0	0
Dentate gyrus-pallidum	0	0	0	0
Field CA2 -pallidum	0	0	0	0
Accessory olfactory bulb mitral layer-pallidum	0	0	0	0
Striatum -pallidum	0	0	0	0
Midbrain -pallidum	0	0	0	0
Medulla-pallidum	-0.00308	-0.0032	-0.011307	-0.011169
Thalamus-internal capsule	0	0	0	0
Cerebellum-internal capsule	0	0	0	0
Superior colliculus-internal capsule	0	0	0	0

ventricular systems-internal capsule	0	0	0	0
Hypothalamus -internal capsule	0	0	0	0
Inferior colliculus -internal capsule	0	0	0	0
periaqueductal gray-internal capsule	0	0	0	0
Isocortex -internal capsule	0	0	0	0
Cortical amygdalar area -internal capsule	0	0	0	0
Olfactory areas -internal capsule	0	0	0	0
Pons-internal capsule	0	0	0	0
Midbrain reticular nucleus-internal capsule	0	0	0	0
Nucleus accumbens-internal capsule	0	0	0	0
fimbria-internal capsule	0	0	0	0
Anterior cingulate area-internal capsule	0	0	0	0
Somatomotor areas-internal capsule	0	0	0	0
Somatosensory areas-internal capsule	0	0	0	0
piriform area-internal capsule	0	0	0	0
Taenia tecta -internal capsule	0	0	0	0
Accessory olfactory bulb glomerular layer-internal capsule	0	0	0	0
Accessory olfactory bulb granular layer-internal capsule	0	0	0	0
Retrohippocampal region -internal capsule	0	0	0	0
Entorhinal area-internal capsule	0	0	0	0
Field CA1-internal capsule	0	0	0	0
Field CA3-internal capsule	0	0	0	0
Dentate gyrus-internal capsule	0	0	0	0
Field CA2 -internal capsule	0	0	0	0
Accessory olfactory bulb mitral layer-internal capsule	0	0	0	0
Striatum -internal capsule	0	0	0	0
Midbrain --internal capsule	0	0	0	0
Medulla-internal capsule	-0.00308	0	-0.226494	-0.182847
Cerebellum-Thalamus	-0.05826	-0.01314	-0.66073	-0.15018
Superior colliculus-Thalamus	0	0	0	0
ventricular systems-Thalamus	-0.5899	0	-0.48368	-0.29644
Hypothalamus -Thalamus	0	0	0	0
Inferior colliculus -Thalamus	0	0	0	0
periaqueductal gray-Thalamus	0	0	0	0
Isocortex -Thalamus	0	0	0	0

Cortical amygdalar area -Thalamus	0	0	0	0
Olfactory areas -Thalamus	0	0	0	0
Pons-Thalamus	-0.01463	0	-0.04131	-0.41112
Midbrain reticular nucleus-Thalamus	0	0	0	0
Nucleus accumbens-Thalamus	0	0	0	0
fimbria-Thalamus	0	0	0	0
Anterior cingulate area-Thalamus	0	0	0	0
Somatomotor areas-Thalamus	0	0	0	0
Somatosensory areas-Thalamus	0	0	0	0
piriform area-Thalamus	0	0	0	0
Taenia tecta -Thalamus	0	0	0	0
Accessory olfactory bulb glomerular layer-Thalamus	0	0	0	0
Accessory olfactory bulb granular layer-Thalamus	0	0	0	0
Retrohippocampal region -Thalamus	0	0	0	0
Entorhinal area-Thalamus	0	0	0	0
Field CA1-Thalamus	0	0	0	0
Field CA3-Thalamus	0	0	0	0
Dentate gyrus-Thalamus	0	0	0	0
Field CA2 -Thalamus	0	0	0	0
Accessory olfactory bulb mitral layer-Thalamus	0	0	0	0
Striatum -Thalamus	0	0	0	0
Midbrain -Thalamus	0	0	0	0
Medulla-Thalamus	-0.00398	-0.01261	-0.0241	-0.02727
Superior colliculus-Cerebellum	-0.64327	0.302408	-0.12488	0.844686
ventricular systems-Cerebellum	0.739952	0.072795	0.205105	0.300375
Hypothalamus -Cerebellum	-0.00525	-0.02341	-0.15476	-0.16042
Inferior colliculus -Cerebellum	-0.04481	-0.0728	-0.008	-0.12668
periaqueductal gray-Cerebellum	0.179314	0.086827	-0.07	-0.53912
Isocortex -Cerebellum	-0.17859	-0.06822	-0.00875	-0.01299
Cortical amygdalar area -Cerebellum	0.031759	0	0	0
Olfactory areas -Cerebellum	0	0	0	-0.03788
Pons-Cerebellum	0.450683	-0.62916	-0.07	0.44698
Midbrain reticular nucleus-Cerebellum	-0.63915	0	0	-0.0974
Nucleus accumbens-Cerebellum	0	0	0	0
fimbria-Cerebellum	0	0	0	0

Anterior cingulate area-Cerebellum	0	0	0	0
Somatomotor areas-Cerebellum	-0.22652	-0.13704	0	0
Somatosensory areas-Cerebellum	-0.07943	-0.10427	-0.13752	-0.20606
piriform area-Cerebellum	0	0	0	0
Taenia tecta -Cerebellum	0	0	0	0
Accessory olfactory bulb glomerular layer-Cerebellum	0	0	0	0
Accessory olfactory bulb granular layer-Cerebellum	0	0	0	0
Retrohippocampal region -Cerebellum	0.005691	0.629161	0	0.928513
Entorhinal area-Cerebellum	0.00836	0.268979	-0.53634	-0.38261
Field CA1-Cerebellum	0.358098	0	0	-0.92947
Field CA3-Cerebellum	0.026344	0.629161	-0.8552	-0.94207
Dentate gyrus-Cerebellum	0.003943	0.08871	-0.78113	0.65477
Field CA2 -Cerebellum	0	0	0	0
Accessory olfactory bulb mitral layer-Cerebellum	0	0	0	0
Striatum -Cerebellum	-0.00569	-0.04094	-0.003	-0.01117
Midbrain -Cerebellum	-0.04451	-0.08683	-0.3335	-0.45908
Medulla-Cerebellum	0.206811	-0.56303	0.64504	0.516015
ventricular systems-Superior colliculus	-0.54885	0	0.343547	-0.30038
Hypothalamus -Superior colliculus	0	0	0	0
Inferior colliculus -Superior colliculus	0	0	0	0
periaqueductal gray-Superior colliculus	0	0	0	0
Isocortex -Superior colliculus	0	0	0	0
Cortical amygdalar area -Superior colliculus	0	0	0	0
Olfactory areas -Superior colliculus	0	0	0	0
Pons-Superior colliculus	0.897172	-0.09293	-0.81798	-0.40585
Midbrain reticular nucleus-Superior colliculus	0	0	0	0
Nucleus accumbens-Superior colliculus	0	0	0	0
fimbria-Superior colliculus	0	0	0	0
Anterior cingulate area-Superior colliculus	0	0	0	0
Somatomotor areas-Superior colliculus	0	0	0	0
Somatosensory areas-Superior colliculus	0	0	0	0
piriform area-Superior colliculus	0	0	0	0
Taenia tecta -Superior colliculus	0	0	0	0
Accessory olfactory bulb glomerular layer-Superior colliculus	0	0	0	0
Accessory olfactory bulb granular layer-Superior colliculus	0	0	0	0

Retrohippocampal region -Superior colliculus	0	0	0	0
Entorhinal area-Superior colliculus	0	0	0	0
Field CA1-Superior colliculus	0	0	0	0
Field CA3-Superior colliculus	0	0	0	0
Dentate gyrus-Superior colliculus	0	0	0	0
Field CA2 -Superior colliculus	0	0	0	0
Accessory olfactory bulb mitral layer-Superior colliculus	0	0	0	0
Striatum -Superior colliculus	0	0	0	0
Midbrain -Superior colliculus	0	0	0	0
Medulla-Superior colliculus	-0.02634	-0.01261	-0.2752	-0.10418
Hypothalamus -ventricular systems	-0.04257	-0.022196	0	-0.028505
Inferior colliculus -ventricular systems	-0.044641	-0.268979	-0.066735	-0.405847
periaqueductal gray-ventricular systems	0.194202	0.021165	-0.957529	-0.333755
Isocortex -ventricular systems	-0.092711	-0.110723	-0.032988	-0.015726
Cortical amygdalar area -ventricular systems	0	0	0	0
Olfactory areas -ventricular systems	0	0	0	0
Pons-ventricular systems	0.406205	0.629161	0.667066	0.196559
Midbrain reticular nucleus-ventricular systems	0	0	0	0
Nucleus accumbens-ventricular systems	0	0	0	0
fimbria-ventricular systems	0	0	0	0
Anterior cingulate area-ventricular systems	0	0	0	0
Somatomotor areas-ventricular systems	0	0	0	0
Somatosensory areas-ventricular systems	0	-0.173247	0	0
piriform area-ventricular systems	0	0	0	0
Taenia tecta -ventricular systems	0	0	0	0
Accessory olfactory bulb glomerular layer-ventricular systems	0	0	0	0
Accessory olfactory bulb granular layer-ventricular systems	0	0	0	0
Retrohippocampal region -ventricular systems	0.020183	0	0	0
Entorhinal area-ventricular systems	0.007333	0.072795	0	-0.196559
Field CA1-ventricular systems	0	0	0	0
Field CA3-ventricular systems	0	0	0	0
Dentate gyrus-ventricular systems	0.000075	0.007346	0	-0.929465
Field CA2 -ventricular systems	0	0	0	0
Accessory olfactory bulb mitral layer-ventricular systems	0	0	0	0
Striatum -ventricular systems	-0.028795	-0.021165	-0.070003	-0.012886

Midbrain -ventricular systems	-0.088333	-0.258988	-0.957529	-0.617851
Medulla-ventricular systems	0.040288	-0.709721	0.031461	0.518408
Inferior colliculus -Hypothalamus	0	0	0	0
periaqueductal gray-Hypothalamus	0	0	0	0
Isocortex -Hypothalamus	0	0	0	0
Cortical amygdalar area -Hypothalamus	0	0	0	0
Olfactory areas -Hypothalamus	0	0	0	0
Pons-Hypothalamus	-0.013931	-0.866725	-0.007545	-0.045959
Midbrain reticular nucleus-Hypothalamus	0	0	0	0
Nucleus accumbens-Hypothalamus	0	0	0	0
fimbria-Hypothalamus	0	0	0	0
Anterior cingulate area-Hypothalamus	0	0	0	0
Somatomotor areas-Hypothalamus	0	0	0	0
Somatosensory areas-Hypothalamus	0	0	0	0
piriform area-Hypothalamus	0	0	0	0
Taenia tecta -Hypothalamus	0	0	0	0
Accessory olfactory bulb glomerular layer-Hypothalamus	0	0	0	0
Accessory olfactory bulb granular layer-Hypothalamus	0	0	0	0
Retrohippocampal region -Hypothalamus	0	0	0	0
Entorhinal area-Hypothalamus	0	0	0	0
Field CA1-Hypothalamus	0	0	0	0
Field CA3-Hypothalamus	0	0	0	0
Dentate gyrus-Hypothalamus	0	0	0	0
Field CA2 -Hypothalamus	0	0	0	0
Accessory olfactory bulb mitral layer-Hypothalamus	0	0	0	0
Striatum -Hypothalamus	0	0	0	0
Midbrain -Hypothalamus	0	0	0	0
Medulla-Hypothalamus	-0.006478	-0.085202	-0.008751	-0.016857
periaqueductal gray-Inferior colliculus	0	0	0	0
Isocortex -Inferior colliculus	0	0	0	0
Cortical amygdalar area -Inferior colliculus	0	0	0	0
Olfactory areas -Inferior colliculus	0	0	0	0
Pons-Inferior colliculus	-0.0504	-0.00735	-0.00568	-0.03216
Midbrain reticular nucleus-Inferior colliculus	0	0	0	0

Nucleus accumbens-Inferior colliculus	0	0	0	0
fimbria-Inferior colliculus	0	0	0	0
Anterior cingulate area-Inferior colliculus	0	0	0	0
Somatomotor areas-Inferior colliculus	0	0	0	0
Somatosensory areas-Inferior colliculus	0	0	0	0
piriform area-Inferior colliculus	0	0	0	0
Taenia tecta -Inferior colliculus	0	0	0	0
Accessory olfactory bulb glomerular layer-Inferior colliculus	0	0	0	0
Accessory olfactory bulb granular layer-Inferior colliculus	0	0	0	0
Retrohippocampal region -Inferior colliculus	0	0	0	0
Entorhinal area-Inferior colliculus	0	0	0	0
Field CA1-Inferior colliculus	0	0	0	0
Field CA3-Inferior colliculus	0	0	0	0
Dentate gyrus-Inferior colliculus	0	0	0	0
Field CA2 -Inferior colliculus	0	0	0	0
Accessory olfactory bulb mitral layer-Inferior colliculus	0	0	0	0
Striatum -Inferior colliculus	0	0	0	0
Midbrain -Inferior colliculus	0	-0.11579	0	0
Medulla-Inferior colliculus	-0.00308	-0.00204	-0.0241	-0.01299
Isocortex -periaqueductal gray	0	0	0	0
Cortical amygdalar area -periaqueductal gray	0	0	0	0
Olfactory areas -periaqueductal gray	0	0	0	0
Pons-periaqueductal gray	0.020183	0.709721	0.031233	-0.25272
Midbrain reticular nucleus-periaqueductal gray	0	0	0	0
Nucleus accumbens-periaqueductal gray	0	0	0	0
fimbria-periaqueductal gray	0	0	0	0
Anterior cingulate area-periaqueductal gray	0	0	0	0
Somatomotor areas-periaqueductal gray	0	0	0	0
Somatosensory areas-periaqueductal gray	0	0	0	0
piriform area-periaqueductal gray	0	0	0	0
Taenia tecta -periaqueductal gray	0	0	0	0
Accessory olfactory bulb glomerular layer-periaqueductal gray	0	0	0	0
Accessory olfactory bulb granular layer-periaqueductal gray	0	0	0	0
Retrohippocampal region -periaqueductal gray	0	0	0	0
Entorhinal area-periaqueductal gray	0	0	0	0

Field CA1-periaqueductal gray	0	0	0	0
Field CA3-periaqueductal gray	0	0	0	0
Dentate gyrus-periaqueductal gray	0	0	0	0
Field CA2 -periaqueductal gray	0	0	0	0
Accessory olfactory bulb mitral layer-periaqueductal gray	0	0	0	0
Striatum -periaqueductal gray	0	0	0	0
Midbrain -periaqueductal gray	0.04257	0	0.071198	0
Medulla-periaqueductal gray	-0.73639	-0.00735	0.256644	-0.05612
Cortical amygdalar area -Isocortex	0	0	0	0
Olfactory areas -Isocortex	0	0	0	0
Pons-Isocortex	-0.2825	-0.01314	-0.00822	-0.05204
Midbrain reticular nucleus-Isocortex	0	0	0	0
Nucleus accumbens-Isocortex	0	0	0	0
fimbria-Ictx	0	0	0	0
Anterior cingulate area-Isocortex	0	0	0	0
Somatomotor areas-Isocortex	0	0	0	0
Somatosensory areas-Isocortex	0	0	0	0
piriform area-Isocortex	0	0	0	0
Taenia tecta -Isocortex	0	0	0	0
Accessory olfactory bulb glomerular layer-Isocortex	0	0	0	0
Accessory olfactory bulb granular layer-Isocortex	0	0	0	0
Retrohippocampal region -Isocortex	0	0	0	0
Entorhinal area-Isocortex	0	0	0	0
Field CA1-Isocortex	0	0	0	0
Field CA3-Isocortex	0	0	0	0
Dentate gyrus-Isocortex	0	0	0	0
Field CA2 -Isocortex	0	0	0	0
Accessory olfactory bulb mitral layer-Isocortex	0	0	0	0
Striatum -Isocortex	0	0	0	0
Midbrain -Isocortex	-0.18714	-0.14153	-0.24972	0
Medulla-Isocortex	-0.02634	-0.00462	-0.02854	-0.01289
Olfactory areas -Cortical amygdalar area	0	0	0	0
Pons-Cortical amygdalar area	0	0	0	0
Midbrain reticular nucleus-Cortical amygdalar area	0	0	0	0

Nucleus accumbens-Cortical amygdalar area	0	0	0	0
fimbria-Cortical amygdalar area	0	0	0	0
Anterior cingulate area-Cortical amygdalar area	0	0	0	0
Somatomotor areas-Cortical amygdalar area	0	0	0	0
Somatosensory areas-Cortical amygdalar area	0	0	0	0
piriform area-Cortical amygdalar area	0	0	0	0
Taenia tecta -Cortical amygdalar area	0	0	0	0
Accessory olfactory bulb glomerular layer-Cortical amygdalar area	0	0	0	0
Accessory olfactory bulb granular layer-Cortical amygdalar area	0	0	0	0
Retrohippocampal region -Cortical amygdalar area	0	0	0	0
Entorhinal area-Cortical amygdalar area	0	0	0	0
Field CA1-Cortical amygdalar area	0	0	0	0
Field CA3-Cortical amygdalar area	0	0	0	0
Dentate gyrus-Cortical amygdalar area	0	0	0	0
Field CA2 -Cortical amygdalar area	0	0	0	0
Accessory olfactory bulb mitral layer-Cortical amygdalar area	0	0	0	0
Striatum -Cortical amygdalar area	0	0	0	0
Midbrain -Cortical amygdalar area	0	0	0	0
Medulla-Cortical amygdalar area	0.05158	0	0.102037	0.433972
Pons-Olfactory areas	0	0	0	0
Midbrain reticular nucleus-Olfactory areas	0	0	0	0
Nucleus accumbens-Olfactory areas	0	0	0	0
fimbria-Olfactory areas	0	0	0	0
Anterior cingulate area-Olfactory areas	0	0	0	0
Somatomotor areas-Olfactory areas	0	0	0	0
Somatosensory areas-Olfactory areas	0	0	0	0
piriform area-Olfactory areas	0	0	0	0
Taenia tecta -Olfactory areas	0	0	0	0
Accessory olfactory bulb glomerular layer-Olfactory areas	0	0	0	0
Accessory olfactory bulb granular layer-Olfactory areas	0	0	0	0
Retrohippocampal region -Olfactory areas	0	0	0	0
Entorhinal area-Olfactory areas	0	0	0	0
Field CA1-Olfactory areas	0	0	0	0
Field CA3-Olfactory areas	0	0	0	0
Dentate gyrus-Olfactory areas	0	0	0	0

Field CA2 -Olfactory areas	0	0	0	0
Accessory olfactory bulb mitral layer-Olfactory areas	0	0	0	0
Striatum -Olfactory areas	0	0	0	0
Midbrain -Olfactory areas	0	0	0	0
Medulla-Olfactory areas	-0.04257	-0.04094	-0.03299	-0.01289
Midbrain reticular nucleus-Pons	0.090501	0	0	0
Nucleus accumbens-Pons	0	0	0	0
fimbria-Pons	0	0	0	0
Anterior cingulate area-Pons	0	0	0	0
Somatomotor areas-Pons	0	0	0	0
Somatosensory areas-Pons	-0.09403	-0.09293	-0.09801	0
piriform area-Pons	0	0	0	0
Taenia tecta -Pons	0	0	0	0
Accessory olfactory bulb glomerular layer-Pons	0	0	0	0
Accessory olfactory bulb granular layer-Pons	0	0	0	0
Retrohippocampal region -Pons	0.026344	-0.43263	0	0
Entorhinal area-Pons	0.00836	0	-0.44563	-0.94207
Field CA1-Pons	0	0	0	0
Field CA3-Pons	0.000728	0.019053	0	0
Dentate gyrus-Pons	0.001346	0.173247	0	0.028906
Field CA2 -Pons	0	0	0	0
Accessory olfactory bulb mitral layer-Pons	0	0	0	0
Striatum -Pons	-0.00702	-0.0371	-0.00875	-0.00825
Midbrain -Pons	0.041613	0.110723	0.070592	0.045267
Medulla-Pons	-0.3809	-0.1352	-0.357	-0.92851
Nucleus accumbens-Midbrain reticular nucleus	0	0	0	0
fimbria-Midbrain reticular nucleus	0	0	0	0
Anterior cingulate area-Midbrain reticular nucleus	0	0	0	0
Somatomotor areas-Midbrain reticular nucleus	0	0	0	0
Somatosensory areas-Midbrain reticular nucleus	0	0	0	0
piriform area-Midbrain reticular nucleus	0	0	0	0
Taenia tecta -Midbrain reticular nucleus	0	0	0	0
Accessory olfactory bulb glomerular layer-Midbrain reticular nucleus	0	0	0	0
Accessory olfactory bulb granular layer-Midbrain reticular nucleus	0	0	0	0

Retrohippocampal region -Midbrain reticular nucleus	0	0	0	0
Entorhinal area-Midbrain reticular nucleus	0	0	0	0
Field CA1-Midbrain reticular nucleus	0	0	0	0
Field CA3-Midbrain reticular nucleus	0	0	0	0
Dentate gyrus-Midbrain reticular nucleus	0	0	0	0
Field CA2 -Midbrain reticular nucleus	0	0	0	0
Accessory olfactory bulb mitral layer-Midbrain reticular nucleus	0	0	0	0
Striatum -Midbrain reticular nucleus	0	0	0	0
Midbrain -Midbrain reticular nucleus	0	0	0	0
Medulla-Midbrain reticular nucleus	-0.88207	0	-0.81798	-0.01573
fimbria-Nucleus accumbens	0	0	0	0
Anterior cingulate area-Nucleus accumbens	0	0	0	0
Somatomotor areas-Nucleus accumbens	0	0	0	0
Somatosensory areas-Nucleus accumbens	0	0	0	0
piriform area-Nucleus accumbens	0	0	0	0
Taenia tecta -Nucleus accumbens	0	0	0	0
Accessory olfactory bulb glomerular layer-Nucleus accumbens	0	0	0	0
Accessory olfactory bulb granular layer-Nucleus accumbens	0	0	0	0
Retrohippocampal region -Nucleus accumbens	0	0	0	0
Entorhinal area-Nucleus accumbens	0	0	0	0
Field CA1-Nucleus accumbens	0	0	0	0
Field CA3-Nucleus accumbens	0	0	0	0
Dentate gyrus-Nucleus accumbens	0	0	0	0
Field CA2 -Nucleus accumbens	0	0	0	0
Accessory olfactory bulb mitral layer-Nucleus accumbens	0	0	0	0
Striatum -Nucleus accumbens	0	0	0	0
Midbrain -Nucleus accumbens	0	0	0	0
Medulla-Nucleus accumbens	0	0	-0.17623	0
Anterior cingulate area-fimbria	0	0	0	0
Somatomotor areas-fimbria	0	0	0	0
Somatosensory areas-fimbria	0	0	0	0
piriform area-fimbria	0	0	0	0
Taenia tecta -fimbria	0	0	0	0
Accessory olfactory bulb glomerular layer-fimbria	0	0	0	0

Accessory olfactory bulb granular layer-fimbria	0	0	0	0
Retrohippocampal region -fimbria	0	0	0	0
Entorhinal area-fimbria	0	0	0	0
Field CA1-fimbria	0	0	0	0
Field CA3-fimbria	0	0	0	0
Dentate gyrus-fimbria	0	0	0	0
Field CA2 -fimbria	0	0	0	0
Accessory olfactory bulb mitral layer-fimbria	0	0	0	0
Striatum -fimbria	0	0	0	0
Midbrain -fimbria	0	0	0	0
Medulla-fimbria	0	0	0	0
Somatomotor areas-Anterior cingulate area	0	0	0	0
Somatosensory areas-Anterior cingulate area	0	0	0	0
piriform area-Anterior cingulate area	0	0	0	0
Taenia tecta -Anterior cingulate area	0	0	0	0
Accessory olfactory bulb glomerular layer-Anterior cingulate area	0	0	0	0
Accessory olfactory bulb granular layer-Anterior cingulate area	0	0	0	0
Retrohippocampal region -Anterior cingulate area	0	0	0	0
Entorhinal area-Anterior cingulate area	0	0	0	0
Field CA1-Anterior cingulate area	0	0	0	0
Field CA3-Anterior cingulate area	0	0	0	0
Dentate gyrus-Anterior cingulate area	0	0	0	0
Field CA2 -Anterior cingulate area	0	0	0	0
Accessory olfactory bulb mitral layer-Anterior cingulate area	0	0	0	0
Striatum -Anterior cingulate area	0	0	0	0
Midbrain -Anterior cingulate area	0	0	0	0
Medulla-Anterior cingulate area	0	-0.02706	-0.04564	-0.01573
Somatosensory areas-Somatomotor areas	0	0	0	0
piriform area-Somatomotor areas	0	0	0	0
Taenia tecta -Somatomotor areas	0	0	0	0
Accessory olfactory bulb glomerular layer-Somatomotor areas	0	0	0	0
Accessory olfactory bulb granular layer-Somatomotor areas	0	0	0	0
Retrohippocampal region -Somatomotor areas	0	0	0	0
Entorhinal area-Somatomotor areas	0	0	0	0

Field CA1-Somatomotor areas	0	0	0	0
Field CA3-Somatomotor areas	0	0	0	0
Dentate gyrus-Somatomotor areas	0	0	0	0
Field CA2 -Somatomotor areas	0	0	0	0
Accessory olfactory bulb mitral layer-Somatomotor areas	0	0	0	0
Striatum -Somatomotor areas	0	0	0	0
Midbrain -Somatomotor areas	0	0	0	0
Medulla-Somatomotor areas	-0.00308	-0.00462	-0.08095	-0.00558
piriform area-Somatosensory areas	0	0	0	0
Taenia tecta -Somatosensory areas	0	0	0	0
Accessory olfactory bulb glomerular layer-Somatosensory areas	0	0	0	0
Accessory olfactory bulb granular layer-Somatosensory areas	0	0	0	0
Retrohippocampal region -Somatosensory areas	0	0	0	0
Entorhinal area-Somatosensory areas	0	0	0	0
Field CA1-Somatosensory areas	0	0	0	0
Field CA3--Somatosensory areas	0	0	0	0
Dentate gyrus-Somatosensory areas	0	0	0	0
Field CA2 -Somatosensory areas	0	0	0	0
Accessory olfactory bulb mitral layer-Somatosensory areas	0	0	0	0
Striatum --Somatosensory areas	0	0	0	0
Midbrain --Somatosensory areas	0	0	0	0
Medulla-Somatosensory areas	-0.00074	-0.0222	-0.01131	-0.02705
Taenia tecta -piriform area	0	0	0	0
Accessory olfactory bulb glomerular layer-piriform area	0	0	0	0
Accessory olfactory bulb granular layer-piriform area	0	0	0	0
Retrohippocampal region -piriform area	0	0	0	0
Entorhinal area-piriform area	0	0	0	0
Field CA1-piriform area	0	0	0	0
Field CA3-piriform area	0	0	0	0
Dentate gyrus-piriform area	0	0	0	0
Field CA2 -piriform area	0	0	0	0
Accessory olfactory bulb mitral layer-piriform area	0	0	0	0
Striatum -piriform area	0	0	0	0
Midbrain -piriform area	0	0	0	0

Medulla-piriform area	-0.0504	0	-0.09801	0
Accessory olfactory bulb glomerular layer-Taenia tecta	0	0	0	0
Accessory olfactory bulb granular layer-Taenia tecta	0	0	0	0
Retrohippocampal region -Taenia tecta	0	0	0	0
Entorhinal area-Taenia tecta	0	0	0	0
Field CA1-Taenia tecta	0	0	0	0
Field CA3-Taenia tecta	0	0	0	0
Dentate gyrus-Taenia tecta	0	0	0	0
Field CA2 -Taenia tecta	0	0	0	0
Accessory olfactory bulb mitral layer-Taenia tecta	0	0	0	0
Striatum -Taenia tecta	0	0	0	0
Midbrain -Taenia tecta	0	0	0	0
Medulla-Taenia tecta	0	0	-0.10664	-0.05065
Accessory olfactory bulb granular layer-Accessory olfactory bulb glomerular layer	0	0	0	0
Retrohippocampal region -Accessory olfactory bulb glomerular layer	0	0	0	0
Entorhinal area-Accessory olfactory bulb glomerular layer	0	0	0	0
Field CA1-Accessory olfactory bulb glomerular layer	0	0	0	0
Field CA3-Accessory olfactory bulb glomerular layer	0	0	0	0
Dentate gyrus-Accessory olfactory bulb glomerular layer	0	0	0	0
Field CA2 -Accessory olfactory bulb glomerular layer	0	0	0	0
Accessory olfactory bulb mitral layer-Accessory olfactory bulb glomerular layer	0	0	0	0
Striatum -Accessory olfactory bulb glomerular layer	0	0	0	0
Midbrain -Accessory olfactory bulb glomerular layer	0	0	0	0
Medulla-Accessory olfactory bulb glomerular layer	0	0	0	0
Retrohippocampal region -Accessory olfactory bulb granular layer	0	0	0	0
Entorhinal area-Accessory olfactory bulb granular layer	0	0	0	0
Field CA1-Accessory olfactory bulb granular layer	0	0	0	0
Field CA3-Accessory olfactory bulb granular layer	0	0	0	0
Dentate gyrus-Accessory olfactory bulb granular layer	0	0	0	0
Field CA2 -Accessory olfactory bulb granular layer	0	0	0	0
Accessory olfactory bulb mitral layer-Accessory olfactory bulb granular layer	0	0	0	0
Striatum -Accessory olfactory bulb granular layer	0	0	0	0

Midbrain -Accessory olfactory bulb granular layer	0	0	0	0
Medulla-Accessory olfactory bulb granular layer	0	0	0	0
Entorhinal area-Retrohippocampal region	0	0	0	0
Field CA1-Retrohippocampal region	0	0	0	0
Field CA3-Retrohippocampal region	0	0	0	0
Dentate gyrus-Retrohippocampal region	0	0	0	0
Field CA2 -Retrohippocampal region	0	0	0	0
Accessory olfactory bulb mitral layer-Retrohippocampal region	0	0	0	0
Striatum -Retrohippocampal region	0	0	0	0
Midbrain -Retrohippocampal region	0	0	0	0
Medulla-Retrohippocampal region	0.282502	-0.11542	-0.12632	-0.44747
Field CA1-Entorhinal area	0	0	0	0
Field CA3-Entorhinal area	0	0	0	0
Dentate gyrus-Entorhinal area	0	0	0	0
Field CA2 -Entorhinal area	0	0	0	0
Accessory olfactory bulb mitral layer-Entorhinal area	0	0	0	0
Striatum -Entorhinal area	0	0	0	0
Midbrain -Entorhinal area	0	0	0	0
Medulla-Entorhinal area	0.106439	-0.08866	-0.66707	-0.41259
Field CA3-Field CA1	0	0	0	0
Dentate gyrus-Field CA1	0	0	0	0
Field CA2 -Field CA1	0	0	0	0
Accessory olfactory bulb mitral layer-Field CA1	0	0	0	0
Striatum -Field CA1	0	0	0	0
Midbrain -Field CA1	0	0	0	0
Medulla-Field CA1	-0.93567	0	-0.21539	-0.0974
Dentate gyrus-Field CA3	0	0	0	0
Field CA2 -Field CA3	0	0	0	0
Accessory olfactory bulb mitral layer-Field CA3	0	0	0	0
Striatum -Field CA3	0	0	0	0
Midbrain -Field CA3	0	0	0	0
Medulla-Field CA3	0.00836	-0.04464	0.635589	-0.33376

Field CA2 -Dentate gyrus	0	0	0	0
Accessory olfactory bulb mitral layer-Dentate gyrus	0	0	0	0
Striatum -Dentate gyrus	0	0	0	0
Midbrain -Dentate gyrus	0	0	0	0
Medulla-Dentate gyrus	0.007805	-0.12145	0.499699	0.396294
Accessory olfactory bulb mitral layer-Field CA2	0	0	0	0
Striatum -Field CA2	0	0	0	0
Midbrain -Field CA2	0	0	0	0
Medulla-Field CA2	0	0	0	-0.08534
Striatum -Accessory olfactory bulb mitral layer	0	0	0	0
Midbrain -Accessory olfactory bulb mitral layer	0	0	0	0
Medulla-Accessory olfactory bulb mitral layer	0	0	0	0
Midbrain -Striatum	0	0	0	0
Medulla-Striatum	-0.00308	-0.00204	-0.00791	-0.01289
Medulla-Midbrain	-0.04262	-0.06157	-0.17623	-0.41995

S13. Adjust p values for group comparisons of pair-wised connectivity for Motor Cortex.

Region Connections	Motor Cortex			
	Left Ipsilateral	Left Contralateral	Right Contralateral	Right Ipsilateral
Caudoputamen-corpus callosum	-0.00164	-0.02569	-0.22118	-0.06715
anterior commissure olfactory limb-corpus callosum	0	0	0	0
pallidum -corpus callosum	-0.00726	0	-0.38651	-0.05441
internal capsule-corpus callosum	0	0	0	0
Thalamus-corpus callosum	0	0	0	-0.01097
Cerebellum-corpus callosum	0	0	0	0
Superior colliculus-corpus callosum	0	-0.24148	0	-0.05068
ventricular systems-corpus callosum	-0.06111	-0.07945	-0.05261	-0.03992
Hypothalamus -corpus callosum	0	0	0	0
Inferior colliculus -corpus callosum	0	0	0	0
periaqueductal gray-corpus callosum	0	0	0	0
Isocortex -corpus callosum	-0.05079	-0.50132	-0.09599	-0.10075
Cortical amygdalar area -corpus callosum	0	0	0	0
Olfactory areas -corpus callosum	-0.00027	-0.66547	0	-0.00349
Pons-corpus callosum	0	0	0	0
Midbrain reticular nucleus-corpus callosum	0	0	0	0
Nucleus accumbens-corpus callosum	0	0	0	0
fimbria-corpus callosum	0	0	0	0
Anterior cingulate area-corpus callosum	-0.00063	-0.12998	-0.0001	-0.00071
Somatomotor areas-corpus callosum	0.474746	0.485603	-0.22523	-0.87372
Somatosensory areas-corpus callosum	-0.07071	0.419247	-0.54909	-0.16652
piriform area-corpus callosum	0.935934	0	0	0.835105
Taenia tecta -corpus callosum	0	0	0	0
Accessory olfactory bulb glomerular layer-corpus callosum	0	0	0	0
Accessory olfactory bulb granular layer-corpus callosum	0	0	0	0
Retrohippocampal region -corpus callosum	0	0	0	0
Entorhinal area-corpus callosum	0	0	0	0
Field CA1-corpus callosum	0	0	0	0
Field CA3-corpus callosum	0	0	0	0
Dentate gyrus-corpus callosum	0	0	0	0
Field CA2 -corpus callosum	0	0	0	0
Accessory olfactory bulb mitral layer-corpus callosum	0	0	0	0

Striatum -corpus callosum	-0.00065	-0.24148	-0.75777	-0.07146
Midbrain -corpus callosum	0	0	0	-0.02004
Medulla-corpus callosum	0	0	0	0
anterior commissure olfactory limb-Caudoputamen	0	0	0	0
pallidum -Caudoputamen	0	0	-0.222409	0
internal capsule-Caudoputamen	0	0	0	0
Thalamus-Caudoputamen	0	0	0	0
Cerebellum-Caudoputamen	0	0	0	0
Superior colliculus-Caudoputamen	0	0	-0.02975	0
ventricular systems-Caudoputamen	0	-0.085473	-0.02975	-0.227438
Hypothalamus -Caudoputamen	0	0	0	0
Inferior colliculus -Caudoputamen	0	0	0	0
periaqueductal gray-Caudoputamen	0	0	0	0
Isocortex -Caudoputamen	-0.002991	-0.360571	-0.02975	-0.438029
Cortical amygdalar area -Caudoputamen	0	0	0	0
Olfactory areas -Caudoputamen	0	-0.485603	0	-0.105157
Pons-Caudoputamen	0	0	0	0
Midbrain reticular nucleus-Caudoputamen	0	0	0	0
Nucleus accumbens-Caudoputamen	0	0	0	0
fimbria-Caudoputamen	0	0	0	0
Anterior cingulate area-Caudoputamen	-0.002991	-0.031094	-0.000052	-0.01254
Somatomotor areas-Caudoputamen	-0.812195	-0.478632	-0.218162	0.837738
Somatosensory areas-Caudoputamen	-0.761126	-0.501322	-0.156992	-0.166521
piriform area-Caudoputamen	0	0	0	0
Taenia tecta -Caudoputamen	0	0	0	0
Accessory olfactory bulb glomerular layer-Caudoputamen	0	0	0	0
Accessory olfactory bulb granular layer-Caudoputamen	0	0	0	0
Retrohippocampal region -Caudoputamen	0	0	0	0
Entorhinal area-Caudoputamen	0	0	0	0
Field CA1-Caudoputamen	0	0	0	0
Field CA3-Caudoputamen	0	0	0	0
Dentate gyrus-Caudoputamen	0	0	0	0
Field CA2 -Caudoputamen	0	0	0	0
Accessory olfactory bulb mitral layer-Caudoputamen	0	0	0	0
Striatum -Caudoputamen	-0.298792	-0.199308	-0.626961	-0.208756
Midbrain -Caudoputamen	0	-0.26511	0	0

Medulla-Caudoputamen	0	0	0	0
pallidum -anterior commissure olfactory limb	0	0	0	0
internal capsule-anterior commissure olfactory limb	0	0	0	0
Thalamus-anterior commissure olfactory limb	0	0	0	0
Cerebellum-anterior commissure olfactory limb	0	0	0	0
Superior colliculus-anterior commissure olfactory limb	0	0	0	0
ventricular systems-anterior commissure olfactory limb	0	0	0	0
Hypothalamus -anterior commissure olfactory limb	0	0	0	0
Inferior colliculus -anterior commissure olfactory limb	0	0	0	0
periaqueductal gray-anterior commissure olfactory limb	0	0	0	0
Isocortex -anterior commissure olfactory limb	0	0	0	0
Cortical amygdalar area -anterior commissure olfactory limb	0	0	0	0
Olfactory areas -anterior commissure olfactory limb	0	0	0	0
Pons-anterior commissure olfactory limb	0	0	0	0
Midbrain reticular nucleus-anterior commissure olfactory limb	0	0	0	0
Nucleus accumbens-anterior commissure olfactory limb	0	0	0	0
fimbria-anterior commissure olfactory limb	0	0	0	0
Anterior cingulate area-anterior commissure olfactory limb	0	0	0	0
Somatomotor areas-anterior commissure olfactory limb	0	0	0	0
Somatosensory areas-anterior commissure olfactory limb	0	0	0	0
piriform area-anterior commissure olfactory limb	0	0	0	0
Taenia tecta -anterior commissure olfactory limb	0	0	0	0
Accessory olfactory bulb glomerular layer-anterior commissure olfactory limb	0	0	0	0
Accessory olfactory bulb granular layer-anterior commissure olfactory limb	0	0	0	0
Retrohippocampal region -anterior commissure olfactory limb	0	0	0	0
Entorhinal area-anterior commissure olfactory limb	0	0	0	0
Field CA1-anterior commissure olfactory limb	0	0	0	0
Field CA3-anterior commissure olfactory limb	0	0	0	0
Dentate gyrus-anterior commissure olfactory limb	0	0	0	0
Field CA2 -anterior commissure olfactory limb	0	0	0	0
Accessory olfactory bulb mitral layer-anterior commissure olfactory limb	0	0	0	0
Striatum -anterior commissure olfactory limb	0	0	0	0
Midbrain -anterior commissure olfactory limb	0	0	0	0
Medulla-anterior commissure olfactory limb	0	0	0	0

internal capsule-pallidum	0	0	0	0
Thalamus-pallidum	0	0	0	0
Cerebellum-pallidum	0	0	0	0
Superior colliculus-pallidum	0	0	0	0
ventricular systems-pallidum	0	0	0	0
Hypothalamus -pallidum	0	0	0	0
Inferior colliculus -pallidum	0	0	0	0
periaqueductal gray-pallidum	0	0	0	0
Isocortex -pallidum	-0.082159	-0.751147	-0.167279	-0.072221
Cortical amygdalar area -pallidum	0	0	0	0
Olfactory areas -pallidum	0	0	0	0
Pons-pallidum	0	0	0	0
Midbrain reticular nucleus-pallidum	0	0	0	0
Nucleus accumbens-pallidum	0	0	0	0
fimbria-pallidum	0	0	0	0
AVA-pallidum	0	0	0	0
Somatomotor areas-pallidum	-0.120348	-0.751147	-0.422483	-0.313411
Somatosensory areas-pallidum	0.423335	0.665474	-0.558614	-0.714355
piriform area-pallidum	0	0	0	0
Taenia tecta -pallidum	0	0	0	0
Accessory olfactory bulb glomerular layer-pallidum	0	0	0	0
Accessory olfactory bulb granular layer-pallidum	0	0	0	0
Retrohippocampal region -pallidum	0	0	0	0
Entorhinal area-pallidum	0	0	0	0
Field CA1-pallidum	0	0	0	0
Field CA3-pallidum	0	0	0	0
Dentate gyrus-pallidum	0	0	0	0
Field CA2 -pallidum	0	0	0	0
Accessory olfactory bulb mitral layer-pallidum	0	0	0	0
Striatum -pallidum	0	0	0	0
Midbrain -pallidum	0	0	0	0
Medulla-pallidum	0	0	0	0
Thalamus-internal capsule	0	0	0	0
Cerebellum-internal capsule	0	0	0	0
Superior colliculus-internal capsule	0	0	0	0

ventricular systems-internal capsule	0	0	0	0
Hypothalamus -internal capsule	0	0	0	0
Inferior colliculus -internal capsule	0	0	0	0
periaqueductal gray-internal capsule	0	0	0	0
Isocortex -internal capsule	0	0	0	0
Cortical amygdalar area -internal capsule	0	0	0	0
Olfactory areas -internal capsule	0	0	0	0
Pons-internal capsule	0	0	0	0
Midbrain reticular nucleus-internal capsule	0	0	0	0
Nucleus accumbens-internal capsule	0	0	0	0
fimbria-internal capsule	0	0	0	0
Anterior cingulate area-internal capsule	0	0	0	0
Somatomotor areas-internal capsule	0	0	0	-0.497295
Somatosensory areas-internal capsule	0	0.458374	0	0
piriform area-internal capsule	0	0	0	0
Taenia tecta -internal capsule	0	0	0	0
Accessory olfactory bulb glomerular layer-internal capsule	0	0	0	0
Accessory olfactory bulb granular layer-internal capsule	0	0	0	0
Retrohippocampal region -internal capsule	0	0	0	0
Entorhinal area-internal capsule	0	0	0	0
Field CA1-internal capsule	0	0	0	0
Field CA3-internal capsule	0	0	0	0
Dentate gyrus-internal capsule	0	0	0	0
Field CA2 -internal capsule	0	0	0	0
Accessory olfactory bulb mitral layer-internal capsule	0	0	0	0
Striatum -internal capsule	0	0	0	0
Midbrain --internal capsule	0	0	0	0
Medulla-internal capsule	0	0	0	0
Cerebellum-Thalamus	0	0	0	0
Superior colliculus-Thalamus	0	0	0	0
ventricular systems-Thalamus	0	0	0	0
Hypothalamus -Thalamus	0	0	0	0
Inferior colliculus -Thalamus	0	0	0	0
periaqueductal gray-Thalamus	0	0	0	0
Isocortex -Thalamus	-0.01098	-0.22492	-0.15229	-0.05441

Cortical amygdalar area -Thalamus	0	0	0	0
Olfactory areas -Thalamus	0	0	0	0
Pons-Thalamus	0	0	0	0
Midbrain reticular nucleus-Thalamus	0	0	0	0
Nucleus accumbens-Thalamus	0	0	0	0
fimbria-Thalamus	0	0	0	0
Anterior cingulate area-Thalamus	0	0	0	0
Somatomotor areas-Thalamus	-0.06268	-0.28622	-0.30797	-0.20696
Somatosensory areas-Thalamus	-0.9041	-0.80657	-0.85972	-0.20839
piriform area-Thalamus	0	0	0	0
Taenia tecta -Thalamus	0	0	0	0
Accessory olfactory bulb glomerular layer-Thalamus	0	0	0	0
Accessory olfactory bulb granular layer-Thalamus	0	0	0	0
Retrohippocampal region -Thalamus	0	0	0	0
Entorhinal area-Thalamus	0	0	0	0
Field CA1-Thalamus	0	0	0	0
Field CA3-Thalamus	0	0	0	0
Dentate gyrus-Thalamus	0	0	0	0
Field CA2 -Thalamus	0	0	0	0
Accessory olfactory bulb mitral layer-Thalamus	0	0	0	0
Striatum -Thalamus	0	0	0	0
Midbrain -Thalamus	0	0	0	0
Medulla-Thalamus	0	0	0	0
Superior colliculus-Cerebellum	0	0	0	0
ventricular systems-Cerebellum	0	0	0	0
Hypothalamus -Cerebellum	0	0	0	0
Inferior colliculus -Cerebellum	0	0	0	0
periaqueductal gray-Cerebellum	0	0	0	0
Isocortex -Cerebellum	-0.00135	-0.04426	-0.05261	-0.03626
Cortical amygdalar area -Cerebellum	0	0	0	0
Olfactory areas -Cerebellum	0	0	0	0
Pons-Cerebellum	0	0	0	0
Midbrain reticular nucleus-Cerebellum	0	0	0	0
Nucleus accumbens-Cerebellum	0	0	0	0
fimbria-Cerebellum	0	0	0	0

Anterior cingulate area-Cerebellum	0	0	0	0
Somatomotor areas-Cerebellum	-0.00118	-0.04426	-0.00367	-0.01254
Somatosensory areas-Cerebellum	-0.29879	-0.12096	-0.22241	-0.02598
piriform area-Cerebellum	0	0	0	0
Taenia tecta -Cerebellum	0	0	0	0
Accessory olfactory bulb glomerular layer-Cerebellum	0	0	0	0
Accessory olfactory bulb granular layer-Cerebellum	0	0	0	0
Retrohippocampal region -Cerebellum	0	0	0	0
Entorhinal area-Cerebellum	0	0	0	0
Field CA1-Cerebellum	0	0	0	0
Field CA3-Cerebellum	0	0	0	0
Dentate gyrus-Cerebellum	0	0	0	0
Field CA2 -Cerebellum	0	0	0	0
Accessory olfactory bulb mitral layer-Cerebellum	0	0	0	0
Striatum -Cerebellum	0	0	0	0
Midbrain -Cerebellum	0	0	0	0
Medulla-Cerebellum	0	0	0	0
ventricular systems-Superior colliculus	0	0	0	0
Hypothalamus -Superior colliculus	0	0	0	0
Inferior colliculus -Superior colliculus	0	0	0	0
periaqueductal gray-Superior colliculus	0	0	0	0
Isocortex -Superior colliculus	-0.01069	-0.12096	-0.22241	-0.05068
Cortical amygdalar area -Superior colliculus	0	0	0	0
Olfactory areas -Superior colliculus	0	0	0	0
Pons-Superior colliculus	0	0	0	0
Midbrain reticular nucleus-Superior colliculus	0	0	0	0
Nucleus accumbens-Superior colliculus	0	0	0	0
fimbria-Superior colliculus	0	0	0	0
Anterior cingulate area-Superior colliculus	0	0	0	0
Somatomotor areas-Superior colliculus	-0.07251	-0.36057	-0.39152	-0.02671
Somatosensory areas-Superior colliculus	-0.39627	-0.31264	-0.22241	-0.22744
piriform area-Superior colliculus	0	0	0	0
Taenia tecta -Superior colliculus	0	0	0	0
Accessory olfactory bulb glomerular layer-Superior colliculus	0	0	0	0
Accessory olfactory bulb granular layer-Superior colliculus	0	0	0	0

Retrohippocampal region -Superior colliculus	0	0	0	0
Entorhinal area-Superior colliculus	0	0	0	0
Field CA1-Superior colliculus	0	0	0	0
Field CA3-Superior colliculus	0	0	0	0
Dentate gyrus-Superior colliculus	0	0	0	0
Field CA2 -Superior colliculus	0	0	0	0
Accessory olfactory bulb mitral layer-Superior colliculus	0	0	0	0
Striatum -Superior colliculus	0	0	0	0
Midbrain -Superior colliculus	0	0	0	0
Medulla-Superior colliculus	0	0	0	0
Hypothalamus -ventricular systems	0	0	0	0
Inferior colliculus -ventricular systems	0	0	0	0
periaqueductal gray-ventricular systems	0	0	0	0
Isocortex -ventricular systems	-0.01069	-0.229981	-0.062423	-0.325094
Cortical amygdalar area -ventricular systems	0	0	0	0
Olfactory areas -ventricular systems	0	0	0	0
Pons-ventricular systems	0	0	0	0
Midbrain reticular nucleus-ventricular systems	0	0	0	0
Nucleus accumbens-ventricular systems	0	0	0	0
fimbria-ventricular systems	0	0	0	0
Anterior cingulate area-ventricular systems	-0.000909	-0.001778	0	-0.004137
Somatomotor areas-ventricular systems	-0.372613	-0.286217	-0.15372	-0.917299
Somatosensory areas-ventricular systems	-0.356105	-0.501322	-0.152294	-0.110458
piriform area-ventricular systems	0	0	0	0
Taenia tecta -ventricular systems	0	0	0	0
Accessory olfactory bulb glomerular layer-ventricular systems	0	0	0	0
Accessory olfactory bulb granular layer-ventricular systems	0	0	0	0
Retrohippocampal region -ventricular systems	0	0	0	0
Entorhinal area-ventricular systems	0	0	0	0
Field CA1-ventricular systems	0	0	0	0
Field CA3-ventricular systems	0	0	0	0
Dentate gyrus-ventricular systems	0	0	0	0
Field CA2 -ventricular systems	0	0	0	0
Accessory olfactory bulb mitral layer-ventricular systems	0	0	0	0
Striatum -ventricular systems	0	0	0	0

Midbrain -ventricular systems	0	0	0	0
Medulla-ventricular systems	0	0	0	0
Inferior colliculus -Hypothalamus	0	0	0	0
periaqueductal gray-Hypothalamus	0	0	0	0
Isocortex -Hypothalamus	-0.003243	-0.24148	-0.09599	-0.072221
Cortical amygdalar area -Hypothalamus	0	0	0	0
Olfactory areas -Hypothalamus	0	0	0	0
Pons-Hypothalamus	0	0	0	0
Midbrain reticular nucleus-Hypothalamus	0	0	0	0
Nucleus accumbens-Hypothalamus	0	0	0	0
fimbria-Hypothalamus	0	0	0	0
Anterior cingulate area-Hypothalamus	0	0	0	0
Somatomotor areas-Hypothalamus	-0.120348	-0.286217	-0.073835	-0.152564
Somatosensory areas-Hypothalamus	-0.537968	-0.751147	-0.152294	-0.835105
piriform area-Hypothalamus	0	0	0	0
Taenia tecta -Hypothalamus	0	0	0	0
Accessory olfactory bulb glomerular layer-Hypothalamus	0	0	0	0
Accessory olfactory bulb granular layer-Hypothalamus	0	0	0	0
Retrohippocampal region -Hypothalamus	0	0	0	0
Entorhinal area-Hypothalamus	0	0	0	0
Field CA1-Hypothalamus	0	0	0	0
Field CA3-Hypothalamus	0	0	0	0
Dentate gyrus-Hypothalamus	0	0	0	0
Field CA2 -Hypothalamus	0	0	0	0
Accessory olfactory bulb mitral layer-Hypothalamus	0	0	0	0
Striatum -Hypothalamus	0	0	0	0
Midbrain -Hypothalamus	0	0	0	0
Medulla-Hypothalamus	0	0	0	0
periaqueductal gray-Inferior colliculus	0	0	0	0
Isocortex -Inferior colliculus	-0.00973	0	0	-0.05689
Cortical amygdalar area -Inferior colliculus	0	0	0	0
Olfactory areas -Inferior colliculus	0	0	0	0
Pons-Inferior colliculus	0	0	0	0
Midbrain reticular nucleus-Inferior colliculus	0	0	0	0

Nucleus accumbens-Inferior colliculus	0	0	0	0
fimbria-Inferior colliculus	0	0	0	0
Anterior cingulate area-Inferior colliculus	0	0	0	0
Somatomotor areas-Inferior colliculus	-0.00036	0	0	-0.00113
Somatosensory areas-Inferior colliculus	0	0	0	0
piriform area-Inferior colliculus	0	0	0	0
Taenia tecta -Inferior colliculus	0	0	0	0
Accessory olfactory bulb glomerular layer-Inferior colliculus	0	0	0	0
Accessory olfactory bulb granular layer-Inferior colliculus	0	0	0	0
Retrohippocampal region -Inferior colliculus	0	0	0	0
Entorhinal area-Inferior colliculus	0	0	0	0
Field CA1-Inferior colliculus	0	0	0	0
Field CA3-Inferior colliculus	0	0	0	0
Dentate gyrus-Inferior colliculus	0	0	0	0
Field CA2 -Inferior colliculus	0	0	0	0
Accessory olfactory bulb mitral layer-Inferior colliculus	0	0	0	0
Striatum -Inferior colliculus	0	0	0	0
Midbrain -Inferior colliculus	0	0	0	0
Medulla-Inferior colliculus	0	0	0	0
Isocortex -periaqueductal gray	-0.000011	-0.03109	-0.00097	-0.06822
Cortical amygdalar area -periaqueductal gray	0	0	0	0
Olfactory areas -periaqueductal gray	0	0	0	0
Pons-periaqueductal gray	0	0	0	0
Midbrain reticular nucleus-periaqueductal gray	0	0	0	0
Nucleus accumbens-periaqueductal gray	0	0	0	0
fimbria-periaqueductal gray	0	0	0	0
Anterior cingulate area-periaqueductal gray	0	0	0	0
Somatomotor areas-periaqueductal gray	-0.00028	-0.41112	-0.02954	-0.00143
Somatosensory areas-periaqueductal gray	-0.00708	-0.15839	-0.15229	-0.08607
piriform area-periaqueductal gray	0	0	0	0
Taenia tecta -periaqueductal gray	0	0	0	0
Accessory olfactory bulb glomerular layer-periaqueductal gray	0	0	0	0
Accessory olfactory bulb granular layer-periaqueductal gray	0	0	0	0
Retrohippocampal region -periaqueductal gray	0	0	0	0
Entorhinal area-periaqueductal gray	0	0	0	0

Field CA1-periaqueductal gray	0	0	0	0
Field CA3-periaqueductal gray	0	0	0	0
Dentate gyrus-periaqueductal gray	0	0	0	0
Field CA2 -periaqueductal gray	0	0	0	0
Accessory olfactory bulb mitral layer-periaqueductal gray	0	0	0	0
Striatum -periaqueductal gray	0	0	0	0
Midbrain -periaqueductal gray	0	0	0	0
Medulla-periaqueductal gray	0	0	0	0
Cortical amygdalar area -Isocortex	0	0	0.745712	-0.26701
Olfactory areas -Isocortex	-0.83295	-0.35068	-0.75777	-0.07623
Pons-Isocortex	-0.00091	-0.01745	-0.00931	-0.03053
Midbrain reticular nucleus-Isocortex	0	0	0	0
Nucleus accumbens-Isocortex	0	-0.22492	0.971012	-0.05068
fimbria-Ictx	0	0	0	0
Anterior cingulate area-Isocortex	-0.12035	-0.12096	-0.39791	-0.22744
Somatomotor areas-Isocortex	0.035192	-0.75115	0.652252	0.152564
Somatosensory areas-Isocortex	0.929535	-0.44231	0.971012	-0.71436
piriform area-Isocortex	0.447771	-0.92156	0.489915	-0.99505
Taenia tecta -Isocortex	0.832945	0.120962	-0.78429	0.559894
Accessory olfactory bulb glomerular layer-Isocortex	0	0	0	0.076229
Accessory olfactory bulb granular layer-Isocortex	0	0	0	0
Retrohippocampal region -Isocortex	-0.00027	-0.22492	0	-0.10875
Entorhinal area-Isocortex	-0.000011	-0.02569	-0.02975	-0.05068
Field CA1-Isocortex	0	-0.18991	-0.53688	-0.07623
Field CA3-Isocortex	-0.10297	-0.43831	-0.78882	-0.57883
Dentate gyrus-Isocortex	-0.00047	-0.10382	-0.94476	-0.26202
Field CA2 -Isocortex	0.687188	-0.75115	0.498724	-0.71436
Accessory olfactory bulb mitral layer-Isocortex	0	0	0	0.341954
Striatum -Isocortex	-0.13479	-0.12096	0.78429	-0.19251
Midbrain -Isocortex	-0.000013	-0.00178	-0.00415	-0.02319
Medulla-Isocortex	-0.000037	-0.03109	-0.02068	-0.03626
Olfactory areas -Cortical amygdalar area	0	0	0	0
Pons-Cortical amygdalar area	0	0	0	0
Midbrain reticular nucleus-Cortical amygdalar area	0	0	0	0

Nucleus accumbens-Cortical amygdalar area	0	0	0	0
fimbria-Cortical amygdalar area	0	0	0	0
Anterior cingulate area-Cortical amygdalar area	0	0	0	0
Somatomotor areas-Cortical amygdalar area	0	0	0	-0.10075
Somatosensory areas-Cortical amygdalar area	0	0.665474	0	0
piriform area-Cortical amygdalar area	0	0	0	0
Taenia tecta -Cortical amygdalar area	0	0	0	0
Accessory olfactory bulb glomerular layer-Cortical amygdalar area	0	0	0	0
Accessory olfactory bulb granular layer-Cortical amygdalar area	0	0	0	0
Retrohippocampal region -Cortical amygdalar area	0	0	0	0
Entorhinal area-Cortical amygdalar area	0	0	0	0
Field CA1-Cortical amygdalar area	0	0	0	0
Field CA3-Cortical amygdalar area	0	0	0	0
Dentate gyrus-Cortical amygdalar area	0	0	0	0
Field CA2 -Cortical amygdalar area	0	0	0	0
Accessory olfactory bulb mitral layer-Cortical amygdalar area	0	0	0	0
Striatum -Cortical amygdalar area	0	0	0	0
Midbrain -Cortical amygdalar area	0	0	0	0
Medulla-Cortical amygdalar area	0	0	0	0
Pons-Olfactory areas	0	0	0	0
Midbrain reticular nucleus-Olfactory areas	0	0	0	0
Nucleus accumbens-Olfactory areas	0	0	0	0
fimbria-Olfactory areas	0	0	0	0
Anterior cingulate area-Olfactory areas	0	0	0	-0.00143
Somatomotor areas-Olfactory areas	0.00385	-0.75115	0.944755	0.162209
Somatosensory areas-Olfactory areas	0.480748	-0.22998	0.397906	-0.48254
piriform area-Olfactory areas	0	0	0	0
Taenia tecta -Olfactory areas	0	0	0	0
Accessory olfactory bulb glomerular layer-Olfactory areas	0	0	0	0
Accessory olfactory bulb granular layer-Olfactory areas	0	0	0	0
Retrohippocampal region -Olfactory areas	0	0	0	0
Entorhinal area-Olfactory areas	0	0	0	0
Field CA1-Olfactory areas	0	0	0	0
Field CA3-Olfactory areas	0	0	0	0
Dentate gyrus-Olfactory areas	0	0	0	0

Field CA2 -Olfactory areas	0	0	0	0
Accessory olfactory bulb mitral layer-Olfactory areas	0	0	0	0
Striatum -Olfactory areas	0	0	0	0
Midbrain -Olfactory areas	0	0	0	0
Medulla-Olfactory areas	0	0	0	0
Midbrain reticular nucleus-Pons	0	0	0	0
Nucleus accumbens-Pons	0	0	0	0
fimbria-Pons	0	0	0	0
Anterior cingulate area-Pons	0	0	0	0
Somatomotor areas-Pons	-0.000024	-0.012	-0.0001	-0.000099
Somatosensory areas-Pons	-0.23397	-0.12096	-0.21816	-0.16652
piriform area-Pons	0	0	0	0
Taenia tecta -Pons	0	0	0	0
Accessory olfactory bulb glomerular layer-Pons	0	0	0	0
Accessory olfactory bulb granular layer-Pons	0	0	0	0
Retrohippocampal region -Pons	0	0	0	0
Entorhinal area-Pons	0	0	0	0
Field CA1-Pons	0	0	0	0
Field CA3-Pons	0	0	0	0
Dentate gyrus-Pons	0	0	0	0
Field CA2 -Pons	0	0	0	0
Accessory olfactory bulb mitral layer-Pons	0	0	0	0
Striatum -Pons	0	0	0	0
Midbrain -Pons	0	0	0	0
Medulla-Pons	0	0	0	0
Nucleus accumbens-Midbrain reticular nucleus	0	0	0	0
fimbria-Midbrain reticular nucleus	0	0	0	0
Anterior cingulate area-Midbrain reticular nucleus	0	0	0	0
Somatomotor areas-Midbrain reticular nucleus	0	0	0	-0.01272
Somatosensory areas-Midbrain reticular nucleus	0	0	0	0
piriform area-Midbrain reticular nucleus	0	0	0	0
Taenia tecta -Midbrain reticular nucleus	0	0	0	0
Accessory olfactory bulb glomerular layer-Midbrain reticular nucleus	0	0	0	0
Accessory olfactory bulb granular layer-Midbrain reticular nucleus	0	0	0	0

Retrohippocampal region -Midbrain reticular nucleus	0	0	0	0
Entorhinal area-Midbrain reticular nucleus	0	0	0	0
Field CA1-Midbrain reticular nucleus	0	0	0	0
Field CA3-Midbrain reticular nucleus	0	0	0	0
Dentate gyrus-Midbrain reticular nucleus	0	0	0	0
Field CA2 -Midbrain reticular nucleus	0	0	0	0
Accessory olfactory bulb mitral layer-Midbrain reticular nucleus	0	0	0	0
Striatum -Midbrain reticular nucleus	0	0	0	0
Midbrain -Midbrain reticular nucleus	0	0	0	0
Medulla-Midbrain reticular nucleus	0	0	0	0
fimbria-Nucleus accumbens	0	0	0	0
Anterior cingulate area-Nucleus accumbens	0	0	0	0
Somatomotor areas-Nucleus accumbens	0.357673	-0.48355	0	-0.32509
Somatosensory areas-Nucleus accumbens	0	0.665474	0	0
piriform area-Nucleus accumbens	0	0	0	0
Taenia tecta -Nucleus accumbens	0	0	0	0
Accessory olfactory bulb glomerular layer-Nucleus accumbens	0	0	0	0
Accessory olfactory bulb granular layer-Nucleus accumbens	0	0	0	0
Retrohippocampal region -Nucleus accumbens	0	0	0	0
Entorhinal area-Nucleus accumbens	0	0	0	0
Field CA1-Nucleus accumbens	0	0	0	0
Field CA3-Nucleus accumbens	0	0	0	0
Dentate gyrus-Nucleus accumbens	0	0	0	0
Field CA2 -Nucleus accumbens	0	0	0	0
Accessory olfactory bulb mitral layer-Nucleus accumbens	0	0	0	0
Striatum -Nucleus accumbens	0	0	0	0
Midbrain -Nucleus accumbens	0	0	0	0
Medulla-Nucleus accumbens	0	0	0	0
Anterior cingulate area-fimbria	0	0	0	0
Somatomotor areas-fimbria	0	0	0	0
Somatosensory areas-fimbria	0	0	0	0
piriform area-fimbria	0	0	0	0
Taenia tecta -fimbria	0	0	0	0
Accessory olfactory bulb glomerular layer-fimbria	0	0	0	0

Accessory olfactory bulb granular layer-fimbria	0	0	0	0
Retrohippocampal region -fimbria	0	0	0	0
Entorhinal area-fimbria	0	0	0	0
Field CA1-fimbria	0	0	0	0
Field CA3-fimbria	0	0	0	0
Dentate gyrus-fimbria	0	0	0	0
Field CA2 -fimbria	0	0	0	0
Accessory olfactory bulb mitral layer-fimbria	0	0	0	0
Striatum -fimbria	0	0	0	0
Midbrain -fimbria	0	0	0	0
Medulla-fimbria	0	0	0	0
Somatomotor areas-Anterior cingulate area	0.02696	0.085473	0.707432	0.62355
Somatosensory areas-Anterior cingulate area	-0.06111	-0.57076	-0.58144	-0.12618
piriform area-Anterior cingulate area	0	0	0	-0.20696
Taenia tecta -Anterior cingulate area	0	0	0	-0.15256
Accessory olfactory bulb glomerular layer-Anterior cingulate area	0	0	0	0
Accessory olfactory bulb granular layer-Anterior cingulate area	0	0	0	0
Retrohippocampal region -Anterior cingulate area	0	0	0	0
Entorhinal area-Anterior cingulate area	0	0	0	-0.00143
Field CA1-Anterior cingulate area	0	0	0	0
Field CA3-Anterior cingulate area	0	0	0	0
Dentate gyrus-Anterior cingulate area	0	0	0	0
Field CA2 -Anterior cingulate area	0	0	0	0
Accessory olfactory bulb mitral layer-Anterior cingulate area	0	0	0	0
Striatum -Anterior cingulate area	-0.00126	-0.04812	-0.02975	-0.04079
Midbrain -Anterior cingulate area	0	0	0	0
Medulla-Anterior cingulate area	0	0	0	0
Somatosensory areas-Somatomotor areas	-0.76113	-0.62725	0.497038	0.714355
piriform area-Somatomotor areas	0.141224	0.998907	0.270575	0.812856
Taenia tecta -Somatomotor areas	0.026768	0.10382	-0.97101	0.538952
Accessory olfactory bulb glomerular layer-Somatomotor areas	0	0	0	0.107526
Accessory olfactory bulb granular layer-Somatomotor areas	0	0	0	0.227438
Retrohippocampal region -Somatomotor areas	-0.00126	0	-0.43671	-0.01859
Entorhinal area-Somatomotor areas	-0.000011	0	-0.22365	-0.03166

Field CA1-Somatomotor areas	-0.01252	0	-0.37135	-0.22813
Field CA3-Somatomotor areas	-0.69115	0	-0.78882	-0.22516
Dentate gyrus-Somatomotor areas	-0.053	0	-0.54909	-0.43803
Field CA2 -Somatomotor areas	0.037854	0.784015	0.112922	0.62559
Accessory olfactory bulb mitral layer-Somatomotor areas	0	0	0	0.070706
Striatum -Somatomotor areas	-0.53064	-0.22162	0.87231	-0.85339
Midbrain -Somatomotor areas	-0.000016	-0.00044	-0.0672	-0.00143
Medulla-Somatomotor areas	-0.00027	-0.00044	-0.02975	-0.00143
piriform area-Somatosensory areas	0.375491	0.404427	0.352254	0
Taenia tecta -Somatosensory areas	0.100149	0.017147	0.698812	0.06531
Accessory olfactory bulb glomerular layer-Somatosensory areas	0	0	0	0
Accessory olfactory bulb granular layer-Somatosensory areas	0	0	0	0
Retrohippocampal region -Somatosensory areas	-0.15693	0	-0.35225	-0.21453
Entorhinal area-Somatosensory areas	-0.14122	-0.00797	-0.20659	-0.02635
Field CA1-Somatosensory areas	-0.06298	0	0.801763	-0.4973
Field CA3--Somatosensory areas	0.800868	-0.75115	-0.99542	-0.45075
Dentate gyrus-Somatosensory areas	-0.47475	-0.24441	-0.97681	-0.16547
Field CA2 -Somatosensory areas	0.331538	-0.316	0.522	-0.66323
Accessory olfactory bulb mitral layer-Somatosensory areas	0	0	0	0
Striatum --Somatosensory areas	0.348631	-0.28622	0.381974	-0.56445
Midbrain --Somatosensory areas	-0.01267	-0.04812	-0.03948	-0.05441
Medulla-Somatosensory areas	-0.14122	0	-0.22365	-0.16891
Taenia tecta -piriform area	0	0	0	0
Accessory olfactory bulb glomerular layer-piriform area	0	0	0	0
Accessory olfactory bulb granular layer-piriform area	0	0	0	0
Retrohippocampal region -piriform area	0	0	0	0
Entorhinal area-piriform area	0	0	0	0
Field CA1-piriform area	0	0	0	0
Field CA3-piriform area	0	0	0	0
Dentate gyrus-piriform area	0	0	0	0
Field CA2 -piriform area	0	0	0	0
Accessory olfactory bulb mitral layer-piriform area	0	0	0	0
Striatum -piriform area	0	0	0	0
Midbrain -piriform area	0	0	0	0

Medulla-piriform area	0	0	0	0
Accessory olfactory bulb glomerular layer-Taenia tecta	0	0	0	0
Accessory olfactory bulb granular layer-Taenia tecta	0	0	0	0
Retrohippocampal region -Taenia tecta	0	0	0	0
Entorhinal area-Taenia tecta	0	0	0	0
Field CA1-Taenia tecta	0	0	0	0
Field CA3-Taenia tecta	0	0	0	0
Dentate gyrus-Taenia tecta	0	0	0	0
Field CA2 -Taenia tecta	0	0	0	0
Accessory olfactory bulb mitral layer-Taenia tecta	0	0	0	0
Striatum -Taenia tecta	0	0	0	0
Midbrain -Taenia tecta	0	0	0	0
Medulla-Taenia tecta	0	0	0	0
Accessory olfactory bulb granular layer-Accessory olfactory bulb glomerular layer	0	0	0	0
Retrohippocampal region -Accessory olfactory bulb glomerular layer	0	0	0	0
Entorhinal area-Accessory olfactory bulb glomerular layer	0	0	0	0
Field CA1-Accessory olfactory bulb glomerular layer	0	0	0	0
Field CA3-Accessory olfactory bulb glomerular layer	0	0	0	0
Dentate gyrus-Accessory olfactory bulb glomerular layer	0	0	0	0
Field CA2 -Accessory olfactory bulb glomerular layer	0	0	0	0
Accessory olfactory bulb mitral layer-Accessory olfactory bulb glomerular layer	0	0	0	0
Striatum -Accessory olfactory bulb glomerular layer	0	0	0	0
Midbrain -Accessory olfactory bulb glomerular layer	0	0	0	0
Medulla-Accessory olfactory bulb glomerular layer	0	0	0	0
Retrohippocampal region -Accessory olfactory bulb granular layer	0	0	0	0
Entorhinal area-Accessory olfactory bulb granular layer	0	0	0	0
Field CA1-Accessory olfactory bulb granular layer	0	0	0	0
Field CA3-Accessory olfactory bulb granular layer	0	0	0	0
Dentate gyrus-Accessory olfactory bulb granular layer	0	0	0	0
Field CA2 -Accessory olfactory bulb granular layer	0	0	0	0
Accessory olfactory bulb mitral layer-Accessory olfactory bulb granular layer	0	0	0	0
Striatum -Accessory olfactory bulb granular layer	0	0	0	0

Midbrain -Accessory olfactory bulb granular layer	0	0	0	0
Medulla-Accessory olfactory bulb granular layer	0	0	0	0
Entorhinal area-Retrohippocampal region	0	0	0	0
Field CA1-Retrohippocampal region	0	0	0	0
Field CA3-Retrohippocampal region	0	0	0	0
Dentate gyrus-Retrohippocampal region	0	0	0	0
Field CA2 -Retrohippocampal region	0	0	0	0
Accessory olfactory bulb mitral layer-Retrohippocampal region	0	0	0	0
Striatum -Retrohippocampal region	0	0	0	0
Midbrain -Retrohippocampal region	0	0	0	0
Medulla-Retrohippocampal region	0	0	0	0
Field CA1-Entorhinal area	0	0	0	0
Field CA3-Entorhinal area	0	0	0	0
Dentate gyrus-Entorhinal area	0	0	0	0
Field CA2 -Entorhinal area	0	0	0	0
Accessory olfactory bulb mitral layer-Entorhinal area	0	0	0	0
Striatum -Entorhinal area	0	0	0	0
Midbrain -Entorhinal area	0	0	0	0
Medulla-Entorhinal area	0	0	0	0
Field CA3-Field CA1	0	0	0	0
Dentate gyrus-Field CA1	0	0	0	0
Field CA2 -Field CA1	0	0	0	0
Accessory olfactory bulb mitral layer-Field CA1	0	0	0	0
Striatum -Field CA1	0	0	0	0
Midbrain -Field CA1	0	0	0	0
Medulla-Field CA1	0	0	0	0
Dentate gyrus-Field CA3	0	0	0	0
Field CA2 -Field CA3	0	0	0	0
Accessory olfactory bulb mitral layer-Field CA3	0	0	0	0
Striatum -Field CA3	0	0	0	0
Midbrain -Field CA3	0	0	0	0
Medulla-Field CA3	0	0	0	0

Field CA2 -Dentate gyrus	0	0	0	0
Accessory olfactory bulb mitral layer-Dentate gyrus	0	0	0	0
Striatum -Dentate gyrus	0	0	0	0
Midbrain -Dentate gyrus	0	0	0	0
Medulla-Dentate gyrus	0	0	0	0
Accessory olfactory bulb mitral layer-Field CA2	0	0	0	0
Striatum -Field CA2	0	0	0	0
Midbrain -Field CA2	0	0	0	0
Medulla-Field CA2	0	0	0	0
Striatum -Accessory olfactory bulb mitral layer	0	0	0	0
Midbrain -Accessory olfactory bulb mitral layer	0	0	0	0
Medulla-Accessory olfactory bulb mitral layer	0	0	0	0
Midbrain -Striatum	0	0	0	0
Medulla-Striatum	0	0	0	0
Medulla-Midbrain	0	0	0	0

S14. Adjust p values for group comparisons of pair-wised connectivity for Somatosensory Cortex.

Region Connections	Sensory Cortex			
	Left Ipsilateral	Left Contralateral	Right Contralateral	Right Ipsilateral
Caudoputamen-corpus callosum	-0.0083	-0.00989	-0.71294	-0.6547
anterior commissure olfactory limb-corpus callosum	0	0	0	0
pallidum -corpus callosum	-0.15473	0	-0.49785	-0.11692
internal capsule-corpus callosum	-0.06705	0	0	-0.5714
Thalamus-corpus callosum	-0.03739	-0.07396	0	-0.28843
Cerebellum-corpus callosum	-0.05773	0	-0.22112	-0.1889
Superior colliculus-corpus callosum	-0.04853	-0.29229	-0.42975	-0.09371
ventricular systems-corpus callosum	-0.02734	-0.04985	-0.1407	0.883305
Hypothalamus -corpus callosum	-0.06771	-0.40255	0	-0.10637
Inferior colliculus -corpus callosum	-0.18834	0	0	-0.09095
periaqueductal gray-corpus callosum	-0.00518	0	0	0
Isocortex -corpus callosum	-0.19589	-0.45769	0.712935	-0.48743
Cortical amygdalar area -corpus callosum	0	0	0	0
Olfactory areas -corpus callosum	-0.20048	0	0	-0.11087
Pons-corpus callosum	-0.02729	0	-0.33587	0
Midbrain reticular nucleus-corpus callosum	0	0	0	0
Nucleus accumbens-corpus callosum	-0.41009	0	0	-0.01481
fimbria-corpus callosum	0	0	0	0
Anterior cingulate area-corpus callosum	-0.06705	-0.30558	0	-0.15171
Somatomotor areas-corpus callosum	-0.13744	0.895968	-0.71294	-0.79706
Somatosensory areas-corpus callosum	-0.42626	0.477429	0.382815	-0.25467
piriform area-corpus callosum	0.289782	0	0	0.154768
Taenia tecta -corpus callosum	0	0	0	-0.33579
Accessory olfactory bulb glomerular layer-corpus callosum	0	0	0	0
Accessory olfactory bulb granular layer-corpus callosum	0	0	0	0
Retrohippocampal region -corpus callosum	0	0	0	0
Entorhinal area-corpus callosum	0	0	0	-0.12802
Field CA1-corpus callosum	-0.0624	0	0	-0.56431
Field CA3-corpus callosum	-0.06705	0	0	-0.25467
Dentate gyrus-corpus callosum	-0.38831	0	0	-0.2495
Field CA2 -corpus callosum	-0.67039	0	0	0.534618
Accessory olfactory bulb mitral layer-corpus callosum	0	0	0	0

Striatum -corpus callosum	-0.22857	-0.09154	-0.441	-0.25467
Midbrain -corpus callosum	-0.00777	-0.10892	0	-0.07569
Medulla-corpus callosum	0	0	-0.22112	-0.25467
anterior commissure olfactory limb-Caudoputamen	0	0	0	0
pallidum -Caudoputamen	-0.269002	0	0	-0.534618
internal capsule-Caudoputamen	0	0	0	0
Thalamus-Caudoputamen	-0.357908	-0.603092	-0.712935	-0.863308
Cerebellum-Caudoputamen	0	-0.331743	-0.129392	-0.842028
Superior colliculus-Caudoputamen	-0.626489	-0.457692	-0.297205	-0.355148
ventricular systems-Caudoputamen	-0.405798	-0.491372	-0.078925	-0.850081
Hypothalamus -Caudoputamen	0	0	-0.814954	-0.254673
Inferior colliculus -Caudoputamen	0	0	0	0
periaqueductal gray-Caudoputamen	0	0	0	0
Isocortex -Caudoputamen	-0.901682	-0.636642	-0.942001	0.873366
Cortical amygdalar area -Caudoputamen	0	0	0	0
Olfactory areas -Caudoputamen	-0.356621	0	-0.440996	-0.28906
Pons-Caudoputamen	0	0	-0.001344	0
Midbrain reticular nucleus-Caudoputamen	0	0	0	0
Nucleus accumbens-Caudoputamen	0	0	0	0
fimbria-Caudoputamen	0	0	0	0
Anterior cingulate area-Caudoputamen	-0.269002	-0.277294	0	-0.071477
Somatomotor areas-Caudoputamen	-0.206963	0.986991	-0.406346	-0.802106
Somatosensory areas-Caudoputamen	0.584577	-0.714658	0.843222	-0.658304
piriform area-Caudoputamen	0.067049	0	0	0
Taenia tecta -Caudoputamen	0	0	0	0
Accessory olfactory bulb glomerular layer-Caudoputamen	0	0	0	0
Accessory olfactory bulb granular layer-Caudoputamen	0	0	0	0
Retrohippocampal region -Caudoputamen	0	0	0	0
Entorhinal area-Caudoputamen	0	0	0	-0.974122
Field CA1-Caudoputamen	-0.17096	0	0	0.802106
Field CA3-Caudoputamen	-0.141472	0	0	0.886925
Dentate gyrus-Caudoputamen	-0.536856	0	0	0.744778
Field CA2 -Caudoputamen	-0.168966	0	0	0.974122
Accessory olfactory bulb mitral layer-Caudoputamen	0	0	0	0
Striatum -Caudoputamen	-0.890265	-0.035987	-0.440996	-0.365209
Midbrain -Caudoputamen	-0.10245	-0.298183	-0.078925	-0.154768

Medulla-Caudoputamen	0	0	0	0
pallidum -anterior commissure olfactory limb	0	0	0	0
internal capsule-anterior commissure olfactory limb	0	0	0	0
Thalamus-anterior commissure olfactory limb	0	0	0	0
Cerebellum-anterior commissure olfactory limb	0	0	0	0
Superior colliculus-anterior commissure olfactory limb	0	0	0	0
ventricular systems-anterior commissure olfactory limb	0	0	0	0
Hypothalamus -anterior commissure olfactory limb	0	0	0	0
Inferior colliculus -anterior commissure olfactory limb	0	0	0	0
periaqueductal gray-anterior commissure olfactory limb	0	0	0	0
Isocortex -anterior commissure olfactory limb	0	0	0	-0.249495
Cortical amygdalar area -anterior commissure olfactory limb	0	0	0	0
Olfactory areas -anterior commissure olfactory limb	0	0	0	0
Pons-anterior commissure olfactory limb	0	0	0	0
Midbrain reticular nucleus-anterior commissure olfactory limb	0	0	0	0
Nucleus accumbens-anterior commissure olfactory limb	0	0	0	0
fimbria-anterior commissure olfactory limb	0	0	0	0
Anterior cingulate area-anterior commissure olfactory limb	0	0	0	0
Somatomotor areas-anterior commissure olfactory limb	0	0	0	0
Somatosensory areas-anterior commissure olfactory limb	0	0	0	0
piriform area-anterior commissure olfactory limb	0	0	0	0
Taenia tecta -anterior commissure olfactory limb	0	0	0	0
Accessory olfactory bulb glomerular layer-anterior commissure olfactory limb	0	0	0	0
Accessory olfactory bulb granular layer-anterior commissure olfactory limb	0	0	0	0
Retrohippocampal region -anterior commissure olfactory limb	0	0	0	0
Entorhinal area-anterior commissure olfactory limb	0	0	0	0
Field CA1-anterior commissure olfactory limb	0	0	0	0
Field CA3-anterior commissure olfactory limb	0	0	0	0
Dentate gyrus-anterior commissure olfactory limb	0	0	0	0
Field CA2 -anterior commissure olfactory limb	0	0	0	0
Accessory olfactory bulb mitral layer-anterior commissure olfactory limb	0	0	0	0
Striatum -anterior commissure olfactory limb	0	0	0	0
Midbrain -anterior commissure olfactory limb	0	0	0	0
Medulla-anterior commissure olfactory limb	0	0	0	0

internal capsule-pallidum	0	0	0	0
Thalamus-pallidum	0	0	0	0
Cerebellum-pallidum	0	0	0	0
Superior colliculus-pallidum	0	0	0	0
ventricular systems-pallidum	0	0	0	0
Hypothalamus -pallidum	0	0	0	0
Inferior colliculus -pallidum	0	0	0	0
periaqueductal gray-pallidum	0	0	0	0
Isocortex -pallidum	-0.027339	-0.423433	-0.359981	-0.055941
Cortical amygdalar area -pallidum	0	0	0	0
Olfactory areas -pallidum	0	0	0	0
Pons-pallidum	0	0	0	0
Midbrain reticular nucleus-pallidum	0	0	0	0
Nucleus accumbens-pallidum	0	0	0	0
fimbria-pallidum	0	0	0	0
AVA-pallidum	0	0	0	0
Somatomotor areas-pallidum	0	0	0	-0.121231
Somatosensory areas-pallidum	-0.950783	-0.603092	-0.942001	-0.071477
piriform area-pallidum	0	0	0	0
Taenia tecta -pallidum	0	0	0	0
Accessory olfactory bulb glomerular layer-pallidum	0	0	0	0
Accessory olfactory bulb granular layer-pallidum	0	0	0	0
Retrohippocampal region -pallidum	0	0	0	0
Entorhinal area-pallidum	0	0	0	0
Field CA1-pallidum	0	0	0	0
Field CA3-pallidum	0	0	0	0
Dentate gyrus-pallidum	0	0	0	0
Field CA2 -pallidum	0	0	0	0
Accessory olfactory bulb mitral layer-pallidum	0	0	0	0
Striatum -pallidum	0	0	0	0
Midbrain -pallidum	0	0	0	0
Medulla-pallidum	0	0	0	0
Thalamus-internal capsule	0	0	0	0
Cerebellum-internal capsule	0	0	0	0
Superior colliculus-internal capsule	0	0	0	0

ventricular systems-internal capsule	0	0	0	0
Hypothalamus -internal capsule	0	0	0	0
Inferior colliculus -internal capsule	0	0	0	0
periaqueductal gray-internal capsule	0	0	0	0
Isocortex -internal capsule	-0.024469	0	0	-0.677442
Cortical amygdalar area -internal capsule	0	0	0	0
Olfactory areas -internal capsule	0	0	0	0
Pons-internal capsule	0	0	0	0
Midbrain reticular nucleus-internal capsule	0	0	0	0
Nucleus accumbens-internal capsule	0	0	0	0
fimbria-internal capsule	0	0	0	0
Anterior cingulate area-internal capsule	0	0	0	0
Somatomotor areas-internal capsule	0	0	0	0
Somatosensory areas-internal capsule	-0.464285	0.277294	0	-0.549698
piriform area-internal capsule	0	0	0	0
Taenia tecta -internal capsule	0	0	0	0
Accessory olfactory bulb glomerular layer-internal capsule	0	0	0	0
Accessory olfactory bulb granular layer-internal capsule	0	0	0	0
Retrohippocampal region -internal capsule	0	0	0	0
Entorhinal area-internal capsule	0	0	0	0
Field CA1-internal capsule	0	0	0	0
Field CA3-internal capsule	0	0	0	0
Dentate gyrus-internal capsule	0	0	0	0
Field CA2 -internal capsule	0	0	0	0
Accessory olfactory bulb mitral layer-internal capsule	0	0	0	0
Striatum -internal capsule	0	0	0	0
Midbrain --internal capsule	0	0	0	0
Medulla-internal capsule	0	0	0	0
Cerebellum-Thalamus	0	0	0	0
Superior colliculus-Thalamus	0	0	0	0
ventricular systems-Thalamus	0	0	0	0
Hypothalamus -Thalamus	0	0	0	0
Inferior colliculus -Thalamus	0	0	0	0
periaqueductal gray-Thalamus	0	0	0	0
Isocortex -Thalamus	-0.06705	-0.12909	-0.88674	-0.01733

Cortical amygdalar area -Thalamus	0	0	0	0
Olfactory areas -Thalamus	0	0	0	0
Pons-Thalamus	0	0	0	0
Midbrain reticular nucleus-Thalamus	0	0	0	0
Nucleus accumbens-Thalamus	0	0	0	0
fimbria-Thalamus	0	0	0	0
Anterior cingulate area-Thalamus	0	0	0	0
Somatomotor areas-Thalamus	-0.06705	0	0	-0.17242
Somatosensory areas-Thalamus	-0.66603	-0.57183	0.995948	-0.01926
piriform area-Thalamus	0	0	0	0
Taenia tecta -Thalamus	0	0	0	0
Accessory olfactory bulb glomerular layer-Thalamus	0	0	0	0
Accessory olfactory bulb granular layer-Thalamus	0	0	0	0
Retrohippocampal region -Thalamus	0	0	0	0
Entorhinal area-Thalamus	0	0	0	0
Field CA1-Thalamus	-0.06705	0	0	0
Field CA3-Thalamus	0	0	0	0
Dentate gyrus-Thalamus	0	0	0	0
Field CA2 -Thalamus	0	0	0	0
Accessory olfactory bulb mitral layer-Thalamus	0	0	0	0
Striatum -Thalamus	0	0	0	0
Midbrain -Thalamus	0	0	0	0
Medulla-Thalamus	0	0	0	0
Superior colliculus-Cerebellum	0	0	0	0
ventricular systems-Cerebellum	0	0	0	0
Hypothalamus -Cerebellum	0	0	0	0
Inferior colliculus -Cerebellum	0	0	0	0
periaqueductal gray-Cerebellum	0	0	0	0
Isocortex -Cerebellum	-0.06771	-0.03599	-0.07893	-0.00784
Cortical amygdalar area -Cerebellum	0	0	0	0
Olfactory areas -Cerebellum	0	0	0	0
Pons-Cerebellum	0	0	0	0
Midbrain reticular nucleus-Cerebellum	0	0	0	0
Nucleus accumbens-Cerebellum	0	0	0	0
fimbria-Cerebellum	0	0	0	0

Anterior cingulate area-Cerebellum	0	0	0	0
Somatomotor areas-Cerebellum	-0.09569	-0.32029	-0.1407	-0.00281
Somatosensory areas-Cerebellum	-0.44284	-0.07396	-0.1007	-0.0062
piriform area-Cerebellum	0	0	0	0
Taenia tecta -Cerebellum	0	0	0	0
Accessory olfactory bulb glomerular layer-Cerebellum	0	0	0	0
Accessory olfactory bulb granular layer-Cerebellum	0	0	0	0
Retrohippocampal region -Cerebellum	0	0	0	0
Entorhinal area-Cerebellum	0	0	0	0
Field CA1-Cerebellum	0	0	0	0
Field CA3-Cerebellum	0	0	0	0
Dentate gyrus-Cerebellum	0	0	0	0
Field CA2 -Cerebellum	0	0	0	0
Accessory olfactory bulb mitral layer-Cerebellum	0	0	0	0
Striatum -Cerebellum	0	0	0	0
Midbrain -Cerebellum	0	0	0	0
Medulla-Cerebellum	0	0	0	0
ventricular systems-Superior colliculus	0	0	0	0
Hypothalamus -Superior colliculus	0	0	0	0
Inferior colliculus -Superior colliculus	0	0	0	0
periaqueductal gray-Superior colliculus	0	0	0	0
Isocortex -Superior colliculus	-0.20048	-0.07396	-0.42975	-0.03146
Cortical amygdalar area -Superior colliculus	0	0	0	0
Olfactory areas -Superior colliculus	0	0	0	0
Pons-Superior colliculus	0	0	0	0
Midbrain reticular nucleus-Superior colliculus	0	0	0	0
Nucleus accumbens-Superior colliculus	0	0	0	0
fimbria-Superior colliculus	0	0	0	0
Anterior cingulate area-Superior colliculus	0	0	0	0
Somatomotor areas-Superior colliculus	-0.14147	-0.45769	0	-0.0139
Somatosensory areas-Superior colliculus	-0.53686	-0.12909	-0.49685	-0.04425
piriform area-Superior colliculus	0	0	0	0
Taenia tecta -Superior colliculus	0	0	0	0
Accessory olfactory bulb glomerular layer-Superior colliculus	0	0	0	0
Accessory olfactory bulb granular layer-Superior colliculus	0	0	0	0

Retrohippocampal region -Superior colliculus	0	0	0	0
Entorhinal area-Superior colliculus	0	0	0	0
Field CA1-Superior colliculus	0	0	0	0
Field CA3-Superior colliculus	0	0	0	0
Dentate gyrus-Superior colliculus	0	0	0	0
Field CA2 -Superior colliculus	0	0	0	0
Accessory olfactory bulb mitral layer-Superior colliculus	0	0	0	0
Striatum -Superior colliculus	0	0	0	0
Midbrain -Superior colliculus	0	0	0	0
Medulla-Superior colliculus	0	0	0	0
Hypothalamus -ventricular systems	0	0	0	0
Inferior colliculus -ventricular systems	0	0	0	0
periaqueductal gray-ventricular systems	0	0	0	0
Isocortex -ventricular systems	-0.304667	-0.277294	0.716061	-0.28456
Cortical amygdalar area -ventricular systems	0	0	0	0
Olfactory areas -ventricular systems	0	0	0	0
Pons-ventricular systems	0	0	0	0
Midbrain reticular nucleus-ventricular systems	0	0	0	0
Nucleus accumbens-ventricular systems	0	0	0	0
fimbria-ventricular systems	0	0	0	0
Anterior cingulate area-ventricular systems	0	0	0	0
Somatomotor areas-ventricular systems	-0.123743	-0.313976	-0.691227	-0.732067
Somatosensory areas-ventricular systems	0.902424	-0.477429	0.646569	-0.974122
piriform area-ventricular systems	0	0	0	0
Taenia tecta -ventricular systems	0	0	0	0
Accessory olfactory bulb glomerular layer-ventricular systems	0	0	0	0
Accessory olfactory bulb granular layer-ventricular systems	0	0	0	0
Retrohippocampal region -ventricular systems	0	0	0	0
Entorhinal area-ventricular systems	0	0	0	0
Field CA1-ventricular systems	0	0	0	0
Field CA3-ventricular systems	0	0	0	0
Dentate gyrus-ventricular systems	0	0	0	0
Field CA2 -ventricular systems	0	0	0	0
Accessory olfactory bulb mitral layer-ventricular systems	0	0	0	0
Striatum -ventricular systems	0	0	0	-0.188899

Midbrain -ventricular systems	0	0	0	0
Medulla-ventricular systems	0	0	0	0
Inferior colliculus -Hypothalamus	0	0	0	0
periaqueductal gray-Hypothalamus	0	0	0	0
Isocortex -Hypothalamus	-0.000043	-0.129089	-0.16968	-0.002813
Cortical amygdalar area -Hypothalamus	0	0	0	0
Olfactory areas -Hypothalamus	0	0	0	0
Pons-Hypothalamus	0	0	0	0
Midbrain reticular nucleus-Hypothalamus	0	0	0	0
Nucleus accumbens-Hypothalamus	0	0	0	0
fimbria-Hypothalamus	0	0	0	0
Anterior cingulate area-Hypothalamus	0	0	0	0
Somatomotor areas-Hypothalamus	-0.381245	0	0	-0.083972
Somatosensory areas-Hypothalamus	-0.464285	-0.443198	-0.429751	-0.057992
piriform area-Hypothalamus	0	0	0	0
Taenia tecta -Hypothalamus	0	0	0	0
Accessory olfactory bulb glomerular layer-Hypothalamus	0	0	0	0
Accessory olfactory bulb granular layer-Hypothalamus	0	0	0	0
Retrohippocampal region -Hypothalamus	0	0	0	0
Entorhinal area-Hypothalamus	0	0	0	0
Field CA1-Hypothalamus	0	0	0	0
Field CA3-Hypothalamus	0	0	0	0
Dentate gyrus-Hypothalamus	0	0	0	0
Field CA2 -Hypothalamus	0	0	0	0
Accessory olfactory bulb mitral layer-Hypothalamus	0	0	0	0
Striatum -Hypothalamus	0	0	0	0
Midbrain -Hypothalamus	0	0	0	0
Medulla-Hypothalamus	0	0	0	0
periaqueductal gray-Inferior colliculus	0	0	0	0
Isocortex -Inferior colliculus	-0.02734	-0.15841	0	-0.06753
Cortical amygdalar area -Inferior colliculus	0	0	0	0
Olfactory areas -Inferior colliculus	0	0	0	0
Pons-Inferior colliculus	0	0	0	0
Midbrain reticular nucleus-Inferior colliculus	0	0	0	0

Nucleus accumbens-Inferior colliculus	0	0	0	0
fimbria-Inferior colliculus	0	0	0	0
Anterior cingulate area-Inferior colliculus	0	0	0	0
Somatomotor areas-Inferior colliculus	0	0	0	-0.00153
Somatosensory areas-Inferior colliculus	-0.02699	-0.31398	0	-0.02076
piriform area-Inferior colliculus	0	0	0	0
Taenia tecta -Inferior colliculus	0	0	0	0
Accessory olfactory bulb glomerular layer-Inferior colliculus	0	0	0	0
Accessory olfactory bulb granular layer-Inferior colliculus	0	0	0	0
Retrohippocampal region -Inferior colliculus	0	0	0	0
Entorhinal area-Inferior colliculus	0	0	0	0
Field CA1-Inferior colliculus	0	0	0	0
Field CA3-Inferior colliculus	0	0	0	0
Dentate gyrus-Inferior colliculus	0	0	0	0
Field CA2 -Inferior colliculus	0	0	0	0
Accessory olfactory bulb mitral layer-Inferior colliculus	0	0	0	0
Striatum -Inferior colliculus	0	0	0	0
Midbrain -Inferior colliculus	0	0	0	0
Medulla-Inferior colliculus	0	0	0	0
Isocortex -periaqueductal gray	-0.02685	-0.00321	-0.23566	-0.00587
Cortical amygdalar area -periaqueductal gray	0	0	0	0
Olfactory areas -periaqueductal gray	0	0	0	0
Pons-periaqueductal gray	0	0	0	0
Midbrain reticular nucleus-periaqueductal gray	0	0	0	0
Nucleus accumbens-periaqueductal gray	0	0	0	0
fimbria-periaqueductal gray	0	0	0	0
Anterior cingulate area-periaqueductal gray	0	0	0	0
Somatomotor areas-periaqueductal gray	0	0	0	-0.02693
Somatosensory areas-periaqueductal gray	-0.03951	-0.00572	-0.42975	-0.00287
piriform area-periaqueductal gray	0	0	0	0
Taenia tecta -periaqueductal gray	0	0	0	0
Accessory olfactory bulb glomerular layer-periaqueductal gray	0	0	0	0
Accessory olfactory bulb granular layer-periaqueductal gray	0	0	0	0
Retrohippocampal region -periaqueductal gray	0	0	0	0
Entorhinal area-periaqueductal gray	0	0	0	0

Field CA1-periaqueductal gray	0	0	0	0
Field CA3-periaqueductal gray	0	0	0	0
Dentate gyrus-periaqueductal gray	0	0	0	0
Field CA2 -periaqueductal gray	0	0	0	0
Accessory olfactory bulb mitral layer-periaqueductal gray	0	0	0	0
Striatum -periaqueductal gray	0	0	0	0
Midbrain -periaqueductal gray	0	0	0	0
Medulla-periaqueductal gray	0	0	0	0
Cortical amygdalar area -Isocortex	0.950783	0	-0.86366	-0.01926
Olfactory areas -Isocortex	-0.02447	-0.39174	-0.71606	-0.06641
Pons-Isocortex	-0.00777	-0.00487	-0.07893	-0.00265
Midbrain reticular nucleus-Isocortex	-0.05287	0	0	-0.02152
Nucleus accumbens-Isocortex	-0.0083	-0.70962	0.995948	-0.11087
fimbria-Ictx	0	0	0	-0.42684
Anterior cingulate area-Isocortex	-0.03739	-0.60309	-0.57984	-0.11038
Somatomotor areas-Isocortex	0.229976	-0.97844	0.440996	0.487426
Somatosensory areas-Isocortex	-0.85065	-0.17779	0.423824	0.886925
piriform area-Isocortex	-0.95078	0.571828	0.335868	0.827126
Taenia tecta -Isocortex	-0.20496	0.298183	-0.96537	-0.85008
Accessory olfactory bulb glomerular layer-Isocortex	0	0	0	0
Accessory olfactory bulb granular layer-Isocortex	0	0	0	0
Retrohippocampal region -Isocortex	-0.09569	-0.00321	-0.42975	-0.0062
Entorhinal area-Isocortex	-0.01392	-0.00321	-0.441	-0.02693
Field CA1-Isocortex	-0.62649	-0.00321	0.429751	-0.25467
Field CA3-Isocortex	-0.62337	-0.00321	0.995948	-0.09738
Dentate gyrus-Isocortex	-0.65823	-0.01212	0.712935	-0.1889
Field CA2 -Isocortex	-0.53182	-0.98699	0.920817	-0.58552
Accessory olfactory bulb mitral layer-Isocortex	0	0	0	0
Striatum -Isocortex	-0.00657	-0.00819	0.684167	-0.06753
Midbrain -Isocortex	-0.00777	-0.00321	-0.1007	-0.00229
Medulla-Isocortex	-0.01392	-0.00321	-0.12939	-0.00822
Olfactory areas -Cortical amygdalar area	0	0	0	0
Pons-Cortical amygdalar area	0	0	0	0
Midbrain reticular nucleus-Cortical amygdalar area	0	0	0	0

Nucleus accumbens-Cortical amygdalar area	0	0	0	0
fimbria-Cortical amygdalar area	0	0	0	0
Anterior cingulate area-Cortical amygdalar area	0	0	0	0
Somatomotor areas-Cortical amygdalar area	0	0	0	0
Somatosensory areas-Cortical amygdalar area	0.694974	-0.33174	0	-0.00975
piriform area-Cortical amygdalar area	0	0	0	0
Taenia tecta -Cortical amygdalar area	0	0	0	0
Accessory olfactory bulb glomerular layer-Cortical amygdalar area	0	0	0	0
Accessory olfactory bulb granular layer-Cortical amygdalar area	0	0	0	0
Retrohippocampal region -Cortical amygdalar area	0	0	0	0
Entorhinal area-Cortical amygdalar area	0	0	0	0
Field CA1-Cortical amygdalar area	0	0	0	0
Field CA3-Cortical amygdalar area	0	0	0	0
Dentate gyrus-Cortical amygdalar area	0	0	0	0
Field CA2 -Cortical amygdalar area	0	0	0	0
Accessory olfactory bulb mitral layer-Cortical amygdalar area	0	0	0	0
Striatum -Cortical amygdalar area	0	0	0	0
Midbrain -Cortical amygdalar area	0	0	0	0
Medulla-Cortical amygdalar area	0	0	0	0
Pons-Olfactory areas	0	0	0	0
Midbrain reticular nucleus-Olfactory areas	0	0	0	0
Nucleus accumbens-Olfactory areas	0	0	0	0
fimbria-Olfactory areas	0	0	0	0
Anterior cingulate area-Olfactory areas	0	0	0	0
Somatomotor areas-Olfactory areas	0.989797	0	0	-0.32065
Somatosensory areas-Olfactory areas	-0.06557	-0.12685	0.429751	-0.05799
piriform area-Olfactory areas	0	0	0	0
Taenia tecta -Olfactory areas	0	0	0	0
Accessory olfactory bulb glomerular layer-Olfactory areas	0	0	0	0
Accessory olfactory bulb granular layer-Olfactory areas	0	0	0	0
Retrohippocampal region -Olfactory areas	0	0	0	0
Entorhinal area-Olfactory areas	0	0	0	0
Field CA1-Olfactory areas	0	0	0	0
Field CA3-Olfactory areas	0	0	0	0
Dentate gyrus-Olfactory areas	0	0	0	0

Field CA2 -Olfactory areas	0	0	0	0
Accessory olfactory bulb mitral layer-Olfactory areas	0	0	0	0
Striatum -Olfactory areas	0	0	0	0
Midbrain -Olfactory areas	0	0	0	0
Medulla-Olfactory areas	0	0	0	0
Midbrain reticular nucleus-Pons	0	0	0	0
Nucleus accumbens-Pons	0	0	0	0
fimbria-Pons	0	0	0	0
Anterior cingulate area-Pons	0	0	0	0
Somatomotor areas-Pons	0	0	0	0
Somatosensory areas-Pons	-0.00777	-0.00532	-0.29721	-0.00265
piriform area-Pons	0	0	0	0
Taenia tecta -Pons	0	0	0	0
Accessory olfactory bulb glomerular layer-Pons	0	0	0	0
Accessory olfactory bulb granular layer-Pons	0	0	0	0
Retrohippocampal region -Pons	0	0	0	0
Entorhinal area-Pons	0	0	0	0
Field CA1-Pons	0	0	0	0
Field CA3-Pons	0	0	0	0
Dentate gyrus-Pons	0	0	0	0
Field CA2 -Pons	0	0	0	0
Accessory olfactory bulb mitral layer-Pons	0	0	0	0
Striatum -Pons	0	0	0	0
Midbrain -Pons	0	0	0	0
Medulla-Pons	0	0	0	0
Nucleus accumbens-Midbrain reticular nucleus	0	0	0	0
fimbria-Midbrain reticular nucleus	0	0	0	0
Anterior cingulate area-Midbrain reticular nucleus	0	0	0	0
Somatomotor areas-Midbrain reticular nucleus	0	0	0	0
Somatosensory areas-Midbrain reticular nucleus	-0.15159	0	0	0
piriform area-Midbrain reticular nucleus	0	0	0	0
Taenia tecta -Midbrain reticular nucleus	0	0	0	0
Accessory olfactory bulb glomerular layer-Midbrain reticular nucleus	0	0	0	0
Accessory olfactory bulb granular layer-Midbrain reticular nucleus	0	0	0	0

Retrohippocampal region -Midbrain reticular nucleus	0	0	0	0
Entorhinal area-Midbrain reticular nucleus	0	0	0	0
Field CA1-Midbrain reticular nucleus	0	0	0	0
Field CA3-Midbrain reticular nucleus	0	0	0	0
Dentate gyrus-Midbrain reticular nucleus	0	0	0	0
Field CA2 -Midbrain reticular nucleus	0	0	0	0
Accessory olfactory bulb mitral layer-Midbrain reticular nucleus	0	0	0	0
Striatum -Midbrain reticular nucleus	0	0	0	0
Midbrain -Midbrain reticular nucleus	0	0	0	0
Medulla-Midbrain reticular nucleus	0	0	0	0
fimbria-Nucleus accumbens	0	0	0	0
Anterior cingulate area-Nucleus accumbens	0	0	0	0
Somatomotor areas-Nucleus accumbens	0	0	0	0
Somatosensory areas-Nucleus accumbens	-0.22998	-0.81751	0	-0.09095
piriform area-Nucleus accumbens	0	0	0	0
Taenia tecta -Nucleus accumbens	0	0	0	0
Accessory olfactory bulb glomerular layer-Nucleus accumbens	0	0	0	0
Accessory olfactory bulb granular layer-Nucleus accumbens	0	0	0	0
Retrohippocampal region -Nucleus accumbens	0	0	0	0
Entorhinal area-Nucleus accumbens	0	0	0	0
Field CA1-Nucleus accumbens	0	0	0	0
Field CA3-Nucleus accumbens	0	0	0	0
Dentate gyrus-Nucleus accumbens	0	0	0	0
Field CA2 -Nucleus accumbens	0	0	0	0
Accessory olfactory bulb mitral layer-Nucleus accumbens	0	0	0	0
Striatum -Nucleus accumbens	0	0	0	0
Midbrain -Nucleus accumbens	0	0	0	0
Medulla-Nucleus accumbens	0	0	0	0
Anterior cingulate area-fimbria	0	0	0	0
Somatomotor areas-fimbria	0	0	0	0
Somatosensory areas-fimbria	0.289782	0	0	-0.80211
piriform area-fimbria	0	0	0	0
Taenia tecta -fimbria	0	0	0	0
Accessory olfactory bulb glomerular layer-fimbria	0	0	0	0

Accessory olfactory bulb granular layer-fimbria	0	0	0	0
Retrohippocampal region -fimbria	0	0	0	0
Entorhinal area-fimbria	0	0	0	0
Field CA1-fimbria	0	0	0	0
Field CA3-fimbria	0	0	0	0
Dentate gyrus-fimbria	0	0	0	0
Field CA2 -fimbria	0	0	0	0
Accessory olfactory bulb mitral layer-fimbria	0	0	0	0
Striatum -fimbria	0	0	0	0
Midbrain -fimbria	0	0	0	0
Medulla-fimbria	0	0	0	0
Somatomotor areas-Anterior cingulate area	0.944873	0	0	-0.41916
Somatosensory areas-Anterior cingulate area	-0.22857	-0.12909	0.382815	-0.147
piriform area-Anterior cingulate area	0	0	0	0
Taenia tecta -Anterior cingulate area	0	0	0	0
Accessory olfactory bulb glomerular layer-Anterior cingulate area	0	0	0	0
Accessory olfactory bulb granular layer-Anterior cingulate area	0	0	0	0
Retrohippocampal region -Anterior cingulate area	0	0	0	0
Entorhinal area-Anterior cingulate area	0	0	0	0
Field CA1-Anterior cingulate area	0	0	0	0
Field CA3-Anterior cingulate area	0	0	0	0
Dentate gyrus-Anterior cingulate area	0	0	0	0
Field CA2 -Anterior cingulate area	0	0	0	0
Accessory olfactory bulb mitral layer-Anterior cingulate area	0	0	0	0
Striatum -Anterior cingulate area	0	0	0	0
Midbrain -Anterior cingulate area	0	0	0	0
Medulla-Anterior cingulate area	0	0	0	0
Somatosensory areas-Somatomotor areas	-0.8677	-0.60309	0.17503	0.458658
piriform area-Somatomotor areas	0.354239	0	0	0.467684
Taenia tecta -Somatomotor areas	0	0	0	0.967221
Accessory olfactory bulb glomerular layer-Somatomotor areas	0	0	0	0
Accessory olfactory bulb granular layer-Somatomotor areas	0	0	0	0
Retrohippocampal region -Somatomotor areas	0	0	0	-0.00587
Entorhinal area-Somatomotor areas	-0.0083	0	0	-0.00288

Field CA1-Somatomotor areas	0	0	0	-0.28843
Field CA3-Somatomotor areas	0	0	0	-0.5497
Dentate gyrus-Somatomotor areas	-0.02734	0	0	-0.09095
Field CA2 -Somatomotor areas	0	0	0	0
Accessory olfactory bulb mitral layer-Somatomotor areas	0	0	0	0
Striatum -Somatomotor areas	-0.23733	-0.29818	0.814954	-0.25467
Midbrain -Somatomotor areas	-0.00777	-0.00321	0	-0.00288
Medulla-Somatomotor areas	0	0	0	-0.000081
piriform area-Somatosensory areas	0.192067	0.571828	0.539913	0.802106
Taenia tecta -Somatosensory areas	-0.7886	0.131824	0.845591	0.78296
Accessory olfactory bulb glomerular layer-Somatosensory areas	0	0	0	0
Accessory olfactory bulb granular layer-Somatosensory areas	0	0	0	0
Retrohippocampal region -Somatosensory areas	-0.50512	-0.01417	-0.54919	-0.00842
Entorhinal area-Somatosensory areas	-0.01874	-0.00321	-0.441	-0.02693
Field CA1-Somatosensory areas	-0.85062	-0.02809	0.440996	-0.5714
Field CA3--Somatosensory areas	0.536856	-0.06984	-0.99595	-0.13122
Dentate gyrus-Somatosensory areas	-0.95078	-0.16058	0.712935	-0.25288
Field CA2 -Somatosensory areas	0.781282	0.816219	0.712935	-0.97412
Accessory olfactory bulb mitral layer-Somatosensory areas	0	0	0	0
Striatum --Somatosensory areas	-0.85065	-0.27729	0.429751	-0.1075
Midbrain --Somatosensory areas	-0.02337	-0.00321	-0.38282	-0.00153
Medulla-Somatosensory areas	-0.00777	-0.07396	-0.42975	-0.02693
Taenia tecta -piriform area	0	0	0	0
Accessory olfactory bulb glomerular layer-piriform area	0	0	0	0
Accessory olfactory bulb granular layer-piriform area	0	0	0	0
Retrohippocampal region -piriform area	0	0	0	0
Entorhinal area-piriform area	0	0	0	0
Field CA1-piriform area	0	0	0	0
Field CA3-piriform area	0	0	0	0
Dentate gyrus-piriform area	0	0	0	0
Field CA2 -piriform area	0	0	0	0
Accessory olfactory bulb mitral layer-piriform area	0	0	0	0
Striatum -piriform area	0	0	0	0
Midbrain -piriform area	0	0	0	0

Medulla-piriform area	0	0	0	0
Accessory olfactory bulb glomerular layer-Taenia tecta	0	0	0	0
Accessory olfactory bulb granular layer-Taenia tecta	0	0	0	0
Retrohippocampal region -Taenia tecta	0	0	0	0
Entorhinal area-Taenia tecta	0	0	0	0
Field CA1-Taenia tecta	0	0	0	0
Field CA3-Taenia tecta	0	0	0	0
Dentate gyrus-Taenia tecta	0	0	0	0
Field CA2 -Taenia tecta	0	0	0	0
Accessory olfactory bulb mitral layer-Taenia tecta	0	0	0	0
Striatum -Taenia tecta	0	0	0	0
Midbrain -Taenia tecta	0	0	0	0
Medulla-Taenia tecta	0	0	0	0
Accessory olfactory bulb granular layer-Accessory olfactory bulb glomerular layer	0	0	0	0
Retrohippocampal region -Accessory olfactory bulb glomerular layer	0	0	0	0
Entorhinal area-Accessory olfactory bulb glomerular layer	0	0	0	0
Field CA1-Accessory olfactory bulb glomerular layer	0	0	0	0
Field CA3-Accessory olfactory bulb glomerular layer	0	0	0	0
Dentate gyrus-Accessory olfactory bulb glomerular layer	0	0	0	0
Field CA2 -Accessory olfactory bulb glomerular layer	0	0	0	0
Accessory olfactory bulb mitral layer-Accessory olfactory bulb glomerular layer	0	0	0	0
Striatum -Accessory olfactory bulb glomerular layer	0	0	0	0
Midbrain -Accessory olfactory bulb glomerular layer	0	0	0	0
Medulla-Accessory olfactory bulb glomerular layer	0	0	0	0
Retrohippocampal region -Accessory olfactory bulb granular layer	0	0	0	0
Entorhinal area-Accessory olfactory bulb granular layer	0	0	0	0
Field CA1-Accessory olfactory bulb granular layer	0	0	0	0
Field CA3-Accessory olfactory bulb granular layer	0	0	0	0
Dentate gyrus-Accessory olfactory bulb granular layer	0	0	0	0
Field CA2 -Accessory olfactory bulb granular layer	0	0	0	0
Accessory olfactory bulb mitral layer-Accessory olfactory bulb granular layer	0	0	0	0
Striatum -Accessory olfactory bulb granular layer	0	0	0	0

Midbrain -Accessory olfactory bulb granular layer	0	0	0	0
Medulla-Accessory olfactory bulb granular layer	0	0	0	0
Entorhinal area-Retrohippocampal region	0	0	0	0
Field CA1-Retrohippocampal region	0	0	0	0
Field CA3-Retrohippocampal region	0	0	0	0
Dentate gyrus-Retrohippocampal region	0	0	0	0
Field CA2 -Retrohippocampal region	0	0	0	0
Accessory olfactory bulb mitral layer-Retrohippocampal region	0	0	0	0
Striatum -Retrohippocampal region	0	0	0	0
Midbrain -Retrohippocampal region	0	0	0	0
Medulla-Retrohippocampal region	0	0	0	0
Field CA1-Entorhinal area	0	0	0	0
Field CA3-Entorhinal area	0	0	0	0
Dentate gyrus-Entorhinal area	0	0	0	0
Field CA2 -Entorhinal area	0	0	0	0
Accessory olfactory bulb mitral layer-Entorhinal area	0	0	0	0
Striatum -Entorhinal area	0	0	0	0
Midbrain -Entorhinal area	0	0	0	0
Medulla-Entorhinal area	0	0	0	0
Field CA3-Field CA1	0	0	0	0
Dentate gyrus-Field CA1	0	0	0	0
Field CA2 -Field CA1	0	0	0	0
Accessory olfactory bulb mitral layer-Field CA1	0	0	0	0
Striatum -Field CA1	0	0	0	0
Midbrain -Field CA1	0	0	0	0
Medulla-Field CA1	0	0	0	0
Dentate gyrus-Field CA3	0	0	0	0
Field CA2 -Field CA3	0	0	0	0
Accessory olfactory bulb mitral layer-Field CA3	0	0	0	0
Striatum -Field CA3	0	0	0	0
Midbrain -Field CA3	0	0	0	0
Medulla-Field CA3	0	0	0	0

Field CA2 -Dentate gyrus	0	0	0	0
Accessory olfactory bulb mitral layer-Dentate gyrus	0	0	0	0
Striatum -Dentate gyrus	0	0	0	0
Midbrain -Dentate gyrus	0	0	0	0
Medulla-Dentate gyrus	0	0	0	0
Accessory olfactory bulb mitral layer-Field CA2	0	0	0	0
Striatum -Field CA2	0	0	0	0
Midbrain -Field CA2	0	0	0	0
Medulla-Field CA2	0	0	0	0
Striatum -Accessory olfactory bulb mitral layer	0	0	0	0
Midbrain -Accessory olfactory bulb mitral layer	0	0	0	0
Medulla-Accessory olfactory bulb mitral layer	0	0	0	0
Midbrain -Striatum	0	0	0	0
Medulla-Striatum	0	0	0	0
Medulla-Midbrain	0	0	0	0

S15. Adjust p values for group comparisons of pair-wised connectivity for IsoCortex.

Region Connections	Isocortex			
	Left Ipsilateral	Left Contralateral	Right Contralateral	Right Ipsilateral
Caudoputamen-corpus callosum	0	-0.00609	-0.01925	-0.05589
anterior commissure olfactory limb-corpus callosum	0	0	0	0
pallidum -corpus callosum	0	-0.00347	-0.02444	-0.01864
internal capsule-corpus callosum	-0.00065	0	0	-0.69819
Thalamus-corpus callosum	0	-0.04005	-0.01661	-0.06326
Cerebellum-corpus callosum	-0.00237	-0.0468	-0.00384	-0.03213
Superior colliculus-corpus callosum	-0.02583	-0.01887	-0.06763	-0.02259
ventricular systems-corpus callosum	-0.01614	-0.00469	-0.00951	-0.03895
Hypothalamus -corpus callosum	-0.18076	-0.00176	-0.00433	-0.01398
Inferior colliculus -corpus callosum	-0.06899	0	-0.31023	-0.09592
periaqueductal gray-corpus callosum	-0.00983	-0.14181	0	-0.03722
Isocortex -corpus callosum	-0.00237	-0.35084	-0.38166	-0.25829
Cortical amygdalar area -corpus callosum	-0.045	0	0	-0.78314
Olfactory areas -corpus callosum	-0.00065	-0.04009	-0.03491	-0.009
Pons-corpus callosum	0.670901	-0.00609	-0.01586	-0.01563
Midbrain reticular nucleus-corpus callosum	0.529102	0	0	0
Nucleus accumbens-corpus callosum	-0.01629	-0.06107	0	-0.03189
fimbria-corpus callosum	-0.00065	0	0	-0.33065
Anterior cingulate area-corpus callosum	-0.01972	-0.29893	-0.00845	-0.03807
Somatomotor areas-corpus callosum	-0.00189	0.573873	-0.11602	0.537449
Somatosensory areas-corpus callosum	0	-0.28921	-0.28851	-0.85851
piriform area-corpus callosum	-0.29907	-0.9657	0.663826	0.07668
Taenia tecta -corpus callosum	0.023197	-0.49677	-0.33073	-0.03873
Accessory olfactory bulb glomerular layer-corpus callosum	-0.53683	0	0	-0.08962
Accessory olfactory bulb granular layer-corpus callosum	0.396376	0	0	0
Retrohippocampal region -corpus callosum	-0.16064	-0.00347	-0.00433	-0.08539
Entorhinal area-corpus callosum	-0.19208	-0.00347	-0.00384	-0.1075
Field CA1-corpus callosum	0	-0.02301	-0.0732	0.84628
Field CA3-corpus callosum	-0.38927	-0.01166	-0.00384	-0.18394
Dentate gyrus-corpus callosum	-0.0053	-0.00176	-0.00384	-0.25829
Field CA2 -corpus callosum	-0.08991	-0.00713	-0.02044	-0.13341
Accessory olfactory bulb mitral layer-corpus callosum	-0.42569	0	0	0

Striatum -corpus callosum	-0.72322	-0.00445	-0.14352	-0.05007
Midbrain -corpus callosum	-0.00542	-0.00469	-0.00384	-0.02339
Medulla-corpus callosum	0	0	-0.03055	-0.10306
anterior commissure olfactory limb-Caudoputamen	-0.001636	0	0	0
pallidum -Caudoputamen	0	-0.04813	-0.064717	-0.070816
internal capsule-Caudoputamen	0	0	0	0
Thalamus-Caudoputamen	0	-0.080607	-0.003839	-0.127916
Cerebellum-Caudoputamen	-0.035725	-0.054034	-0.06179	-0.041694
Superior colliculus-Caudoputamen	0	-0.080607	-0.031109	-0.04388
ventricular systems-Caudoputamen	-0.018285	-0.015606	-0.003839	-0.173406
Hypothalamus -Caudoputamen	-0.05088	-0.018403	-0.190897	-0.015628
Inferior colliculus -Caudoputamen	-0.048785	0	0	0
periaqueductal gray-Caudoputamen	-0.014914	-0.046796	-0.021442	0
Isocortex -Caudoputamen	-0.405541	-0.570642	-0.151533	-0.7719
Cortical amygdalar area -Caudoputamen	0	0	0	0
Olfactory areas -Caudoputamen	-0.001393	-0.285643	-0.003839	-0.157304
Pons-Caudoputamen	-0.871366	0	-0.074232	-0.032916
Midbrain reticular nucleus-Caudoputamen	0.409971	0	0	0
Nucleus accumbens-Caudoputamen	-0.018285	0	0	0
fimbria-Caudoputamen	-0.011155	0	0	0
Anterior cingulate area-Caudoputamen	0	-0.210578	-0.006015	-0.133306
Somatomotor areas-Caudoputamen	0	-0.537612	-0.172058	0.132606
Somatosensory areas-Caudoputamen	0	-0.18123	-0.137826	0.096446
piriform area-Caudoputamen	-0.325356	0.900496	0.663826	0.100102
Taenia tecta -Caudoputamen	0.228237	-0.35084	-0.265002	-0.379827
Accessory olfactory bulb glomerular layer-Caudoputamen	0.238307	0	0	0
Accessory olfactory bulb granular layer-Caudoputamen	0.007867	0	0	0
Retrohippocampal region -Caudoputamen	-0.711155	-0.09813	-0.143522	-0.033999
Entorhinal area-Caudoputamen	0	-0.198689	-0.003839	-0.064379
Field CA1-Caudoputamen	0	-0.197549	-0.115345	-0.378258
Field CA3-Caudoputamen	-0.032212	0	0	-0.607691
Dentate gyrus-Caudoputamen	-0.00502	0	-0.021436	-0.847884
Field CA2 -Caudoputamen	-0.009093	-0.13532	0	-0.055894
Accessory olfactory bulb mitral layer-Caudoputamen	-0.872047	0	0	0
Striatum -Caudoputamen	-0.558683	-0.006093	-0.140489	-0.138039
Midbrain -Caudoputamen	0	-0.00253	-0.06179	-0.007338

Medulla-Caudoputamen	0	0	0	-0.028151
pallidum -anterior commissure olfactory limb	-0.00183	0	0	0
internal capsule-anterior commissure olfactory limb	0	0	0	0
Thalamus-anterior commissure olfactory limb	0	0	0	0
Cerebellum-anterior commissure olfactory limb	0	0	0	0
Superior colliculus-anterior commissure olfactory limb	0	0	0	0
ventricular systems-anterior commissure olfactory limb	0	0	0	0
Hypothalamus -anterior commissure olfactory limb	0	0	0	0
Inferior colliculus -anterior commissure olfactory limb	0	0	0	0
periaqueductal gray-anterior commissure olfactory limb	0	0	0	0
Isocortex -anterior commissure olfactory limb	0	0.296986	0	-0.158073
Cortical amygdalar area -anterior commissure olfactory limb	0	0	0	0
Olfactory areas -anterior commissure olfactory limb	0	0	0	0
Pons-anterior commissure olfactory limb	0.529102	0	0	0
Midbrain reticular nucleus-anterior commissure olfactory limb	0	0	0	0
Nucleus accumbens-anterior commissure olfactory limb	0	0	0	0
fimbria-anterior commissure olfactory limb	0	0	0	0
Anterior cingulate area-anterior commissure olfactory limb	0	0	0	0
Somatomotor areas-anterior commissure olfactory limb	0	0	0	0
Somatosensory areas-anterior commissure olfactory limb	0	0	0	0
piriform area-anterior commissure olfactory limb	0	0	0	0
Taenia tecta -anterior commissure olfactory limb	0	0	0	0
Accessory olfactory bulb glomerular layer-anterior commissure olfactory limb	0	0	0	0
Accessory olfactory bulb granular layer-anterior commissure olfactory limb	0	0	0	0
Retrohippocampal region -anterior commissure olfactory limb	0	0	0	0
Entorhinal area-anterior commissure olfactory limb	0	0	0	-0.212171
Field CA1-anterior commissure olfactory limb	0	0	0	0
Field CA3-anterior commissure olfactory limb	0	0	0	0
Dentate gyrus-anterior commissure olfactory limb	0	0	0	0
Field CA2 -anterior commissure olfactory limb	0	0	0	0
Accessory olfactory bulb mitral layer-anterior commissure olfactory limb	0	0	0	0
Striatum -anterior commissure olfactory limb	0	0	0	0
Midbrain -anterior commissure olfactory limb	0	0	0	0
Medulla-anterior commissure olfactory limb	0	0	0	0

internal capsule-pallidum	0	0	0	0
Thalamus-pallidum	0	0	0	-0.527924
Cerebellum-pallidum	0	0	0	0
Superior colliculus-pallidum	0	0	0	0
ventricular systems-pallidum	0	0	0	-0.128989
Hypothalamus -pallidum	0	0	0	0
Inferior colliculus -pallidum	0	0	0	0
periaqueductal gray-pallidum	0	0	0	0
Isocortex -pallidum	0	-0.35084	-0.45485	-0.041842
Cortical amygdalar area -pallidum	0	0	0	0
Olfactory areas -pallidum	0	0	0	-0.220593
Pons-pallidum	-0.022793	0	0	0
Midbrain reticular nucleus-pallidum	0	0	0	0
Nucleus accumbens-pallidum	-0.009191	0	0	0
fimbria-pallidum	0	0	0	0
AVA-pallidum	0	-0.006093	0	-0.0352
Somatomotor areas-pallidum	0	-0.570642	0	-0.645646
Somatosensory areas-pallidum	0	-0.223373	-0.40717	-0.443071
piriform area-pallidum	0	0.230311	0	0
Taenia tecta -pallidum	-0.396369	0	0	0
Accessory olfactory bulb glomerular layer-pallidum	-0.451739	0	0	0
Accessory olfactory bulb granular layer-pallidum	0	0	0	0
Retrohippocampal region -pallidum	0	0	0	-0.104421
Entorhinal area-pallidum	0	-0.193503	-0.140489	-0.01539
Field CA1-pallidum	0	0	0	-0.158073
Field CA3-pallidum	-0.003852	0	0	0
Dentate gyrus-pallidum	-0.002843	0	0	0
Field CA2 -pallidum	0	0	0	0
Accessory olfactory bulb mitral layer-pallidum	0	0	0	0
Striatum -pallidum	0	-0.295396	0	-0.133306
Midbrain -pallidum	0	0	0	0
Medulla-pallidum	0	0	0	0
Thalamus-internal capsule	0	0	0	0
Cerebellum-internal capsule	0	0	0	0
Superior colliculus-internal capsule	0	0	0	0

ventricular systems-internal capsule	0	0	0	0
Hypothalamus -internal capsule	0	0	0	0
Inferior colliculus -internal capsule	0	0	0	0
periaqueductal gray-internal capsule	0	0	0	0
Isocortex -internal capsule	0	0.300788	-0.372729	-0.63996
Cortical amygdalar area -internal capsule	0	0	0	0
Olfactory areas -internal capsule	0	0	0	0
Pons-internal capsule	-0.017323	0	0	0
Midbrain reticular nucleus-internal capsule	0	0	0	0
Nucleus accumbens-internal capsule	0	0	0	0
fimbria-internal capsule	0	0	0	0
Anterior cingulate area-internal capsule	0	0	0	0
Somatomotor areas-internal capsule	0	0	0	0
Somatosensory areas-internal capsule	0	0	0	0
piriform area-internal capsule	0	0	0	0
Taenia tecta -internal capsule	0	0	0	0
Accessory olfactory bulb glomerular layer-internal capsule	0	0	0	0
Accessory olfactory bulb granular layer-internal capsule	0	0	0	0
Retrohippocampal region -internal capsule	0	0	0	0
Entorhinal area-internal capsule	0	0	0	0
Field CA1-internal capsule	0	0	0	0
Field CA3-internal capsule	0	0	0	0
Dentate gyrus-internal capsule	0	0	0	0
Field CA2 -internal capsule	0	0	0	0
Accessory olfactory bulb mitral layer-internal capsule	0	0	0	0
Striatum -internal capsule	0	0	0	0
Midbrain --internal capsule	0	0	0	0
Medulla-internal capsule	0	0	0	0
Cerebellum-Thalamus	0	0	0	-0.95978
Superior colliculus-Thalamus	0	0.547707	0	-0.56705
ventricular systems-Thalamus	0	-0.33486	-0.41592	-0.03292
Hypothalamus -Thalamus	0	0	0	0
Inferior colliculus -Thalamus	-0.42311	0	0	0
periaqueductal gray-Thalamus	-0.32124	0	0	0
Isocortex -Thalamus	0	-0.27785	-0.87216	-0.08497

Cortical amygdalar area -Thalamus	0	0	0	0
Olfactory areas -Thalamus	0	-0.16914	0	-0.02583
Pons-Thalamus	-0.05095	0	0	0
Midbrain reticular nucleus-Thalamus	0	0	0	0
Nucleus accumbens-Thalamus	-0.08149	0	0	0
fimbria-Thalamus	0	0	0	0
Anterior cingulate area-Thalamus	0	0	0	0
Somatomotor areas-Thalamus	0	-0.39196	0	-0.16221
Somatosensory areas-Thalamus	0	-0.4062	-0.42496	-0.21502
piriform area-Thalamus	0	0.542655	0	0.161843
Taenia tecta -Thalamus	-0.35079	0	0	-0.10248
Accessory olfactory bulb glomerular layer-Thalamus	0.337489	0	0	0
Accessory olfactory bulb granular layer-Thalamus	0.529102	0	0	0
Retrohippocampal region -Thalamus	0	0	-0.4891	-0.17842
Entorhinal area-Thalamus	0	-0.08061	-0.03558	-0.01864
Field CA1-Thalamus	0	0	0	-0.1265
Field CA3-Thalamus	-0.75591	0	0	0
Dentate gyrus-Thalamus	-0.04152	0	0	-0.77559
Field CA2 -Thalamus	-0.38436	0	0	0
Accessory olfactory bulb mitral layer-Thalamus	0	0	0	0
Striatum -Thalamus	0	-0.5404	-0.14049	-0.07668
Midbrain -Thalamus	0	0	0	-0.45048
Medulla-Thalamus	0	0	0	0
Superior colliculus-Cerebellum	0	0	0	-0.75332
ventricular systems-Cerebellum	0	-0.39058	-0.4527	-0.11903
Hypothalamus -Cerebellum	0	0	0	0
Inferior colliculus -Cerebellum	0.223635	0	0	0
periaqueductal gray-Cerebellum	0.692753	0	0	0
Isocortex -Cerebellum	0	-0.59665	-0.91828	0.119025
Cortical amygdalar area -Cerebellum	0	0	0	0
Olfactory areas -Cerebellum	0	-0.05376	0	-0.05085
Pons-Cerebellum	0.223635	0	0	0
Midbrain reticular nucleus-Cerebellum	0	0	0	0
Nucleus accumbens-Cerebellum	0	0	0	0
fimbria-Cerebellum	0	0	0	0

Anterior cingulate area-Cerebellum	0	0	0	-0.07119
Somatomotor areas-Cerebellum	0	-0.04881	0	-0.06119
Somatosensory areas-Cerebellum	0	-0.12196	-0.51858	-0.03895
piriform area-Cerebellum	0	0	0	0.677841
Taenia tecta -Cerebellum	-0.01972	0	0	0
Accessory olfactory bulb glomerular layer-Cerebellum	-0.54403	0	0	0
Accessory olfactory bulb granular layer-Cerebellum	0	0	0	0
Retrohippocampal region -Cerebellum	0	0.542655	0	0.704262
Entorhinal area-Cerebellum	0	-0.72679	-0.17206	0.546804
Field CA1-Cerebellum	0	0	0	0
Field CA3-Cerebellum	0.174939	0	0	0
Dentate gyrus-Cerebellum	0	0	0	0
Field CA2 -Cerebellum	0	0	0	0
Accessory olfactory bulb mitral layer-Cerebellum	0	0	0	0
Striatum -Cerebellum	0	-0.13941	0	-0.05647
Midbrain -Cerebellum	0	0	0	-0.81618
Medulla-Cerebellum	0	0	0	0
ventricular systems-Superior colliculus	0	-0.69364	-0.52335	-0.27355
Hypothalamus -Superior colliculus	0	0	0	-0.26111
Inferior colliculus -Superior colliculus	0	0	0	-0.30791
periaqueductal gray-Superior colliculus	0.615264	0	0	0
Isocortex -Superior colliculus	0.606182	-0.28426	0.667112	-0.62552
Cortical amygdalar area -Superior colliculus	0	0	0	0
Olfactory areas -Superior colliculus	0	-0.17818	-0.13188	-0.01563
Pons-Superior colliculus	0.299065	0	0	0
Midbrain reticular nucleus-Superior colliculus	0	0	0	0
Nucleus accumbens-Superior colliculus	-0.17705	0	0	0
fimbria-Superior colliculus	0	0	0	0
Anterior cingulate area-Superior colliculus	0	-0.12196	0	-0.06533
Somatomotor areas-Superior colliculus	0	-0.29175	-0.56319	-0.08721
Somatosensory areas-Superior colliculus	0	-0.48339	-0.1577	-0.17627
piriform area-Superior colliculus	0	0	0	0
Taenia tecta -Superior colliculus	-0.09435	0	0	-0.03824
Accessory olfactory bulb glomerular layer-Superior colliculus	-0.76499	0	0	0
Accessory olfactory bulb granular layer-Superior colliculus	0	0	0	0

Retrohippocampal region -Superior colliculus	0	0	0.781568	-0.10992
Entorhinal area-Superior colliculus	0	0	-0.32135	-0.09841
Field CA1-Superior colliculus	0	0	-0.84464	-0.16429
Field CA3-Superior colliculus	-0.07762	0	0	-0.13905
Dentate gyrus-Superior colliculus	-0.43402	0	0.440784	-0.16025
Field CA2 -Superior colliculus	-0.44906	0	0	0
Accessory olfactory bulb mitral layer-Superior colliculus	-0.38173	0	0	0
Striatum -Superior colliculus	-0.64962	0	-0.74153	-0.034
Midbrain -Superior colliculus	0	0	-0.90454	-0.05039
Medulla-Superior colliculus	0	0	0	0
Hypothalamus -ventricular systems	-0.197802	0	-0.763602	-0.438199
Inferior colliculus -ventricular systems	0	0	0	0
periaqueductal gray-ventricular systems	0	0	0	0
Isocortex -ventricular systems	-0.032212	-0.762318	0.90454	-0.307906
Cortical amygdalar area -ventricular systems	0	0	0	0
Olfactory areas -ventricular systems	0	-0.035549	-0.01621	-0.01398
Pons-ventricular systems	0.621981	0	0	0
Midbrain reticular nucleus-ventricular systems	0	0	0	0
Nucleus accumbens-ventricular systems	-0.036522	0	0	0
fimbria-ventricular systems	0	0	0	0
Anterior cingulate area-ventricular systems	0	-0.061932	-0.003839	-0.022587
Somatomotor areas-ventricular systems	0	-0.541014	-0.321354	-0.537449
Somatosensory areas-ventricular systems	0	-0.334862	-0.334811	0.645646
piriform area-ventricular systems	-0.00183	0.899225	0.359977	0.379827
Taenia tecta -ventricular systems	-0.201446	-0.197173	-0.116016	-0.01398
Accessory olfactory bulb glomerular layer-ventricular systems	0.621981	0	0	0
Accessory olfactory bulb granular layer-ventricular systems	0.850247	0	0	0
Retrohippocampal region -ventricular systems	-0.325356	-0.019341	-0.05358	-0.154742
Entorhinal area-ventricular systems	0	-0.00177	-0.021436	-0.028151
Field CA1-ventricular systems	0	0	-0.217079	-0.033999
Field CA3-ventricular systems	-0.451739	0	-0.229287	-0.499476
Dentate gyrus-ventricular systems	-0.018285	-0.462993	0.978448	-0.867753
Field CA2 -ventricular systems	-0.297196	0	0	0
Accessory olfactory bulb mitral layer-ventricular systems	0.973493	0	0	0
Striatum -ventricular systems	0.665084	-0.004687	-0.03342	-0.018641

Midbrain -ventricular systems	0	-0.09813	-0.74908	-0.100022
Medulla-ventricular systems	0	0	0	0
Inferior colliculus -Hypothalamus	-0.343006	0	0	0
periaqueductal gray-Hypothalamus	0	0	0	0
Isocortex -Hypothalamus	0	-0.169402	-0.277985	-0.03213
Cortical amygdalar area -Hypothalamus	0	0	0	0
Olfactory areas -Hypothalamus	0	-0.082522	0	-0.05007
Pons-Hypothalamus	-0.002368	0	0	0
Midbrain reticular nucleus-Hypothalamus	0	0	0	0
Nucleus accumbens-Hypothalamus	-0.023197	0	0	0
fimbria-Hypothalamus	0	0	0	0
Anterior cingulate area-Hypothalamus	0	0	0	-0.10586
Somatomotor areas-Hypothalamus	0	-0.221463	0	-0.139047
Somatosensory areas-Hypothalamus	0	-0.719741	-0.115345	-0.186109
piriform area-Hypothalamus	0	0.462993	0	0
Taenia tecta -Hypothalamus	-0.024101	0	0	0
Accessory olfactory bulb glomerular layer-Hypothalamus	-0.304139	0	0	0
Accessory olfactory bulb granular layer-Hypothalamus	0.353267	0	0	0
Retrohippocampal region -Hypothalamus	0	0	0	-0.27489
Entorhinal area-Hypothalamus	0	-0.35084	-0.073195	-0.017408
Field CA1-Hypothalamus	0	0	0	-0.420055
Field CA3-Hypothalamus	-0.280839	0	0	0
Dentate gyrus-Hypothalamus	-0.012746	0	0	0
Field CA2 -Hypothalamus	0	0	0	0
Accessory olfactory bulb mitral layer-Hypothalamus	0	0	0	0
Striatum -Hypothalamus	0	0	0	-0.084974
Midbrain -Hypothalamus	0	0	0	0
Medulla-Hypothalamus	0	0	0	0
periaqueductal gray-Inferior colliculus	0	0	0	0
Isocortex -Inferior colliculus	0	-0.50022	-0.11535	-0.30791
Cortical amygdalar area -Inferior colliculus	0	0	0	0
Olfactory areas -Inferior colliculus	0	0	0	0
Pons-Inferior colliculus	-0.08084	0	0	0
Midbrain reticular nucleus-Inferior colliculus	0	0	0	0

Nucleus accumbens-Inferior colliculus	0	0	0	0
fimbria-Inferior colliculus	0	0	0	0
Anterior cingulate area-Inferior colliculus	0	0	0	0
Somatomotor areas-Inferior colliculus	0	0	0	0
Somatosensory areas-Inferior colliculus	0	0	0	-0.13331
piriform area-Inferior colliculus	0	0	0	0
Taenia tecta -Inferior colliculus	0	0	0	0
Accessory olfactory bulb glomerular layer-Inferior colliculus	-0.43402	0	0	0
Accessory olfactory bulb granular layer-Inferior colliculus	0	0	0	0
Retrohippocampal region -Inferior colliculus	0	0	0	0.940367
Entorhinal area-Inferior colliculus	0	0	0	0.606442
Field CA1-Inferior colliculus	0	0	0	0
Field CA3-Inferior colliculus	0	0	0	0
Dentate gyrus-Inferior colliculus	0	0	0	0
Field CA2 -Inferior colliculus	0	0	0	0
Accessory olfactory bulb mitral layer-Inferior colliculus	0	0	0	0
Striatum -Inferior colliculus	0	0	0	0
Midbrain -Inferior colliculus	0	0	0	0
Medulla-Inferior colliculus	0	0	0	0
Isocortex -periaqueductal gray	0	-0.01273	-0.34137	-0.02178
Cortical amygdalar area -periaqueductal gray	0	0	0	0
Olfactory areas -periaqueductal gray	0	0	0	0
Pons-periaqueductal gray	-0.00348	0	0	0
Midbrain reticular nucleus-periaqueductal gray	0	0	0	0
Nucleus accumbens-periaqueductal gray	0	0	0	0
fimbria-periaqueductal gray	0	0	0	0
Anterior cingulate area-periaqueductal gray	0	0	0	0
Somatomotor areas-periaqueductal gray	0	0	0	0
Somatosensory areas-periaqueductal gray	0	-0.07266	-0.32135	0
piriform area-periaqueductal gray	0	0	0	0
Taenia tecta -periaqueductal gray	-0.01946	0	0	0
Accessory olfactory bulb glomerular layer-periaqueductal gray	-0.0913	0	0	0
Accessory olfactory bulb granular layer-periaqueductal gray	0	0	0	0
Retrohippocampal region -periaqueductal gray	0	0	0	0
Entorhinal area-periaqueductal gray	0	0	0	0

Field CA1-periaqueductal gray	0	0	0	0
Field CA3-periaqueductal gray	0	0	0	0
Dentate gyrus-periaqueductal gray	0	0	0	0
Field CA2 -periaqueductal gray	0	0	0	0
Accessory olfactory bulb mitral layer-periaqueductal gray	0	0	0	0
Striatum -periaqueductal gray	0	0	0	0
Midbrain -periaqueductal gray	0	0	0	0
Medulla-periaqueductal gray	0	0	0	0
Cortical amygdalar area -Isocortex	0	-0.07798	-0.17206	0.546804
Olfactory areas -Isocortex	0	-0.02627	0.978448	-0.01563
Pons-Isocortex	0	-0.00609	-0.01017	-0.02142
Midbrain reticular nucleus-Isocortex	0.033644	-0.01145	-0.03055	-0.03824
Nucleus accumbens-Isocortex	-0.32061	-0.05376	0.981244	-0.13804
fimbria-Ictx	-0.00284	-0.17818	-0.0174	-0.50421
Anterior cingulate area-Isocortex	-0.00578	-0.20437	-0.32135	-0.14454
Somatomotor areas-Isocortex	-0.06528	-0.94946	-0.97845	0.15394
Somatosensory areas-Isocortex	0.343006	-0.19717	-0.81957	0.015628
piriform area-Isocortex	-0.22526	-0.26404	0.741532	0.033999
Taenia tecta -Isocortex	0.051466	-0.28564	0.819565	-0.12792
Accessory olfactory bulb glomerular layer-Isocortex	0.391269	0	-0.70966	-0.86847
Accessory olfactory bulb granular layer-Isocortex	0.000737	0	0	-0.05085
Retrohippocampal region -Isocortex	0.455658	-0.01561	-0.10281	0.035061
Entorhinal area-Isocortex	0.325356	-0.01145	-0.06179	0.7719
Field CA1-Isocortex	-0.32124	-0.03203	-0.25862	0.06652
Field CA3-Isocortex	0.542941	-0.02176	-0.18873	0.211761
Dentate gyrus-Isocortex	-0.70023	-0.05998	0.792982	0.07668
Field CA2 -Isocortex	0.040893	-0.16932	-0.90454	0.220593
Accessory olfactory bulb mitral layer-Isocortex	0.13842	0	0	-0.70316
Striatum -Isocortex	0.073899	-0.01455	-0.81957	-0.30791
Midbrain -Isocortex	0.136574	-0.00645	-0.0137	-0.04799
Medulla-Isocortex	-0.94265	-0.00609	-0.17206	-0.03917
Olfactory areas -Cortical amygdalar area	-0.00304	0	0	0
Pons-Cortical amygdalar area	-0.01922	0	0	0
Midbrain reticular nucleus-Cortical amygdalar area	0	0	0	0

Nucleus accumbens-Cortical amygdalar area	0	0	0	0
fimbria-Cortical amygdalar area	0	0	0	0
Anterior cingulate area-Cortical amygdalar area	0	0	0	0
Somatomotor areas-Cortical amygdalar area	0	0	0	-0.07119
Somatosensory areas-Cortical amygdalar area	0	-0.37344	0	-0.85036
piriform area-Cortical amygdalar area	0	0	0	0
Taenia tecta -Cortical amygdalar area	0	0	0	0
Accessory olfactory bulb glomerular layer-Cortical amygdalar area	0.129212	0	0	0
Accessory olfactory bulb granular layer-Cortical amygdalar area	0	0	0	0
Retrohippocampal region -Cortical amygdalar area	0	0	0	0
Entorhinal area-Cortical amygdalar area	0	0	0	0.202695
Field CA1-Cortical amygdalar area	0	0	0	0
Field CA3-Cortical amygdalar area	0	0	0	0
Dentate gyrus-Cortical amygdalar area	0.009191	0	0	0
Field CA2 -Cortical amygdalar area	0	0	0	0
Accessory olfactory bulb mitral layer-Cortical amygdalar area	0	0	0	0
Striatum -Cortical amygdalar area	0	0	0	0
Midbrain -Cortical amygdalar area	0	0	0	0
Medulla-Cortical amygdalar area	0	0	0	0
Pons-Olfactory areas	0	0	0	0
Midbrain reticular nucleus-Olfactory areas	0	0	0	0
Nucleus accumbens-Olfactory areas	0	0	0	-0.06119
fimbria-Olfactory areas	0	0	0	0
Anterior cingulate area-Olfactory areas	0	-0.43304	-0.04126	-0.08849
Somatomotor areas-Olfactory areas	-0.05311	0.416863	0.310233	0.0352
Somatosensory areas-Olfactory areas	0	-0.0492	-0.85127	-0.42158
piriform area-Olfactory areas	-0.97349	-0.94946	0.563191	-0.04955
Taenia tecta -Olfactory areas	0.00502	-0.10646	-0.54258	-0.03824
Accessory olfactory bulb glomerular layer-Olfactory areas	-0.18714	0	0	-0.06828
Accessory olfactory bulb granular layer-Olfactory areas	-0.02631	0	0	-0.97599
Retrohippocampal region -Olfactory areas	-0.33749	-0.16914	-0.1909	-0.0352
Entorhinal area-Olfactory areas	-0.07246	-0.07798	-0.02698	-0.01864
Field CA1-Olfactory areas	-0.23083	-0.13878	-0.07423	-0.01563
Field CA3-Olfactory areas	-0.01924	0	-0.15153	-0.12815
Dentate gyrus-Olfactory areas	-0.00923	-0.09813	-0.06565	-0.03807

Field CA2 -Olfactory areas	-0.17601	0	0	-0.10611
Accessory olfactory bulb mitral layer-Olfactory areas	-0.18129	0	0	-0.1813
Striatum -Olfactory areas	-0.07762	-0.01445	-0.3897	-0.67594
Midbrain -Olfactory areas	0	-0.14113	0	-0.01864
Medulla-Olfactory areas	-0.18825	0	0	0
Midbrain reticular nucleus-Pons	-0.08091	0	0	0
Nucleus accumbens-Pons	0	0	0	0
fimbria-Pons	0	0	0	0
Anterior cingulate area-Pons	0	0	0	0
Somatomotor areas-Pons	0	0	0	0
Somatosensory areas-Pons	0	-0.09236	0	-0.10218
piriform area-Pons	0	0	0	0
Taenia tecta -Pons	-0.03012	0	0	0
Accessory olfactory bulb glomerular layer-Pons	0	0	0	0
Accessory olfactory bulb granular layer-Pons	0	0	0	0
Retrohippocampal region -Pons	0	0	0	0
Entorhinal area-Pons	0	0	0	0
Field CA1-Pons	0	0	0	0
Field CA3-Pons	0	0	0	0
Dentate gyrus-Pons	0	0	0	0
Field CA2 -Pons	0	0	0	0
Accessory olfactory bulb mitral layer-Pons	0	0	0	0
Striatum -Pons	0	0	0	0
Midbrain -Pons	0	0	0	0
Medulla-Pons	0	0	0	0
Nucleus accumbens-Midbrain reticular nucleus	0	0	0	0
fimbria-Midbrain reticular nucleus	0	0	0	0
Anterior cingulate area-Midbrain reticular nucleus	0	0	0	0
Somatomotor areas-Midbrain reticular nucleus	0	0	0	0
Somatosensory areas-Midbrain reticular nucleus	0	0	0	0
piriform area-Midbrain reticular nucleus	0	0	0	0
Taenia tecta -Midbrain reticular nucleus	0	0	0	0
Accessory olfactory bulb glomerular layer-Midbrain reticular nucleus	0	0	0	0
Accessory olfactory bulb granular layer-Midbrain reticular nucleus	0	0	0	0

Retrohippocampal region -Midbrain reticular nucleus	0	0	0	0
Entorhinal area-Midbrain reticular nucleus	0	0	0	0
Field CA1-Midbrain reticular nucleus	0	0	0	0
Field CA3-Midbrain reticular nucleus	0	0	0	0
Dentate gyrus-Midbrain reticular nucleus	0	0	0	0
Field CA2 -Midbrain reticular nucleus	0	0	0	0
Accessory olfactory bulb mitral layer-Midbrain reticular nucleus	0	0	0	0
Striatum -Midbrain reticular nucleus	0	0	0	0
Midbrain -Midbrain reticular nucleus	0	0	0	0
Medulla-Midbrain reticular nucleus	0	0	0	0
fimbria-Nucleus accumbens	0	0	0	0
Anterior cingulate area-Nucleus accumbens	0	0	0	-0.06966
Somatomotor areas-Nucleus accumbens	0	0	0	-0.64565
Somatosensory areas-Nucleus accumbens	0	0	0	-0.59605
piriform area-Nucleus accumbens	0.819444	0	0	0
Taenia tecta -Nucleus accumbens	0.081576	0	0	0
Accessory olfactory bulb glomerular layer-Nucleus accumbens	-0.86006	0	0	0
Accessory olfactory bulb granular layer-Nucleus accumbens	0	0	0	0
Retrohippocampal region -Nucleus accumbens	0	0	0	-0.21176
Entorhinal area-Nucleus accumbens	0	0	0	-0.01398
Field CA1-Nucleus accumbens	0	0	0	0
Field CA3-Nucleus accumbens	0	0	0	0
Dentate gyrus-Nucleus accumbens	-0.03221	0	0	0
Field CA2 -Nucleus accumbens	0	0	0	0
Accessory olfactory bulb mitral layer-Nucleus accumbens	0	0	0	0
Striatum -Nucleus accumbens	0	0	0	0
Midbrain -Nucleus accumbens	0	0	0	0
Medulla-Nucleus accumbens	0	0	0	0
Anterior cingulate area-fimbria	0	0	0	0
Somatomotor areas-fimbria	0	0	0	0
Somatosensory areas-fimbria	0	0	0	0
piriform area-fimbria	0	0	0	0
Taenia tecta -fimbria	0	0	0	0
Accessory olfactory bulb glomerular layer-fimbria	0	0	0	0

Accessory olfactory bulb granular layer-fimbria	0	0	0	0
Retrohippocampal region -fimbria	0	0	0	0
Entorhinal area-fimbria	0	0	0	-0.03895
Field CA1-fimbria	0	0	0	0
Field CA3-fimbria	0	0	0	0
Dentate gyrus-fimbria	0	0	0	0
Field CA2 -fimbria	0	0	0	0
Accessory olfactory bulb mitral layer-fimbria	0	0	0	0
Striatum -fimbria	0	0	0	0
Midbrain -fimbria	0	0	0	0
Medulla-fimbria	0	0	0	0
Somatomotor areas-Anterior cingulate area	0	0.116995	0.527625	0.976734
Somatosensory areas-Anterior cingulate area	0	-0.04498	0.763602	-0.45103
piriform area-Anterior cingulate area	0	0	0.45485	-0.70316
Taenia tecta -Anterior cingulate area	0.019239	-0.04881	-0.76438	-0.13202
Accessory olfactory bulb glomerular layer-Anterior cingulate area	-0.18825	0	0	0
Accessory olfactory bulb granular layer-Anterior cingulate area	-0.9303	0	0	0
Retrohippocampal region -Anterior cingulate area	0.990459	0	0	-0.04039
Entorhinal area-Anterior cingulate area	0	-0.00744	-0.02144	-0.04388
Field CA1-Anterior cingulate area	0	0	0	-0.29114
Field CA3-Anterior cingulate area	0	0	0	-0.13331
Dentate gyrus-Anterior cingulate area	-0.00324	0	0	-0.1787
Field CA2 -Anterior cingulate area	0	0	0	0
Accessory olfactory bulb mitral layer-Anterior cingulate area	0	0	0	0
Striatum -Anterior cingulate area	0	-0.0304	-0.53096	-0.37983
Midbrain -Anterior cingulate area	0	-0.00405	-0.10989	-0.10068
Medulla-Anterior cingulate area	0	0	0	-0.03213
Somatosensory areas-Somatomotor areas	0	-0.08061	0.321354	0.100322
piriform area-Somatomotor areas	0	0.931566	0	0.096245
Taenia tecta -Somatomotor areas	0	0.070112	0.079126	0.038241
Accessory olfactory bulb glomerular layer-Somatomotor areas	0.74136	0	0	0.044893
Accessory olfactory bulb granular layer-Somatomotor areas	0.02536	0	0	0
Retrohippocampal region -Somatomotor areas	0.005372	0	0	-0.08438
Entorhinal area-Somatomotor areas	0.009093	0	-0.1909	-0.0352

Field CA1-Somatomotor areas	0	0	-0.51858	-0.86847
Field CA3-Somatomotor areas	-0.00787	0	-0.53744	-0.37021
Dentate gyrus-Somatomotor areas	-0.00284	0	0	-0.63996
Field CA2 -Somatomotor areas	0	0	0.151814	0
Accessory olfactory bulb mitral layer-Somatomotor areas	0	0	0	0.040391
Striatum -Somatomotor areas	-0.02165	-0.16914	0.321354	0.139047
Midbrain -Somatomotor areas	0.343006	-0.04813	0	-0.02583
Medulla-Somatomotor areas	0.004538	0	0	0
piriform area-Somatosensory areas	-0.00183	0.264038	0.197812	0.028151
Taenia tecta -Somatosensory areas	-0.01621	0.158279	-0.22507	0.139047
Accessory olfactory bulb glomerular layer-Somatosensory areas	0	0	0	0
Accessory olfactory bulb granular layer-Somatosensory areas	0.004296	0	0	0
Retrohippocampal region -Somatosensory areas	0.858574	-0.29893	-0.21708	-0.15807
Entorhinal area-Somatosensory areas	0	-0.0073	-0.11602	-0.17341
Field CA1-Somatosensory areas	0	0	0.490035	0.047495
Field CA3--Somatosensory areas	-0.67617	0	-0.45573	0.191613
Dentate gyrus-Somatosensory areas	-0.25245	-0.35084	0.90454	0.409256
Field CA2 -Somatosensory areas	0.019239	0	-0.90454	0.539204
Accessory olfactory bulb mitral layer-Somatosensory areas	0.049582	0	0	0
Striatum --Somatosensory areas	0.113044	-0.16486	-0.45485	0.624701
Midbrain --Somatosensory areas	0.189997	-0.02318	-0.14049	-0.13905
Medulla-Somatosensory areas	0	0	0	0
Taenia tecta -piriform area	-0.13166	0.046796	0	-0.36836
Accessory olfactory bulb glomerular layer-piriform area	-0.04958	0	0	-0.59605
Accessory olfactory bulb granular layer-piriform area	0	0	0	0
Retrohippocampal region -piriform area	-0.17494	0	0	0.176478
Entorhinal area-piriform area	-0.18994	0	-0.24457	0.842327
Field CA1-piriform area	0	0	-0.34577	0.291144
Field CA3-piriform area	0.928325	0	0	0.073278
Dentate gyrus-piriform area	-0.18825	0	0.566167	0.032805
Field CA2 -piriform area	0.160764	0	0	0.368362
Accessory olfactory bulb mitral layer-piriform area	0.116068	0	0	-0.05007
Striatum -piriform area	-0.81072	0.463101	0.819565	0.138039
Midbrain -piriform area	0.223635	0	0	0

Medulla-piriform area	0	0	0	0
Accessory olfactory bulb glomerular layer-Taenia tecta	0	0	0	0
Accessory olfactory bulb granular layer-Taenia tecta	0	0	0	0
Retrohippocampal region -Taenia tecta	0	0	0	-0.22813
Entorhinal area-Taenia tecta	0	-0.16464	-0.01621	-0.02354
Field CA1-Taenia tecta	0	0	0	-0.13905
Field CA3-Taenia tecta	-0.06528	0	0	0
Dentate gyrus-Taenia tecta	-0.00183	0	0	-0.45103
Field CA2 -Taenia tecta	0	0	0	0
Accessory olfactory bulb mitral layer-Taenia tecta	0	0	0	0
Striatum -Taenia tecta	0	-0.02309	-0.45485	-0.90604
Midbrain -Taenia tecta	0	0	0	-0.12899
Medulla-Taenia tecta	0	0	0	0
Accessory olfactory bulb granular layer-Accessory olfactory bulb glomerular layer	0	0	0	0
Retrohippocampal region -Accessory olfactory bulb glomerular layer	0	0	0	0
Entorhinal area-Accessory olfactory bulb glomerular layer	0	0	0	0
Field CA1-Accessory olfactory bulb glomerular layer	0	0	0	0
Field CA3-Accessory olfactory bulb glomerular layer	0	0	0	0
Dentate gyrus-Accessory olfactory bulb glomerular layer	0	0	0	0
Field CA2 -Accessory olfactory bulb glomerular layer	0	0	0	0
Accessory olfactory bulb mitral layer-Accessory olfactory bulb glomerular layer	0	0	0	0
Striatum -Accessory olfactory bulb glomerular layer	0	0	0	0
Midbrain -Accessory olfactory bulb glomerular layer	0	0	0	0
Medulla-Accessory olfactory bulb glomerular layer	0	0	0	0
Retrohippocampal region -Accessory olfactory bulb granular layer	0	0	0	0
Entorhinal area-Accessory olfactory bulb granular layer	0	0	0	0
Field CA1-Accessory olfactory bulb granular layer	0	0	0	0
Field CA3-Accessory olfactory bulb granular layer	0	0	0	0
Dentate gyrus-Accessory olfactory bulb granular layer	0	0	0	0
Field CA2 -Accessory olfactory bulb granular layer	0	0	0	0
Accessory olfactory bulb mitral layer-Accessory olfactory bulb granular layer	0	0	0	0
Striatum -Accessory olfactory bulb granular layer	0	0	0	0

Midbrain -Accessory olfactory bulb granular layer	0	0	0	0
Medulla-Accessory olfactory bulb granular layer	0	0	0	0
Entorhinal area-Retrohippocampal region	0	-0.07581	-0.01476	0.447046
Field CA1-Retrohippocampal region	0	0	-0.32135	-0.77127
Field CA3-Retrohippocampal region	0	0	0	-0.13072
Dentate gyrus-Retrohippocampal region	-0.39638	0	0	-0.07668
Field CA2 -Retrohippocampal region	-0.20113	0	0	-0.09841
Accessory olfactory bulb mitral layer-Retrohippocampal region	-0.40997	0	0	0
Striatum -Retrohippocampal region	0.425694	-0.15604	-0.12264	-0.05589
Midbrain -Retrohippocampal region	0	0	0	-0.02259
Medulla-Retrohippocampal region	0	0	0	0
Field CA1-Entorhinal area	-0.41712	-0.00645	-0.0098	0.992993
Field CA3-Entorhinal area	0	0	0	-0.23254
Dentate gyrus-Entorhinal area	0	-0.0676	-0.07467	-0.64845
Field CA2 -Entorhinal area	-0.75591	-0.07684	0	-0.30791
Accessory olfactory bulb mitral layer-Entorhinal area	-0.01614	0	0	0
Striatum -Entorhinal area	-0.02227	-0.00347	-0.06472	-0.02178
Midbrain -Entorhinal area	0	-0.01854	0	-0.03213
Medulla-Entorhinal area	0	0	0	0
Field CA3-Field CA1	0	0	0	0
Dentate gyrus-Field CA1	0	0	0	-0.16855
Field CA2 -Field CA1	0	0	0	0
Accessory olfactory bulb mitral layer-Field CA1	0	0	0	0
Striatum -Field CA1	0	0	0	-0.03213
Midbrain -Field CA1	0	0	0	-0.44923
Medulla-Field CA1	0	0	0	0
Dentate gyrus-Field CA3	-0.33749	0	0	0
Field CA2 -Field CA3	0	0	0	0
Accessory olfactory bulb mitral layer-Field CA3	0	0	0	0
Striatum -Field CA3	0	0	0	0
Midbrain -Field CA3	0	0	0	-0.12185
Medulla-Field CA3	0	0	0	0

Field CA2 -Dentate gyrus	0	0	0	0
Accessory olfactory bulb mitral layer-Dentate gyrus	0	0	0	0
Striatum -Dentate gyrus	0	-0.19717	0	-0.16025
Midbrain -Dentate gyrus	0	0	0	-0.1787
Medulla-Dentate gyrus	0	0	0	0
Accessory olfactory bulb mitral layer-Field CA2	0	0	0	0
Striatum -Field CA2	0	0	0	0
Midbrain -Field CA2	0	0	0	0
Medulla-Field CA2	0	0	0	0
Striatum -Accessory olfactory bulb mitral layer	0	0	0	0
Midbrain -Accessory olfactory bulb mitral layer	0	0	0	0
Medulla-Accessory olfactory bulb mitral layer	0	0	0	0
Midbrain -Striatum	0	-0.01145	-0.17206	-0.02815
Medulla-Striatum	0	0	0	0
Medulla-Midbrain	-0.01266	0	0	0
